# Physiotherapy and related management for childhood obesity: A systematic scoping review

**DOI:** 10.1371/journal.pone.0252572

**Published:** 2021-06-14

**Authors:** Kim Truong, Sandra Park, Margarita D. Tsiros, Nikki Milne

**Affiliations:** 1 Faculty of Health Sciences and Medicine, Bond Institute of Health and Sport, Bond University, Gold Coast, Queensland, Australia; 2 UniSA Allied Health and Human Performance, Alliance for Research in Exercise, Nutrition and Activity, University of South Australia, Adelaide, South Australia, Australia; Universitat de les Illes Balears, SPAIN

## Abstract

**Introduction:**

Despite targeted efforts globally to address childhood overweight/obesity, it remains poorly understood and challenging to manage. Physiotherapists have the potential to manage children with obesity as they are experts in movement and physical activity. However, their role remains unclear due to a lack of physiotherapy-specific guidelines. This scoping review aims to explore existing literature, critically appraising and synthesising findings to guide physiotherapists in the evidence-based management of childhood overweight/obesity.

**Method:**

A scoping review was conducted, including literature up to May 2020. A review protocol exists on Open Science Framework at https://osf.io/fap8g/. Four databases were accessed including PubMed, Embase, CINAHL, Medline via OVID, with grey literature searched through google via “file:pdf”. A descriptive synthesis was undertaken to explore the impact of existing interventions and their efficacy.

**Results:**

From the initial capture of 1871 articles, 263 intervention-based articles were included. Interventions included qualitative focused physical activity, quantitative focused physical activity and multicomponent interventions. Various outcome measures were utilised including health-, performance- and behaviour-related outcomes. The general trend for physiotherapy involvement with children who are obese appears to favour: 1) multicomponent interventions, implementing more than one component with environmental modification and parental involvement and 2) quantitative physical activity interventions, focusing on the quantity of bodily movement. These approaches most consistently demonstrated desirable changes across behavioural and health-related outcome measures for multicomponent and quantitative physical activity interventions respectively.

**Conclusion:**

When managing children with obesity, physiotherapists should consider multicomponent approaches and increasing the quantity of physical activity, given consistent improvements in various obesity-related outcomes. Such approaches are well suited to the scope of physiotherapists and their expertise in physical activity prescription for the management of childhood obesity. Future research should examine the effect of motor skill interventions and consider the role of environmental modification/parental involvement as factors contributing to intervention success.

## Introduction

Over 340 million children worldwide are classified as overweight or obese [1]. There are no reports in the empirical literature to demonstrate that any country has been successful with significantly decreasing obesity rates in the last three decades [2].

Obesity is strongly related to both short- and long-term co-morbidities [3]. Common conditions associated with obesity include type 2 diabetes mellitus, hypertension, early onset of puberty, menstrual irregularities, polycystic ovarian syndrome, steatohepatitis, sleep apnoea, asthma, benign intracranial hypertension, musculoskeletal disorders and psychological problems [3]. If obesity is not addressed appropriately during childhood, it can lead to further comorbidities such as a greater risk of health complications in adulthood [4] and increased cardiovascular-related mortality [5–7]. Hence, early interventions for preventing and managing obesity in childhood are important in the prevention of further chronic disease, morbidity or mortality.

Childhood obesity interventions are commonly based on the concept of energy balance [8]. Energy balance is equivalent to energy intake, minus energy expenditure. Based on this theory, if energy intake is greater than energy expenditure, it results in excess adiposity. Currently, two common approaches to obesity management involve either, or a combination of both; decreasing energy intake through nutritional education and healthy eating, or increasing energy expenditure through physical activity [8]. Conversely, Flatt et al. highlight that obesity management is not simplistic but rather multifactorial in nature [9]. Childhood obesity may be influenced by age, gender, genetics, psychological and environmental factors such as school policies, parent’s work-related demands and lifestyle [10].

Due to the complexity of obesity, a multicomponent behavioural intervention carried out by a multidisciplinary team is considered best practice and has been shown to be effective [11–15]. Furthermore, Rajjo et al. [16] suggested that physical activity, behavioural, pharmacological, dietary and educational components, with an early intervention approach have a positive impact on weight and healthy lifestyle behaviours. However, the nature of these research-based interventions has limitations regarding the generalisability to wider populations in real-life contexts [16]. The interventions are usually tested in unblinded trials [16]. Additionally, many interventions being used clinically to address child obesity in real world settings are multifaceted and reactive to individual needs. Therefore, it is challenging to replicate these interventions in research in order to acquire evidence to support practice guidelines [16].

Some evidence suggests that childhood obesity may be successfully treated with enhanced fundamental movement skills, motor coordination and physical activity [17]. This field of research is commonly based on the assumption that children with well-developed fundamental motor skills are more likely to engage in high levels of physical activity than those with poorly developed functional motor skills [17]. Since children and adolescents with obesity have lower coordination, balance, speed, agility and fine and gross motor skills compared to their healthy-weight peers [17], they are often unable to meet the physical activity recommendations and unable to reap the benefits that physical activity offers [18–20]. Physiotherapists play a major role in improving functional motor skills and enhancing physical activity in children with overweight or obesity [21]. However, no previous reviews have explored physiotherapy specific interventions for managing or preventing child obesity. Physiotherapy interventions may be focused on increasing participation in physical activities or improving the quality of movement in physical activity.

In a recently published ‘call-to-action’ for physical therapists, Tsiros and Shultz [22], proposed “Ten Action Points” whereby physiotherapists are encouraged to be aware of physical activity and healthy eating guidelines to set measurable goals around family-level lifestyle behaviours instead of making weight the sole focus of assessment and intervention [22]. Additionally, the National Health and Medical Research Council (NHMRC) recommend that abnormal gait; problems with feet, hips and knees; difficulties with balance and coordination; and hip and knee joint pain should be appropriately managed [14,15]. Physiotherapists have a unique skillset to ensure these aspects are addressed through tailored physical activity programs appropriate to the individual child’s needs (e.g. play-based and family-centred), whilst ensuring injury prevention [20,23].

Despite physiotherapists being identified as health professionals with appropriate skills and knowledge to suitably care for children with obesity, a cross-sectional survey of Australian physiotherapy practice trends and professional needs, found that only half of physiotherapists, who provide care for children, provided services specifically to children with overweight or obesity, with ‘lack of service prioritisation and resources to support their non-acute care’ as the main reasons for not providing services [24]. The findings from this same study demonstrated that just under half of physiotherapists were assessing the motor skills of children who were overweight or obese and this was attributed to not having enough time [24] which may have led to unidentified needs for intervention or not having a physiotherapy-specific evidence-based guideline to support their intervention choices. This suggests that despite their appropriate training and skill set, physiotherapists are currently providing little input into the management of children who are overweight or obese for a variety of reasons. Instead, there is a common trend for other professionals including physical education specialists, exercise physiologists and school nurses, to be implementing interventions that are within the scope of physiotherapists [25] even though physiotherapists have a unique skill set in assessing the underlying reasons for lack of physical activity in children [21].

There are gaps in the literature regarding appropriate and universal physiotherapy protocols for management of childhood obesity. Previous research regarding physiotherapy intervention for managing children with obesity has concluded with recommendations to develop evidence-based guidelines to assist physiotherapists in the development of effective interventions [24]. However, to our knowledge, no prior reviews have examined available literature in the context of physiotherapy practice. Hence, this scoping review aims to (i) explore and critically appraise current evidence regarding physiotherapy and related interventions to manage childhood obesity and; (ii) broadly synthesise the findings of articles regarding interventions to guide physiotherapists in evidence-based management of childhood obesity.

## Methods

This scoping review is reported using the PRISMA Extension for Scoping Reviews (S1 Table) [26]. A review protocol exists on Open Science Framework and can be viewed at https://osf.io/fap8g/.

### Search strategy

The search was initially undertaken on 26^th^ August 2019 and repeated on 23^rd^ May 2020. A combination of search terms was used (S2 Table). Searching of literature occurred in the following five databases: PubMed, Embase, CINAHL, and Medline via OVID. Grey literature was identified with Google searches ending in “file:pdf” and expert referral.

### Study selection

After duplicate articles were removed, two authors SP and KT independently conducted title and abstract screening and identified potentially relevant articles for full-text review. A process of consensus for included articles at full text occurred between the two authors during face-to-face meetings. Outstanding disagreements between the two authors were resolved by a third researcher (NM), in collaboration with SP and KT to determine the list of possibly relevant articles to examine at full text.

Studies that appeared to meet the inclusion criteria based on title and abstract screening were retrieved in full text and inclusion/exclusion criteria were applied at full text level. A final consensus between the two authors (SP and KT) was achieved on included articles through face-to-face discussions. Discrepancies were resolved by a third researcher (NM) to achieve a final consensus on included full text articles.

### Inclusion/Exclusion criteria

Articles were screened using the below criteria (Table 1).

**Table 1:** Inclusion and exclusion criteria.

Inclusion	Exclusion
Infants <2, children 2–12 years, adolescents 13–18 years who are overweight/obese. Management/intervention used in article may be physical, behavioural, nutritional or educational in nature (or a combination of all). Intervention used in article must be delivered by a physiotherapist OR able to be delivered by a physiotherapist according to the scope of physiotherapy (e.g., school-based physical education programs, physical activity programs prescribed by exercise physiologists). Article published in English language only. Interventional studies, reviews, protocols, policies, procedures, guidelines, recommendations, position statements or perspectives reporting on obesity management/intervention.	Non-human studies Pregnancy-related obesity literature (e.g., intervention focused on mother during pregnancy). Studies where no interventions are provided (e.g., profiling studies, exploring trajectories or correlation/cross-sectional studies, protocol only). No accompanying full texts made available (e.g., abstract or poster only, unable to source).

### Critical appraisal tools

Critical appraisal of each included publication was undertaken by two reviewers (SP and KT) independently. A Kappa statistic was calculated to determine the level of agreement in critical appraisal scoring between the two reviewers. After independently scoring, a process of consensus occurred between the two authors (SP and KT) during face-to-face meetings.

Three appraisal tools were used to assess the quality of included studies/articles, selected according to the relevant study designs. The Mixed Methods Appraisal Tool (MMAT) was used to assess the quality of quantitative and qualitative studies [27]. It has been demonstrated as a tool used to appraise the quality of empirical studies including primary research, based on experiments or observations [28,29]. It appraises quality of five categories: qualitative research, randomised control trials, non-randomised studies, quantitative descriptive studies and mixed-methods studies [27]. The ratings better inform the methodological quality of the included studies to assist with decision making in data synthesis. Criteria were assessed using the following: ‘yes’ scoring as ‘1’ and ‘no’ or ‘can’t tell’ scoring as ‘0’, with possible total scores ranging from 0–7. Only studies that achieved a score of five or more out of seven (Critical Appraisal Score (CAS) of ≥71%) on the MMAT were considered to have strong methodological quality and were included in the descriptive synthesis (see ‘Data extraction and synthesis’). This method is consistent with a previous utilisation of the MMAT in a high-quality systematic review [30].

The iCAHE Guideline Quality Checklist was used to assess the quality of guidelines and included grey literature [31]. This tool measures methodological quality of guidelines across six domains: availability, dates, underlying evidence, guideline developers, guideline purpose and users, ease of use. Criteria was assessed using dichotomous responses with ‘yes’ scoring as ‘1’ and ‘no’ scoring as ‘0’, with possible total scores ranging from 0–14.

The Joanna Briggs Institute Checklist for Systematic Reviews and Research Syntheses was used to assess the quality of systematic reviews [32]. This tool allows assessment of the methodological quality and the extent to which a study has addressed the possibility of bias in its design, conduct and analysis. Criteria was assessed using the following response options: ‘yes’ scoring as ‘1’ and ‘no’ or ‘unclear’ scoring as ‘0’, with possible total scores ranging from 0–11.

To standardise scoring across multiple critical appraisal tools, after each article was critically appraised with the appropriate tool, a critical appraisal score (CAS) was calculated as a percentage of ‘yes’ responses over the total possible score, revealing a consistent percentage CAS for all included articles. Articles achieving a CAS of ≥71% were considered to have high methodological quality.

### Data extraction and synthesis

Data extraction was completed by two authors SP and KT independently using a standardised data extraction form to collect relevant information. This included: first author, year of publication, country of origin, aims/purpose, study population and sample size, methodology (including study design and statistical models used), intervention type, comparator, duration of intervention, outcomes, key findings related to the aims of this scoping review. Discrepancies in data extraction were resolved through a process of consensus using face-to-face meetings after independent data extraction was completed.

All included studies were narratively reported according to study type including guidelines, reviews and clinical trials by authors KT and SP. Clinical trials were explored under presenting key themes highlighting the most predominant form of interventions. The intervention themes were then described within the International Classification of Functioning, Disability and Health—Child and Youth Version (ICF-CY) model [33]. As a classification system recognised by the World Health Organisation (WHO), the ICF-CY uses a universal language for health professionals and acknowledges health and disability from a multidimensional perspective [33]. Studies mapped to the ICF-CY domains were clinical trials of high quality, with a score of five or more out of seven on the MMAT [30]. Clinical trial interventions were categorised into the taxonomy within the existing ICF-CY model. This included “Body Function and Structure Impairments”, “Activity Limitations”, “Participation Restrictions”, “Environmental Factors”, “Personal Factors” [33]. Multicomponent interventions were replicated amongst applicable categories to demonstrate the overall distribution of intervention modes within the ICF-CY model. The same procedure was repeated to demonstrate the distribution of outcome measures utilised in the included studies.

A descriptive synthesis was conducted to explore major trends regarding intervention types for children with overweight/obesity, considered to be within the scope of physiotherapy practice. Clinical trials were reviewed to identify the most predominant form of intervention and classified in a descriptive synthesis accordingly under “quantitative focused physical activity”, “qualitative focused physical activity” and “multicomponent interventions”. “Quantitative focused physical activity” is defined as the quantity of any bodily movement produced by skeletal muscles, focused on maximal energy expenditure [34]. “Qualitative focused physical activity” is defined as improving quality of movement using planned and structured bodily movement, focused on the development of motor skills [34]. “Multicomponent interventions” have a physical activity intervention with more than one other intervention implemented including diet/nutritional education, healthy lifestyle education and/or addressing environmental factors [11–13]. The outcome measures were reported according the ICF-CY model including “body structure and function”, “activity limitation”, “personal factors” and “environmental factors”. Outcomes mapped to “body structure and function” were further divided into two categories: health-related physical fitness and performance-related physical fitness measures. For example, health-related physical fitness measures are related to measures of good health including: (i) anthropometry, (ii) cardiovascular/cardiorespiratory fitness, and (iii) blood serum analyses [34]. Performance-related fitness measures are related to measures of enhanced motor proficiency including: (i) coordination, (ii) balance, (iii) speed, (iv) agility, (v) strength, (vi) flexibility, (vii) power, and (viii) posture and gait [34,35]. Outcome measures mapped to “activity limitation” were associated with measuring difficulties an individual may have in the execution of a task or action including physical activity-, sedentary behaviour- and sleep-related outcomes [33]. Outcomes mapped to “personal factors” were behavioural measures [33]. An example of this included readiness for change assessment. Outcomes mapped to “environmental factors” were associated with measuring the physical, social and attitudinal environment in which the individual lives and conducts their daily living including parental engagement and parental satisfaction outcomes [33].

Two stages of coding were undertaken during the descriptive synthesis. Firstly, individual investigations were coded based on whether the effect was positive/desirable (+), negative/undesirable (-) or no effect (0) (i.e., a decrease in waist circumference for children with overweight or obesity is a desirable direction of change, whereas an increase in waist circumference is an undesirable direction of change, and an insignificant change in waist circumference is deemed to have no effect). The second stage of coding was then applied whereby, if at least two thirds (66.6%) of the investigations were reported to have significant results in the same direction of change, then the overall result was considered consistent and the appropriate code was applied [36]. Specifically, if 66.6% or more of the studies included in the descriptive synthesis for the specific intervention mode demonstrated a significant positive or negative change, or no significant change, then summary codes of desirable (D), undesirable (U) or no effect (0) were given respectively. If more than one investigation and less than five investigations were undertaken assessing the impact of an intervention on outcomes for children with obesity or the above criteria was not met, then it was coded as questionable (?).

The total number of studies (n_s_) and the total number of participants (n_p_) included to determine the summary code which described the impact of the interventions on outcomes for children with obesity, were calculated.

### Sensitivity analysis

A post-hoc sensitivity analysis was conducted to determine if the findings of this scoping review would change when we explored the results with all overweight/obesity ‘prevention’ studies removed from the analysis (i.e., only leaving overweight/obesity ‘management’ interventions). Management of childhood obesity interventions is specifically inclusive of: (i) a population that is overweight or obese as the intervention group; and (ii) aims to improve measures of health, performance- and behaviour-related outcomes of the targeted population. All included studies not fulfilling these criteria were considered to be preventative studies and were excluded in the sensitivity analysis. The descriptive synthesis method for analysis of data was repeated with the included studies for outcome measures with five or more investigations, as per the original descriptive analysis. When less than five investigations were undertaken, exploring the impact of intervention on a given outcome, they were not included in the sensitivity analysis, as it does not fulfil the criteria for descriptive analysis outlined above.

## Results

### Study selection

**Fig 1:**
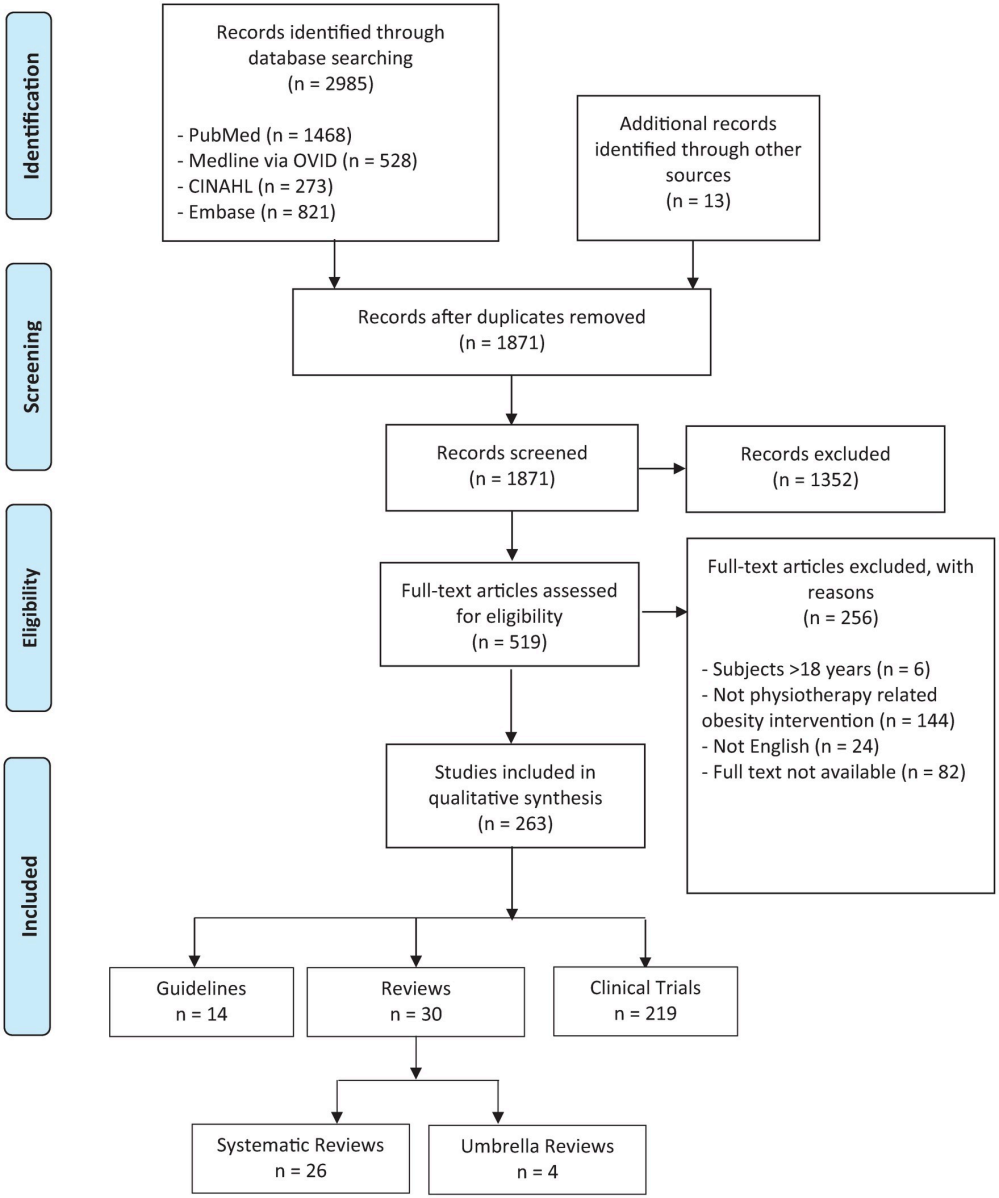
PRISMA flow diagram. Processes for study screening, eligibility and inclusion [26].

The literature search yielded 1871 titles and abstracts for review. From these, 519 potentially relevant articles were selected for full text review. Two hundred and sixty-three of the full text articles were identified as meeting the inclusion criteria for the present scoping review. This included 14 guidelines, 30 reviews (26 systematic and 4 umbrella) and 219 clinical trials (Fig 1).

### Methodological quality assessment

The percentage agreement between reviewers was moderate with 75.50%. Cohen’s Kappa analysis confirmed moderate agreement between the two raters, k = 0.44 (p < 0.001). One hundred percent agreement was achieved on all papers during the consensus process and this was the final CAS reported. One hundred and fifty-eight articles achieved a CAS of ≥71% and were considered high quality. One hundred and five articles were graded <71%. The mean CAS (percentage score) for methodological quality of the included articles was 68.07%.

### Guidelines

**Fig 2:**
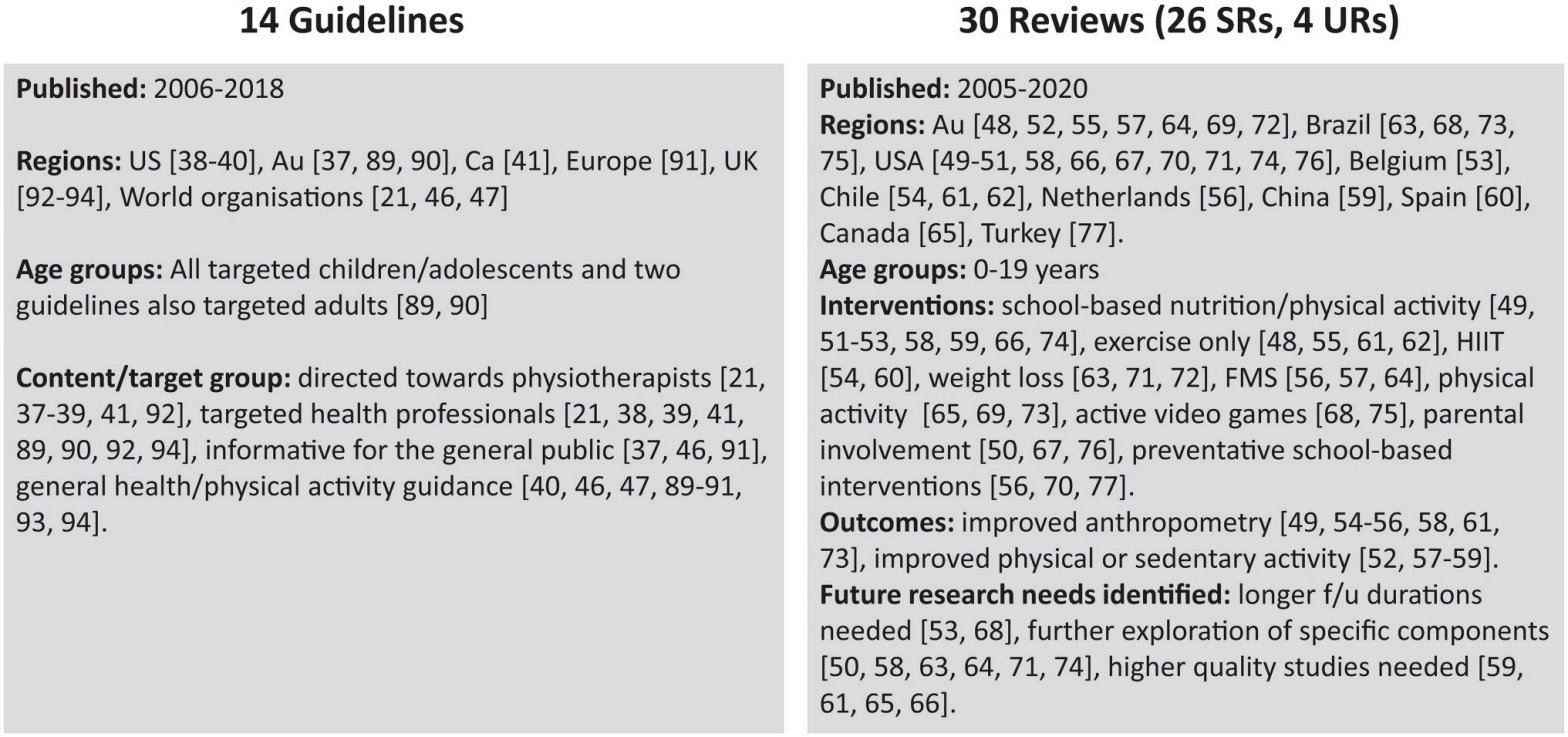
Summary of secondary research. Citations are included in square brackets [21,37–41,46–77,78–83]. Refer to S3 and S4 Tables for detailed information for Guidelines and Reviews respectively. Abbreviations: Au—Australia, Ca—Canada, SRs—systematic reviews, UK—United Kingdom, URs—Umbrella reviews, US—United States.

Fourteen of the 263 articles were guidelines for the management of children with obesity [14,15,21,37–47] as summarised in Fig 2 and S3 Table. The content included in the guidelines covered clinical recommendations and health promotion strategies including the importance of physical activity management.

### Reviews

Thirty of the 263 articles were reviews [48–77] as summarised in Fig 2 and S4 Table. Twenty-six were systematic reviews and four were umbrella reviews. Of the 26 systematic reviews included, a total of 556 studies were captured. Thirty-one (14.16%) of the 219 clinical trials included in this scoping review were found to overlap with the 556 studies identified in these systematic reviews. Nine (4.11%) of the 219 clinical trials were included in two systematic reviews [84–92]. Four (1.83%) of the 219 clinical trials were included in three or more systematic reviews [87,88,93,94]. The four umbrella reviews (systematic reviews of systematic reviews) included in this scoping review, captured a total of 86 systematic reviews. Nine (34.62%) of the 26 systematic reviews included in this scoping review were found to overlap with 86 systematic reviews identified in the umbrella reviews. No systematic reviews were duplicated amongst the four umbrella reviews.

### Clinical trials

Two hundred and nineteen of the 263 included articles were clinical trials [84–94,95–302] as summarised in Table 2 and S5 Table. Clinical trial articles were published between 1970 to 2020 and included from three to 17066 participants aged two to 19 years. The sum of all participants from the 219 included articles was 112,524. Clinical trials included childhood obesity prevention (50%) and management (50%) interventions. Intervention studies included non-randomised (56%) and randomised trials (44%) clinical trials. Clinical trials included in the present scoping review were focused on increasing physical activity (n = 36) [114,119,133,136,146,148,149,153,168,172,188–191,205,207,208,210,214,239,240,242,244,246–248,256,263,271,272,274,276,281,283,286,294]; increasing physical activity and improving diet or nutritional education (n = 19) [93,98,127,129,150,162,164,218,222,229,251,254,258,260,267–269,277,288]; increasing physical activity and offering healthy lifestyle education (n = 13) [84,90,120,160,161,182,204,219,238,241,253,279,293]; enhancing posture and gait (n = 3) [173,213,270]; improving targeted motor skills (n = 7) [107,135,142,209,216,235,249]; increasing aerobic exercise (n = 26) [88,97,101,104,125,140,144,165,166,169,181,183,184,193,197,199,223,224,228,230,236,257,265,285,292,300]; increasing strength exercise (n = 4) [203,250,261,275,292]; increasing strength and aerobic exercise combined (n = 13) [109,123,138,143,158,175,196,202,215,262,264,290,298]; reducing sedentary behaviour (n = 1) [167]; technology-based interventions, including exergaming and mHealth (n = 9) [85,132,145,154,157,195,212,226,234]; multicomponent and multidisciplinary (n = 74) [89,92,94–96,99,100,102,103,105,108,111–113,115–118,121,122,124,128,134,137,139,141,147,151,152,155,156,159,163,170,171,176–180,185–187,192,198,200,201,206,211,216,217,220,221,225,227,231–233,237,243,245,252,255,266,269,273,278,280,282,284,287,289,291,296]; family-based and/or environmental interventions (n = 88) [86,90–92,95,96,98–100,105,108,110–112,114–119,123,127–131,139,141,142,145,149,150,155,156,160–162,170,172,174,176,179,180,182,187–190,192,195,207,211,216,217,219,221,225,227,231–233,237,241,243,245,247,248,252–255,258–262,266,270,278,282,283,289,291,296,299,301,302]. Studies that were applicable to multiple themes were replicated within appropriate categories and account for the overlap in the total number of clinical trials. Table 2 provides a summary of the interventions and outcome measures utilised in the clinical trials.

**Table 2:** Summary of interventions and outcome measures utilised in the 219 included clinical trials.

No. of trials & focus	Intervention	Outcome measures
36 PA	No./duration of PE classes [146,153,188–191,210,239,244,246,247,256,263,274,276,283], sport-related [119,136,146,148,153,172,207,248,256,281], classroom PA [114,189,190,205,210,240,244,246,256], recess/lunch organised PA [149,256], walking/jogging [114,148,149,168,208,242,286], exercise training [214,239,248,256,271,272,294], adventure education [119], active commuting to/from school [283], family-based and/or environmental interventions [114,119,149,172,188–190,207,247,248,283].	Anthropometric [114,119,133,136,146,148,153,168,172,188,189,191,205,207,208,210,239,242,244,247,248,263,271,272,274,276,281,294], CV/CR [133,136,168,189,205,207,210,239,242,246,248,263,271,272,274,276,281,286], blood serum analyses [133,189,207,239,248,271,274,281,294], physical performance [189,190,214,263,272], other [114,117,146,149,189,210,214,240,244,246,247,256,263,274,283].
19 PA + diet/nutrition education	Nutrition education [93,98,127,129,150,218,251,254,260,268], dietary counselling [258,277], dietary changes/restrictions [98,150,222,229,267,277,288], cooking classes [127,162,218], provision of snacks/meals [127,162,164,229,260], family-based and/or environmental interventions [98,127,129,150,218,254,258].	Anthropometric [93,98,127,129,150,162,164,218,222,229,251,254,260,267,268,277,288], CV/CR [93,127,150,162,164,222,229,251,260,267,288], blood serum analyses [93,129,222,251,258,260,277], physical performance [129,218,229,258], other [98,129,251,254,260,268].
9 Technology based	Exergaming [132,154,157,212,234], mobile-phone apps [145,195,226], website-based education [85]. Some studies included nutrition and/or healthy lifestyle education [85,145,195,226], family-based interventions [85,145,195].	Anthropometric [85,132,145,195,226], other [85,132,145,154,157,195,212,226,234].
1 Sedentary behaviour	“Switch Off-Get Active”, health education intervention aimed to increase physical activity at the expense of screen time [167].	Anthropometric, CV/CR, PA/sed, PA self-efficacy [167].
13 PA & health lifestyle education	Lifestyle education (+CBT) e.g., PA/screen time [84,90,120,160,161,182,204,219,238,241,253,279,293]; and assistance with academic areas [293]. Family-based and/or environmental interventions [90,160,182,219,241,253].	Anthropometric [84,90,120,204,219,238,241,253,279,293], CR/CV [120,204,238,293], other [84,160,161,182,204,241,253].
3 Posture & gait	Movement quality/games [213], lower extremity neuromuscular & core exercises [173], stretching/flexibility/strengthening/agility/aerobic endurance with “locomotion-emphasis” [270]. Diet/nutrition education with parental involvement + posture/gait intervention [270].	Anthropometric [270], physical performance [173], other outcomes [173,213,270].
88 Family Based and/or environmental	Individual/group counselling with a multidisciplinary team member (physiotherapist, dietitian, psychologist, paediatrician) [95,96,111,123,139,141,145,174,225,227,232,253,254,258,270,299]; nutrition and/or healthy lifestyle education [92,96,99,100,108,111,112,114,116–118,123,127,128,130,131,139,142,145,150,156,162,170,172,176,179,180,182,192,195,211,219,225,227,231–233,237,241,243,245,252–255,258,260,266,270,278,282,291,296,299,302]; parents supporting behavioural changes [99,115,145,170,187,192,216,225,231,241,243,248,255,262,278,282,301,302]; parents involved in meetings/support groups [108,291], exercise programs [129,139,160,176,227,258,299,301,302]; sports [100,237,255]; active commuting [283]; motor development [142]; online resources [145,156,172,195,296]; and or family events/activities [100,105,127,128,221,253]. Environmental interventions include grants/funding [105,108,127,245,301]; professional development e.g. upskilling physical educators [86,90,96,98,100,105,108,112,114,115,117,127,161,172,176,188,189,207,221,248,261,296]; availability of healthy/unhealthy food [91,99,108,110,119,127,128,155,247,259,289,291]; changes to physical infrastructure [91,99,108,117,139,149,190,217,232,233,247,255,262,296], changes to school policy [91,96,108,117,127,172,178,182,232,233,259,289,291,296]; provision of social support [115,116,225]; and community physical activity [172].	Parental engagement and/or satisfaction [108,210,231,266,268].
26 Aerobic exercise	High/low intensity interval training [88,97,144,193,224,236,257,300]; walking/jogging [165,169,183,197,228,230,295,297], treadmill or cycle ergometer [101,181,184]; equbic air board [265]; plyometrics [223,236]; group activities [125], exercise programs [140,166,199,285,292], aerobic exercise + calorie restrictions [101,199], aerobic exercise + nutrition and/or healthy lifestyle education [125,166,181,199,230,257,285,292].	Anthropometric [88,97,101,104,125,140,144,165,166,169,181,183,193,197,199,223,224,230,236,257,265,295,297], CV/CR [88,97,104,144,166,169,181,184,193,197,199,224,230,236,257,265,285,292,295], blood serum analyses [101,166,181,183,197,199,230,236,292,295,297], physical performance [140,144,165,181,193,223,224,228,285], other [140,165,166,230].
4 Strength exercise	Exercise programs [261,275,292], exercises using different equipment (e.g. sleeping mats, tennis balls, volleyballs, basketballs, swiss balls, pool floats, elastic bands) [203,250], strength exercises + stretching program [250], strength exercises + motivational interviewing [275]. Some studies also included nutritional and/or healthy lifestyle education [203,261,292].	Anthropometric [203,275], CV/CR [203,292], blood serum analyses [203,292], physical performance [203,250], other [250,261,275,292].
13 Strength & aerobic	Resistance band/weight machines [109,143,262], treadmill/elliptical/cycling [109,123,138,143,175,290,298], exercise program/circuit training [123,138,175,196,202,215,262], walking/running [202,215,298], sport [158,262,290,298]. Some studies also included dietary restriction [123,138,143,158,175,196], nutrition/healthy lifestyle education [123,138,158,262], psychological group sessions [123,138,175], family-based and/or environmental interventions [123,196,262].	Anthropometric [109,123,138,143,175,196,202,215,262,264,290], CV/CR [143,202,264,290], blood serum analyses [109,123,175,196,202,215,264], physical performance [143,202,262], other outcomes [158,202,262].
7 Motor skills	Motor development [107,142], GM/FM equipment [107], exercise programs [209,216,235,249], tennis-specific training [135], motor skill development with nutrition education/counselling [209,216].	Anthropometric [142,209,216,249], CV/CR [135,209,235,249], physical performance [107,209,216,249], other [107,216].
41 Multicomponent/multidisciplinary	Physiotherapy [102,103,206,225,273,280,284], dietitian/nutritionist [95,108,111,124,139,141,152,170,185,198,200,201,206,216,220,232,243,252,269,280,284], psychology [95,111,122,139,141,152,159,163,171,200,201,243,252,280,291], nurse [115,206,291], paediatrician/physician [95,139,220], increasing PE class intensity/no./duration [89,95,96,99,113,118,137,139,163,176–178,206,217,252,287,289,296], exercise program [94,111,112,115–117,122,124,134,141,147,151,152,155,156,159,170,171,185,187,192,198,200,201,216,220,232,233,243,245,255,266,269,278,282,291], walking/running [179,220,252,269], classroom PA [92,128,217], organised recess/lunch PA [89,96], sport [100,102,103,108,111,112,118,134,147,159,178,186,201,220,221,227,232,237,252,266,280,291], recreational activities [89,100,105,128,180,211,227,231], active commuting [147,206], nutrition and/or healthy lifestyle education [89,92,94–96,99,100,102,103,105,108,111–113,115–118,121,122,124,128,134,137,139,141,147,151,152,155,156,163,170,171,176,177,179,180,185–187,192,198,201,206,211,216,217,220,221,225,227,231–233,237,243,245,252,255,269,273,278,280,282,284,287,291,296], cooking classes [96,139,186,245,266], provision of snack/meals [94,103,115,155,156,186,237,266], dietary restriction [94,124,141,147,159,163,199,200,220,269,287], and/or behavioural modification [102,103,111,113,115,116,118,122,139,155,170,187,216,221,225,227,231,243,266,282,287,289,291], family-based and/or environmental interventions [92,95,96,99,100,105,108,111,112,115–118,124,128,139,141,155,156,170,176,178–180,187,192,198,211,216,217,220,221,225,231–233,237,243,245,252,255,266,278,282,289,291,296].	Anthropometric [89,92,94–96,99,100,102,103,105,111,115–118,122,124,128,134,137,139,141,147,151,152,155,156,159,163,170,171,178–180,185–187,192,198,200,201,206,211,216,217,220,227,231–233,237,243,245,252,255,266,269,273,278,280,282,284,287,291,296], CV/CR [92,94,95,99,105,116,122,124,134,137,139,152,171,180,186,200,211,217,220,225,227,231,233,243,273,287,289], blood serum analyses [89,92,94,113,147,152,155,163,180,186,198,206,211,284,287], physical performance [94,103,151,171,178,216,233,269,296], other [89,92,94,95,99,100,108,113,121,122,128,139,156,176,177,185,186,201,211,216,217,221,225,227,231–233,237,255,266,278,280,291].

*Note*. See S5 Table for detailed information. Examples of ‘other’ outcomes include: Physical activity/sedentary behaviours, diet/dietary behaviours, knowledge/attitudes/self-efficacy/self-competence/enjoyment relating to various aspects of lifestyle behaviours, quality of life, general health, perceived ratings of exertion, self-esteem, depression, body image/size, menstrual regularity, alignment during gait/posture loading conditions, foot pressure values and/or pain, trunk alignment, metabolic rate, perceived fitness, sleep, cognitive abilities. Abbreviations: CBT—cognitive behaviour therapy, CV/CR—cardiovascular/cardiorespiratory, FM—fine motor, GM—gross motor, PA- physical activity, PA/sed—physical activity and/or sedentary behaviours, PE—physical education, no.–number.

### Interventions mapped to the International Classification of Functioning, Disability and Health—Child and Youth Version

**Fig 3:**
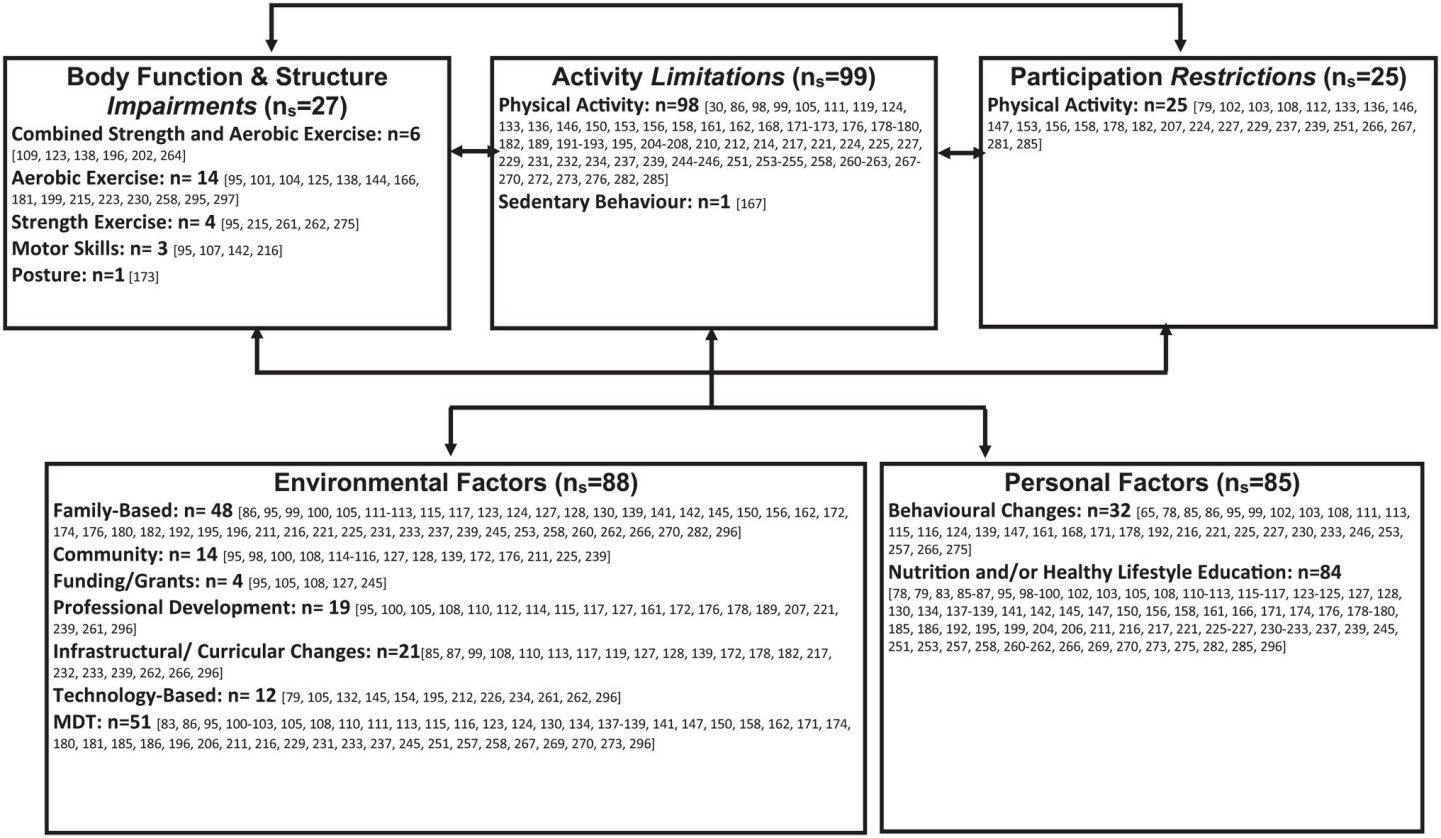
ICF-CY model with mapped interventions.

**Fig 4:**
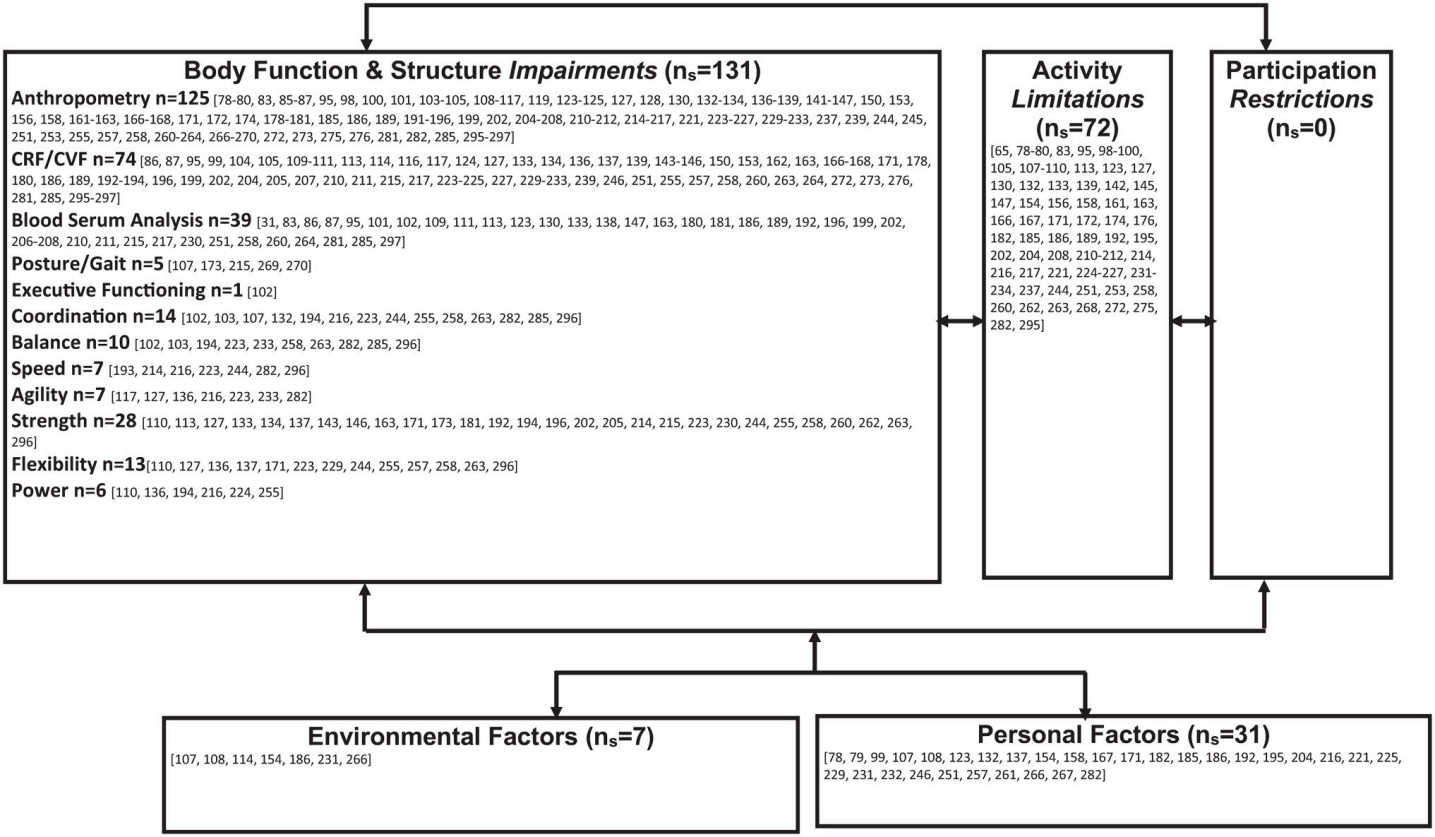
ICF-CY model with mapped outcome measures.

From the 219 clinical trials included in this scoping review, 138 clinical trials were considered high-quality studies and were consequently mapped on the ICF-CY. “Activity Limitation” interventions were represented by the greatest number of studies (n_s_ = 100) and “Participation Restriction” interventions represented the least number of studies (n_s_ = 26) (Fig 3). Outcome measures in the “Body Function and Structure Impairment” domain of the ICF-CY were represented by the greatest number of studies (n_s_ = 134) and “Participation Restriction” outcome measures were least represented (n_s_ = 0) (Fig 4).

### Descriptive synthesis of results

Four of the eight quantitative focused physical activity intervention types resulted in desirable changes in outcome measures; neither of the two qualitative focused physical activity intervention types resulted in desirable changes in outcome measures; and four of the nine multicomponent intervention types resulted in desirable changes based on outcome measures utilised (Table 3; S6 Table).

**Table 3:** Descriptive synthesis of results for studies investigating the effects of child overweight and obesity preventative and treatment strategies.

	Anthropometric	CRF, CVF	Blood serum analysis	Coordination	Balance	Speed	Agility	Strength	Flexibility	Power	Posture+ gait	Physical activity assessment	Sedentary behaviour assessment	Sleep quality/duration	Self-esteem, -efficacy and -perception assessment	Food/drink behaviour and consumption
**Quantitative focused physical activity**																
Walking/running	D	?	D	?	?			?			?	?				?
Combined aerobic + resistance exercise	D	?	D					?								?
Physical education + increased physical activity/exercise	D	D	0	?		?			?			?	?		?	
Sports-based	?	?	D							?						
Exergaming	?											?	?			
Physical activity in classroom (activity breaks)	?	?		?												
Plyometrics	?	?		?	?		?	?	?							
Intensity training	D	?					?			?		?				
**Qualitative focused physical activity**																
Motor skills				?							?					
Alignment during gait								?			?					
	Anthropometric	CRF, CVF	Blood serum analysis	Coordination	Balance	Speed	Agility	Strength	Flexibility	Power	Posture+ gait	Physical activity assessment	Sedentary behaviour assessment	Sleep quality/duration	Self-esteem, -efficacy and -perception assessment	Food/drink behaviour and consumption
**Multicomponent intervention**																
Physical activity + healthy lifestyle education	?	?		?	?							?	?		?	
Healthy lifestyle education + environment	?											?	?		?	
Physical activity + diet/nutrition education	?	D	D					?	?			?			?	
Physical activity + environment	0	?	0									?				
Diet/nutrition education + healthy lifestyle education + environment	?	?	?									?	?			?
Physical activity + healthy lifestyle education + diet/nutrition education	?	?	?	?	?			D	?			?	?	?	?	?
Physical activity + healthy lifestyle education + environment	?											?	?		?	?
Physical activity + diet/nutrition education + environment	?	?	?	?	?	?	?	D	?	?	?	?	?			?
Physical activity + healthy lifestyle education + diet/nutrition education + environment	?	?	?		?	?	?	D	?	?		D	D	?	D	?

Key: D Desirable, 0 No effect, U Undesirable, ? Questionable, No data.

Intervention types with desirable changes in anthropometric outcomes included quantitative focused physical activity such as walking/running (n_s_ = 5, n_p_ = 1525), combined aerobic and resistance exercise (n_s_ = 4, n_p_ = 345), physical education and increased physical activity/exercise (n_s_ = 12, n_p_ = 3842), and intensity training (n_s_ = 2, n_p_ = 235). Interventions demonstrating no change based on anthropometric outcomes included multicomponent interventions such as physical activity with environmental changes (n_s_ = 6, n_p_ = 8466).

Interventions with desirable changes in cardiorespiratory/cardiovascular (CRF/CVF) outcomes included quantitative focused physical activity such as physical education and increased physical activity/exercise (n_s_ = 9, n_p_ = 1602); and multicomponent interventions such as physical activity with diet/nutrition education (n_s_ = 6, n_p_ = 372).

Interventions demonstrating desirable changes in blood serum results included quantitative focused physical activity such as walking/running (n_s_ = 2, n_p_ = 90), combined aerobic and resistance exercise (n_s_ = 4, n_p_ = 345), sports-based activities (n_s_ = 1, n_p_ = 30); and multicomponent interventions including physical activity with diet/nutrition education (n_s_ = 4, n_p_ = 181). Interventions demonstrating nil change on blood serum analysis included quantitative focused physical activity such as physical education and increased physical activity/exercise (n_s_ = 1, n_p_ = 137); and multicomponent interventions such as physical activity with environmental changes (n_s_ = 3, n_p_ = 1578).

Interventions resulting in desirable changes on strength included multicomponent interventions such as physical activity with health lifestyle education and diet/nutrition education (n_s_ = 5, n_p_ = 336), physical activity with diet/nutrition education and environmental changes (n_s_ = 5, n_p_ = 3032), and physical activity with healthy lifestyle education and diet/nutrition education and environmental changes (n_s_ = 3 n_p_ = 1442).

Multicomponent interventions such as physical activity with healthy lifestyle education, plus diet/nutrition education and environmental changes showed desirable changes with improving quantified physical activity (n_s_ = 21, n_p_ = 28565), reducing sedentary behaviours (n_s_ = 10, n_p_ = 6209) and improving self-esteem, -efficacy and -perception (n_s_ = 6, n_p_ = 2037).

### Sensitivity analysis

A sensitivity analysis was undertaken to determine if studies which were focused on prevention (rather than management) of obesity were influencing the results. Once prevention studies were removed, leaving only overweight and obesity management studies, seven intervention modes showed varied results to the initial descriptive synthesis (S7 Table).

Specifically, interventions focused on increasing physical education and increasing physical activity/exercise, and intensity training changed from a desirable to questionable impact on anthropometric outcomes. Further, interventions focused on physical activity with diet/nutrition education changed from questionable to desirable impact on anthropometric outcomes when prevention studies were removed, and interventions focused on enhancing physical activity with environmental modification varied from no effect to a questionable impact. Interventions focused on physical activity with diet/nutrition education and environmental modification varied from questionable effect to no effect.

When obesity prevention studies were removed from the analysis (leaving only overweight and obesity treatment studies), interventions focused on combining physical activity with healthy lifestyle education and diet/nutrition education were shown to have desirable outcomes on CRF/CVF, which was initially questionable.

Interventions focused on increasing physical education and increased physical activity/exercise; and interventions focused on enhancing physical activity with environmental modification changed from no effect to questionable impact on blood serum analyses. Further, interventions focused on physical activity with diet/nutrition education and environmental changes varied from questionable to desirable changes on blood serum analyses.

Interventions focused on physical activity with diet/nutrition, education and environmental modification; and interventions focused on physical activity with healthy lifestyle education, diet/nutrition education and environmental modification varied from a desirable to questionable impact on strength outcomes.

Interventions focused on physical activity with healthy lifestyle education, diet/nutrition education and environmental modification varied from a desirable to questionable impact on sedentary behaviour outcomes.

Interventions focused on physical activity with healthy lifestyle education, diet/nutrition education and environmental modification varied from a desirable to questionable impact on self-esteem, self-efficacy, and self-perception outcomes.

## Discussion

The objective of this scoping review was to explore and critically appraise current evidence regarding physiotherapy and related interventions to manage childhood obesity and to broadly synthesise the findings of articles regarding interventions to guide physiotherapists with evidence-based management of childhood obesity. The key finding from our review suggests that quantitative physical activity facilitates desirable changes in health-related outcomes whereas multicomponent interventions facilitate desirable changes in behaviour measures.

From the 263 articles including guidelines, systematic reviews and clinical trials, the interventions most commonly applied were multicomponent interventions targeting physical activity, diet or nutrition education and lifestyle factors, commonly including environmental modification with parental involvement. The considerable interest in conducting multicomponent interventions may be linked to guidelines by WHO’s global strategy on diet, physical activity and health [303]. Due to WHO’s recognition of the existing heavy and growing burden of non-communicable diseases like obesity, governments have been recommended to promote applied research, with intervention programs aimed at improving diet and/or physical inactivity [303]. This increase in awareness and targeted funding provided by governments may have contributed to the mass amount of research relating to multicomponent interventions and obesity.

Multicomponent interventions have demonstrated a lack of improvement in anthropometric measures but desirable changes in physical activity; sedentary behaviour; self-esteem, self-efficacy and self-perception measures. To guide the determination of effectiveness or “success” of this intervention, the National Institute for Health and Clinical Excellence guidelines should be considered [44,45]. These guidelines suggest that childhood obesity interventions should not be “weight focused” but instead include behaviour change strategies to increase physical activity levels or decrease inactivity and improve eating behaviours [44,45]. These guidelines suggest that outcome measures focused on improvements in weight (or body composition) alone may not be the most appropriate way to measure the ‘success’ of an intervention. As demonstrated by this scoping review, multicomponent interventions target the factors and/or behaviours contributing to obesity and have demonstrated improvements in these areas, despite not achieving positive changes in anthropometric measures. This indicates that multicomponent interventions have achieved goals set by guidelines and therefore, are interventions that may be beneficial in the management of childhood obesity.

The most recent Cochrane reviews, inclusive of only long-term (6 to 24 month) randomised controlled trials, explored childhood obesity management and highlighted aspects of diet, physical activity and behavioural intervention components [11–13]. These Cochrane reviews have demonstrated that multidisciplinary or multicomponent interventions are effective for improving obesity-related outcome measures. In contrast to this scoping review, where multicomponent interventions resulted in a lack of improvement in anthropometric measures, the Cochrane reviews found low quality evidence that these interventions reduce BMI and moderate quality evidence of reduced weight in those who are overweight compared to controls [11–13] and that long-term studies led to desirable anthropometric changes [11–13]. Therefore, one may surmise that multicomponent intervention studies induce positive behavioural changes (as demonstrated in this scoping review) and if maintained over a period of 6 to 24 months may demonstrate positive improvements in anthropometric measures.

Most multi-component interventions included in the present scoping review incorporated environmental modification, as it is widely recognised that the physical environments and surrounding infrastructure influences the risk of childhood obesity [304]. However, the environment is complex as it is influenced by socio-economic status, affordability and accessibility to facilities, location of neighbourhood, attractiveness of the environment, geographical distance of home to central business district areas and city centres [305]. For example, pollution, the weight of a child’s school bag, footpath condition, lack of sheltered walkways, distance from home to school, and time constraints to extra-curricular activities before or after school, demonstrate the numerous barriers to the simple act of active commuting to school [305]. An environment where children can, under supervision, have the opportunity to engage in activities with some structure, focusing on skill development, whilst allowing children “to be children” and express themselves spontaneously is crucial [306]. This is not as simple as building new playgrounds, which was a common intervention noted in this scoping review. Interventions in this review inclusive of physical activity and environmental modification failed to demonstrate beneficial changes in outcome measures relevant to overweight or obesity. Purposeful consideration of social factors is likely needed to observe greater strides within the multi-component interventions. By holding greater awareness of environmental factors and its influence on physical activity levels in children, physiotherapists can achieve greater appreciation of such barriers, decrease stigma against obesity perpetuated through beliefs of a lack of perceived effort, to further individualise interventions for greater success [307].

An important part of a child’s environment includes the home environment, which commonly involves parental influences. Parents are considered ‘agents of change’ and have profound influence on various aspects of a child’s life [308]. For example, one of the most influential environments for development of eating behaviours and obesity in children is often determined by what is eaten at home [309]. Food exposure or availability, parental control, attitudes and behaviours around food will influence a child’s food intake and therefore, may influence the risk of obesity [310]. Furthermore, parental levels of physical activity are directly associated with the physical activity children receive and are exposed to [311]. Particularly in the earlier years of a child’s life, the family serves as the dominant source of social support which is linked to good physical, social, mental health and well-being [312]. Equally, poor family function due to poor communication, family conflict and parenting style (i.e., a non-authoritative, unengaged, permissive) have direct links to obesity [312]. The findings from the present scoping review highlight the breadth of multicomponent interventions that included parental components and similarly demonstrate favourable results where parental components were included. Family-based components are crucial in the management of childhood obesity [313].

Despite the benefits associated with family-based interventions, Edmunds conducted a qualitative exploration highlighting how various parental concerns may act as barriers for optimal childhood obesity management [314]. For example, fear that obesity management interventions may further perpetuate negative stereotypes around their child being overweight/obese within a society that values thinness and stigmatises adiposity [314]. Even acknowledging and “realizing that one is overweight is likely to be stressful and psychologically scarring” [315]. Parents may believe that such interventions may inadvertently increase communication to the child that their body size is undesirable and thus affect mental health negatively [315]. Such beliefs may mean that weight gain could go unnoticed or ignored and thus, reduce the likelihood of parents obtaining professional advice [314]. Contrary to these beliefs and concerns, our results demonstrate the opposite effect. Multicomponent interventions observed positive changes in self-esteem, self-efficacy and perception. Lowry et al. in a study investigating weigh management programs on self-esteem, reported that through parental involvement, increased social support, targeting self-esteem and related issues, improved locus of control through education and activities that were fun and engaging [316]. Additionally, Lowry et al. reported that self-esteem improvements were related to weight change, implying that the increase in self-esteem was due to the improvement of physical appearance [316]. This was contrary to findings from this scoping review which suggests that multicomponent interventions can improve self-esteem without requiring a change in anthropometric measures.

Multi-component interventions are further supported by previous NHMRC guidelines [14,15]. Part of the multidisciplinary team includes physiotherapists who have a large role and expertise both in physical activity and exercise but also in the understanding of the complex barriers and perceived barriers, that may affect an individual’s ability to make positive lifestyle changes [14,15]. Physiotherapy is constantly evolving and involves a holistic approach to the prevention, diagnosis and therapeutic management of movement difficulties or optimisation of function [317]. As demonstrated by this scoping review, quantitative focused physical activity demonstrates a large space available for exploration by physiotherapists in the treatment of childhood obesity. Physiotherapists play a role in the development, prescription, implementation, supervision and progression of appropriate physical activity, including cardiovascular training as well as resistance exercise, to increase muscle strength, flexibility, and endurance, and maintain weight loss under safe and controlled conditions [42]. Furthermore, physiotherapists have an understanding of physical function and movement and how to address any impairments in physical function that may be limiting movement and activity participation [81]. Participation in sport and being more active in school are major contributors to increased physical activity, energy expenditure and are recommended interventions [44,45]. Physiotherapists could play an integral role in working alongside physical education teachers, in the development of high-quality physical education programs in schools to facilitate maximal improvements in health and well-being of children with overweight or obesity [21].

Physical activity is widely acknowledged to be a critical factor in the effective management of childhood obesity [318]. As reflected by this scoping review, increases in the quantity of physical activity result in improvements in anthropometric, cardiorespiratory or cardiovascular fitness and blood serum analyses outcomes. These outcome measures are also risk factors for cardiovascular disease in later life [319–324]. Children who are overweight have an increased risk of pre-hypertension by 50% and double or triple the odds of hypertension, compared with children who are healthy weight [325]. Children with obesity have been linked to a 12-fold increase in fasting insulin concentration with abnormal levels of triglycerides, total cholesterol, LDL-c and HDL-c compared to children who are of healthy weight [326]. Therefore, by increasing the quantity or amount of physical activity, the improvements in anthropometric, cardiorespiratory or cardiovascular fitness and blood serum analyses outcomes may be associated with a decrease in weight and may lead to a decreased risk of cardiovascular disease.

Several research gaps were identified through this scoping review. For example, there was only one high quality clinical trial article which described motor skill development as an intervention. There is a lack of application of standardised motor skill outcome measures reported in clinical trials. The Bruininks-Oseretsky Test of Motor Proficiency, Second Edition (BOT-2) is an example of a standardised, internationally recognised comprehensive assessment of both gross and fine motor skills for children aged four to 21 years [327]. It objectively assesses manual control, manual coordination, body coordination, strength and agility [327]. Ideally the BOT-2 would support the strength and rigor that many of the observed papers lack for their motor skill outcome measures. However, limitations exist including the costs associated with purchasing the BOT-2 assessment tool and the time required for completion of the tests (often taking 45–60 minutes to administer) [327]. Furthermore, the learning effect of tasks within this outcome measure means that ideally children should not be retested within 6 months; which would therefore require a trial to be greater than 6 months in duration in order to appropriately understand the effects of an intervention on motor performance [327]. These limitations are possible explanations to the existing gap in the literature regarding motor skill interventions for children with overweight or obesity.

Faigenbaum and colleagues discuss a need for experienced paediatric professionals to be providing developmentally appropriate physical activity interventions that recognise the unique physical and psychosocial needs of children [328]. Physiotherapists are ideally suited to the provision of such programs, given that professional entry-level training incorporates paediatric-specific content. However, there is also a perceived need to expand paediatric content delivery in entry-level physiotherapy curricula [328,329], including child obesity-specific content [24]. Furthermore, physiotherapists have identified a need for guidance around obesity management/exercise prescription [24], highlighting the timeliness of the current scoping review. The current scoping review provides evidence of the value and scope of physiotherapy practice for this population, which may serve to advocate for services/resources to increase the engagement of physiotherapists in this important clinical area and to guide evidence-based practice. Central to physiotherapists, this scoping review has highlighted the importance of physiotherapists utilising health-related measures such as anthropometry to assess the clinical benefits of enhancing physical activity levels, whilst multicomponent interventions may first need to be implemented to induce positive behavioural changes that will support long term health improvements from physical activity related interventions that are fun, engaging and age appropriate. Furthermore, physiotherapists need to be mindful of the social and environmental barriers which may negatively impact their intervention outcomes (e.g., lack of resources or funding to access sports or perpetuating negative beliefs such as a lack of perceived effort from children or parents) when engaging clinically with children who are obese and their families. Emphasis should be focused on family-based components of intervention (i.e., engaging parents in therapy planning and implementation) as these are crucial to enhancing intervention outcomes for children with obesity.

### Strengths and limitations

Strengths of this review include the breadth of searches undertaken. Several major databases were searched using a broad inclusion criterion to fully understand the scope of existing literature up to this point. The study selection, critical analysis and extraction processes were performed in duplicate, reducing the risk of reviewer error or bias. Further, a sensitivity analysis was conducted which allowed for the exploration of obesity management with obesity ‘prevention’ studies removed from the synthesis.

Scoping reviews are intended to be broad, bringing together evidence from a range of study designs, and are particularly useful for a large, complex or heterogenous body of literature [330,331]. While a coding method was applied to create a descriptive synthesis of the results from included trials, we recognise that a large degree of heterogeneity existed between studies. Where an individual included study may have concluded an intervention to be successful overall, the current scoping review synthesised information based on individual study outcome measures which may not have reflected the overall concluding findings of the clinical trials.

Furthermore, due to the inclusion of umbrella reviews, systematic reviews, and clinical trials, it is important to recognise that overlap in primary studies may have occurred. Due to the number of primary studies included more than once in this scoping review, there may have been bias in the interpretation of results. To address this problem, an analysis of the results through a calculated percentage of overlap was created.

Despite the present limitations, the current scoping review provides an increased understanding of the available evidence that may be used as information to guide physiotherapy management of childhood obesity and has highlighted existing gaps in the literature.

### Future directions for research

Future reviewers should consider focused systematic reviews and meta-analyses to further the findings collected in this scoping review as part of ongoing improvement of standardised physiotherapy related management guidelines for childhood obesity. In particular, systematic reviews/meta-analyses of physiotherapy-specific interventions stratified by study designs (e.g., RCTs) may add further value. The large number of interventions with a questionable effect on health-, performance- and behaviour- related outcomes highlight areas for further exploration within the literature. Additionally, a multitude of personal and environmental factors may have influenced the observed results in this scoping review to varying degrees. Information from personal and environmental factors are confounding factors and if analysed, may guide direction for future researchers to further our understanding of factors contributing to successful interventions.

## Conclusion

Despite the plethora of childhood obesity interventions, findings from this review suggest that increases in the quantity of physical activity facilitates desirable changes in health-related outcomes (i.e., anthropometric measures, cardiorespiratory/cardiovascular measures, and blood serum analyses), whereas multicomponent interventions facilitate desirable changes in behavioural measures (i.e., physical activity and sedentary measures, and self-esteem, -efficacy and -perception measures). Such interventions are ideally suited to the scope and role of physiotherapists, particularly in relation to the prescription and progression of physical activity (either as an isolated intervention, or as part of multicomponent programs). Further research is needed to explore the effectiveness of motor skill interventions for obesity management in addition to the impact of environmental modification and parental involvement as possible moderating factors that may contribute to the success of physiotherapy interventions for children who are overweight or obese.

## Supporting information

S1 Table PRISMA checklist. (DOC)

S2 Table Search strategy. (DOCX)

S3 Table Clinical guidelines data extraction. (DOCX)

S4 Table Reviews data extraction. (DOCX)

S5 Table Clinical trials data extraction. (DOCX)

S6 Table Descriptive synthesis of studies with strong methodological quality. (XLSX)

S7 Table Sensitivity analysis. (DOCX)
