# Supplementary material for: Physiotherapy and related management for childhood obesity: A systematic scoping review
Source: PLoS One. 2021 Jun 14;16(6):e0252572. doi: 10.1371/journal.pone.0252572 (PMC8202913; doi:10.1371/journal.pone.0252572)
Supplement: S2 Table — (DOCX) [file pone.0252572.s002.docx]

**S2 Table. Search Strategy**

| # | Search Strategy | Search field | Source | # Articles |
| --- | --- | --- | --- | --- |
| 1 | (Pediatric OR paediatric OR child* OR juvenile OR adolescen* OR infant OR young OR teen* OR toddler OR baby OR newborn OR neonate OR school-age* OR preschool-age* OR preschooler* OR kindergarten* OR "preparatory year" OR youth)  AND  (obes* OR overweight)  AND  (physiotherap* OR "physical therap*" OR "exercise phys*" OR "physical educat*")  AND  ("gross motor" OR "motor proficiency" OR "physical literacy" OR "motor skill" OR fitness OR "pain management" OR "pain treatment" OR "physical activity" OR lifestyle OR "quality of life" OR QOL OR function* OR cardiovascular OR cardiometabolic OR BMI OR "body mass index" OR balance OR movement OR coordination OR strength OR power OR "motor control" OR cardiorespiratory OR endurance OR "aerobic fitness" OR agility OR "motor learning" OR weight OR exercis* OR education OR advice OR "screen time" OR "sedentary time")  AND  (prevent* OR treat* OR interven* OR trial* OR manag* OR effect* OR impact* OR program* OR polic* OR guideline OR curriculum OR perspective OR procedure OR recommend* OR plan) | Title/Abstract | PubMed | 1402+(61) |
| 2 | (Pediatric OR paediatric OR child* OR juvenile OR adolescen* OR infant OR young OR teen* OR toddler OR baby OR newborn OR neonate OR school-age* OR preschool-age* OR preschooler* OR kindergarten* OR preparatory year OR youth)  AND  (obes* OR overweight)  AND  (physiotherap* OR physical therap* OR exercise phys* OR physical educat* )  AND  (gross motor OR motor proficiency OR physical literacy OR motor skill OR fitness OR pain management OR pain treatment OR physical activity OR lifestyle OR quality of life OR QOL OR function* OR cardiovascular OR cardiometabolic OR BMI OR body mass index OR balance OR movement OR coordination OR strength OR power OR motor control OR cardiorespiratory OR endurance OR aerobic fitness OR agility OR motor learning OR weight OR exercis* OR education OR advice OR screen time OR sedentary time)  AND  (prevent* OR treat* OR interven* OR trial* OR manag* OR effect* OR impact* OR program* OR polic* OR guideline OR curriculum OR perspective OR procedure OR recommend* OR plan) | Abstract | Medline via OVID | 513+(15) |
| 3 | (Pediatric OR paediatric OR child* OR juvenile OR adolescen* OR infant OR young OR teen* OR toddler OR baby OR newborn OR neonate OR school-age* OR preschool-age* OR preschooler* OR kindergarten* OR "preparatory year" OR youth)  AND  (obes* OR overweight)  AND  (physiotherap* OR "physical therap*" OR "exercise phys*" OR "physical educat*")  AND  ("gross motor" OR "motor proficiency" OR "physical literacy" OR "motor skill" OR fitness OR "pain management" OR "pain treatment" OR "physical activity" OR lifestyle OR "quality of life" OR QOL OR function* OR cardiovascular OR cardiometabolic OR BMI OR "body mass index" OR balance OR movement OR coordination OR strength OR power OR "motor control" OR cardiorespiratory OR endurance OR "aerobic fitness" OR agility OR "motor learning" OR weight OR exercis* OR education OR advice OR "screen time" OR "sedentary time")  AND  (prevent* OR treat* OR interven* OR trial* OR manag* OR effect* OR impact* OR program* OR polic* OR guideline OR curriculum OR perspective OR procedure OR recommend* OR plan) | Abstract | CINAHL | 269+(4) |
| 4 | (Pediatric OR paediatric OR child* OR juvenile OR adolescen* OR infant OR young OR teen* OR toddler OR baby OR newborn OR neonate OR school-age* OR preschool-age* OR preschooler* OR kindergarten* OR "preparatory year" OR youth)  AND  (obes* OR overweight)  AND  (physiotherap* OR "physical therap*" OR "exercise phys*" OR "physical educat*")  AND  ("gross motor" OR "motor proficiency" OR "physical literacy" OR "motor skill" OR fitness OR "pain management" OR "pain treatment" OR "physical activity" OR lifestyle OR "quality of life" OR QOL OR function* OR cardiovascular OR cardiometabolic OR BMI OR "body mass index" OR balance OR movement OR coordination OR strength OR power OR "motor control" OR cardiorespiratory OR endurance OR "aerobic fitness" OR agility OR "motor learning" OR weight OR exercis* OR education OR advice OR "screen time" OR "sedentary time")  AND  (prevent* OR treat* OR interven* OR trial* OR manag* OR effect* OR impact* OR program* OR polic* OR guideline OR curriculum OR perspective OR procedure OR recommend* OR plan) | Title/Abstract | Embase | 796+(25) |
| 5 | childhood obesity intervention physiotherapy filetype:pdf |  | Google | 81 |
