# Supplementary material for: Physiotherapy and related management for childhood obesity: A systematic scoping review
Source: PLoS One. 2021 Jun 14;16(6):e0252572. doi: 10.1371/journal.pone.0252572 (PMC8202913; doi:10.1371/journal.pone.0252572)
Supplement: S3 Table — (DOCX) [file pone.0252572.s003.docx]

**S3 Table. Clinical Guidelines Extraction**

| **Organisation** | **Title, Year, Country** | **Aims/Purpose** | **Recommendations** | **iCAHE CAS (%)** |
| --- | --- | --- | --- | --- |
| American Physical Therapy Association | **Title:** Clinical Recommendations Provide Guidance for Physical Therapists Treating Childhood Obesity  **Year:** 2016  **Country:** United States of America | **Aims/Purpose:** To provide clinical recommendations provide guidance for physical therapists  treating childhood obesity. | **Recommendations:** Along with prescribing exercise, physical therapists should try to enhance self- efficacy in children and adolescents with obesity. Physiotherapist should explain why the exercise is beneficial and, along with the parent or guardian, participate in the prescribed exercise. | 42.86 |
| Academy of Pediatric Physical Therapy Fact Sheet/Resource | **Title:** FACT SHEET: The Role and Scope of Pediatric Physical Therapy in Fitness, Wellness, Health Promotion, and Prevention    **Year:** 2012  **Country:** United States of America | **Aims/Purpose:** To provide paediatric physical therapists with a rationale for incorporating health promotion strategies into practice, general considerations for health promotion interventions, and resources and references. | **Recommendations:** Non-competitive games; strength training with resistance bands; information about self- management, goal setting, social support, health, and nutrition; strength and aerobic exercises; lunchtime walking programmes; games; family sessions to identify triggers for overeating and discussions about being overweight. | 35.71 |
| Australia Physiotherapy Association | **Title:** Health and Wellbeing of Children and Young People  **Year:** August 2013  **Country:** Australia | **Aims/Purpose:** NR | **Recommendations:** Physiotherapists are well placed to assist children, young people and their families or carers with service navigation and long-term planning and management, and support self-directed care decisions, for example, in relation to individualised funding under DisabilityCare Australia. Optimal use of technology to help engage children and adolescents, such as development of apps, use of social media as an education tool on the benefits of exercise and electronic reminders of appointment times. In addition, advantage should be taken of telehealth and assessment and management strategies. Integrated care settings and collaborative models of care involving health, education is encouraged. | 35.71 |
| Australian Government (National Health and Medical Research Council) | **Title:** Summary guide for the management of overweight and obesity in primary care  **Year:** December 2013  **Country:** Australia | **Aims/Purpose:** To include key messages, recommendations, practice points, and management models in relation to the assessment and management of obesity in adults, adolescents and children. | **Recommendations:** Promote physical activity, dietary modification and healthy behaviours (drinking water and reducing screen time) to families, with the aim of weight maintenance and frequent monitoring. | 50 |
| Australian Government (National Health and Medical Research Council) | **Title:** Clinical Practice Guidelines for the management of Overweight and Obese adults, adolescents and children in Australia  **Year:** 2013  **Country:** Australia | **Aims/Purpose:** The guidelines provide detailed, evidence-based recommendations for assessing and managing overweight and obesity in adults, adolescents and children. | **Recommendations:** For children and adolescents, focus lifestyle programs on parents, carers and families. Frequent contact and monitoring (ideally 3 monthly or more frequently). For children and adolescents who are overweight or obese, recommend lifestyle change— including reduced energy intake and sedentary behaviour, increased physical activity and measures to support behavioural change. | 85.71 |
| Bagby | **Title:** Evidence based practice guideline: Increasing physical activity in schools – Kindergarten through 8^th^ grade.  **Year:** 2007  **Country:** United States of America | **Aims / Purpose:** To provide inexpensive, easy to implement, effective strategies, to increase physical activity in students. | **Recommendations:** Increase time spent in moderate to vigorous physical activity in: structured physical education classes, during recess or free play time. Reduce sedentary activity. | 50 |
| Canadian Physiotherapy Association | **Title:** Position Statement  Physical Activity for Youth and Children  **Year:** June 2006  **Country:** Canada | **Aims/Purpose:** N/A | **Recommendations:** To encourage safe, formal and informal activities: school based physical-education classes, sports, recreation, transportation, household tasks, work, and planned exercise programs. Discourage sedentary behaviours. To adhere to government recommendations of at least 20 minutes of moderate intensity physical activity and 10 minutes of vigorous physical activity and increasing this to 90 minutes a day. | 35.71 |
| European Childhood Obesity Group (ECOG) | **Title:**  Physical Activity and Play in Children who are Obese  **Year:** 2015  **Country:** Europe | **Aims/Purpose:** To introduces the reader concepts including: importance of physical activity, the relationship between physical activity and obesity, measurement of physical activity and barriers to physical activity in children who are obese | **Recommendations:** Follow Australia's Physical Activity and Sedentary Behaviour Guidelines, when prescribing activity consider FITT (Frequency, Intensity, Time, Types),age, ability, gender, preferences, socioeconomic status, set SMART goals, use 'gain-framed' messages, take time to understand barriers to obesity so to overcome them, provide education on the importance of activity (to child and family), encourage families to meet with other families outside treatment, physical activity should be aerobic and fun (i.e. games) | 35.71 |
| National Institute for Health and Clinical Excellence | **Title:** Obesity: Guidance on the prevention, identification, assessment and management of overweight and obesity in adults and children  **Year:** December 2006  **Country:** United Kingdom | **Aims/Purpose:** To stem the rising prevalence of obesity and diseases associated with it; increase the effectiveness of interventions to prevent overweight and obesity; improve the care provided to adults and children with obesity. | **Recommendations:** Multicomponent interventions including the family are the treatment of choice and should include behaviour change strategies to increase physical activity levels (overweight children may need >60 minutes/day) or decrease inactivity, improve eating behaviour and reduce energy intake as well as addressing lifestyle within the family and in social settings e.g. active play, encourage family to be more active, participation in sport and exercising at school. This should not be 'weight focused'. | 50 |
| National Institute for Health and Clinical Excellence | **Title:** Obesity in children and young people: Prevention and Lifestyle weight management programs  **Year:** 23 July 2013  **Country:** United Kingdom | **Aims/Purpose:** To cover a range of approaches at a population level to prevent children and  young people aged under 18 years from becoming overweight or obese. | **Recommendations:** Intervention should focus on improving diet, physical activity, reducing sedentary behaviour and improving self-esteem. Intervention should actively involve family members in obesity treatment and management | 50 |
| Chartered Society of Physiotherapy | **Title:** Physiotherapy Works for Obesity  **Year:** 2015  **Country:** United Kingdom | **Aims/Purpose:** NR | **Recommendations:** Provision of personalised lifestyle advice, taking  into account individual attitudes, beliefs,  circumstances, cultural and social preferences,  and readiness to change. Prescription, supervision, and progression of appropriate physical activity to increase muscle strength, flexibility, and endurance, and sustain energy output to enhance and maintain weight loss under safe and controlled conditions. Co-ordination of comprehensive and sustainable  programs of management in collaboration  with service users, other health and social care  professionals, and community services, provision of individualised physical activity program, gradual and appropriate progression, prescription of a cardiovascular training program as well as resistance exercises, prescription of moderate-intensity physical activity 30 min/day, 3-5x a week, where 30-60 mins of physical activity a week is recommended for weight management. | 35.71 |
| World Confederation for Physical Therapy | **Title:** Promoting Physical Activity in Children, the Role of Physiotherapists  **Year:** 11 January 2018  **Country:** Europe | **Aims/Purpose:** With this action/commitment the aim is to contribute to the efforts of the European Commission  and Member States to prevent and combat overweight and obesity in the young by  promoting physical activity in children. | **Recommendations:** Children 5 to 17 years should be physically active at least one hour of moderate intensity every day. Vigorous-intensity activities should be incorporated, including those that strengthen muscle and bone, at least 3 times per week. Amounts of physical activity greater than 60 minutes provides additional health benefits. Sedentary behaviour in children, 5 to 18 years, seems to correlate with increased  weight, reduced aerobic fitness, lower self-esteem and learning ability. It is recommended that children under 5 years of age should be physically active for at least 180 minutes a day, spread throughout the day. Physiotherapists should be an integral partner in the development, implementation and follow-up of high-quality physical education programs in  schools, where physiotherapists should be an integral part of school care. | 35.71 |
| World Health Organisation | **Title:** Taking Action on Obesity  **Year:** 2018  **Country:** Switzerland | **Aims/Purpose:** Aims to illustrate the progress being made, with examples of actions at national level to taking action in relation to childhood obesity. | **Recommendations:** To follow WHO's guidelines around physical activity. | 35.71 |
| World Confederation for Physical Therapy | **Title:** World Physical Therapy Day  **Year:** 2018  **Country:** UK, London | **Aims/Purpose:** To provide facts, research findings, statistics and articles to help demonstrate the contribution of physical therapists. | **Recommendations:** To follow WHO’s guidelines around physical activity.  Physical therapy assessment covers: parental beliefs around healthy childhood development, cardiorespiratory, musculoskeletal, sedentarism, sleep, physical activity levels and perceived barriers to reaching recommended levels.  Treatment includes: general health literacy education for child and parent, management of any associated conditions identified in physical assessment, age-appropriate and fun exercise training to increase physical fitness, assisting parent/s to make changes at home to prevent obesity developing or progressing, providing education and practical strategies to improve sleep and energy balance, liaison and onward referral within the interdisciplinary team. | 21.43 |
