# Supplementary material for: Physiotherapy and related management for childhood obesity: A systematic scoping review
Source: PLoS One. 2021 Jun 14;16(6):e0252572. doi: 10.1371/journal.pone.0252572 (PMC8202913; doi:10.1371/journal.pone.0252572)
Supplement: S4 Table — (DOCX) [file pone.0252572.s004.docx]

**S4 Table. Reviews Data Extraction**

| **First Author** | **Title, Year, Country** | **Aims/Purpose** | **Study Design/Inclusion Criteria** | **Key Findings/Conclusions** | **JBI CAS (%)** |
| --- | --- | --- | --- | --- | --- |
| Andrade | **Title:** The psychological effects of exergames for children and adolescents with obesity: A systematic review and meta-analysis **Year:** 2019 **Country:** Brazil | **Aims/Purpose:** To summarise and perform a meta-analysis on psychological effects of exergames on children and adolescents with overweight or obesity | **Study Design:** Systematic review  **n_s_:** 9 **n_p_:** 336 **Inclusion Criteria:** Children or adolescents (up to 19 years) with obesity (z score > +2, >90th percentile) or overweight (z score > +1, >75th percentile) and without any other comorbidities | **Key Findings:** The intervention period varied from studies that used from 1 to 100 sessions. It was observed that the Nintendo Wii was used in five studies. In one study, the interventions took place with eight different consoles: DDR, Exerbike XG, Nintendo Wii, Makoto Interactive Arena, Lightspace Play Floor, Cybex Bring, Treadwall, and Xavix System. Only one study did not report the console model. All the studies verified at least one psychological outcome with positive effects. No studies reported negative psychological effects of exergames on children and adolescents. Children and adolescents with overweight or obesity liked to practice exergames more than sedentary video games or walking.  **Conclusions:** There was an increase in self-esteem after playing exergames, an improvement in self-esteem and self-efficacy compared with the control group. Children liked to practice exergames more than other selected activities. | 54.50 |
| Atlantis | **Title**: Efficacy of exercise for treating overweight in children and adolescents: a systematic review  **Year**: 2006  **Country**: Australia | To examine quality and effectiveness of exercise in overweight children and adolescents | **Study Design**: Systematic review  **n_s_:** 14  **n_p_:**481  **Age:** 8-16 years  **Inclusion Criteria**: Children and adolescents up to 18 years, defined as being overweight or obese/overweight | **Key Findings**: Varied intensity and duration of interventions including weight training, dietary interventions.  **Conclusions**: Physically active boys and girls are at decreased risk of metabolic syndrome. Greater improvements in blood pressure, blood vessel properties (i.e. resistance and compliance), multiple coronary heart disease, dyslipidemia following exercise and dietary intervention when combined compared to the dietary intervention alone. | 90.91 |
| Brown | **Title**: A Systematised Review of Primary School Whole Class Child Obesity Interventions: Effectiveness, Characteristics, and Strategies  **Year**: 2016  **Country**: USA | To assess the effects of school-based interventions with healthy eating and PA in the prevention and treatment of obesity whilst identifying factors that contribute to effectiveness | **Study Design**: Systematic review  **n_s_:** 17  **n_p_:**16,995  **Age:** NR  **Inclusion Criteria**: Included pre and post analysis of BMI in elementary school aged children | **Key Findings**: 7 studies lasted l> 12 months, with 5 demonstrating improved BMI. 7 studies had interventions lasting 6-12 months with 4 demonstrating effectiveness. 1 study had an intervention lasting < 6 months with no BMI improvement. 8 studies had a behavioural component (social cognitive theory), with 4 demonstrating improvement in BMI.  **Conclusions**: Long term interventions that involve a multidisciplinary approach with involvement of parents are the most promising | 72.73 |
| Cislak | **Title**: Family-related predictors of body weight and weight-related behaviours among children and adolescents: a systematic umbrella review  **Year**: 2012  **Country**: USA | Explore relations between family variables including perceptions, beliefs and practices and body weight indices of children and adolescents. | **Study Design**: Umbrella review  **n_s_:** 18  **Inclusion Criteria**: Weight related outcomes such as body weight indices. Children 2-19 years. No language restrictions | **Key Findings**: 2/3 reviews demonstrated strong support for parental involvement (i.e. positive reinforcement). 3/3 reviews demonstrated strong support for CBT. 2/2 reviews with family counselling for parents/children demonstrated positive results. In correlation studies, the most common outcome was diet (used for 50% of family variables), followed by PA (46.3%). 6/27 reviews involving behavioural components demonstrated positive results.  **Conclusions**: Future treatment programs should target behaviour specific practice rather than general family involvement for effective treatment and prevention of childhood obesity | 81.82 |
| Clark | **Title**: School programs to reduce the prevalence of obesity in children  **Year**: 2008  **Country**: USA | Explore literature regarding school-based interventions in the reduction of childhood obesity | **Study Design**: Systematic review  **n_s_:** 10  **n_p_:** 5909144  **Age:** NR  **Inclusion Criteria**: School-based intervention in school-aged children | **Key Findings**: 5% of programs demonstrated effective obesity prevention in prolonged f/u. Pilot studies with selected recruitment were more effective than larger trials.  **Conclusions**: Further researched is needed to determine the optimal amount and type of PA and nutrition education to create consistently favourable outcomes in the school setting. | 36.36 |
| Craike | **Title:** Interventions to improve PA among socioeconomically disadvantaged groups: an umbrella review  **Year:** 2018  **Country:** Australia | Examine the effectiveness of interventions to improve participation in PA among disadvantaged groups; to determine the characteristics of effective interventions to provide recommendations | **Study Design:** Umbrella review  **n_s_:** 17  **n_p_:** NR  **Age:** 0-18 years  **Inclusion Criteria:** English only, targeted at disadvantaged populations or involve sub-group analysis with a socioeconomically disadvantaged population | **Key Findings:** 6/ 11 studies examining PA outcomes observed a significant effect. 2/ 3 community- and pre-school-based interventions involving parents were effective in improving PA. 3 reviews including children <12 years old demonstrated that school-based multicomponent interventions were likely to be effective, particularly if longer in duration. 3/ 3 reviews that focused on prevention, behaviours including PA- were effective. However, this was less effective in adolescents.  **Conclusions:** People from socioeconomically disadvantaged groups are less likely to meet recommended PA guidelines. The characteristics of effective interventions are unclear and are more effective in children than adolescents or older adults. Interventions should have multiple components. Future studies are needed in order to help create 'recommendations'. | 81.82 |
| De Bourdeaudhuij | **Title**: School-based interventions promoting both PA and healthy eating in Europe: a systematic review within the HOPE project  **Year**: 2011  **Country**: Belgium | Summarise evidence of school-based interventions combining nutrition, PA in children 6-18 | **Study Design**: Systematic review  **n_s_:** 27  **n_p_:**NR  **Age:** 6-18 years  **Inclusion Criteria**: Peer reviewed papers from 1990 - 2007, children in primary (6-12 years) & secondary (12-18 years), school-based intervention focused on prevention of obesity and related diseases via healthy diet and PA | **Key Findings**: 0/11 studies were rated as 'strong'. 10 were considered 'moderate', 1 considered 'weak'. 6 promoted PA and healthy eating, 2 on education alone, 4 included education and environmental elements. 4 studies reporting effects of a multicomponent (nutrition, PA curriculum, modifying school meals, providing equipment, parental involvement) intervention showed favourable results than education alone.  **Conclusions**: Combining education and environmental components (organised PA opportunities, after-school availability of space, increasing physical education curriculum, access to health food) may be preferable in the school setting for reducing childhood obesity. Future studies should focus on longer duration studies to understand if positive effects are sustained. | 81.82 |
| Delgado-Floody | **Title:** Feasibility of incorporating high-intensity interval training into physical education programs to improve body composition and cardiorespiratory capacity of overweight and obese children: A systematic review  **Year:** 2019  **Country:** Chile | To critically analyse the feasibility of HIIT programs in PE classes to improve body composition, cardiorespiratory fitness of overweight and obese students | **Study Design:** Systematic Review  **n_s_:** 6  **n_p_:** 136  **Age:** 12-18 years  **Inclusion Criteria:** Children 6-19 classified as overweight or obese. Studies including HIIT alone or combined with other methods | **Key Findings**: HIIT protocols consisted of 2-3 sessions per week, with 15 seconds of work and 15 seconds of passive or active rest for a total of 6 mins work over 6-24 weeks. Significant changes in body composition, BMI, BMI z-score, %body fat, waist circumference, sum of skinfolds, body mass, increased muscle mass and VO2 max.  **Conclusions**: HIIT can result in improvements in body composition and cardiorespiratory capacity as well as improvements in health markers. Due to time efficiency it may be more feasible to incorporate into PE class or during the school day. However, to guarantee success 2-3 sessions per week are needed. | 72.73 |
| Dias | **Title**: Exercise and Vascular Function in Child Obesity: A Meta-Analysis  **Year**: 2015  **Country**: Australia | Meta-analyse and examine the effects of exercise training on vascular function in obese children | **Study Design**: Systematic review  **n_s_:** 6  **n_p_:** 219  **Age:** 7-15 years  **Inclusion Criteria**: Classified as overweight or obese and have complete a 6-week aerobic intervention | **Key Findings**: 3 studies looked at circuit training, with aerobic exercise, aerobic exercise with resistance training and aerobic exercise with resistance and agility training. Set intensities were provided to maintain 50-65% VO2 max, 2-5 sessions a week. Pooling of the data from the 6 trials with a mean difference in FMD was 1.54%(95% CI 0.24 to 2.84), mean difference in weight was 20.55kg (95% CI 21.66 to 0.57), mean difference in BMI was 20.14kg/m2 (95% CI 20.60 to 0.32) and 3.64mL/kg/min for cardiorespiratory fitness (95% CI 1.57 to 5.70), suggesting that all above outcome measures favour exercise.  **Conclusions**: This meta-analysis indicates that  exercise is able to induce improvements in FMD in overweight and obese children and adolescents. | 90.91 |
| Doak | **Title**: The prevention of overweight and obesity in children and adolescents: a review of interventions and programs  **Year**: 2006  **Country**: Netherlands | Identify aspects of prevention programs that are likely to succeed on a large scale in the prevention of overweight and obesity in children. | **Study Design**: Systematic review  **n_s_:** 25  **n_p_:** 7,525  **Age:** 4-16 years  **Inclusion Criteria**: Children 6-19, intervention on diet or PA or both | **Key Findings**: Of the education only, exercise intervention and exercise plus education intervention, exercise only was shown to be the most effective in improving skinfold thickness. The 5 other programs that addressed fitness only, school nutrition only, home nutrition only did not result in significant reductions in skinfolds or BMI.  **Conclusions**: Mixed results suggests more research is needed to fine-tune existing interventions to determine what is effective. Prevention programs require support not only from a multidisciplinary team but multi-sectors in society such as school administration, industry and government agencies. | 63.64 |
| Engel | **Title**: Exploring the Relationship Between Fundamental Motor Skill Interventions and PA Levels in Children: A Systematic Review and Meta-analysis  **Year**: 2018  **Country**: Australia | Identify studies included an FMS and PA related intervention in children 3-12 and to determine whether there is a relationship between these outcome measures. | **Study Design**: Systematic review  **n_s_:** 19  **n_p_:**6014  **Age:** 3-11  **Inclusion Criteria**: FMS Intervention which measures PA and FMS in health children 3-12 years of age | **Key Findings**: 10/14 studies were classified as teacher led (TL) and 4/10 as teacher educated (TE). TE interventions lasted ~25 weeks with ~3 session per week, lasting ~39 minutes. TL interventions ran for 43 weeks. TL interventions showed small improvements (SMD = 0.28 [95% CI 0.14–0.43]; p = 0.0001). TE interventions showed a small, insignificant improvement (SMD = 0.23 [95% CI - 0.02 to 0.49]; p = 0.07). When all TE and TL interventions were pooled, there was a small, significant improvement (SMD = 0.36 [95% CI 0.20–0.52]; p<0.0001). This was the same for pooling of PA (SMD = 0.27 [95% CI 0.11–0.44]; p = 0.002) and MVPA (SMD = 0.22 [95% CI 0.07–0.38]; p = 0.005) alone. For SB (n = 6) a small, insignificant decrease was observed (SMD = - 0.36 [95% CI - 0.71 to - 0.01]; p = 0.05)  **Conclusions**: TL interventions 3+ times a week with associated with increased functional motor skills and increased PA, MVPA. Not all TL studies demonstrated significant relationships. Training FMS increases PA, reduces sedentary behaviour which can help reduce the burden of childhood obesity. | 90.91 |
| Errisuriz | **Title**: Systematic Review of Physical Education-Based PA Interventions Among Elementary School Children  **Year**: 2018  **Country**: USA | To systematically review evidence on experimental and quasi-experimental PE interventions implemented by PE teachers and to discuss the effectiveness of theoretically based interventions. | **Study Design**: Systematic review  **n_s_:** 12  **n_p_:**9,309  **Age:** 6-11 years  **Inclusion Criteria**: Interventions focused on increasing fitness, PA and/or maintaining body composition | **Key Findings**: Four studies added new or additional PE classes, 6 extended PE duration and either increased PA or without lengthening PE incorporated more fitness components (n=2). One study had no modifications but provided schools with sports equipment. Consistent demonstration that there was increased time spent in MVPA, with less consistent impact outside-class. Most studies utilised BMI, skinfold thickness and found a significant improvement. With PE reducing the increase of BMI with consideration for maturation and its effect on BMI. Half of all the studies assessing fitness demonstrated improvement, with only 1 study demonstrating strong positive impact on girls.  **Conclusions**: Evidence to suggest that interventions targeting PA are sufficient to increase MVPA during PE. Future work needed to explore the mixed evidence in relation to PA outside school and changes in body composition and fitness | 72.73 |
| Feng | **Title:** Systematic review and meta-analysis of school-based obesity interventions in mainland China  **Year:** 2017  **Country:** China | To qualitatively evaluate the effectiveness of school-based intervention studies | **Study Design:** Systematic review  **n_s_:** 76  **n_p_:** 72620  **Age:** 6-19 years  **Inclusion Criteria:** Primary and secondary schools in mainland China. Duration >3 months | **Key Findings:** 40/76 studies were classified as treatment studies targeting overweight/obese children. 36/76 studies were prevention studies irrespective of weight. More than half were non-RCTs. Majority of the studies were conducted at school. 7 studies included improving intensity and duration of PA in PE, health education, diet component, school policy changes, psychological counselling, physical infrastructure support. 34 treatments were effective in at least one anthropometric outcome. Interventions combining PA with education were more effective than PA alone (88.9% vs 80%).  **Conclusions:** Comprehensive treatment (PA, education) interventions were more effective than prevention. Future studies should focus on producing more high-quality studies to confirm effectiveness. | 90.91 |
| Garcia-Hermoso | **Title**: Is high-intensity interval training more effective on improving cardiometabolic risk and aerobic capacity than other forms of exercise in overweight and obese youth? A meta-analysis  **Year**: 2016  **Country**: Spain | To determine the effectiveness of HIIT interventions on cardiometabolic risk and aerobic capacity in overweight and obese youth compared with other forms of exercise | **Study Design**: Systematic review  **n_s_:** 9  **n_p_:** 274  **Age:** 6-17 years  **Inclusion Criteria**: Children and/or adolescents (6-17 years) classified as overweight or obese; HIIT intervention, >4 weeks duration | **Key Findings**: Majority of programs used walking and running, running or cycling only. 15 seconds to 4 minutes with passive or active recovery between repetition of 100-120% maximum aerobic speed, between 80-95% maximal heart rate and 80-90% VO2 max at 100% maximum velocity sprint. Majority of interventions lasted 12 weeks. 3 studies lasted 4 or 6 weeks. Differences were observed between HIIT and other forms of exercise for systolic blood pressure (SMD = 0.39, 95% CI 0.09 to 0.69; p = 0.01; I2 = 1%; WMD=-3.63 mmHg) (greater reduction with HIIT programs). VO2max with HIIT programs had a higher increase (SMD = 0.59, 95% CI 0.17 to 1.01, p = 0.006; I2 = 35%; WMD= 1.92 ml/kg/min) than other forms of exercise.  **Conclusions**: HIIT interventions have capacity to improve aerobic capacity, blood pressure when compared to moderate intensity training or of lower volume activity. HIIT is perceived as more enjoyable therefore having implications for participation adherence and overall effectiveness of interventions | 72.73 |
| Garcia-Hermoso | **Title:** Exercise- based interventions and C-reactive protein in overweight and obese youths: a meta-analysis of randomised controlled trials  **Year:** 2016  **Country:** Chile | To examine the evidence for the effectiveness of exercise interventions on modifying the levels in overweight/obese youth | **Study Design**: Systematic review  **n_s_:** 9  **n_p_:**635  **Age:** 7-17  **Inclusion Criteria**: Interventions including physical exercise or dietary restriction. Subjects aged 6-18 years classified as overweight or obese | **Key Findings**: Content of interventions focused on sports such as soccer, basketball, handball, running, swimming, water games etc. 2 studies focused on body-weight strength training or elastic bands. Studies varied in duration but the majority lasted 12 weeks including 4x 30-minute session a week. Of the 9 included RCTs spanning the diverse interventions, results suggest a nonsignificant trend in ability to reduced C-reactive protein  **Conclusions**: The number of RCTs was small, interventions varied largely with little information about maturation, compliance, exercise intensity etc. Therefore, lack of homogeneity and large consistencies observed. Future research is needed to clarify recommendations | 100 |
| Garcia-Hermoso | **Title:** Exercise, health outcomes, and paediatric obesity: A systematic review of meta-analyses  **Year:** 2019  **Country:** Chile | To explore the effects of exercise-based interventions alone and the health outcomes (anthropometry, body composition and cardiometabolic, hepatic, vascular and cardiorespiratory fitness parameters) in overweight and obese children and adolescents | **Study Design:** Umbrella review  **n_s_:** 18  **Inclusion Criteria:** Children classified as overweight/obese | **Key Findings**: When results pooled, small overall effect size for body weight (g=-0.23, 95%CI, -0.41 to -0.05; p=0.013, I2=35.8%). Small reduction in BMI was observed in 3 meta-analyses, with effect sizes from small to medium (g= -0.36=-0.78). The pooled effect size of fat mass % was small (g=-0.38, 95% CI, -0.55 to -0.21; p<0.001; I2=45.2%). Duration and weekly frequency did not seem to influence the parameters studied except for body mass and fat-free mass. Programs of 4-12 weeks were only effective in improving BMI, BMI z-score and visceral/subcutaneous fat. Programs of 1500 minutes or more were effective in improving in BMI, BMI z-score, fat mass (%), visceral and subcutaneous fat. Aerobic programs improved LDL-C, TG, fasting glucose, fasting insulin, HOMA-IR, intrahepatic fat, systolic blood pressure, flow-mediated dilation. Resistance programs only improved fasting glucose and insulin, intrahepatic fat. Combination programs improved HDL-C, fasting glucose and leptin  **Conclusions**: Exercise based interventions improved some anthropometric and cardiovascular parameters as well as cardiorespiratory fitness. The evidence concerning other parameters need deeper study. Generally aerobic programs demonstrate improvements in most parameters (15/26). For cardiometabolic and vascular parameters, programs 4-12 weeks with a total of 1500 mins (or 3 sessions per week for 60 mins) were effective in improving HDL-C, fasting glucose, fasting glucose, fasting insulin, HOMA-IR, intrahepatic fat systolic BP and carotid intima-media thickness. | 90.91 |
| Guerra | **Title**: PA and nutrition education at the school environment aimed at preventing childhood obesity: evidence from systematic reviews  **Year**: 2016  **Country**: Brazil | Explore school-based interventions to reduce overweight/obese children and adolescents | **Study Design**: Umbrella review  **n_s_:** 33  **Inclusion Criteria**: School environment aiming at preventing and/or reducing overweight/obese children (theoretical and/or practical contents of PA and/or nutrition education) | **Key Findings**: Nine reviews highlighted the effectiveness of interventions less than 6 months in duration/ 8 looked at the importance of parental involvement. One review looked at gender as a differentiating factor for effectiveness. It found that structural interventions were more effective on boys and behavioural interventions were more effective for girls  **Conclusions**: Parental involvement is crucial. Further studies are also needed to investigate strategies to best address differences in gender and age in identifying the best strategies to decrease childhood obesity. | 45.45 |
| Han | **Title:** Effectiveness of exercise intervention on improving FMS/MC in overweight/obese children and adolescents: A systematic review  **Year:** 2018  **Country:** Australia | To determine the effectiveness of exercise and PA on improving fundamental movement skill and motor coordination in overweight/obese children and adolescents | **Study Design:** Systematic review  **n_s_:** 17  **n_p_:** 2495  **Age:** 4-17 years  **Inclusion Criteria:** RCTs, interventional and longitudinal studies. FMS/MC measurements. Classified as obese/overweight participants between 0-18 years. Exercise or PA interventions including structured or unstructured training or PA based in any location (school, clinics etc) | **Key Findings:** All exercise/PA interventions were supervised programs. Duration averaged at 36 weeks. 3 implemented exercise focusing on FMS improvement. 8 focused on strength, flexibility training, aerobic, gymnastics. The remaining 6 included activities such as swimming, cycling, active break, active video games etc. Majority of interventions showed that exercise had a positive effect on locomotor skill competence in sprint, jumping agility and gait.  **Conclusions:** PA intervention is highly effective at improving FMS/MC skill. 17 studies demonstrated improvements in overweight and obese children. To maximise skill improvements in this population enjoyable interventions that focus on FMS and MC competence are needed to reduce obesity. | 81.82 |
| Harris | **Title**: Effect of school-based PA interventions on body mass index in children: a meta-analysis  **Year**: 2009  **Country**: Canada | To determine whether school-based PA interventions improve children's BMI | **Study Design**: Systematic review  **n_s_:** 18  **n_p_:** 18141  **Age:** 5-18 years  **Inclusion Criteria**: 5-18 years. To have complete both pre-post measures. Minimum 6-month duration interventions | **Key Findings**: School based interventions did not improve BMI thus not likely to significantly affect BMI. Reasons for failure were unclear, however it is suspected to be related to dose, quantity and adherence.  **Conclusions**: PA should be included and promoted in schools, however findings suggest that future studies that improve on previous methodologic weakness are needed should policies be successfully implemented to improve children’s body composition. Interventions involving diet in the school setting should also be pursued. | 90.91 |
| Lobelo | **Title**: School-based programs aimed at the prevention and treatment of obesity: evidence-based interventions for youth in Latin America  **Year**: 2013  **Country**: USA | examine the effectiveness of school-based intervention aimed at preventing or treating obesity among youth in LA. | **Study Design**: Systematic review  **n_s_:** 10  **n_p_:** NR  **Age:** 6-14.5 years  **Inclusion Criteria**: Obesity related interventions (not general health promotion) | **Key Findings**: 1 intervention included a parental component. Intervention ranged from 3-24 months ranging 1-5x/week. 1 Intervention had a school and home component. 3 interventions aimed to increased PA and healthy eating, 5 interventions aimed to increased PA and 2 focused on exclusively on healthy eating. 3 Interventions were led by health professions (nutritionists, PE teachers), 2 by teachers and 1 by trained personal of unknown training. Despite differing focuses, it appeared that prevention focused interventions were more successful than treatment.  **Conclusions**: Sufficient evidence to support school-based intervention to prevent overweight and obesity. Most interventions included multicomponent strategies addressing energy balance, focused on prevention, had long term follow ups (>6 months), involved teachers, allied health professionals, stronger study designs. Future studies should replicate and refine current research | 63.64 |
| Morgan | **Title**: Caregiver Involvement in Interventions for Improving Children's dietary intake and physical activity behaviours  **Year**: 2020  **Country**: United States | **Aims/Purpose:** To assess effects of caregiver involvement in interventions for improving children’s, dietary intake and PA behaviours | **Study Design:** Systematic review  **n_s_:** 23 **n_p_:** 12,192 **Inclusion Criteria:** RCTs, quasi-RCTs, children between 2-18 years, no pre-existing conditions, children not from orphanages and school environments. | **Key Findings:**  No difference in dietary behaviour change, total PA when comparing carer intervention with interventions without. Combined dietary and PA intervention observed a small positive impact with the caregiver component on children’s' sugar-sweetened beverages. **Conclusions:** Insignificant evidence to support inclusion of caregiver involvement in interventions to improve children's dietary intake or PA behaviour, or both. | 72.70 |
| Niemeier | **Title**: Parent participation in weight-related health interventions for children and adolescents: a systematic review and meta-analysis  **Year**: 2012  **Country**: USA | To review child and adolescent weight-related health intervention characteristics with a focus on parental participation. | **Study Design**: Systematic review  **n_s_:** 36  **n_p_:** 7455  **Age:** 2-19 years  **Inclusion Criteria**: Weight-related interventions. Children and adolescents. January 2004- December 2010. | **Key Findings**: Parental involvement significantly contributes to intervention success. 90% of studies were preventative interventions (prevent BMI increase as opposed to BMI reduction). The average for treatment group was 0.28kg/cm2 compared to control of 1.17kg/cm2  **Conclusions**: Parental involvement contributes to the success of interventions and should focus on ways parents can incorporate behaviour changes strategies to aid in reducing obesity | 63.64 |
| Oliveira | **Title**: Effects of active video games on children and adolescents: A systematic review with meta-analysis  **Year**: 2019  **Country**: Brazil | Effects of active video games on children and adolescents in reducing obesity and increase PA in children and adolescents | **Study Design**: Systematic review  **n_s_:** 12  **n_p_:** 1016  **Age:** 7-19 years  **Inclusion Criteria**: Randomised controlled trials and quasi randomised controlled trials investigating the efficacy of AVGs compared with minimal intervention in children aged 2-19 years were included | **Key Findings**: Dance games were the most common intervention (n=6), followed by sports games (n=2), a combination (n=3) and a peripheral device (n=1). 11 trials reported weight-related outcomes. High quality evidence suggest that video games were more effective than no intervention for BMI, zBMI data in short (6 trials, SMD=-0.34; 95%CI: -0.62 to -0.05) intermediate (2 trials, SMD=-0.36, 95% CI: -0.71 to -0.01). However moderate quality evidence suggests that active video games were in fact not more effective than the control for increasing PA level in the short term (SMD=0.96, 95% CI: -0.19-0.31)  **Conclusions**: Active video games are more effective at reducing BMI than no intervention in both short term and immediate follow up. However, it did not increase PA level in youth. Further studies investigating the long-term effects are needed. | 81.82 |
| Salmon | **Title**: Promoting PA participation among children and adolescents  **Year**: 2007  **Country**: Australia | To review effectiveness of physical interventions in children 4-19 | **Study Design**: Systematic review  **n_s_:** 85  **n_p_:** NR  **Age:** 4-19 years  **Inclusion Criteria**: Children 13-19. Sample size >16. Excluded if reported fitness only outcomes. | **Key Findings**: 5 interventions targeted curriculum strategies only (only 1 was effective), 7 were school based (teaching, multimedia and internet intervention). Only 1 intervention resulted in significantly increased PA participation. 2 studies combined curriculum and PE strategies. Both reported some positive effect. Ineffective interventions had various methodological weakness. Other interventions involved new PE strategies, changes in the school environment, changes in curriculum, targeting activity breaks, advice and counselling. 9 interventions used a combination of school curriculum changes, family components with 7 reporting success and 2 reporting no effects. Six even included community involvement.  **Conclusions**: It is recommended that future interventions include a longer follow-up period (1-2 years) to determine maintenance effects. In addition, there is a need to develop and test theoretical approaches through mediator analyses. Although there is some evidence of intervention effectiveness in both the school and family setting, the use of motivationally tailored strategies and program delivery in the primary care setting. | 54.55 |
| Sharma | **Title**: School-based interventions for childhood and adolescent obesity  **Year**: 2006  **Country**: USA | Review school-based interventions for preventing childhood obesity | **Study Design**: Systematic review  **n_s_:** 22  **n_p_:** NR  **Age:** 5-17  **Inclusion Criteria**: School setting. Research published between 1999-2004 | **Key Findings**: Eleven interventions from the US and UK. Majority of interventions targeted both PA and nutrition with some addressing sedentary behaviours. Most interventions had behavioural components (social cognitive theory), focusing on individual level change in the short term. Modest and mixed results in terms of improvements in indicators of obesity.  **Conclusions**: Only 11 interventions were found with mixed results. Future population-based prevention programs are needed. | 45.45 |
| Stuart | **Title**: An integrative review of interventions for adolescent weight loss  **Year**: 2005  **Country**: USA | Describe findings from a critical review of weight loss interventions with the adolescent population. | **Study Design**: Systematic review  **n_s_:** 17  **n_p_:** 811  **Age:** 11-19 years  **Inclusion Criteria**: Weight loss interventions. Participants between 11-10. Between 1980-2003 | **Key Findings**: Interventions included a range of components e.g. Parental, exercise, diet, medical intervention, monetary rewards and incentives, telephone and mail-based intervention, behavioural therapy. Thirteen used group behavioural intervention, 2 were individual and the remaining 2 were physiological interventions.  **Conclusions**: Inconsistent findings re. weight loss despite multiple approaches. It is still thought that prevention is the better choice, but further research is needed. | 81.82 |
| Sun | **Title**: Aerobic exercise VS Control  **Year**: 2013  **Country**: Australia | Effect of obesity intervention on adiposity, fitness, cardiometabolic measures in school-aged children and adolescence | **Study Design**: Systematic review  **n_s_:** 18  **n_p_:** 6207  **Age:** 5-18 years  **Inclusion Criteria**: N/A | **Key Findings**: Eighteen RCTs. Duration varied from 6 weeks to 3 years.  **Conclusions**: Interventions consistently increased fitness with larger, higher quality RCTs providing strong evidence for interventions to decrease skin-fold thickness, increase fitness and high-density lipoprotein cholesterol. Future studies are required to further explore the evidence for BMI, bodyfat, waist circumference, blood pressure, triglycerides, low-density lipoprotein cholesterol and total cholesterol in relation to these interventions | 90.91 |
| Vasconcellos | **Title**: PA in overweight and obese adolescents: systematic review of the effects on physical fitness components and cardiovascular risk factors  **Year**: 2014  **Country**: Brazil | Explore effect of PA interventions on body composition, physical fitness components, hemodynamic variables, biochemical markers, endothelial function and low-grade inflammation in overweight and obese adolescents | **Study Design**: Systematic review  **n_s_:** 24  **n_p_:** 1635  **Age:** 12-18 years  **Inclusion Criteria**: Children 11-19. Weight-loss interventions | **Key Findings**: Fifteen of the 24 studies were PA only. Nine studies were PA plus lifestyle or dietary intervention. Of the 22 studies that looked at BMI, 15 were effective at reducing BMI. Six studies reported no significant change and 1 reported an increase in BMI. Of the 15 studies that looked at both BMI and fat percentage, 6 trials reported a decrease in body fat. Nine reported no significant differences. In 13 studies, the duration of training was <1hour. In 9 studies the intensity was low to moderate. Isolated and combined effects of frequency, duration and intensity of PA were not addressed. Therefore, making an effective 'dose' unclear. Nine studies applied cycle ergometer, 4 observed effects of school activities and 1 involved dance. A quarter of studies demonstrated a decrease in BMI whilst the remaining 3 did not. The predominant type of PA was running, either performed continuously or with low-high intermittent intensity. Of the 4 school interventions, 3 reported no significant change.  **Conclusions**: PA programs are likely to induce favourable adaptations on body composition and physical fitness as well as biochemical variables, inflammatory markers, endothelial function of overweight and obese adolescents. | 72.73 |
| Yuksel | **Title**: School-Based Intervention Programs for Preventing Obesity and Promoting Physical Activity and Fitness: A systematic review with meta-analysis  **Year**: 2019  **Country**: Turkey | **Aims/Purpose:** To explore possibility of school-based interventions on promoting PA and physical fitness as well as preventing obesity | **Study Design:** Systematic review  **n_s_:** 19 **n_p_:** 9,586 **Inclusion Criteria:** Primary and secondary school students; interventions including organising PE courses, increasing duration of PE courses, increasing PA opportunities in class, changing the school environment, extracurricular activities; published between 2010-2019; measurements at least one of the outcomes: body composition, waist circumference, skinfold, PA level and physical fitness; English language. | **Key Findings:** Significant improvement in obesity in 4/8 studies, with no significant difference founds in 3. In studies more focused on PA, BMI observed improvements. PA levels increased in a significant proportion of the 13 studies. 7/10 observed significant increase. 4/7 studies observed improvement in aerobic endurance.   **Conclusions:** 18/19 studies achieved significant improvements in at least one variable. It is suggested that the content and details of the school-based intervention program are the most important factors in determining the efficiency in the studies examined. To prevent obesity and promote PA and physical fitness, the characteristics of more effective programs should be examined in detail. From what we know programs should be multicomponent and longitudinal to foster lifelong habits. | 81.8% |
| Zenzen | **Title**: Integrative review of school-based childhood obesity prevention programs  **Year**: 2009  **Country**: USA | Describe the degree of variability in methodological approaches of school-based obesity prevention programs that utilise one or more of the following interventions: dietary, PA, healthy lifestyle education, and/or parental involvement | **Study Design**: Systematic review  **n_s_:** 16  **n_p_:** 10407  **Age:** 4-18 years  **Inclusion Criteria**: School-based childhood obesity prevention programs. Children 4-18. | **Key Findings**: Intervention duration ranged from 5 weeks to 8 years. Fourteen of 16 studies implemented dietary interventions which were diverse and included classroom based nutritional education sessions, introduction of dietary practices to increase lower-fat foods into their diet, interactive age-appropriate games and more. These interventions also included PE programs which were fairly ambiguous. Fourteen of 16 held formal classroom sessions. Nine of 16 studies integrated the family into the intervention. Nine of 16 evaluated the effect of their intervention based on BMI.  **Conclusions**: The most effective school-based obesity intervention programs should be guided by behavioural theoretical frameworks involving dietary, PA modification, healthy lifestyle education, parental involvement. Duration also should be of long enough duration to observed sustained benefits. Involvement of school food programs also appear effective in creating habits at school which are reinforced at home (parental involvement). Future research is needed to provide definitive recommendations | 72.73 |

*Note. Abbreviations:* CI – confidence interval, SMD- standard mean difference, f/u - follow-up, n_p_ - population number, n_s_ - number of studies, NR- not recorded, RCT- randomised controlled trials, BMI- body mass index, PA- physical activity, PE- physical education, FMS/MC- fundamental movement skills/motor coordination
