# Supplementary material for: Physiotherapy and related management for childhood obesity: A systematic scoping review
Source: PLoS One. 2021 Jun 14;16(6):e0252572. doi: 10.1371/journal.pone.0252572 (PMC8202913; doi:10.1371/journal.pone.0252572)
Supplement: S5 Table — (DOCX) [file pone.0252572.s005.docx]

**S5 Table Clinical Trials Data Extraction**

| **Author** | **Title,**  **Year,**  **Country** | **Aims/Purpose,**  **Sample Size (n=),**  **Participants** | **Duration,**  **Intervention** | **Key Findings, Conclusions** | **MMAT CAS (%)** |
| --- | --- | --- | --- | --- | --- |
| *Aires | **Title:** Exercise intervention and cardiovascular risk factors in obese children. Comparison between obese youngsters taking part in a physical activity school-based program with and without individualised diet counselling: the ACORDA project   **Year:** 2016  **Country:** Portugal | **Aims/Purpose:** To evaluate and compare the changes that occurred in several CVD risk factors with individualised and without dietary counselling   **Sample Size:** 46  **Participants:** Overweight/obese children aged 6-16 years old | **Duration:** 8 months  **Physical Activity:** Additional 2 hours of after-school sessions to the 3 hours of PE (included in the curriculum) for a total of 5 hours/week. Physical exercise sessions included 15 minutes of warm-up with aerobic endurance and flexibility, 30 minutes of working circuits for aerobics, strength training, co-ordination and balance with balls, bows, strings and callisthenic exercises, 10 minutes of games to promote the enjoyment and 5 minutes of stretching.  **Diet/Nutrition + Healthy Lifestyle Education + Family-Based:** Dietary counselling was delivered every 3 months and aimed to give healthy dietary knowledge for the whole family. All sessions included a family-based intervention involving a multidisciplinary team (paediatrician, nutritionist and psychologist). A recorded-dietary plan was analysed, weak and strong points were discussed, and an agreement for the next 3 months was assumed by the children/adolescents, the family and the team. | **Key Findings:** SBP, sedentary time improved, trunk fat %, SBP, TC, sedentary time, MVPA and kcal/day (p<0.05).  **Conclusion:** School-based interventions are successful in promoting health behaviours, increasing habitual physical activity and that individualized support has the potential to create greater more impactful change. | 85.71 |
| Alexander | **Title:** A prospective multifactorial intervention on subpopulations of predominately Hispanic children at high risk for obesity  **Year:** 2014  **Country:** USA | **Aims/Purpose:** To evaluate the efficacy of multiple interventions as compared to no intervention. To measure the responses to these interventions as a function of the degree of body habitus, as indicated by BMI subgroups.  **Sample Size:** 561  **Participants:** Children aged 6-8 years in first and second grade | **Duration:** 6 months  **Physical Activity:** Daily recess time was structured into organised play 2 days per week to increase exercise. 1 extra PE class was given daily for a total of 150 extra minutes of exercise per week. Classes included activities such as relay races and obstacle course games.  **Diet/Nutrition:** Children who stayed on-campus after school received 2x 30-minute cooking classes per week. Assemblies were held every 3 months that exposed the children to nutritional and recreational topics. Nutritional education in the classroom consisted of weekly 45-minute courses. A “Chef in the Classroom” program occurred bi-monthly in each class. The 1-month intersessions between quarters contained 1 or 2 week-long “Health Camps.”  **Healthy Lifestyle Education + Family-Based:** Parents of children who exceeded the 85th BMI percentile received 1-on-1 counselling twice. Topics included learning about the importance of proper sleep time, selecting the right produce, the importance of non-sweetened beverages, reducing screen time to <2 h, and increasing daily activities. All parents were requested to attend monthly group activities regarding nutrition, obesity, and physical activity.  **Environment:** Teachers received 2 seminars where health statistics and nutritional curricula were introduced. Administrators received monthly 15-minute nutritional presentations to highlight or update the curricular modifications that were occurring. | **Key Findings:** No statistical difference in BMI between groups (p<0.64) for children below the 25th percentile BMI. 25th-95th percentile children demonstrated significantly less BMI gain and for 75-95th percentile, the mean weight gain was 0.074kg/ms compared to the control of 0.68kg/m2 (p<0.00007).  **Conclusion:** Interventions involving augmenting physical activity, nutrition education, fixed-meals, motivational interventions at school decreased BMI gain over the 6-month period. Children in the 75-95th percentile observed the greater BMI reduction. Further research is needed to explore children that fall into neighbouring percentiles. | 57.14 |
| Alonso- Fernandez | **Title:** Impact of a high intensity interval training protocol on body composition and VO2max in adolescents  **Year:** 2019  **Country:** Spain | **Aims/Purpose:** To verify the impact of based on functional exercises on the VO2max and % BF of variables in the context of PE classes in an adolescent population  **Sample Size:** 26  **Participants:** Children aged 15-16 years | **Duration:** 9 weeks  **Physical Activity:** The intervention group did 14 functional sessions (2 sessions per week), while the control group did the 14 ordinary scheduled warm-ups (2 sessions per week). It consists of a 4-minute workout block where eight 20-second intervals of maximal effort are alternated with 10-second rest periods. The proposed exercises are based on functional bodyweight exercises and involves multiple articulations and muscle groups. This included squat jump, push up, high skipping, burpees, isometric front plant, multi-jumps on the bench, mountain climbers, lateral sprints of 5 metres. Workload increased by "Tabata" Blocks from week 1 to 7. | **Key Findings:**  Weight, BMI was not significant. Post-hoc analysis demonstrated significant % BF reduction (p<0.01) FM (p<0.05), FFM compared to pre-test (p<0.01) and improvements in VO2 max (p<0.001).  **Conclusion:**  HIIT is a promising strategy to combat obesity in children and adolescents. | 57.14 |
| *Alves | **Title:** Multicomponent training with different frequencies on body composition and physical fitness in obese children  **Year:** 2019  **Country:** Portugal | **Aims/Purpose:** To compare the effects of training with difference exercise frequencies on body composition and physical fitness   **Sample Size:** 40  **Participants:** Children aged 12-15 with overweight/obesity | **Duration:** 10 weeks  **Physical Activity:** Training sessions in addition to physical education classes. Consisted of warm up (5 minutes), aerobic exercises or combined aerobic and strength training (50 minutes), cool down with static stretching (5 minutes). Aerobic tasks set to 75% VO2max | **Key Findings:** Curl-up and BMI variables showed small effect size with significant differences (p=0.036, 0.007, respectively). No significant differences in VO2max, sit and reach, push-ups, BF.  **Conclusion:** The intervention provided positive results on body composition and physical fitness components. | 28.60 |
| An | **Title:** A school-based mentoring program developing healthy behaviors of adolescents with intellectual and developmental disabilities: A pilot feasibility study  **Year:** 2019  **Country:** USA | **Aims/Purpose:** To examine a school-based intervention using the "I can do it!" model of adolescents  with intellectual disability   **Sample Size:** 14  **Participants:** Children aged 12-15 years from segregated special education classrooms | **Duration:** 4 months  **Physical Activity + Diet/Nutrition:** A 60-70-minute mentoring session took place once a week at the mentee's school for 14 weeks during the lunch hour. The mentorship involved tracking and ensuring the choice of food and beverage and participating in physical activity. Mentees were encouraged to record daily the time spent in physical activity, types of physical activity, step counts using a pedometer, and fruits/vegetable and water intake.  **Environment:** Mentors participated in 2.5-hour training session lead by an adapted PE specialist, involving a program overview, mentor's responsibility, pre/post measurements, knowledge of intellectual disability including communication strategies, and self-report of the mentoring sessions. | **Key Findings:** WC and BMI z-score did not change. Improved physical activity time (p<0.0001), daily water plus fruit and vegetable consumption (p<0.001). Frequency of physical activity not significantly increased (p=0.31).  **Conclusion:** School-based settings are appropriate in improving nutritional behaviour, exercise time but less so in anthropometric measures. Future research is needed. | 85.71 |
| Angelopoulos | **Title:** Changes in BMI and blood pressure after a school-based intervention: the CHILDREN study  **Year:** 2009  **Country:** Greece | **Aims/Purpose:** To evaluate the effectiveness of a school-based intervention program on obesity indices and blood pressure in primary school children  **Sample Size:** 646  **Participants:** Children from first to sixth grade | **Duration:** 12 months  **Physical Activity:** Special emphasis was placed on increasing children’s fun and excitement for exercise. 2x45-minute PE sessions per week (a total of about 60 classes per year) were delivered in the playground. The sessions were enjoyable, fitness-oriented (rather than motor-oriented) and of moderate intensity. Little attention was placed on competition and winning while verbal rewards were given for all levels of effort and ability.  **Health Lifestyle Education + Diet/Nutrition:** An intervention material was developed and constituted of a student’s workbook and a teacher’s manual. The themes covered through this manual were self-esteem, body image, nutrition, physical activity, fitness and environmental issues. The material was implemented for 1–2 hours per week. Educational material and motivational intervention implemented promoted the consumption of a balanced diet.  **Environment:** Playgrounds and school yards were accessible for children to play after the end of the curricular program.   **Family-based:** Parental support was achieved via meetings. Parents were given a file containing the results of their child’s medical and nutritional assessment. The intervention focused on increasing parental involvement and availability of fruits and vegetables at home and school. Parents were advised to support their children in being physically active rather than to encourage sedentary behaviours. | **Key Findings:** Increased MVPA, daily consumption of fruits, decreased fat/oil, sweets/beverages and decrease in dairy products in the intervention group whereas the control demonstrated decrease in MVPA daily consumption of fruits with an increase in consumption of fat/oil, sweets/beverages and increase in dairy consumption.  BMI, SBP, DBP were not significant (β = –0.08, p=0.123, β = –0.11, p=0.065, β = –0.13, p=0.053 respectively).  **Conclusion:** Developing a social and physical environment that encourages and promotes good eating behaviour and extra-curricular physical activity have a positive effect on BMI and blood pressure and therefore towards tacking chronic disease in early life. | 85.71 |
| Ariza | **Title:** The Incidence of Obesity, Assessed as Adiposity, Is Reduced After 1 Year in Primary Schoolchildren by the POIBA Intervention  **Year:** 2019  **Country:** Spain | **Aims/Purpose:** The aim of this study was to evaluate the effectiveness of a multicomponent school-based intervention  **Sample Size:** 2450  **Participants:** Children aged 9-10 years in 3rd and 4th grade | **Duration:** 12 months  **Physical Activity:** At school, the intervention was intended to ensure a minimum of 2 hours of PE/week. Teachers promoted the practice of ≥2 hours of activities outside school. The teachers controlled the level of extracurricular physical activity and promoted pupil and family participation in free or organized recreational activities on weekends.  **Healthy Lifestyle + Diet/Nutrition + Family-based:** Families collaborated in the classroom intervention by carrying out some activities from the family booklet at home. They also received fortnightly information about free sporting events taking place in their neighbourhoods and information on weekend opening of the playgrounds in some schools in their district. Families were invited to participate together with their children in the workshop “A plan for change,” (1.5 hours) which provided families with the skills to achieve a healthy and balanced diet, improve their sleep, hygiene, and use of leisure time, and reduce screen time.   **Environment:** All teachers were trained in the main objectives and contents of the program and the materials to be used. The teachers’ material includes a guide with instructions on how to implement the program, a digital learning platform with audio-visual and interactive material on diet and physical activity, and an interactive videogame test in the last session. | **Key Findings:** No significant differences for BMI. Increased positive changes: water, fruit, meets, sweets, fried potatoes as well as “having dinner in front of the TV”, “starting to do extracurricular physical activity”, daily screen use in intervention compared to control group.  **Conclusion:** The multicomponent, multilevel programme reduced the incidence of childhood obesity as measured by adiposity in a feasible, sustainable manner. | 100 |
| *Ashem | **Title:** Physical therapy protocol for obese adolescent girls with polycystic ovarian syndrome: A within-subject design  **Year:** 2019  **Country:** Egypt | **Aims/Purpose:** To investigate the effect of physical therapy on anthropometric and hormonal outcomes   **Sample Size:** 20  **Participants:** Children aged 14-18 years with BMI 30-35 +/- PCOS | **Duration:** 6 months  **Physical Activity:** Aerobic exercise program 3 sessions/week. Warm up: pedalling on an electronic bicycle ergometer at 60 rpm without load (5 minutes). Active stage: pedalling at 60 rpm with load at 60% maxHR (30 minutes). Cool down: pedalling at a speed of 60 rpm without load (5 minutes).  **Diet/Nutrition:** Specific diet therapy (1200 Kcal/day for 3 days/week and 800 Kcal/day for 4 days/week). The calories were divided into small frequent meals (55% carbohydrates, 30% fat and 15% proteins). | **Key Findings:** Statistically significant difference in weight, waist-hip ratio, LH/FSH ratio pre and post 3 months of intervention, and 6 months (p<0.0001).  **Conclusion:** Physical therapy for obese adolescent girls with PCOS effectively decreased weight, waist-hip ratio and LH/FSH ratio. | 100 |
| *Augustijn | **Title:** Weight loss, behavioural change, and structural neuroplasticity in children with obesity through a multidisciplinary treatment program  **Year:** 2019  **Country:** Belgium | **Aims/Purpose:** To evaluate the effect of a multidisciplinary program on motor competence, executive functioning, and brain structure  **Sample Size:** 51  **Participants:**  Children aged 8-12 years +/- obesity | **Duration:** 5 months  **Physical Activity:** Physical activity (2032 +/- 26 kcal/day) was provided as physiotherapy sessions and exercise sessions (3 hours/week) with team sports and group games for 1 hour/day (5 hours/week). **Diet/Nutrition:** Diet consisted of 3 main meals and 3 healthy snacks daily (1593 +/- 595 kcal/day) and information sessions (30 minutes/week) on healthy food choices.  **Healthy Lifestyle Education:** Cognitive behavioural therapy sessions (30 minutes/week) about solution-based thinking to improve behavioural modification. | **Key Findings:** Significant interaction effects between time and group for height (p=0.008), body weight (p<0.001), % BF (p<0.001), and BMI (p<0.001). Obese group demonstrated impaired motor competence compared to control.  **Conclusion:** A multidisciplinary residential program is effective for weight loss, inhibition control and/or poor balance. | 100 |
| *Augustijn | **Title:** Role of Motor Competence and Executive Functioning in Weight Loss: A Study in Children with Obesity  **Year:** 2018  **Country:** Belgium | **Aims/Purpose:** To assess motor competence and executive functioning in children with obesity  **Sample Size:** 64  **Participants:** Obese children aged 7-11 years | **Duration:** 5 months  **Physical Activity:** Physical activity (2032 +/- 26 kcal/day) was provided as physiotherapy sessions and exercise sessions (3 hours/week) with team sports and group games for 1 hour/day (5 hours/week). **Diet/Nutrition:** Diet consisted of 3 main meals and 3 healthy snacks daily (1593 +/- 595 kcal/day) and information sessions (30 minutes/week) on healthy food choices.  **Healthy Lifestyle Education:** Cognitive behavioural therapy sessions (30 minutes/week) about solution-based thinking to improve behavioural modification. | **Key Findings:** Significant time by group effects found for body weight (p≤.001), %BF (p≤.001), WC (p≤ .001), and BMI (p≤.001), balance skills (p=.008) but not for motor competence, manual dexterity, ball skills (p>.05) or total intracranial volume (p>0.5)  **Conclusion:** The multidisciplinary treatment program is efficacious in treating children with obesity. | 100 |
| *Azad | **Title:** Effects of aerobic exercise on lung function in overweight and obese students  **Year:** 2011  **Country:** Iran | **Aims/Purpose:** To determine the effect of continuous treadmill running on lung function in overweight and obese students’ lung function were significantly lower than the predicted values  **Sample Size:** 30  **Participants:** Children aged 15-18 years with BMI>25 or BMI>30 | **Duration:** 24 weeks  **Physical Activity:** The intervention group participated in the exercise training program (3 days/week) of continuous treadmill running (grade=0%), at a minimum speed required to achieve/maintain 75-85% maxHR. The running time was 15 minutes at the first session, increasing by 1 minute every 2 sessions up to a maximum of 30 minutes. Once the running time reached 30 minutes, it was maintained until the final session. The speed of running was adjusted according to target HR zone (75-85% maxHR). A warmup period of 10 minutes was allocated prior to the start of each exercise session. | **Key Findings:** In the intervention group, repeated measure analyses showed significant differences between pre, mid (12th week) 24 weeks post lung function, weight, and BMI.  **Conclusion:** Regular, prolonged (>24 weeks) physical activity (i.e. aerobic exercise training) and reaching a normal BMI (BMI <25) can partly improve lung function in overweight and sedentary patients. Whereas physical inactivity and obesity impair these measures. | 85.71 |
| Bai | **Title:** The Longitudinal Impact of NFL PLAY 60 Programming on Youth Aerobic Capacity and BMI  **Year:** 2017  **Country:** USA | **Aims/Purpose:** To systematically evaluate the longitudinal impact of the NFL PLAY 60 programs on fitness and weight status  **Sample Size:** 497  **Participants:** Children from elementary, middle and high school | **Duration:** 4 years  **Physical Activity:** A program, the NFL Play 60 Challenge had the goal of promoting the recommended daily 60 minutes of physical activity for kids in school and at home. Supplemental materials were available, including Student Game Planner for students to track their physical activity minutes; online platform where the enrolled school can compete with other schools across the country  **Healthy Lifestyle Education:** Children had more than 60 subject-based lesson plans and over 100 Physical Activity breaks and homework assignments. **Diet/Nutrition:** Healthy eating promotion plans are equipped with additional resources which can be customized for schools to implement.   **Family-Based:** The programs emphasise the leadership of educators as well as the engagement of parents and communities to collectively create healthy eating and Physical Activity awareness for students through events and activities.   **Environment:** The program provides various resources and includes: ten different healthy eating and Physical Activity Playbooks, up to $4,000 in available grants, and NFL rewards to enrolled schools. It provides a Teacher Guide that includes ideas, tips, and directions about how to implement a 4-week Physical Activity Challenge in school and how to engage students. Teachers in the network received training on the effective use of the FitnessGram program through online classes and webinars, as well as through content provided in monthly eNewsletters. | **Key Findings:** The rate of aerobic capacity achievement had a mean increased from 64.8% to 72.0% from Year 1-4. This was lower in the non-programming schools (64.1% to 66.1%).   **Conclusion:** The findings support the utility of less structured, multilevel school-driven programs which focus more on just the individual but incorporate teachers, school leaders influenced by system-level practices in districts and by local school administrators. | 85.71 |
| *Barbeau | **Title:** Correlates of individual differences in body-composition changes resulting from physical training in obese children  **Year:** 1999  **Country:** USA | **Aims/Purpose:** To study individual differences in body composition changes that result from an intervention program designed to alter body composition  **Sample Size:** 71  **Participants:** Obese children aged 7-11 years | **Duration:** 4 months  **Physical Activity:** Training sessions were offered 5 days/week and the children were asked to attend a minimum of 3 days/week. Each session lasted 40 minutes: 20 minutes were spent exercising on machines (e.g. treadmill, stationary cycle, and trampoline) and the remaining 20 minutes playing games (e.g. basketball, dodge ball, and tag). Each child wore a HR monitor during each session and was encouraged to maintain a HR 150 bpm. | **Key Findings:** Children who spent more time doing VPA had greater decreases in FM (r=20.10, p=0.13) and % BF (r=20.13, p=0.06). A larger total energy intake was related to a larger increase in total mass (r=0.12, p=0.10) and FM (r=0.14, p=0.01)  **Conclusion:** Engaging in physical training and diet may be an important correlate of fat loss, with intensity associated with increased bone mineral density and smaller increases in FFM. | 57.14 |
| Bedard | **Title:** A Quasi-Experimental Study of a Movement and Preliteracy Program for 3- and 4-Year-Old Children  **Year:** 2017  **Country:** UK | **Aims/Purpose:** To evaluate the effectiveness of an evidence-based intervention targeting movement and preliteracy skills  **Sample Size:** 19  **Participants:** Children aged 3-5 years | **Duration:** 10 weeks  **Physical Activity:** The program (60 minutes once/week) consisted of direct functional motor skill instruction, unstructured exploratory free play, and a dialogic storybook reading activity. The first segment of the program focused each week on a specific movement skill through single-step skill acquisition strategies. The free-play segment of the program allowed children the opportunity to self-direct their own activities with access to gross motor and fine motor equipment. Dialogic shared book reading circle occurred each week with 1 book to develop 1 to 2 preliteracy skills. Specific strategies and books were selected from several evidence-based curricula. | **Key Findings:** Significant group effect on gross motor raw scores (p < 0.05), print-concept knowledge (p < 0.05) but not in uppercase letter recognition (p=0.83). Post hoc analyses demonstrate a significant difference in improvement of object manipulation but not stationery and locomotor domains  **Conclusion:** Community-based, parent-oriented movement, preliteracy programs can significantly improve movement and preliteracy skill levels of preschool children with typical development. | 100 |
| Benjamins | **Title:** A culturally appropriate school wellness initiative: results of a 2-year pilot intervention in 2 Jewish schools  **Year:** 2010  **Country:** USA | **Aims/Purpose:** This study describes the process used to create and implement the culturally appropriate wellness program and presents a summary of the results of the process and outcome evaluations  **Sample Size:** 581  **Participants:** Children aged 1-8 years | **Duration:** 2 years  **PE:** The pilot schools selected CATCH for grades 1-3, EatWell and Keep Moving for grades 4-5, and Planet Health for grades 6-8.   **Diet/Nutrition:** The dietitian was available to teach 1 ‘‘sample’’ lesson per class per year. Private consultations with the project dietitian were offered to parents.  **Healthy Lifestyle Education:** A special focus on mental health was added midway through the pilot period. This consultant developed a culturally appropriate curriculum guide and held at least 1 6-week series of sessions for each class in grades 5-8 in the girls’ school. The classes focused on stress management, conflict resolution, body image, and other issues related to mental health and eating disorders.   **Family-Based:** Parents were an integral part of the wellness council. All parents were invited to every council meeting through the school newsletter. In addition, many activities focused on increasing parental knowledge concerning nutrition, physical activity, and eating disorders.   **Environment:** A grant of $10,000 per year was provided to each school to implement or improve PE tailored to their own needs. A school purchased sports equipment and funded activity-based field trips and after-school activities. Schools removed their soda vending machines. All relevant staff received a brief orientation to the health curriculum selected for their grade. Staff were also invited to all educational sessions and activities offered to parents. | **Key Findings:** In the first analysis, students grade 2-4 observed greatest improvements in nutritional knowledge (15.9% to 33.6%, p<.01). The percentage reporting that they were slightly or very unhappy with their body decreased was not statistically significant. There was a significant increase in children getting the recommended amount of daily activity (p < .001). Availability of healthy and unhealthy foods at home and school, extent of parental encouragement to partake in physical activity found no significant changes.  **Conclusion:** The pilot schools who participated in the current initiative demonstrated wiliness to make substantial changes in their curriculum, policies, and practices. The challenge will be to replicate the model in other schools without the significant financial support and professional guidance received in this paper. | 85.71 |
| *Bharath | **Title:** Combined resistance and aerobic exercise training reduces insulin resistance and central adiposity in adolescent girls who are obese: randomized clinical trial  **Year:** 2018  **Country:** USA | **Aims/Purpose:** To determine the benefits of combined resistance and endurance exercise in adolescent girls who are obese and hyperinsulinemic.  **Sample Size:** 40  **Participants:**  Obese females (Tanner 2-3 stage) who are sedentary | **Duration:** 12 weeks  **Physical Activity:** The combined resistance aerobic exercise program was performed for 60 minutes with 5 minutes of warm-up and cool-down/day, 5 times/week for 12 weeks. Combined resistance aerobic exercise consisted of 20 minutes of various resistance band exercises (upper: seated rows, biceps curl, shoulder flexion, elbow flexion, push up; lower: hip flexion, hip extension, calf raise, leg press, squat) and 30 minutes of treadmill walking. The exercises were performed at a moderate intensity with 15–20 repetitions. The warm-up and cool-down consisted of static stretching. Exercise intensity was gradually increased from 40 to 50% HHR in weeks 1–4, to 60–70% HRR in weeks 9–12. Each training session was fully supervised by the researchers. | **Key Findings:** Significantly reduced leptin levels and increased adiponectin (p < 0.05). Improved metabolic status, body weight, BF, and WC, blood glucose, insulin, and IR (p < 0.05).  **Conclusion:** Exercise-trained adolescent girls had a significant improvement in body weight, BMI, plasma glucose, insulin, and adiponectin to leptin ratio; all correlating with overall improvement in health. | 85.71 |
| Bhave | **Title:** Effectiveness of a 5-year school-based intervention program to reduce adiposity and improve fitness and lifestyle in Indian children; the SYM-KEM study  **Year:** 2016  **Country:** India | **Aims/Purpose:** To improve diet and increase activity among an entire 2-year cohort of children in order to reduce adiposity and increase physical fitness  **Sample Size:** 865  **Participants:** Children aged 7-15 years | **Duration:** 5 years  **Physical Activity:** Increased PE sessions to 6/week. Children engaged in daily yoga-based breathing exercises and physical activity sessions (e.g. ‘Bollywood dancing’) during holidays.  **Diet/Nutrition:** Weekly interactive 1-hour sessions about the importance of diet, activity and lifestyle, and the creation of short modules for teachers to use at the start of regular science classes.  **Environment:** The project nutritionist interacted with the school kitchen staff to develop healthier meal options and increase the fruit and vegetable content of lunches. School banned fast-food sellers from outside the school gates, so that fizzy drinks, sweets, ice creams and fried snacks were inaccessible during the school day. | **Key Findings:** Mean BMI Z-score and WC remained similar. Less time was spent watching TV, more time in active play and higher fruit intakes. Few children scored average or above in the fitness tests, but percentages were significantly higher in the intervention group for sit-ups, long-jump and sprint tests.  **Conclusion:** Children in the intervention group were fitter, had better lifestyle indicators, WC than controls. | 71.43 |
| *Bianchini | **Title:** Multidisciplinary therapy reduces risk factors for metabolic syndrome in obese adolescents  **Year:** 2013  **Country:** Brazil | **Aims/Purpose:** To evaluate the effects of a 16-week Multidisciplinary Program of Obesity Treatment on the control of metabolic syndrome and dyslipidemia in obese adolescents  **Sample Size:** 86  **Participants:** Children aged 10-18 years with excess body weight | **Duration:** 16 weeks  **Physical Activity:** 1-hour sessions (3 times/week). Exercises included sit ups, bending arms, squats, and exercises with the medicine ball (60-66 % maxHR) (20 % of the intervention), walking/jogging (54-59/75-84 % maxHR; 30 %) and basketball (82-89 % maxHR; 50 %).   **Diet/Nutrition:** Weekly 1-hour interventions about: the food pyramid, energy density of food, importance of micro and macronutrients to health, the nutritional composition of food.  **Healthy Lifestyle Education:** Weekly 1-hour group psychology (goals, self-observation, discrimination of feelings, body image, self-knowledge, self-motivation, self-control, and interpersonal relationship). Weekly 1-hour lectures about: benefits of exercise.   **Family-Based:** A paediatrician conducted individual appointments with each family and informed them about the importance of healthy habits. | **Key Findings:** WC, TC, systolic and DBP reduced.  43 of the intervention group were obese but this value reduced to 38 adolescents after 16 weeks. 36 adolescents in the control group classified as obese at baseline, increased to 37 following the intervention.  **Conclusion:** The multidisciplinary intervention reduces the number of risk factors for metabolic syndrome in obese adolescents. Future studies should evaluate these same metabolic parameters following longer periods i.e. 1 year, or until adulthood. | 85.71 |
| Bilinska | **Title:** Effectiveness of a school-based intervention to reduce the prevalence of overweight and obesity in children aged 7-11 years from Poznan (Poland)  **Year:** 2017  **Country:** Brazil | **Aims/Purpose:** To evaluate the effects of annual school interventions on the incidence of overweight and obesity among school children  **Sample Size:** 5293  **Participants:** Children aged 7-11 years | **Duration:** 1 year  **Physical Activity:** Group physical activities carried out at school including general development exercises, corrective exercises, dance, classes at a swimming pool under advice by physiotherapist.   **Diet/Nutrition + Healthy Lifestyle Education:** Education about increase active leisure time in daily life, promoting balance and healthy diet and other behavioural factors.  **Environment + Family-Based:** Education directed towards parents and teaching staff. | **Key Findings:** Average BMI increased. Boys BMI increased respectively by 0.40 (control) and 0.41 (intervention) which were almost identical to what was observed in girls.  **Conclusion:** Overweight and obesity among children requires immediate, firm actions and – as demonstrated by this research – will probably be a slow and difficult process. | 71.43 |
| *Branco | **Title:** Effects of the Order of Physical Exercises on Body Composition, Physical Fitness, and Cardiometabolic Risk in Adolescents Participating in an Interdisciplinary Program Focusing on the Treatment of Obesity  **Year:** 2019  **Country:** Brazil | **Aims/Purpose:** To investigate the effects of the order of physical exercises on body composition, physical fitness, and cardiometabolic risk in an interdisciplinary program  **Sample Size:** 44  **Participants:** Overweight or obese females aged 13-17 years | **Duration:** 12 weeks  **Physical Activity:** PE exercises (3 times/week) controlled by timing the activities. During the first mesocycle, the effort: pause ratio was 30" by 30"; the second mesocycle, the effort: pause ratio was 40" by 20" using a 1:1 ratio between the concentric and eccentric phases. Before the session, the adolescents performed light walking and stretching (10-minute warm-up).  **Diet/Nutrition:** Energy intake was set at recommended levels for subjects by following a balanced diet. Nutritional education (2 times/week, 1 hour) aimed to guide the adolescents in nutritional aspects such as: introduction to healthy eating; food manufacturers, regulators, energy, and the food pyramid; frequency and portioning of food; soluble and insoluble fibres; the importance of fibre consumption; a list of food replacements, according to the food groups; pre- and post-physical activity feeding; minimally processed, processed, and ultra-processed foods; amounts of sugar, salt, and fat in foods; how to read and interpret food labels; diet and “light” foods; eating disorders; the dangers of “fad diets”; and ingesting food for health and quality of life using food re-education as an instrument.   **Healthy Lifestyle Education:** Cognitive-behavioural therapy (1 time/week, 1 hour) with topics including self-monitoring, control of anxiety, negative feelings, and health education. The activities also aimed to reduce negative emotions regarding the participants’ relationships with food consumption and strategies used to improve the assessment of body self-image. | **Key Findings:** No significant differences were observed in the level of physical activity, rate of perceived exertion, internal training load, and rate of perceived recovery were identified (p > 0.05). MM (p = 0.02), rate of perceived recovery (p=0.03) , FM (p = 0.008), % BF (p = 0.001), WC (p = 0.002), insulin (p=0.004), IR (p=0.01), TG (p=0.001), TC (p = 0.000), LDL-c (p=0.003), maximal isometric handgrip strength (p = 0.05), maximal isometric trunk strength (p=0.01), maximal isometric strength of the lower limbs (p = 0.0008) and VO2max (p<0.001) had an time effect. Fasting glycemia levels observed no time or interaction effects.  **Conclusion:** Concurrent training within a multi-professional context positively impacted anthropometric variables and rate of perceived recovery. Regardless of the order of concurrent exercise, glycaemic and lipid profiles, physical fitness, maximum isometric strength, cardiopulmonary fitness tests improved. | 85.71 |
| *Brand | **Title:** Effects and prevalence of responders after a multicomponent intervention on cardiometabolic risk factors in children and adolescents with overweight/obesity: Action for health study  **Year:** 2020  **Country:** Brazil | **Aims/Purpose:** To verify the effect of a multicomponent intervention on cardiometabolic risk factors **Sample Size:** 35 **Participants:**  Children aged 7-13 years with overweight/obesity | **Duration:** 12 weeks  **Physical Activity:** Consisted of exercise sessions (twice/week; 1 hour) with warm-up (10 minutes), circuit training (30 minutes), recreation games/sports (15 minutes), resting activities (5 minutes). Training intensity at 65% maxHR.  **Diet/Nutrition + Family-Based:** Dietary counselling with parents (once/month) where goals were set based on frequency and quantity of adequate and inadequate food and water consumption. Parents also participated in exercise sessions | **Key Findings:** There were significant improvements in BF (p=0.04), HOMA-IR (p=0.04), HDL-c (p=0.01), TC (p=0.01) but no significant differences in TG, LDL-c, aspartate aminotransferase, alanine aminotransferase.  **Conclusion:** There is a positive effect of a multicomponent intervention including exercise training, nutritional education and parental support on cardiometabolic risk factors in children with overweight/obesity. | 85.70 |
| Braun | **Title:** Impact of an Elementary School-Based Intervention on Physical Activity Time and Aerobic Capacity, Georgia, 2013-2014  **Year:** 2017  **Country:** USA | **Aims/Purpose:** The objectives of this evaluation were to determine the impact of a 1-year elementary school physical activity intervention on changes in physical activity time  **Sample Size:** 3479  **Participants:** Children in fourth grade | **Duration:** 1 year  **Physical Activity:** Strategies implemented to emphasize MVPA included: classroom exercise DVDs, classroom physical activity integration strategies and activities, pedometer math games, morning announcements, recess games and reminders, and an emphasis on not taking away recess as a punishment, The Build Our Kids’ Success before-school program, exercise sequence creation worksheets, and walking and pedometer games.  **Family-Based:** Children brought take-home educational materials and physical activity resources to share with parents.  **Environment:** Staff members had individualized health training, pedometer challenges, and challenges to catch teachers practicing healthy behaviours. | **Key Findings:** 23 of 39 (59%) schools increased, 10 (26%) decreased, 6 (15%) maintained the mean estimated total teacher-reported time in physical activity. However, on average, the mean estimated total weekly teacher-reported time in physical activity increased by 39 minutes post intervention (21 minutes in recess, 17 minutes in the classroom, and 1 minute in PE). The overall mean student-level BMI was unchanged.  **Conclusion:** Multi-component school-based physical activity improved aerobic capacity particularly in this population. Future studies should apply more Rigorous testing, including larger scale randomised controlled trials, in order to understand how to generalise such findings. | 71.43 |
| *Brownell | **Title:** A school-based behavior modification, nutrition education, and physical activity program for obese children  **Year:** 1982 **Country:** USA | **Aims/Purpose:** To evaluate a combination of behaviour modification and nutrition education administered in schools.  **Sample Size:** 77  **Participants:** Overweight children aged 5-12 years in fifth grade | **Duration:** 10 weeks  **Physical Activity:** Daily exercises for improving aerobic capacity, strength and flexibility. Children played some team games, aerobic activities and stretched.  **Diet/Nutrition:** Nutrition education including constituents of a balanced diet, reasons for obesity, methods of trimming calories from meals, reading food labels, preparing low calorie dishes. A special diet meal was offered to the program participants during the school lunch and consisted of a salad plate with protein and low-calorie salad dressing, bread, fruit, and low-fat milk.  **Healthy Lifestyle Education:** Behaviour modification strategies implemented with methods for increasing daily activity. Provided with a guide including the caloric expenses of various exercises.  **Family-Based:** Parents encouraged to attend 3 meetings held by the nurse's aide. Meetings provided further instruction on behaviour modification. The parents were taught to praise changes in behaviour and weight, to help their children set realistic goals and to avoid condemning their children for periodic transgression.   **Environment:** Created positive social environment in which overweight children received support from parents, the nurse's aide, teachers, the PE instructor, peers, food service personnel and school administrators. Teachers were instructed in methods of encouraging the children to lose weight, teaching the children about nutrition and exercise, and enlisting the aid of parents. | **Key Findings:** At the 1-year follow up, there were no significant differences in prevalence of overweight, and weight loss.  There were correlations between change in weight and change in systolic pressure of 0.3 (p<0.07) at 16 weeks and 0.42 (p<0.04) at 1 year. After 1-year net changes from base line were -1.9/-1.2 mmHg.  **Conclusion:** The nature of parent involvement is important (e.g. the presence or absence). A behavioural program with involvement of mothers can produce and sustain large weight losses for as long as 1 year after treatment. Weight changes in children are not associated with weight loss in their mothers but is associated with improvements in blood pressures. | 85.71 |
| *Brownell | **Title:** Treatment of obese children with and without their mothers: changes in weight and blood pressure  **Year:** 1983 **Country:** USA | **Aims/Purpose:** To test 3 methods of parent involvement in the treatment of obese adolescents   **Sample Size:** 42  **Participants:** Obese children aged 12-16 years | **Duration:** 16 weeks  **Physical Activity:** Received a program with exercise instruction which included aerobic conditioning  **Diet/Nutrition:** Nutrition education included information on basic food groups; maintaining a balanced diet low in sugar, salt and fat; specific methods for buying, preparing, storing and serving food; misconceptions about diet and nutrition; self-monitoring of food intake **Healthy Lifestyle Education:** The group leader and the subjects discussed the week's didactic material and matters such as feelings about being overweight, family difficulties and food preparation. Received a program of behaviour modification; stimulus control and cue elimination; behaviour chains and preplanning; attitude restructuring and cognitive control; using alternatives to over-eating. Increasing physical activity was encouraged by discussions of the caloric expense of exercise; programmed life-style activity; and methods for gradually increasing energy expenditure  **Environment:** Social support methods for enlisting the aid of family and friends  **Family-Based:** Interventions involved mothers who received healthy lifestyle and nutrition education | **Key Findings:** Program children lost more weight than control children. Of the 63 program children, 60 lost weight, compared to 3 of the 12 control children  **Conclusion:** Schools may be ideally suited for a weight control program. The offer many potential sources of social support | 85.71 |
| Burgi | **Title:** Effect of a lifestyle intervention on adiposity and fitness in socially disadvantaged subgroups of preschoolers: a cluster-randomized trial (Ballabeina)  **Year:** 2012 **Country:** Switzerland | **Aims/ Purpose:** To determine whether the intervention was equally effective in the 2 predefined subgroups of high-risk children (migrant and/or low education level parents) regarding its effects on adiposity and fitness  **Sample Size:** 652  **Participants:** Children aged 4-6 years | **Duration:** 1 year  **Physical Activity:** Program consisting of 4x45-minute/week of lessons aimed to increase aerobic fitness and coordination skills; they were designed to be playful and organised into themes. Health promoters gave initially one physical activity lesson/week which was reduced to twice a month after 4 months. The remaining lessons were provided by the regular preschool teacher.  **Diet/Nutrition +Health Lifestyle Education:** There were 22 lessons on healthy nutrition, media use, and sleep. Healthy snacks during recess and healthy treats for anniversaries were promoted.  **Family-Based:** Parents participated in 3 interactive information and discussion evenings about promotion of physical activity, healthy food, limitation of television use and the importance of sufficient sleep. Further support was provided by brochures, funny cards, worksheets, and physical activity exercises that children brought home. Information leaflets were provided in ten different languages and native speakers of the main foreign languages were available to answer questions.  **Environment:** Besides curricular changes, the built environment in and around the preschool class was adapted to promote physical activity including mobile equipment such as climbing walls, hammocks, balls, cords, or stilts were installed or provided in and around classrooms. This aimed to promote children's physical activity during recess and during school time. Teachers participated in 2x 3-hour workshops about the content and the practical aspects of the intervention. They were supported by visits of health promoters and by hands-on training. | **Key Findings:** Children of migrant and non-migrant parents benefitted from the intervention with significant effects on %BF and WC.   Aerobic fitness effects were only significant in non-migrant children. Agility changes were only significant in-migrant children.   Low education level parent children benefitted less compared to their middle/high education level parent counterpart (p≥0.2 for adiposity measures and agility and p=0.06 for aerobic fitness) except for WC (p=0.02).  **Conclusion:** This intervention was beneficial for pre-schoolers of both migrant and non-migrant background with similar effect irrespective of region. Children of low education level parents had a smaller benefit (i.e. had smaller intervention effect sizes) compared to middle/high education level groups, despite lack of significant difference. | 71.43 |
| Burguera | **Title:** ACTYBOSS: activity, behavioral therapy in young subjects--after-school intervention pilot project on obesity prevention  **Year:** 2011 **Country:** Spain | **Aims/Purpose:** To test the feasibility of a school-based intervention, which combines an incentive-driven physical activity program with lifestyle lectures, and its potential beneficial outcome on children’s metabolic parameters  **Sample Size:** 90  **Participants:** Children in first, second and third grade of Obligatory Secondary School of Spain (corresponds to seventh, eighth and ninth grades in the USA) | **Duration:** 6 months  **Physical Activity:** In supervised physical activity sessions, children received points as a reward for the hours they spent exercising. The credit-earned points obtained could be exchanged for sport equipment, trips, gym subscriptions, or leisure and sport events tickets. The children had the opportunity to participate in enjoyable, non-competitive sports and physical activities. The ACTYBOSS exercise toolbox included team sports, racket games as well as dancing and music games. The activity was free, supervised by PE instructors and scheduled 15 minutes after their regular school classes ended. The maximum exposure to the activity offered was 3 hours every day, Monday through Thursday (divided into 2 periods of 90 minutes) and was offered in their school sport facilities. Children received 1 point for each minute they participated in the activity.  **Diet/Nutrition + Healthy Lifestyle Education:** Participants were offered 2 nutritional and 2 behavioural modification workshops during the intervention period. Children received points as a reward for their attendance to lectures. The topics were: How to eat healthy?; Influence of the mass media on what we eat; Impact of physical activity on body and brain development; and Build up our self-esteem.   **Family-Based:** 2 nutritional conferences were offered to the parents/tutors during the 6 months that the intervention lasted. Children received points for each session that their parents/tutors attended to. The topics were: How to cook a healthy meal?; and Benefits of the Mediterranean diet. | **Key Findings:** There were no statistically significant differences in anthropometric variables at baseline compared to 6-months follow-up. None of the biochemical or hormonal parameters showed significant changes.  Beneficial changes in MM (+3.4%), FFM, fasting plasma glucose and insulin levels were observed.  Improvement in all parameters tested were observed including a 0.5s improvement in speed, 3cm increase in flexibility as well as coordination.  **Conclusion:** School interventions based on an incentive-driven physical and nutritional advice, resulted improvements on anthropometrical measurements, and fitness in participants. Future research should help introduce inactive children to the benefits of physical activity. | 57.14 |
| Cadzow | **Title:** School-Based Obesity Intervention Associated with 3 Year Decrease in Student Weight Status in a Low-Income School District  **Year:** 2015 **Country:** USA | **Aims/Purpose:** To presents the results of a longitudinal analyses among those who have at least baseline and endpoint height and weight measurements  **Sample Size:** 2259  **Participants:** Children in third to twelfth grade | **Duration:** 3 years  **Physical Activity:** The program facilitated non-competitive types of activity that includes new equipment. Adventure education was added to the district PE curriculum  **Environment:** The equipment purchased included virtual reality bikes, cardio equipment, spinning bikes and strength training equipment for the high school. Two middle schools were provided with ‘‘sport walls’’. Seven elementary schools and the middle schools were equipped with ‘‘XerDance Pads’’. Traverse climbing walls were installed in all seven elementary schools with a vertical wall and ropes course at the high school. Extensive training was provided to PE staff. Coca-cola vending machines had no soda or caffeinated beverages. | **Key Findings:** The mean BMI percentile among those categorized as obese or overweight at baseline decreased (p<0.001).  **Conclusion:** This study suggests that school-based interventions targeting grades 3–12 may have a significant effect on student weight status. A multifaceted intervention may result in a reduction in BMI percentile in school children. | 100 |
| Camhi | **Title:** The influence of BMI on long-term fitness from PE in adolescent girls  **Year:** 2011  **Country:** USA | **Aims/Purpose:** To determine the longitudinal effects of an 8-month PE class on fitness in a group of urban, mostly African American, adolescent girls  **Sample Size:** 131  **Participants:** Children aged 13-15 years | **Duration:** 8 months  **Physical Activity:** Classes were scheduled 5 days/week for 47 minutes. Girls were given approximately 12 minutes to change their clothes, resulting in 35 minutes of structured class time. Instructional units and daily class activities were designed to appeal to adolescent girls and maximise active time in each class. Small groups and station work were strategies frequently used to increase class activity. Other activity units were walking/jogging, fitness (resistance training, circuit training), and swimming in the fall, and basketball, volleyball, and recreational games in the spring.  **Healthy Lifestyle Education:** Units and lesson plans aimed to increase physical activity in class and promote out-of-class physical activity through behavioural skills training. | **Key Findings:** Mean HR improved from baseline (p=0.008), 10th (p=0.0004), and 11th grade (p=0.0003). Differences between BMI groups were significantly different between BMI groups from baseline to 11th grade (p = 0.008) but not to 9th grade (p=0.24) and 10th grade (p=0.11). Obese girls demonstrated no improvements in fitness.  **Conclusion:** These findings have implications for the inclusion of PE programs for adolescent girls to prevent the decline and improvement of fitness. | 42.86 |
| Canavera | **Title:** Development and pilot testing a social cognitive theory-based intervention to prevent childhood obesity among elementary students in rural Kentucky  **Year:** 2008 **Country:** USA | **Aims/Purpose:** To design and test a social cognitive theory driven intervention for reducing childhood obesity.  **Sample Size:** 318  **Participants:** Children aged 10-12 years in fifth grade | **Duration:** 12 weeks  **Physical Activity:** Introduced the participants to different types of physical activity, the benefits of physical activity, and techniques to monitoring physical activity. **Diet/Nutrition:** Sessions included introduction of the different types of fruits and vegetables, benefits of fruits and vegetables, intake of fruits and vegetables, replacing sweetened beverages with water.  **Healthy Lifestyle Education:** Education on limiting television usage, self-efficacy and -control for limiting television usage. | **Key Findings:** There were significant changes in watching television (p = .002), drinking water (p = .049), and the number of glasses of water consumed (p = .022).  **Conclusion:** The intervention was successful in influencing only 2 constructs, namely expectations for watching television and expectations for drinking water. | 57.14 |
| *Carlone Baldino Garcia | **Title:** Multidisciplinary obesity treatment program improved health-related quality of life and positively correlated with anthropometric and body composition but not with cardiorespiratory fitness parameters in adolescents  **Year:** 2019 **Country:** Brazil | **Aims/Purpose:** The aim of the present study was to investigate the effects of a 16-week multidisciplinary obesity treatment program on health-related quality of life in adolescents with weight excess  **Sample Size:** 124  **Participants:** Overweight children aged 15-18 years | **Duration:** 16 weeks  **Physical Activity:** Physical activity program that consisted of 3x60-minute sessions/week. Exercises intensively performed in a playful environment.  **Diet/Nutrition:** The discussed topics were: the food pyramid; energy density of food; the importance of micro and macronutrients; nutritional composition of food; portion control; strategies for eating out; flexible dietary restraint; preparation of healthy food; feeding frequency.  **Healthy Lifestyle Education:** Lectures discussed the practice of exercise and its benefits. Reflections about the intensity and volume of physical activity sessions were also held during the lectures. Psychological educational sessions (1 hour/session) discussed: setting goals; self-observation of own behaviour and its consequences; identification of feelings and emotional analysis; body image and self-perception; self-knowledge and analysis of thoughts and their consequences; self-motivation and self-control; and discussion on the development of social skills. | **Key Findings:** After 16 weeks all anthropometric variables and CRF improved in the intervention group whilst none in the control.   Significant improvements for the total (p≤0.001), psychosocial (p≤0.001), physical (p<0.005), emotional (p<0.001), and social (p≤0.001) for the health-related quality of life domains, except in the school domain (p=0.303).  **Conclusion:** The program improved HR quality of life in overweight adolescents, showing a significant correlation with improvements in weight, BMI z-score, WC, and BF. A lack of consensus in the literature, suggests further longitudinal studies are required. | 57.14 |
| *Carnier | **Title:** Obese adolescents with eating disorders: analysis of metabolic and inflammatory states  **Year:** 2012 **Country:** Brazil | **Aims/Purpose:** To compare the effects of interdisciplinary therapy on the physical and metabolic profiles of obese adolescents with and without eating disorder symptoms  **Sample Size:** 83  **Participants:** Obese children aged 15-19 years, BMI >95th percentile | **Duration:** 1 year  **Physical Activity:** Personalised aerobic training program (30 minutes) plus resistance training (30 minutes) 3 times/week. The aerobic exercises were performed at the cardiac frequency intensity of the VTI (±4 bpm) on a treadmill. Each of the main muscle groups was worked with resistance training. Adaptation to training occurred for 2 weeks (3 sets x 15–20RM). Every eight weeks, volume and intensity decreased (repetitions from 15–20 to 10–12 and 6–8, 3 sets). Physiotherapy intervention (1 time/week) including postural re-education, isostretching, diaphragmatic breathing, hydrotherapy, balance and stretching.    **Diet/Nutrition:**  Energy intake was set at the levels recommended by the dietary reference intake. Nutritional lessons once/week (food pyramid, recordatory inquiry, weight loss diets, diet and light concepts, fat and cholesterol and eating disorders) and were seen individually twice a month.  **Healthy Lifestyle Education:** All adolescents had weekly psychological orientation group sessions based on the psychodynamic approach with 1 trained psychologist, where they discussed family problems, body image, low self-esteem and eating disorders such as bulimia, anorexia nervosa, and binge eating, their signals, symptoms and consequences for health.   **Family-Based:** All obese adolescents visited the endocrinologist with their parents once each month. The doctor monitored and evaluated all clinical exams of adolescents and treated health problems during therapy. The team discussed with the patients and their parents some possible changes in lifestyle to promote their health. | **Key Findings:** Both groups demonstrated improvements in body mass, BMI, % BF, lean mass, visceral fat, subcutaneous fat, VLDL-c, LDL-c, IR, TC, adiponectin and leptin concentrations after short- and long-term therapy.  **Conclusion:**  Long-term interdisciplinary therapy was effective in reducing the chances of developing several co-morbidities. Little studies have explored obesity and improving metabolic profile in adolescents with eating disorders. | 71.43 |
| *Carrel | **Title:** Improvement of fitness, body composition, and insulin sensitivity in overweight children in a school-based exercise program: a randomized, controlled study  **Year:** 2005 **Country:** USA | **Aims/Purpose:** To investigate whether a school-based fitness program makes a difference in fitness, fatness, and insulin sensitivity  **Sample Size:** 50  **Participants:** Children BMI >95th percentile | **Duration:** 9 months  **Physical Activity:** The frequency of fitness-oriented and standard PE classes was 5 times every 2 weeks for a 45-minute class period. The curriculum was personalized to match the student’s skill levels and encourage student participation. Competitive games were de-emphasized, and lifestyle focused activities (walking, cycling, and snowshoeing) were encouraged. A consistent warm-up plan brought student into movement participation as quickly as possible soon after they entered the gym. Typical movement time was 42 minutes of a 45-minute class period. Skills were taught with the class broken down into groups of 2 for promoting more movement and less time watching.   **Diet/Nutrition:** Students also received a small nutrition education component. This consisted of educational handouts to participants to develop healthier eating habits. The nutrition portion focuses on the Food Guide Pyramid, recommended servings of food, appropriate portion sizes, healthier food choices, and the benefits of those choices. | **Key Findings:** End-of-treatment of mean %BF measures of 32.6%±6.4% and 34.5%±5.8%, respectively. BMI was 33±10 and 30±5 post intervention in the treatment and control respectively (p=.10).   Significant improvements in VO2max (p<.001), fasting insulin levels (p=.02), 1/insulin ratio (p=.01), and glucose-insulin ratio (p=.02) compared to baseline.  **Conclusion:** School curricula may be an effective vehicle for increasing physical activity and improving cardiovascular health for children, and further study is warranted. | 71.43 |
| *Carvalho | **Title:** Peak oxygen uptake responses to training in obese adolescents: a multilevel allometric framework to partition the influence of body size and maturity status  **Year:** 2013 **Country:** Brazil | **Aims/Purpose:** To model the changes in peak VO2-body size relations in obese boys exposed to a 12-week endurance exercise-based intervention   **Sample Size:** 30  **Participants:** Obese boys aged between 10-16 years, BMI >95th percentile | **Duration:** 3 months  **Physical Activity:** All participants were required to attend exercise sessions from 2 to 3 times per week for 3 months or 12 weeks (200-300 minutes/week). Exercise consisted of 45 minutes indoor cycling, 45 minutes outdoor walking/running, and 20 minutes stretching. During the first four weeks, intensity was set as 35–55% of HHR and was increased to 55–75% during the final eight weeks.   **Diet/Nutrition:** The diet emphasized the consumption of abundant vegetables, fresh fruit, regular consumption of dairy products (principally cheese and yogurt), fish and poultry consumed in low to moderate amounts, and a reduced intake of red meat. This diet has been specifically devised for children by our nutritionist. Total fat in this diet is from 25% to 35% of the total caloric intake. A nutritionist delivered education on daily diet.  **Healthy Lifestyle Education:** All participants received an orientation encouraging them to maintain an active lifestyle during and after the program. The adolescents participated in educational meetings once-per month for 60 minutes. The meeting were taught by physical educators and focused on physical activities.  **Family-Based:** Families were provided with additional nutritional instruction, including interpretation of food labels and shopping, and were taught stimulus control to reduce access to high-calorie foods and increase access to healthy lower-calorie foods. | **Key Findings:** Significant changes in body size and FFM, BMI, BMI z-score, WC, and FM with positive Changes in peak oxygen consumption suggested a positive effect.  **Conclusion:** Obese boys presented a consistent improvement in peak oxygen consumption, where changes in the peak oxygen consumption- body size relationship were evident and at least partially influenced by chronological age. | 100 |
| Chehab | **Title:** "Energy Up": a novel approach to the weight management of inner-city teens  **Year:** 2007 **Country:** USA | **Aims/Purpose:** To describes the experience of a school-based, nutrition and fitness program that addresses obesity within the framework of food addiction  **Sample Size:** 46  **Participants:** Children from an all-girl, parochial high school | **Duration:** 9 months  **Physical Activity:** Group aerobic activity (1 hour/week) and homework for daily individual exercise.  **Diet/Nutrition:** Overeating was explained to be the result of food addiction that can be prompted by negative self-views and behaviours, and triggered by certain foods (i.e. flour, sugar, and salt).   **Healthy Lifestyle Education:** Promoted knowledge, regular exercise, positive self-views, and avoidance of “trigger foods”. Weight is not the focus of “Energy Up”; the program avoids using stigmatizing language, such as “obesity”. | **Key Findings:** 50% of all participants lost weight. For obese and overweight girls weight loss correlated with extent of program participation (r=0.06, p < .01).  **Conclusion:** Student involvement in program planning increases likelihood of program success. | 85.71 |
| Chilton | **Title:** Effect of the Total Girl Wellness Program on Wellness Behaviors in Adolescent Females (Chapter 4)  **Year:** 2012 **Country:** USA | **Aims/Purpose:** To explore the effect of a comprehensive wellness intervention, titled Total Girl Wellness Program, on overall wellness, physical fitness, and self-efficacy for health promoting behaviours   **Sample Size:** 2042  **Participants:** Children from 14-19 years | **Duration:** 8 weeks  **Physical Activity:** The program consists of eight 45-minute long, interactive education modules to be presented during class time.   **Healthy Lifestyle Education:** Modules cover wellness (1 session), obesity prevention (2 sessions), relationships (2 sessions), and risky behaviours (3 sessions). Participants were provided with supplemental material for each session. | **Key Findings:** Total wellness score was significant (p =.039). Post-test fitness scores were not significantly different.   Mean self-efficacy scores for intervention demonstrated significant decrease in nutrition (p <.01), exercise (p <.01), total self-efficacy (p <.05).  **Conclusion:** Female adolescence is a critical point in development where health behaviours implemented have life-long implications. Programs such as the Total Girl Wellness Program need further research. | 57.14 |
| Chomitz | **Title:** Healthy Living Cambridge Kids: a community-based participatory effort to promote healthy weight and fitness  **Year:** 2010 **Country:** USA | **Aims/Purpose:** To describe a community-initiated, implemented, and evaluated healthy weight intervention, Healthy Living Cambridge Kids  **Sample Size:** 1859  **Participants:** Children in kindergarten to eighth grade | **Duration:** 3 years  **Physical activity:** “New PE” expanded to all K-8 schools, including non-traditional activities (i.e., yoga, ballroom dance, “Project Adventure”); before- and after-school programming expanded. Outreach events: ; fitness expo (24 exhibitors)  **Diet/Nutrition:** School nutritionist and consultant chef introduced 15 new recipes emphasizing fresh, local ingredients; 110 “taste-tests” in 12 schools, including staff coaching to prepare recipe; 4 group technique trainings; farm-to-school activities; 45 healthy cooking classes; 74 nutrition education sessions.  **Family-Based:** “Fit Together” family nights. Nutrition education was offered to families of obese children   **Environment:** Quarterly professional development for teachers; training for 20 after-school organizations; Citywide policies: “5-2-1”guidelines; local food preference policy; establishment of youth sports commission (13 members); Healthy Living Cambridge poster campaign; mini-grants to 15 community-based organizations to promote 5-2-1; community fitness program; >4,000 physical activity directories distributed annually; school wellness policy; Food Service Advisory Board meetings; nutrition and vending machine guidelines; food purchasing system established with local farmer; a health and fitness progress report: 4,000 K-8 reports distributed district-wide annually via mail. | **Key Findings:** A significant decrease in mean unadjusted BMI z-score (−0.04, p ≤ 0.001) was observed for the overall sample. The prevalence of “healthy weight” increased significantly by 2.4% (p < 0.05). The prevalence of obesity decreased significantly 2.2% (p< 0.05).  Fitness test scores improved significantly for all children, with mean number of fitness tests passed (3.7 (1.32 s.d.) to 3.9 (1.27 s.d.)).  **Conclusion:** To address obesity, it is important to incorporate local priorities, enhance community capacity, build constituencies of support facilitated sustainable and positive changes in policies, system changes, and program elements (i.e., “New PE”, school gardens, cafeteria taste-tests, and food service staff training). Future studies should focus on multilevel health as such. | 71.43 |
| Cluss | **Title:** School-Based Health Promotion Initiative Increases Children's Physical Activity  **Year:** 2016 **Country:** USA | **Aims/Purpose:** The purpose of this study was to evaluate the impact of the program on schoolchildren’s physical activity during the school day over a 10-year period  **Sample Size:** 2897  **Participants:** Children enrolled in an elementary school | **Duration:** 10 years  **Physical Activity:** School-based wellness facilitators designed activities geared to engaging children of all physical capacities and motivation. These activities included: morning announcement exercises (children and teachers doing jumping jacks, hopping up and down, jogging in place, doing lunges, knee bends for 3 minutes every day); morning walk program (students walk for 20 minutes in the gymnasium upon entrance into the building); hoops/jumping for heart; pumpkin run, turkey trot, joy joy jog, and shamrock shuffle (monthly full-school walks that occur during the school day for approximately 30 to 40 minutes); easter eggs-ercises hunt (plastic eggs are stuffed with exercise instructions - the activity typically lasts 30–35 minutes); the great apple crunch (a day celebrating apples as healthy snacks with station activities); brain breaks (give students time to get out of their chairs and move during class time); StoryWalk fitness trails (encourages both physical activity and reading); fitness as a reward (teachers are encouraged to offer organised fitness activities as rewards for classroom behaviour).   **Diet/Nutrition + Healthy Lifestyle Education:** Nutrition information and fitness activities, divided into 10 lessons includes include teaching children about “Whoa, Slow and Go” foods, healthy snacks, nutritious “happy” breakfasts, and how to help in the kitchen with worksheets and activity station signs to support session delivery.  **Environment + Family-Based:** Local marketing such as billboards promoting healthy behaviours, assisting schools in giving students and parents opportunities to learn about and engage in healthy behaviours, working with grocery stores to provide healthy recipes of the week for the community, and hosting health-focused community events. | **Key Findings:** At baseline the total number of KidMinutes was 379,808 for the study month. After 1 year there was a 43% increase for the following 2 years. In 2010, minutes were 74% higher than in 2009. From 2010 to 2015, KidMinutes remained high, with a 293% increase in physically active minutes.  The average number of monthly KidMinutes per student per school showed a statistically increasing trend (p=.001). Average KidMinutes increased by 22.4 minutes per year (95% confidence interval, 12.7– 32.1). BMI observations of overweight and obesity in the district varied from 2005-2013, ranging of 36-39%, but demonstrating a slight decline in 2014,2015.  **Conclusion:** With supports in place (i.e. providing opportunity to be more active, purchase of equipment), schools can be successful in increasing physical activity levels. Future studies should explore similar interventions as they can be implemented easily within school. | 71.43 |
| *Cohen | **Title:** Changes in eating behavior and plasma leptin in children with obesity participating in a family-centered lifestyle intervention  **Year:** 2018 **Country:** Canada | **Aims/Purpose:** To examine changes in eating behaviors and plasma leptin concentrations in overweight and obese children participating in a 1-year family-centered lifestyle intervention  **Sample Size:** 73  **Participants:** Overweight children aged 6-8 years | **Duration:** 1 year  **Diet/Nutrition:** Seven sessions with dietitian: once/month for 6 months and 1 time at the end of their 8th month for a follow-up session. Participants were encouraged to meet the current Canadian recommendations for diet.  **Healthy Lifestyle Education:** Canadian children are encouraged to engage in 60 minutes of MVPA/day, with weight-bearing types of activity being performed 3 times/week.  **Family-Based:** Families participated in a basic teaching of Canada’s Food Guide and physical activity guidelines. | **Key Findings:** Reductions in FM and %BF at 6 and 12-months. Child Eating Behavior Questionnaire scores were lower for Food Responsiveness (p=0.05), Emotional Overeating (p=0.01), Desire to Drink (p=0.005), Food Approach Scores (p=0.0002). Mean leptin concentrations were significantly lower. Physical activity and sedentary behaviours were not significant.  **Conclusion:** A family-centred lifestyle approach favourably changed eating behaviours, reduced leptin concentrations. | 71.43 |
| *Cohen | **Title:** Bone Health is Maintained, While Fat Mass is Reduced in Pre-pubertal Children with Obesity Participating in a 1-Year Family-Centered Lifestyle Intervention  **Year:** 2017 **Country:** Canada | **Aims/Purpose:** To test whether increasing milk and milk products and weight-bearing types of physical activities resulted in favourable changes in bone outcomes in children with obesity   **Sample Size:** 73  **Participants:** Overweight children aged 6-8 years | **Duration:** 1 year  **Diet/Nutrition:** Seven sessions with dietitian: once/month for 6 months and 1 time at the end of their 8th month for a follow-up session. Participants were encouraged to meet the current Canadian recommendations for diet.  **Healthy Lifestyle Education:** Canadian children are encouraged to engage in 60 minutes of MVPA/day, with weight-bearing types of activity being performed 3 times/week.  **Family-Based:** Families participated in a basic teaching of Canada’s Food Guide and physical activity guidelines. | **Key Findings:** BMI Z-scores (p<0.001), intakes of dietary fat (p=0.08) and meat (p=0.07) significantly decreased. FM significantly increased (p=0.02). Change in osteocalcin, bone specific alkaline phosphatase, C-terminal telopeptide of type 1 collagen, PTH, calcium and time and frequency in participation of weight-bearing activities were not significant.  **Conclusion:** A family-centred lifestyle intervention was successful at reducing adiposity while maintaining bone outcomes. | 57.14 |
| *Cohen | **Title:** Long-term effects of a lifestyle modification exercise program on the fitness of sedentary, obese children  **Year:** 1991 **Country:** USA | **Aims/Purpose:** To increase the awareness and appreciation of the importance of health and fitness in young children and to establish these children a pattern of regular aerobic physical activity and a balanced diet  **Sample Size:** 12  **Participants:** Obese children aged 8-12 years | **Duration:** 1 month  **Physical Activity + Family Based:** Met 3 days/week for 2 hours/session, parents required to attend >1 session/week. The physical activity began with five minutes of slow stretching, followed by a 15-minute walk/run and 1 minute of modified sit-ups. Also included were swimming, gymnastics, rope jumping, rhythmic activities and several game-type activities. Children were given an individualised exercise program to do at home. This program included: five minutes of stretching, 15-30 minutes of aerobic activity, 1-minute modified sit-up.  **Diet/Nutrition:** Nutrition education was deemed critical to encourage children to understand how to make appropriate food choices for themselves. No counting of calories was utilised. | **Key Findings:** There was a significant increase (p<0.01) in total sit-ups, total distance run (p<0.05). TC did not have a significant change. Daily food intake records indicated positive changes including adding 6 oz. glass of milk to each meal, reduced snacks and desserts to 1 /day but little increase in intake of vegetables and no reduction in number of fast food meals.  **Conclusion:** A program as such will encourage children to be aware of the importance of exercise. | 42.86 |
| Coknaz | **Title:** A digital movement in the world of inactive children: favourable outcomes of playing active video games in a pilot randomized trial  **Year:** 2019 **Country:** Turkey | **Aims/Purpose:** To determine the quantitative effect of active video games on physical fitness parameters, enjoyment levels, attitude changes  **Sample Size:** 106  **Participants:** Children aged 8-14 years who have not played active video games at home | **Duration:** 12 weeks  **Physical Activity:** Children played Nintendo Wii® active video games from sports (boxing, tennis, golf, baseball, and bowling), balance (ski slalom, heading ball, balance bubble, ski jumping and penguin playing), aerobics (rhythm boxing, hula-hoop, cycling, step, and run), resort (jet-skiing, water skiing, table tennis, basketball, swordplay, archery, canoeing and frisbee) and training (rhythm kung fu, snowball, turning ball, Segway circuit, perfect 10, skateboard, major, obstacle course and bicycle) categories for 50–60 minutes, 3 days a week, for 12 weeks in laboratory environment supervised by 3 experienced personnel. | **Key Findings:** BMI and BMI z score mean values decreased significantly. The most significant reduction in reaction time was observed for visual reaction time in non-dominant hands (p=0.000). Increases in all subscales of physical self-perception including physical self-worth (p=0.007), global self-worth (p=0.003) were significant. No significant differences for physical activity enjoyment.  **Conclusion:** Active video games might be used as an adjunctive tool in overcoming inactivity and obesity-related comorbidities. | 71.43 |
| Cordova | **Title:** Physical activity and cardiovascular risk factors in Spanish children aged 11-13 years  **Year:** 2012 **Country:** Spain | **Aims/Purpose:** The aim of this study was to determine whether a daily physical activity intervention at school would be associated with lower overweight and risk factors related to obesity in children  **Sample Size:** 137  **Participants:** Children aged 11-13 years | **Duration:** 3 months  **Physical Activity:** The children were divided into 3 groups based on their parents’ decision to enrol them in additional school sports programs over time: sedentary group (control), completing 2 hours per week of PE at school; active group, 2 hours per week of PE at school plus 3 hours extra physical activity, and sports group, adding 5 hours per week of physical activity to the 2 hours of weekly activity at school. | **Key Findings:** Weight, BMI, WC, skinfolds decreased significantly. FFM index indicated no significance. TC (HDL-c, LDL-c), atherogenic index, and glucose concentration show no significance. VO2max and maxHR increased. No significant differences in blood pressure.  **Conclusion:** Results report an association between physical activity and an improvement in CVD factors – WC, FMI, SBP and IR, thus indicating the importance of exercise in children to prevent overweight and obesity. | 85.71 |
| *Crouter | **Title:** Effects of an afterschool community center physical activity program on fitness and body composition in obese youth  **Year:** 2017 **Country:** USA | **Aims/Purpose:** To describe program delivery and impact in a case series of 30 obese girls and boys  **Sample Size:** 30  **Participants:** Children aged 7-18 years, BMI >95^th^ percentile | **Duration:** 24 weeks  **Physical Activity:** The program (90-minutes/session, Monday-Thursday) included 60–75 minutes of physical activity consisting of a warmup (15 minutes), aerobic training (15 minutes), strength training (20 minutes), and active games (15–25 minutes). Aerobic training included exercise with treadmills, elliptical machines, relay races and circuits. Sessions started with 5 minutes (65-75% of maxHR), followed by 10 minutes (75-85% maxHR.). Strength training with Cybex machines (8–12 repetitions, 2–4 sets, 70% 1RM), resistance bands, free weights, and body weight exercises. Active games included plyometrics, strength-endurance circuit training, tag, soccer, football, Exergaming.  **Diet/Nutrition + Healthy Lifestyle Education:** Increase self-efficacy for habitual exercise and understanding of how physical activity and healthy eating contribute to a healthy lifestyle. | **Key Findings:** Height and FFM increased. Significant decrease on body mass, WC, BMI, BMI z-score, and % BF (all, p< 0.014). Leg press and chest press 1RM improved (p < 0.031)  **Conclusion:** Obesity-sensitive fitness targeted at community youth can improve body composition, aerobic fitness, and strength. | 85.71 |
| Crova | **Title:** Cognitively challenging physical activity benefits executive function in overweight children  **Year:** 2014 **Country:** Italy | **Aims/Purpose:** To test the association between children’s physical fitness and 2 core executive functions  **Sample Size:** 70  **Participants:** Children aged 8-10 years | **Duration:** 21 weeks  **Physical Activity:** 1 curricular PE class/week plus 2 additional hours of skill-based and tennis-specific training. First training hour aimed at developing fundamental motor skills and perceptual–motor adaptation abilities in situational games preceded by warm-up and followed by static stretching; a second hour dedicated to object control skills in tennis and specifically to learning main tennis shoots and playing individual or team point games. | **Key Findings:** VO2max improved (p = 0.015) with no change in BMI (p = 0.193).  **Conclusion:** Physical activity is important in aiding aspects of mental functioning important to cognitive development. | 57.14 |
| *Cvetkovic | **Title:** Exercise training in overweight and obese children: Recreational football and high-intensity interval training provide similar benefits to physical fitness  **Year:** 2018 **Country:** Australia | **Aims/Purpose:** To compare the effects of recreational football on body composition, muscular fitness, and CRF measures in overweight and obese children  **Sample Size:** 42  **Participants:** Overweight or obese male children aged 11-13 years | **Duration:** 12 weeks  **Physical Activity:** Regular PE classes (2 times/week) plus training intervention. Football training was performed 3 times/week (Monday, Wednesday, and Friday). Each training session lasted 60 minutes (10-minute low-intensity warm-up, 4x8-minute periods of play, 2 minutes of passive rest, 10-minute cool-down.) During weeks 0-4, weeks 5-8, and weeks 9-12, the training volume (number of repetitions and duration) was progressively increased. | **Key Findings:** A significant decrease in body mass (p=.034), BMI (p=.017). Non-significant changes (p>.05) for lean body mass, MM, and FM. Lower-body power increased (p=.107). Significant (p<0.05) improvements in total distance covered in the Yo-Yo Endurance test (p=.025).  No significant change in SBP and DBP.  **Conclusion:** Recreational football showed improvements in multiple measures of muscular and CRF after 12 weeks of training in overweight and obese male children. | 85.71 |
| *Da Silva | **Title:** Impact of readiness to change behavior on the effects of a multidisciplinary intervention in obese Brazilian children and adolescents  **Year:** 2015 **Country:** Brazil | **Aims/Purpose:** To investigate the impact of stage of readiness for behavioural change on the effects of a multidisciplinary program  **Sample Size:** 113  **Participants:** Overweight/obese children aged 10-18 years | **Duration:** 16 weeks  **Physical Activity + Diet/Nutrition + Healthy Lifestyle Education:** Interventions occur 3 times a week (Mondays, Wednesdays and Fridays) in the afternoon, lasting 2 hours per session. In the first hour, children and adolescents participate in group educational activities with the PE teachers (Monday), nutrition (Wednesday) and psychology (Friday) to increase knowledge and awareness about the respective topics and how they relate to healthy lifestyle and body weight regulation. In the second hour, the intervention is performed PE through active engagement in physical activity. | **Key Findings:** Percentage changes in BMI (p=0.045), waist-hip ratio, DBP, VO2max, abdominal strength/resistance (p<0.001) significantly improved.  **Conclusion:** Children and adolescents from the later stage observed positive outcomes for most anthropometric parameters (e.g., WHR), DBP, CRF. Future studies follow control teens to ensure that the SRBC influenced the observed changes. | 100 |
| *Da Silveira Campos | **Title:** Homeostasis Model Assessment-Adiponectin: the role of different types of physical exercise in obese adolescents  **Year:** 2017 **Country:** Brazil | **Aims/Purpose:** To investigate the effects of different kinds of exercise in the sensitive index predictor of IR  **Sample Size:** 148  **Participants:** Obese children aged 14-18 years | **Duration:** 1 year  **Physical Activity:** Aerobic only group (60 minutes/session, 3-times/week) performed running on a treadmill. Aerobic and resistant training group (3 times/week) consisted of 30 minutes of bicycle or treadmill; and 30 minutes of strength training of major muscle groups. After a general warm up (10 minutes), individuals performed 15 repetitions with progressively heavier weights until maximal 15 repetitions.  **Diet/Nutrition:** Nutritional therapy, food consumption was set at recommended levels of dietary intake for individuals with low levels of physical activity, based on age, gender and a balanced diet. Discussed topics (once/week) related to food consumption. Received individual consultations during the intervention program.  **Healthy Lifestyle Education:** Psychological group sessions (once/week) on body image, low self-esteem, family problems, eating disorders. Individualised psychological therapy was recommended when necessary. | **Key Findings:** *Aerobic only*- A significant reduction in BMI (p<0.001), BF mass (p<0.0001), %FM (p<0.001), visceral (p<0.001) and subcutaneous fat (p<0.001) with a significant increase in lean tissue % (p<0.001)  *Aerobic and resistance training-* Demonstrated a significant reduction in BMI (p<0.001), body mass (p<0.001), %FM (p<0.001), visceral (p<0.001) and subcutaneous fat (p<0.001) with a significant increase in lean tissue % (p<0.001).  **Conclusion:** Aerobic plus resistance training associated with other therapies was more effective in reduction of fat mass, lean tissue, subcutaneous fat. | 85.71 |
| Dauenhauer | **Title:** Effects of a 3-Tiered Intervention Model on Physical Activity and Fitness Levels of Elementary School Children  **Year:** 2016 **Country:** USA | **Aims/Purpose:** To explore the effectiveness of the response to intervention model in promoting physical activity and fitness among elementary school children  **Sample Size:** 99  **Participants:** Children aged 7-10 years in grades 2-5 | **Physical Activity + Diet/Nutrition + Healthy Lifestyle Education + Family-Based + Environment:**  Tier 1 consisted of 150 minutes per week of PE and school-wide efforts to reinforce healthy behaviours. Students were taught by a certified PE specialist 3 days per week with 1x30-minute lesson focusing on health content and 2x60-minute lessons devoted solely to PE. In addition to quality PE, the tier 1 intervention also consisted of environmental strategies designed to reinforce healthy messages. A school health advisory council met once a month to review pressing issues related to the health of students and to implement monthly activities.  Tier 2 consisted of weekly meetings with the PE specialist in small groups of six or less students. Sessions were held in the morning for 30 minutes, 1 day per week during a scheduled intervention time. Students were taught the basics of goal setting and were closely supervised as they monitored their own physical activity and dietary behaviours. Frequent verbal reinforcement and small incentives were provided to sustain motivation and keep the sessions positive and enjoyable. Each session included 15–20 minutes of instruction with the remainder of the time spent in physical activity based on student interests.  Tier 3 consisted of an after-school program designed for students and parents together. Lead by a paediatrician with an interdisciplinary team of dietitians, psychologists, and physical activity specialists, children and parents attended sessions after school once a week for 3 h. The sessions were designed to provide parents and children with the knowledge and skills for healthy living. Sessions included making healthy snacks and meals together, participating in physical activities, and sensitively addressing some of the psychological factors associated with obesity. | **Key Findings:** Physical activity, steps, VO2max increased, whereas BMI z-scores declined slightly.  Results of the paired samples t tests revealed significant increases in physical activity steps in tier 2, with significant increases in VO2max in both tier 1,2. Changes in BMI z-scores were non-significant in all 3 tiers;  **Conclusion:** A 3-tiered approach can have some positive effects on the physical activity and fitness levels of elementary school children. Educators, administrators, and school health professionals need to be aware of the model, identify students in need of additional support, and take the appropriate steps to ensure that this is received. | 85.71 |
| *Davis | **Title:** Effects of aerobic exercise on overweight children's cognitive functioning: A randomized controlled trial  **Year:** 2007 **Country:** USA | **Aims/Purpose:** To determine if children assigned to a systematic aerobic exercise training program would have greater improvements on a standardized test of cognitive function than children in a non-exercise control condition  **Sample Size:** 94  **Participants:** Overweight children aged 7-11 years, BMI >85th percentile | **Duration:** 2 years  **Physical Activity:** Children assigned to exercise treatments attended programs, which met 5 days per week for 15 weeks. The exercise intervention focused on aerobic fitness. The emphasis was on intensity, enjoyment and safety, not competition nor the enhancement of skills; therefore, activities were selected based on ease of comprehension, fun, and ability to elicit a HR greater than 150 bpm. Points were awarded for maintaining an average HR >150 bpm. The 5-minute daily warm-up included moderate cardiovascular activity (brisk walking, up to 10 jumping jacks) and static and dynamic stretching (toe touches, lunges). Sessions ended with a water break, light cool-down cardiovascular activity (slow walking), and static stretching. | **Key Finding:** A statistically significant effect was seen in the Cognitive Assessment System Planning group where the control group had a significantly lower post-test score than the high-dose exercise group, t(88) = -2.55, p = .01. The low-dose group had a lower post-test score than the high-dose group, t(88) = -1.98, p = .05. The control and low-dose groups did not differ, t(88) = -0.6, p = .52.   No significant group differences on Attention, Simultaneous, or Successive scales, or on BMI z-score or physical activity. Significant improvements in treadmill time were observed in the exercise groups than control t(87) = -2.8, p = .007.  **Conclusion:** This study suggests that devoting school time to sedentary classwork during and after school can be helpful in providing a healthy dose of VPA. | 57.14 |
| *De Miguel-Etayo | **Title:** Body composition changes during a multidisciplinary treatment program in overweight adolescents: EVASYON study  **Year:** 2015 **Country:** Spain | **Aims/Purpose:** To assess the body composition changes in overweight and obese adolescents using different body composition methods after intervention  **Sample Size:** 112  **Participants:** Overweight or obese children aged 13-16 years | **Duration:** 13 months  **Physical Activity:** This program increased physical activity while reducing sedentary behaviour. Aimed to achieve >60 minutes of MVPA 3 days/week, increasing to 5 days/week.   **Diet/Nutrition + Healthy Lifestyle Education:** The intensive nutritional therapy had moderate calorie restriction (10-40%) over 3 weeks; and six weeks fixed full-day meal plans. Psycho-educational workshops focused on eating and physical activity.   **Family-Based:** Individual and family-based psychological support was provided to encourage achievable long-term lifestyle goals. | **Key Findings:** Significantly improved weight loss (p < 0.05), BMI (p < 0.05), lean mass (p < 0.05), FMI (p < 0.05), waist-hip ratio (p < 0.05), waist-height ratio (p < 0.05).  **Conclusion:** A multidisciplinary approach to lifestyle intervention had a favourable impact on adolescent BMI, FMI and waist-height ratio over at least the 13-months. | 71.43 |
| De Vries | **Title:** An activity stimulation program during a child's first year reduces some indicators of adiposity at the age of 2-and-a-half  **Year:** 2015  **Country:** Netherlands | **Aims/Purpose:** To evaluate the effect of an activity stimulating program during a child’s first year on growth, body composition, motor activity and motor development at the age of 2-and-a-half  **Sample Size:** 13  **Participants:** Children born from April 2006-July 2007 | **Duration:** 1 year  **Physical Activity + Family-Based:**  Stimulated an active lifestyle and motor development. Parents were advised to spend 1 hour/day to play with their infant with the infant in the prone position. Bright and colourful toys were used to stimulate activity. After this stage, stimulating the practice of the normal milestones of motor development were promoted. The focus at 2 weeks was to engage symmetric handling. The focus at 2 months was to encourage variation in the infant’s position, and the focus at 4 months was to expand on this. At 8 months, the recommendations were to encourage the infant to crawl. Then at 11 months, parents were instructed to encourage their infant to walk without support.   **Diet/Nutrition:** A youth physician and a specialised nurse see the children. The nurse’s role includes teaching parents about food habits and daily care of their infant. | **Key Findings:** Sum of skinfolds was significantly lower (p < 0.05), with improvements in % BF (p = 0.10). No differences in weight, height, waist and hip circumferences between groups.  **Conclusion:** An activity stimulation program in a child’s first year resulted in lower skinfolds. In girls, the intervention was associated with a lower weight, smaller waist, hip circumferences, lower skinfolds. Further research is needed to determine whether these results are sustained and prevent obesity at an older age. | 71.43 |
| *Deldin | **Title:** Influence of Sex on the Changes in Regional Fat and Skeletal Muscle Mass in Response to Exercise Training in Adolescents with Obesity  **Year:** 2019 **Country:** USA | **Aims/Purpose:** To examine changes in total fat, total and regional subcutaneous adipose tissue, visceral fat, intramuscular fat and total and regional skeletal muscle distribution in response to aerobic or resistance exercise  **Sample Size:** 55  **Participants:** Obese children | **Duration:** 3 months  **Physical Activity:** Aerobic exercise group used either treadmills, ellipticals or stationary bikes at 50-75% of VO2 peak, 3 times/week (60-minute session). Resistance exercise group used weight machines and performed a series of 10 whole body resistance exercises (1-2 sets, 8-12 repetitions), 3 times/week (60 minutes per session).  **Diet/Nutrition:** All participants were asked to follow a weight maintenance diet (55-60% carbohydrate, 15-20% protein, 20-25% fat) during the intervention period to be able to assess that any changes in body composition are consequent to the effects of regular exercise alone and not from caloric restriction. | **Key Findings:** VO2max was greater (p<0.05) in boys with both exercise modalities. Increase in muscular strength index, greater reduction in abdominal subcutaneous adipose tissue (p=0.027) was significantly greater in boys with greater increases in total, upper body and abdominal skeletal muscle.  **Conclusion:** Both aerobic exercise and resistance exercise without caloric restriction are beneficial in reducing total and regional fat whilst increasing skeletal MM. | 100 |
| *Delgado-Floody | **Title:** Effects of 28 weeks of high-intensity interval training during PE classes on cardiometabolic risk factors in Chilean schoolchildren: a pilot trial  **Year:** 2018 **Country:** Chile | **Aims/Purpose:** To determine the effects of 28 weeks during PE classes on the weight status, cardiorespiratory capacity, and blood pressure  **Sample Size:** 197  **Participants:** Overweight and obese children aged 6-11 years | **Duration:** 28 weeks  **Physical Activity:** All the activities took place during PE class time, with a total of 56 sessions of 60 minutes each, twice per week. exercises wherein 4–6-minute repetitions were performed, followed by 1–2 minutes of recovery (four to five sets). The circuit (running, jumping, throwing) included a 1:1 ratio of activity to recovery: 30–60 s of activity and 30–60 s of recovery time (2 to four sets), working progressively. The supervised program consisted of a 5-minute warm-up period—involving comfortable running and joint movement exercises—followed by varied high-intensity exercises at 80–95% maxHR. In the last part of every class, a 5-minute cool-down period was performed to regulate body temperature and reduce HR levels. | **Key Findings:** Significant improvements in BMI (p<0.001) and six-minute walk test. The number of hypertensive children was reduced but the number of children with a prehypertensive condition remained unchanged, and the number of children with a normal condition increased (p < 0.001).  **Conclusion:** A 28-week program, applied during PE classes, achieved a significant reduction in the number of overweight, obese, prehypertensive and hypertensive children whilst improving CRF and anthropometric variables. | 71.43 |
| Delisle Nystrom | **Title:** A 12-month follow-up of a mobile-based (mHealth) obesity prevention intervention in pre-school children: the MINISTOP randomized controlled trial  **Year:** 2018 **Country:** Sweden | **Aims/Purpose:** To investigate if the MINISTOP intervention improved FMI and had a maintained effect on a composite score  **Sample Size:** 315  **Participants:** Children aged 4.5 years | **Duration:** 6 month, 12 month follow-up  **Diet/Nutrition +Healthy Lifestyle Education + Family Based:** Included a range of theory-based behaviour change techniques and was based upon current guidelines for a healthy diet and physical activity patterns. The app included 12 themes: healthy foods; breakfast; healthy small meals; physical activity and sedentary behaviour; candy and sweets; fruits and vegetables; drinks; eating between meals; fast food; sleep; food outside the home; and foods at special occasions. A new theme was introduced every second week. Parents had the ability to register their child’s intake of fruits, vegetables, candy, sweetened beverages and sedentary time within the app and received graphical feedback and automated comments at the end of every week. Within the app parents had the opportunity to submit questions to a dietitian and/or a psychologist. The parents of the children in the intervention group received the MINISTOP intervention via a web-based application for 6 months. | **Key Findings:** No significant difference was observed between the intervention and control group for FMI (p = 0.57), dietary or physical activity variables (p= 0.10 to 0.71).   Significant increase in FFM index (p=0.05)  **Conclusion:** The intervention effect observed at 6-month was not maintained at the 12-months, with no effect on FMI being observed at either follow-up. Future studies using mHealth are needed to investigate how changes in obesity related markers in young children can be maintained over longer time periods. | 85.71 |
| DeRenne | **Title:** Afterschool physical activity program to reduce obesity-related cancer risk: a feasibility study  **Year:** 2008 **Country:** USA | **Aims/Purpose:** To determine the feasibility of incorporating a physical activity intervention into the afterschool program as a method for achieving healthy body weights  **Sample Size:** 68  **Participants:** Children in sixth grade | **Duration:** 12 weeks  **Physical Activity:** School A followed the Exemplary PE Curriculum. This was designed to help youth attain necessary fitness levels, motor skills, knowledge, and attitudes to be fit for life. School A engaged students in a variety of daily, vigorous, purposeful, and planned activities and not just “recess play.” School B's supervisor had a background in PE and was a 4-year, competitive, interscholastic athlete. The activity program featured 3-week block sessions of flag football, basketball, volleyball, and softball. Children had an organized warm-up consisting of a 10-minute flexibility-calisthenics session followed by 10 minutes of aerobic jogging. After the warm-up, the teacher introduced each sport with a specific-sport skill session followed by the sport’s competitive game. | **Key Findings:** Students in School B significantly lowered BMI, increased sit-ups, and improved 3-minute walk-run test distance (p<.001). compared to School A. School A also had a slight decrease in sum of skinfolds (p<.05), a slight decrease in predicted % BF, increased distance covered in the 3-minute walk-run test.  **Conclusion:** Schools serious about improving the physical activity of their students should hire teachers with PE training and/or elementary PE. | 71.43 |
| *Di Pietro | **Title:** Role of camping in the treatment of childhood obesity  **Year:** 2004 **Country:** Italy | **Aims/Purpose:** To present the effect of a summer camp in obese children in Italy  **Sample Size:** 41  **Participants:** Obese children | **Duration:** 8 days + 1 year follow up  **Physical Activity:** Children played or participated to a physical activity program for at least 3-4 hours/day. Children agreed to participate in sport and in every activity for at least 3 hours/day.   **Diet/Nutrition:** The participants had 5 slightly hypocaloric meals a day. The staff of school-camp also provided a theoretical nutritional learning program.  **Healthy Lifestyle Education:** Obese children were encouraged in physical activity as much as possible (1 hour a day, or at least 4 hours a week), recommending walking to school rather than taking bus or cars, participating in sports or in other activities, changing their activity habits e.g. limiting the time spent watching television. | **Key Findings:** Decrease in FM in all obese children, with boys showing a further reduction in both weight excess and BMI and skinfolds, reduction in glycaemic and insulinemic levels, ACTH and cortisol levels among obese boys.  **Conclusion:** Intervention with dietary restriction with/without physical activity program in outpatient setting often fails. Camps for children provide more intensified interventions and may be useful to children weight management. | 85.71 |
| Domaradzki | **Title:** Effects of Tabata Training During Physical Education Classes on Body Composition, Aerobic Capacity, and Anaerobic Performance of Under-, Normal- and Overweight Adolescents  **Year:** 2020  **Country:** Poland | **Aims/Purpose: T**o evaluate the effects of a HIIT program based on the Tabata procedure on body composition, aerobic capacity, and motor performance **Sample Size:** 84 **Participants:** Children aged 16 years from a Polish secondary school | **Duration:** 10 weeks  **Physical Activity:** HIIT exercise regimen (14 minutes) baed on the Tabata training method, presented in the form of a video during one of three PE lessons per week. | **Key Findings:** Significant effects of HIIT on body weight (p<0.001), BMI (p<0.001), waist-hip ratio (p<0.001), %BF (p<0.001), aerobic capacity (p<0.001) and agility (p=0.010).  **Conclusion:** Tabata program represents a useful tool for combating the problem of being overweight while avoiding adverse effects in terms of weight and body fat reduction. | 100 |
| Donnelly | **Title:** Nutrition and physical activity program to attenuate obesity and promote physical and metabolic fitness in elementary school children  **Year:** 1996 **Country:** USA | **Aims/Purpose:** To implement a nutrition and activity program and demonstrate the effectiveness in modifying the diet, decreasing weight gain, increasing physical fitness, increasing the knowledge, awareness of diet in health and markers of health risk  **Sample SIze:** 200  **Participants:** Children in third to fifth grade | **Duration:** 2 years  **Physical Activity:**  Activities were designed to use large muscle groups for 30-40 minutes, 3 days/week. Aerobic activities that can be easily incorporated into the individual’s lifestyle were emphasised e.g. hopping, skipping, and games   **Diet/Nutrition:** Meals were planned with the existing kitchen staff to reflect the Lunchpower! Program (reduced energy, fat, and sodium for lunch). Average fat content is limited to 30% of the total energy with saturated fat comprising < 10%. Sodium is limited to 1000 mg, cholesterol to 100 mg, and dietary fibre is increased to 8 to 10 g.   **Healthy Lifestyle Education:** The existing classroom teachers delivered activities designed to promote energy expenditure and decrease time-off-task. | **Key Findings:** Total energy and fat significantly decreased. Percentage of total energy from carbohydrates showed significant increases. Percentage of protein were not significantly different between groups. Improved 1-mile run times and no change in aerobic capacity, %BF. Significant increases in BMI.  **Conclusion:** Although the physical activity intervention was marginally successful, the nutrition intervention was successful. Obesity and metabolic fitness components (except HDL-c) was unchanged in the intervention compared to the control. | 42.86 |
| Eichner | **Title:** A Physical Activity Intervention and Changes in BMI at a Middle School With a Large American Indian Population, Oklahoma, 2004-2009  **Year:** 2016 **Country:** USA | **Aims/Purpose:** The objective of this study was to determine the effect of a physical activity intervention at a middle school in southwestern Oklahoma on BMI-z score  **Sample Size:** 66  **Participants:** Children aged 12-15 years in sixth, seventh and eighth grade | **Duration:** 5 years  **Physical Activity:** The program’s objective was to habituate middle school students to daily walking or running. Participants walked or ran 1 mile each school day and then engaged in a team activity such as basketball, soccer, football, dodge ball, or volleyball. | **Key Findings:** Mean BMI z scores remained the same in the intervention and increased in control.  **Conclusion:** Health education curriculum that encourages a healthy lifestyle are important with evidence of positive impact on BMI z score. Our program demonstrated that improvements in BMI can be achieved by providing a well-regimented program of MVPA. | 42.86 |
| Elder | **Title:** Effects of a multi-pronged intervention on children's activity levels at recess: the Aventuras para Ninos study  **Year:** 2011  **Country:** USA | **Aims/Purpose:** To examine the extent to which a social and physical environmental intervention delivered by Community Health Advisors  **Sample Size:** 1206  **Participants:** Children aged 5-7 years | **Duration:** 2 years  **Physical Activity:** The school-based promotoras organised “walking clubs" for children. Participating children received stickers and other incentives such as water bottles, jump ropes, and small balls. The walking clubs were replaced by a new program called “Super Aventuras”. 1-3 stations operated at a time, including parachute games, aerobic dance, rope jumping, and a locomotor movement course. Signs were made to illustrate the movements, and cones and other colourful markers were used to mark the circular course.  **Environment:** The staff identified active games that used play area designs, tailored the designs with input from teachers, and then worked with principals and district staff to have initial area outlines painted with striping machines. | **Key Findings:** In areas with organised activities in VPA decreased (p < 0.05). In areas with equipment, nil overall improvements were seen in terms of walking, sedentary behaviour and proportion engaged in MVPA (p < 0.01).  **Conclusion:** The current intervention designed specifically for a Mexican- American community, should be modelled for future studies to further explore the management and treatment of childhood obesity. | 57.14 |
| *Epstein | **Title:** Effects of weight loss on fitness in obese children  **Year:** 1983 **Country:** USA | **Aims/Purpose:** To presents weight change and fitness data during a six-month interval  **Sample Size:** 113  **Participants:** Obese children | **Duration:** 4 months  **Physical Activity:** An exercise point system was developed to promote regular activity. Subjects were given lists of various activities with their corresponding point values. Children were instructed to meet weekly point goals that corresponded to caloric expenditure goals.  **Diet/Nutrition + Family-Based:** Parents and children were instructed in calorie counting and basic nutrition, using the traffic light diet. Children were instructed to consume between 900-1,200 calories /day and to eat no more than four red (high calorie) foods per week, and parents were instructed to remove red foods from the home. | **Key Findings:** Significant improvement in weight (p=.0001), average HR (p=.0001).  **Conclusion:** Weight loss in obese children are strongly related to improved fitness. Although the average child remained above their ideal weight there was reductions in maximal and recovery HR. Analyses revealed that the combination of effective weight loss and an initially moderate degree of overweight (40% to 50%) combined to produce the greatest fitness changes. | 71.43 |
| Erfle | **Title:** Effects of daily PE on physical fitness and weight status in middle school adolescents  **Year:** 2015 **Country:** USA | **Aims/Purpose:** To report the outcomes of the Active Schools Program and the effects of daily PE on physical fitness and weight status  **Sample Size:** 10206  **Participants:** Children in sixth to eighth grade | **Duration:** 1 year  **Physical Activity + Diet/Nutrition + Healthy Lifestyle Education:** Schools implemented an evidence-based PE program of their choice from a list including HOPSports®, SPARK™, CATCH®, Project Fit America®, Physical Best, and HEALTHY PE; or to propose and implement its own PE program to implement 30 minutes of daily PE. | **Key Findings:** Significant improvement in BMI, mile run, curl-ups, push-ups. The largest effect was observed for change in BMI among at-risk males (SMD=0.19; 95% CI [0.10, 0.28])  **Conclusion:** Significantly improved physical fitness and weight status among overweight/obese children. This supports integrating 30minutes of daily PE into middle school adolescents’ school day to improve health behaviours and reduce childhood obesity through regular physical activity. | 28.57 |
| *Farah | **Title:** Does exercise intensity affect blood pressure and HR in obese adolescents? A 6-month multidisciplinary randomized intervention study  **Year:** 2014 **Country:** USA | **Aims/Purpose:** To compare low-intensity vs. high-intensity aerobic exercise on blood pressure, HR in obese adolescents in a 6-month randomized multidisciplinary intervention trial  **Sample Size:** 43  **Participants:** Obese children | **Duration:** 9 months  **Physical Activity:** Participants in both HIIT and LIIT groups underwent treadmill training 3 times/week. HIIT group intensity corresponding to VTI. LIIT group exercised at a speed 20% below the VTI.  **Diet/Nutrition:** Nutritional group counselling (1 hour/week) addressed healthy eating behaviour, the food pyramid, weight loss diets, diet vs. reduced-calorie products, recording energy intake and provided general nutrition information. Subjects were not given specific diet prescriptions but were encouraged to reduce overall calorie intake of food and follow a balanced diet.  **Healthy Lifestyle Education:** Psychological (1 hour/week) group session addressed body image, eating disorders, relationship between food and feelings, family and social problems, mood, anxiety and depression. | **Key Findings:** The intervention demonstrated significant improvements in physical fitness and weight.  **Conclusion:** HIIT might produce a more favourable CVD risk profile in the obese adolescents than LIIT. Future research should focus on the sustainability of this type of intervention and the resulting impact on cardiovascular health. | 57.14 |
| Farias Edos | **Title:** Effects of programmed physical activity on body composition in post-pubertal schoolchildren  **Year:** 2015 **Country:** Brazil | **Aims/Purpose:** To investigate the changes in body composition after a physical activity intervention   **Sample Size:** 386  **Participants:** Children aged 15-17 years | **Duration:** 1 year  **Physical Activity:** 2 PE classes weekly, lasting 60 minutes each. Each class had 83 PE classes. Consisted of 3 parts: aerobic activity (exercises for flexibility, muscular strength, jumping rope, walking, alternating running, continuous jumping, recreational games), lasting 30 minutes; sports games (volleyball, soccer, handball), lasting 20 minutes; and with stretching, lasting 10 minutes. | **Key Findings:** A positive significant effect on body composition of adolescents was observed (p<0.001). Reductions in BF, WC, as well as an increase in LM were observed.  **Conclusion:** The regular practice of programmed physical activity, resulted in a significant reduction of BF in post-pubertal adolescents. | 71.43 |
| *Fogel | **Title:** The effects of exergaming on physical activity among inactive children in a PE classroom  **Year:** 2010 **Country:** USA | **Aims/Purpose:** To evaluate the effects of exergaming implemented in a typical PE classroom  **Sample Size:** 4  **Participants:** Obese children in fifth grade | **Physical Activity:** A classroom was converted into an exergaming lab in which nine stations had 11 activities with seven pieces of exergaming equipment available. The types of exergaming included the following: Kanomi Dance Dance Revolution, Gamercize, Batman and Robin, 3 Rivers Game Cycle, Monster 4 x 4, Electronic Sports Dog Fighter Simulator, Cateye Virtual Bike, Nintendo Wii Sports Baseball, Nintendo Wii Sports Tennis, Nintendo Wii Boxing, Tech Fitness XrBoard, Fit Interactive 3 Kick. | **Key Findings:** Higher levels of physical activity for all participants (mean of 9.2 minutes/session vs 1.6 minutes). The exergaming condition resulted in higher levels of opportunities (mean of 11.6 minutes of opportunities vs 3.8 minutes). The mean % of time spent engaged in physical activity was 78%.  **Conclusion:** Exergaming produced more physical activity across all four participants than did regular PE and could be a possible intervention choice for increasing physical activity among inactive 5th graders. Future research is needed to provide additional evaluation of the effects of exergaming on physical activity levels as well as health factors among inactive children. | 85.71 |
| Foster | **Title:** A school-based intervention for diabetes risk reduction  **Year:** 2010 **Country:** USA | **Aims/Purpose:** To evaluate the effects of a 3-year, multicomponent, school-based program on risk factors for type 2 diabetes.   **Sample Size:** 1736  **Participants:** Children from sixth grade | **Physical Activity:** The PE component was designed to increase the amount of time students spent in MVPA (>130 bpm).  **Diet/Nutrition + Environment:** The nutrition component targeted the quantity and nutritional quality of foods and beverages that were served throughout the school environment  **Healthy Lifestyle Education:**  Behavioural knowledge and skills were communicated with the use of a classroom-based program addressing self-awareness, knowledge, behavioural skills, and peer involvement for behavioural change. | **Key Findings:** Significant reductions in BMI with the mean BMI z score in the 90th percentile being significantly lower with time in the intervention group (p=0.04). This was not significantly different to the control group. No significant differences in mean plasma glucose levels.  **Conclusion:** The program did not result in greater decreases in the combined prevalence of overweight and obesity than control. | 42.86 |
| Gallotta | **Title:** Effects of combined PE and nutritional programs on schoolchildren's healthy habits  **Year:** 2016 **Country:** Italy | **Aims/Purpose:** To evaluate the effectiveness of different PE programs, in combination with a nutritional intervention, on schoolchildren's healthy habits  **Sample Size:** 230  **Participants:** Children aged 8-11 years from third to fifth grade | **Duration:** 5 months  **Physical Activity:** Warm-up (15 minutes), MVPA (35 minutes), cooldown and stretching (10 minutes). The experimental intervention 1 primarily focused on endurance, strength, flexibility exercises and circuit training for cardiovascular health. The experimental intervention 2 was focused on improving the coordination and dexterity of the participants. It was organised in 4 different didactic modules lasting 5 weeks each one.   **Diet/Nutrition + Healthy Lifestyle Education:** Topics discussed (monthly) included health/nutrition, science, affective and sensory, environmental, social. Staff provided 1 free piece of fresh fruit (or vegetable) weekly, as a snack. Every child consumed fruits or vegetables at least 36 times during the program, and at least ten different kinds of fruit or vegetable.   **Family-Based:** An information campaign targeted at parents on a specific website section | **Key Findings:** %FM, body weight, body height, lean body mass increased after intervention (all, p<0.0001). Physical activity time significantly increased (p<0.0001). Sedentary time significantly decreased (p<0.001). Consumption of potatoes, meat, eggs, cold cuts, sweets, dairy products, bread and snacks decreased but vegetables and fruits increased (all, p<0.05). No change in the consumption of pasta and rice, legumes and milk.  **Conclusion:** No significant changes in adiposity were observed, involvement of parents seems to be essential to reinforce patterns of healthful dietary practices, nutritional knowledge and physical activity. | 71.43 |
| Gao | **Title:** Home-based exergaming on preschoolers' energy expenditure, cardiovascular fitness, body mass index and cognitive flexibility: a randomized controlled trial **Year:** 2019 **Country:** USA | **Aims/Purpose:** To examine the effects of a home-based educational exergaming intervention on urban preschool children's energy expenditure, cardiovascular fitness, body mass index, and cognitive flexibility  **Sample Size:** 32 **Participants:** Children aged 4-6 years | **Duration:** 12 weeks  **Physical Activity + Family-Based + Environment:** Parent-child dyads were provided with a LeapTV gaming console all necessary peripherals, and several age-appropriate educational exergames (dance and sports) for use during their time completing the educational exergaming intervention. Parents were instructed to have their children perform educational exergaming on the LeapTV for 30 min/session, 5 times per week. Developed specific daily gameplay duration goals with the parents to ensure this dosage was achieved. | **Key Findings:** No significant difference in energy expenditure, cardiovascular fitness and BMI. Significant improvement in cognitive flexibility.  **Conclusion:** Participation in the home-based exergaming program has potential to improve preschool children’s cognition. No known studies have yet examined the effects of a developmentally appropriate and innovative educational exergaming intervention on preschoolers’ physical health and cognition at the home setting in the U.S. | 85.70 |
| Gao | **Title:** Impact of exergaming on young children's school day energy expenditure and moderate-to-vigorous physical activity levels  **Year:** 2017 **Country:** USA | **Aims/Purpose:** To investigate the long-term impact of a multigame exergaming intervention   **Sample Size:** 261  **Participants:** Children aged 7-9 years in second to third grade | **Physical Activity:** Exergaming and PE alternated on a daily basis (125 minutes/week). 12 stations were set up in a large classroom with each station equipped with 2 exergaming systems and a television. A number of exergames were offered, including but not limited to Kinect Ultimate Sports, Just Dance, Wii Sports, and Wii Fit, allowing a variety of choices and promoting autonomy and sustained motivation for participation throughout the duration of the intervention. Congruent with the definition of a multiactivity curriculum, 25 different learning activity or sport units (e.g., basketball, tag games, soccer, fitness, etc.) were taught in rotation every 4 weeks. | **Key Findings:** LPA and MVPA significantly increased (p < 0.01).  **Conclusion:** Exergaming elicited the same effect on children’s MVPA, LPA as PE and played into the higher MVPA levels seen at follow-up among intervention children. Future studies should explore maintenance of MVPA as it may eventually lead to integration into PE curriculum. | 57.14 |
| *Garcia-Hermoso | **Title:** The Intention to be Physically Active in Sedentary Obese Children: A Longitudinal Study  **Year:** 2018 **Country:** Spain | **Aims/Purpose:** To determine how a long-term program of physical exercise and a low-calorie diet influences sedentary obese children’s intention to be physically active  **Sample Size:** 27  **Participants:** Children aged 8-11 year, BMI >97th percentile | **Duration:** 3 years  **Physical Activity:** The exercise program consisted of 3x90 minute sessions/week. The sessions consisted of a warm-up (15–20 minutes), multi-sport games and aerobic exercise (MVPA) (60–65 minutes) combined with strength work, and a cool-down (5–10 minutes).  **Diet/Nutrition**: The low-calorie diet consisted of five balanced meals spread throughout the day, with an energy intake of 1500 kcal/day. The diet consisted of 57% carbohydrates, 17% proteins, and 26% fats. The subjects were given a list of food groups (vegetables, fruit, meat, fish, eggs, cereals, legumes, and dairy) and their cooking methods, the weekly consumption frequencies, and maximum daily amounts. General recommendations were established focused on basic healthy lifestyle eating. | **Key Findings:** Longitudinal changes in the intention to be physically active (p < 0.001). Daily physical activity increased significantly.  **Conclusion:** After a longitudinal exercise program with and without diet, obese children increased their intention to be physically active, with increased levels of daily physical activity not just immediately following the program but also after a period of detraining. This seems indicative of the suitability of exercise adapted to the obese population in generating and maintaining a greater intention to be physically active. | 71.43 |
| *Gentier | **Title:** Multidisciplinary residential treatment can improve perceptual-motor function in obese children  **Year:** 2015 **Country:** Belgium | **Aims/Purpose:** To evaluate the effect that this 10-month treatment program for obese children had on perceptual-motor function  **Sample Size:** 52  **Participants:** Children recruited from a local rehabilitation centre | **Duration:** 10 months  **Physical Activity:** Each child received 3 h of individual exercises per week, focusing on aerobic activities such as running, swimming or fitness training. In addition, the children participated in supervised team sports and group games for approximately 1 h a day or 5 h a week and also had the opportunity to be physically active in their free time.  **Healthy Lifestyle Education:** Received psychological support  **Diet/Nutrition:** Moderate dietary restriction – with a limit of 1450–2050 kcal/day | **Key Findings:** A significant improvement in weight, BMI and the % BF (all, p<0.001). All participants classified as healthy weight at baseline, maintained weight over time. Obese and healthy weight children’s performance on reaction time tasks did not change significantly over time.  **Conclusion:** Obese children’s suboptimal motor planning and control processes of discrete motor actions did not benefit from weight loss and increased physical activity. However, tracking performance significantly improved. | 57.14 |
| *Gesell | **Title:** Accuracy of perception of body size among overweight Latino preadolescents after a 6-month physical activity skills building intervention  **Year:** 2010  **Country:** USA | **Aims/Purpose:** To examine whether the intervention had an effect on how accurately children saw their body size over time  **Sample Size:** 61  **Participants:** Children aged 8-11 years, BMI>85th percentile | **Duration:** 6 months  **Hour-long month sessions included the following:  Physical Activity:** Practice the skills taught (e.g. soccer, dance, volleyball, and outdoor games) (30 minutes) where parent-child dyads participated in 6 monthly sessions between baseline and 6-month follow-up.  **Healthy Lifestyle Education:** A 20-minute skills building teaching session (e.g., how to recognise if you are exercising enough during aerobic activity; how to warm-up and cool-down; how to avoid dehydration; how to utilize a recreation facility; how to avoid cramps) and goal setting session.   **Family-Based:** The skills building topics and exercises incorporated into the sessions were informed by 3 focus groups with Latino parents and children. | **Key Findings:** At 6 months, 40.7% (11/27) in the intervention group rated their body size accurately compared to 21.2% (7/33) in the control.  **Conclusion:** There is a strong association between self-perceived weight status and weight control behaviour where for children to actively participate in early obesity intervention efforts they must first be aware they are overweight. Future studies should further explore this. | 28.57 |
| Going | **Title:** The effects of the Pathways Obesity Prevention Program on physical activity in American Indian children  **Year:** 2003 **Country:** USA | **Aims/Purpose:** To report the effects of the intervention on physical activity   **Sample Size:** 580  **Participants:** Children in second to fifth grade | **Duration:** 3 years  **Physical Activity:** PE (3 times/week, 30 minutes/class) was based on the SPARK program (Sports, Play and Active Recreation for Kids). 1-2 activities were performed to target a single fitness component (muscle strength, endurance, flexibility, aerobic endurance) or as circuits to address multiple components. Daily recess (15 minutes) and exercise breaks (1–2/day, 5–10 minutes) were encouraged.  **Healthy Lifestyle Education:** The program promoted positive attitudes toward physical activity and encouraged children to cultivate and sustain an active lifestyle.   **Environment:**  Centralised training sessions for PE specialists and classroom teachers, lasting 1.5 days, held before each fall and spring semester. | **Key Findings:** No differences between intervention and control were significant. Boys were significantly (p< .01) more active by 17.  **Conclusion:** The intervention did not result in significant increases in physical activity. Future studies should refine the methodology to ensure potentially important changes in physical activity can be detected. | 71.43 |
| Gortmaker | **Title:** Reducing obesity via a school-based interdisciplinary intervention among youth: Planet Health  **Year:** 1999 **Country:** USA | **Aims/Purpose:** To evaluate the impact of a school-based health behavior intervention known as Planet Health on obesity children  **Sample Size:** 1295  **Participants:** Children in sixth and seventh grade | **Duration:** 2 years  **Diet/Nutrition + Healthy Lifestyle Education + Environment:** The intervention focused on 4 behavioural changes including reducing television viewing to less than 2 hours /day, increasing MVPA, decreasing consumption of high-fat foods, increasing consumption of fruits and vegetables to 5 a day or more. In classroom lessons, each theme was addressed in 1 lesson/subject for a total of 16 core lessons each in year 1 and year 2. An additional lesson developed a 2-week campaign to reduce television viewing in households. Classroom lessons were designed for 1-2 45-minute periods, depending on the level of detail chosen by teachers. All teachers received training from project staff either in workshops, or with the field coordinator. | **Key Findings:** Obesity prevalence declined from 23.6%-20.3%. Television hours/day among girls reduced in the intervention (p=0.001) which was similar amongst boys (p=0.001)  **Conclusion:** Obesity reduced among girls, with no significant differences in boys. Reductions in television viewing time for both sexes, and girls in the intervention schools experiencing increased fruit and vegetable consumption, reduced dietary energy intake. Future studies should explore the relations between television viewing time and reductions in obesity. | 71.43 |
| *Graf | **Title:** Who benefits from intervention in, as opposed to screening of, overweight and obese children?  **Year:** 2006 **Country:** Germany | **Aims/Purpose:** To evaluate the effect on the anthropometric data, WC, and blood pressure in children undergoing intervention  **Sample Size:** 40  **Participants:** Overweight and obese children aged 6-11 years in first to fourth grade | **Duration:** 7 months  **Physical Activity:** Children received a physical activity program lasting between 60 and 90 minutes.   **Diet/Nutrition:** The nutritionists cooked and ate with the children twice a week. **Family-Based:** Parents were involved in 6 information evenings and 2 family events. | **Key Findings:** Height and weight increased significantly due to growth (p< 0.001). BMI did not change significantly (p<0.05). SBP decreased significantly. DBP values and HR did not differ, between the groups.  **Conclusion:** An interdisciplinary school- and family-based intervention was efficacious for overweight/ obese children in BMI, SBP when compared to the control group. | 85.71 |
| *Guerendiain | **Title:** Changes in plasma fatty acid composition are associated with improvements in obesity and related metabolic disorders: A therapeutic approach to overweight adolescents  **Year:** 2018 **Country:** Spain | **Aims/Purpose:** To explore the associations between anthropometric and cardiometabolic parameters and plasma fatty acid levels in overweight and obese adolescents subjected to a multidisciplinary anti-obesity program  **Sample Size:** 127  **Participants:** Overweight and obese children aged 12-17 years | **Duration:**  6 months  **Physical Activity + Diet/Nutrition + Healthy Lifestyle Education:** It comprised a calorie-restricted diet (10-40%), increased physical activity (at least 60 minutes/day, 5 days a week), psychological therapy and nutritional education. The macronutrient distribution was as follow: 50% of energy from carbohydrates, 30% from fat and 20% from proteins. | **Key Findings:** Myristic acid and stearic acid decreased significantly (p < 0.01), while total MUFAs increased (p < 0.001). Total PUFAs decreased (p < 0.05). Decreased myristic acid associated with a reduction HDL-c, LDL-c, apoB, insulin (all, p<0.05). Increases in MUFAs was inversely associated with weight, BF, FMI, glucose and HDL-c (all, p < 0.05).  **Conclusion:** In conclusion, we found that the modification in plasma fatty acid composition, especially PUFAs, MUFAs and myristic acid, is related to changes in adiposity and cardiometabolic risk factors in anti-obesity programs aimed at adolescents. | 85.71 |
| Gutin | **Title:** Preliminary findings of the effect of a 3-year after-school physical activity intervention on fitness and body fat: the Medical College of Georgia Fitkid Project  **Year:** 2008 **Country:** USA | **Aims/Purpose:** To evaluate the effect of a 3-year after-school physical activity intervention on aerobic fitness and % BF  **Sample Size:** 617  **Participants:** Children with mean age 8.5 years in third grade | **Duration:** 3 years  **Physical Activity:** Afterschool session offered 5 days/week on school days. Youth did not have to attend every day. The 80 minutes of physical activity included a variety of activities designed to improve sport skills, aerobic fitness, strength, and flexibility; 40 minutes were devoted to VPA. The activities were designed to be mastery-oriented rather than competitive.  **Diet/Nutrition:** A 40-minute period during which the youths were provided with a healthy snack and academic enrichment activities. No attempt was made to limit the energy intake of the children. | **Key Findings:** During the first year a favourable impact on % BF, HR and bone density in the intervention group, with greater attendance leading to greatest benefit. The intervention group improved in fitness, reduced FM during the school, returning after summer months to levels similar of the control. Significant group by time interactions of intervention for bone density, BMI (increase) but less significant for fat mass.  **Conclusion:** An after-school program emphasising large amounts of MVPA, without emphasis on restriction of energy intake, improving in aerobic fitness and body composition, have beneficial effects on fitness and fatness. | 28.57 |
| Halfon | **Title:** The influence of a physical ability intervention program on improved running time and increased sport motivation among Jerusalem schoolchildren  **Year:** 1988 **Country:** Germany | **Aims/Purpose:** To improve the physical ability of 13-year-olds and the sport motivation of the participants  **Sample Size:** 610  **Participants:** Children aged 13 years in eighth grade | **Duration:** 3 months  **Physical Activity:** The children ran in groups of five. There were 4-time units. The first was 5 minutes, the second 7 minutes, the third 9 minutes, the fourth 10 minutes. The children were required to increase the distance of their run over the 4-time units. Walking was not permitted. | **Key Findings:** Mean running time in the first 1000 m run was not significant compared to the 2nd. A statistical difference between 2nd, 3rd run was observed (p<0.001). Sport motivation in the intervention group decreased by an average 0.04, and 1.17 in the control. A significant difference in skinfold thickness.  **Conclusion:** Several health parameters were influenced, such as coronary heart disease risk profile. | 42.86 |
| Harrel | **Title:** School-based interventions improve heart health in children with multiple cardiovascular disease risk factors  **Year:** 1998  **Country:** Australia | **Aims/Purpose:** To determine the relative effects of a classroom based intervention and an individualised, risk-based intervention   **Sample Size:** 425  **Participants:** Children in third and fourth grade | **Duration:** 8 weeks  **Physical Activity:** Children received an aerobically oriented physical activity program 3 times/week, taught by physical educators using lesson plans developed by the exercise physiologist coinvestigator and derived from standard PE curriculum texts. Examples of the aerobic activities used are jumping rope to music, endless relay, parachute games, and aerobic dance.   **Diet/Nutrition + Healthy Lifestyle Education:** Content included information about selecting healthy foods, the importance of getting regular exercise, the dangers of smoking, and way to combat pressure to smoke. | **Key Findings:** MANOVA test of the physiologic variables demonstrated a significant intervention effect (p=0.021). Behavioural measures were also significant for intervention (p=0.004).  **Conclusion:** This classroom-based intervention was equally effective in improving CVD risk profiles compared an intensive intervention. The classroom-based approach is easier to implement, avoids stigmatisation, uses positive peer pressure, whilst providing some benefits to all children. | 85.71 |
| Harrison | **Title:** Influence of a health education intervention on physical activity and screen time in primary school children: 'Switch Off--Get Active'  **Year:** 2006  **Country:** Ireland | **Aims/Purpose:** To determine the efficacy of a health education intervention on BMI, targeted sedentary behaviours and physical activity  **Sample Size:** 312  **Participants:** Children in fourth grade | **Duration:** 16 weeks **Healthy Lifestyle Education:** Ten lessons of 30-minute duration were delivered. Children were required to reflect on how they spend their leisure time and challenged to identify realistic alternative to television viewing and computer game usage. A continuous aspect of the intervention was the self-monitoring, budgeting and goal setting practised to decrease screen time and increase physical activity. There was an attempt to revive traditional playground and street games, popular with previous generations of children but had fallen into decline.  **Family-Based:** Diaries formed part of the child's homework and were signed by parents | **Key Findings:** MVPA and self-efficacy for physical activity were higher in the intervention children (p<0.05), whereas screen time were not significantly difference (0.13) with BMI, aerobic fitness similar between intervention and controls. High users of television had a higher BMI, lower levels of MVPA, lower self-efficacy for physical activity and lower aerobic fitness (p<0.05).  **Conclusion:** Health education supplemented with specific lessons and activity-modification techniques has the potential to increase physical activity. Behavioural modification alone may not be sufficient to impact on screen time. | 85.71 |
| Hawthorne | **Title:** Grand Canyon Trekkers: school-based lunchtime walking program  **Year:** 2011 **Country:** USA | **Aims/Purpose:** To examine the effects of a structured walking program on components of health-related physical fitness   **Sample Size:** 1293  **Participants:** Children in kindergarten to sixth grade | **Duration:** 16 weeks  **Physical Activity:** The program was implemented during recess time, 3 days/week for 16 weeks. Each walker received an index-sized ‘‘mileage card’’ bordered with 20 hiking boots, each boot represented a ¼ mile, and a boot was marked off or hole punched by a volunteer for each ¼ mile lap completed. Upon completion of each 5-mile card, a new card was provided with the walker’s name, teacher’s name, and the number of the card. Incentives were provided at predetermined mileage intervals to encourage students to walk distances equivalent to the mileage of well-known hiking trails within the Grand Canyon, resembling a likeness to a ‘‘virtual trail.’’ | **Key Findings:** No significant difference observed for mean miles walked by gender. 5^th^ graders walked the most miles while kindergartners and 6^th^ graders the least. Obese participants walked significantly less compared to healthy weight. Cardio-respiratory fitness increased (p < .01).  **Conclusion:** The walking program proved to be an adequate supplement for physical activity at the elementary school level. | 71.43 |
| *Hayashi | **Title:** Echocardiographic and electrocardiographic measures in obese children after an exercise program  **Year:** 1987 **Country:** Japan | **Aims/Purpose:** To apply exercise training and study the influence on the anthropometric, hemodynamic and anatomic adaptive changes in their cardiovascular system  **Sample Size:** 18  **Participants:** Obese children aged 10 years | **Duration:** 1 year  **Physical Activity:** A jogging session held 5 days/week (22km/week) was conducted by the obese children. Each exercise session consisted of a 20-minute jogging session during the noon recess and after school. The objective was to maintain the 145-155 bpm after the 20-minute jogging session. | **Key Findings:** Obese children decreased weight, resting HR decreased. Left ventricular end-diastolic dimension increased significantly despite no difference in left ventricular posterior wall thickness and interventricular septal thickness.  **Conclusion:** Jogging 5 days/week may produce weight reduction, decrease resting HR and increase in left ventricular end-diastolic dimension. Future studies should continue to explore this. | 42.86 |
| *Hills | **Title:** Obesity management via diet and exercise intervention  **Year:** 1988 **Country:** Australia | **Aims/Purpose:** To assess the effectiveness of exercise and diet on specific body composition measures   **Sample Size:** 35  **Participants:** Obese and normal weight children | **Duration:** 16 weeks  **Physical Activity:** The program (weekly, 50 minutes) involved a warmup; gymnastics, dance and motor skill activities (20 minutes); games. The subjects were encouraged to participate in aerobic activity (20-minutes, 3-4 times/week).  **Diet/Nutrition:** Individual consultations with a dietitian was focused on food selection, goals and behavioural aspects such as eating slowly and self-monitoring. An on-going aspect of nutrition education included films, videotapes and impromptu discussions about nutrition and common dietary practices.  **Healthy Lifestyle Education:** Suggestions regarding the importance of increased habitual physical activity emphasised the necessity for increased daily activity levels and discouragement of sedentary pursuits such as television viewing.  **Family-Based:** Parental support in regard to caloric intake of the children and providing motivation to the children to resolve weight problems was encouraged. | **Key Findings:** Anthropometric changes were non-significant. Skinfold reductions were significant (p<0.001) for the intervention.  **Conclusion:** An exercise and diet program are effective in reducing skinfolds, % BF and body weight. This interrelated approach should be utilised with appropriate behaviour modification and familial support. | 57.14 |
| *Hintze | **Title:** Multidisciplinary program for obesity treatment: Summary of results with adolescents  **Year:** 2012  **Country:** Brazil | **Aims/Purpose:** To assess the effects of the multidisciplinary program on readiness to change, physical self-description and fitness   **Sample Size:** 33  **Participants:** Overweight children aged 10-17 years | **Duration:** 16 weeks  **Physical Activity:** Physical activities (3 times/week) included resistance training and stretching and aerobic exercises   **Diet/Nutrition + Healthy Lifestyle Education:** The classes included topics about the quantitative and qualitative characteristics of the healthy diet, highlighting the more common causes of impairment in the nutritional habits. Psychology (weekly) discussions included the reasons for change habits, factors that improve and others that can impair the changes at the long time and so on. | **Key Findings:** BMI, WC SBP, aerobic fitness, flexibility, abdominal strength and upper limb had significant improvements (p < 0.05). Hip circumference and waist-hip ratio were not significant.  **Conclusion:** Almost all anthropometric and physical fitness related variables had positive responses. | 71.43 |
| Hollis | **Title:** Effects of a 'school-based' physical activity intervention on adiposity in adolescents from economically disadvantaged communities: secondary outcomes of the 'Physical Activity 4 Everyone' RCT  **Year:** 2016  **Country:** Australia | **Aims/Purpose:** To reduce the decline in physical activity typically observed during adolescence. To determine whether the intervention impacted on adiposity outcomes (weight, BMI and BMI z-score)  **Sample SIze:** 1150  **Participants:** Children aged 12-13 years in seventh grade | **Duration:** 12 months  **Physical Activity:** Physical activity focused on short- and long-term actions and timelines, fitness assessments, recording actions and goal achievements, and rewards for goal attainment (for example, balls, wrist bands and drink bottles). All students participated an enhanced school sport program (10 weeks).  **Environmental:** PE teachers received 2 professional learning workshops (conducted at 6-month intervals). School policies were established or modified with the aim of enhancing students’ physical activity. For example; incorporating pedometer-based lessons with PE, offering the enhanced school sport program as a standard school sport option, routinely providing physical activity information to parents. Schools were provided with physical activity equipment and encouraged to offer supervised physical activity on at least 2 days per week during recess and lunch breaks. Schools were supported to host a physical activity expo that promoted local physical activity providers to students in Grade 8. Community physical activity providers were also promoted in school newsletters.  **Family-Based:** Information was regularly sent to the parents via existing school newsletters, the school website and newsletters on physical activity recommendations, school-based physical activity strategies, promotion of community physical activity providers and strategies to support their child’s physical activity. | **Key Findings:** Group-by-time effects for weight (p=0.01), BMI (p=0.01), BMI z-score (p=0.02) were observed in the intervention group  **Conclusion:** Findings are robust in the main analysis of weight, BMI and BMI z-score. Despite implementation over a 24-month period, the study did not assess whether changes were sustained longer term. Students recruited were moderate-to-large sized, socio-economically disadvantaged schools from one area in Australia, which affects generalisability. Future studies should address the above. | 85.71 |
| *Horsak | **Title:** Effects of a lower extremity exercise program on gait biomechanics and clinical outcomes in children and adolescents with obesity: A randomized controlled trial  **Year:** 2019 **Country:** Austria | **Aims/Purpose:** To evaluate a program that combines strength and neuromuscular exercises on dynamic control of the frontal knee and hip alignment during weight-bearing conditions  **Sample Size:** 35  **Participants:** Children aged 10-18 years, BMI >97th percentile | **Duration:** 12 weeks  **Physical Activity:** Each session lasted 60 minutes. A physical therapist supervised the program, which consisted of a warming up, strength exercises for the knee and hip muscles and neuromuscular exercises for the lower extremity and core muscles. The main intention of that program was to increase the children’s ability to maintain a controlled lower-extremity alignment during loading conditions such as single support during walking and stair climbing. Quadriceps strength training involved non-weight bearing and weight bearing exercises. The intensity of the exercises was monitored continuously and adjusted to the self-perceived level of effort for each participant throughout the program. | **Key Findings:** Self-rated pain across was reduced after the session with no significant interactions or main effects. Wilcoxon signed rank test indicated a significant increase of the hip abductor strength.  **Conclusion:** Despite small effects of this short-term intervention, there is potential to counteract progressive development of biomechanical malalignments of the lower extremity where knee health outcomes indicate that the EP did not cause more pain suggesting feasibility and the need for future investigations. | 71.43 |
| *Hystad | **Title:** A randomised study on the effectiveness of therapist-led v. self-help parental intervention for treating childhood obesity  **Year:** 2013 **Country:** Norway | **Aims/Purpose:** To compare the long-term effectiveness of these interventions by assessing changes in adiposity and dietary intake, by achieving changes in lifestyle based on the families’ situation at baseline  **Sample Size:** 99  **Participants:** Children aged 7-12 years, BMI z-score >2 | **Duration:** 24 months  **Diet/Nutrition + Healthy Lifestyle Education:** Topics included(10 sessions): goal setting; communication about obesity, diet and physical activity; physical activity; dietary habits; mastery and motivation; guidance and setting boundaries; the role of siblings and the social network; parent’s history of diet and physical activity; body image; vacations and birthday parties. Children participated in groups led by a clinical dietitian and a physiotherapist to establish regular mealtimes, increase the intake of fruits, vegetables and other high-fibre food, reduce the intake of added sugar and fat, conduct >1 hour/day MVPA and reduce sedentary behaviour <2 hours/day.   **Family-Based:** All families attended five individual counselling sessions with a clinical dietitian and physiotherapist to discuss the family's progress and to define new goals. | **Key Findings:** In both intervention groups, BF, BMI z-scores, energy intake significantly decreased.  **Conclusion:** Children acquired favourable changes in their eating habits, which were sustained 6-24 months during the treatment as well as 18 months after. The improvements in BF mass may contribute to a reduced risk for childhood obesity tracking into adult life. Future research should explore the optimal way to involve parents to achieve persistent, cost-effective reduction in the adiposity of obese children. | 100 |
| *Inoue | **Title:** Linear and undulating periodised strength plus aerobic training promote similar benefits and lead to improvement of insulin resistance on obese adolescents  **Year:** 2015 **Country:** Brazil | **Aims/Purpose:** To compare the effectiveness of 3 physical trainings on IR, and on anthropometric and metabolic profile in obese adolescents submitted to a long-term interdisciplinary therapy  **Sample Size:** 45  **Participants:** Obese children, BMI>95th percentile | **Duration:** 1 year  **Physical Activity:** The program included (3 times/week) aerobic training at VTI (± 4 bpm) on a treadmill or a cycle ergometer. The predominant aerobic training group was initiated with 14 weeks performing 60 minutes of aerobic exercises only. From 14 to 26 weeks the adolescents performed 30 minutes of aerobic plus 30 minutes of strength training non-periodised with sub-maximal repetitions All subjects performed ten exercises: bench press; leg press, sit-ups, lat pull-down, hamstring curls, lower back, military press, calf raises, arm curls, triceps pushdown.  **Diet/Nutrition + Healthy Lifestyle Education:** Energy intake was set at recommended levels. Adolescents received dietetic lessons and individual nutritional orientation. Individualised psychological therapy was recommended when weight problems or poor dietary habits were found. Obese adolescents visited the endocrinologist once each month. | **Key Findings:** Aerobic training improved TG, FFM. Linear periodisation improved FFM, maintained RMR values (all, p<0.01). Linear periodisation and undulating periodisation showed significant improvement in TC, LDL-c (p<0.05), with significant increase in adiponectin. Glucose values, including HOMA-IR and insulin reduced significantly (p < 0.01).  **Conclusion:** Interdisciplinary interventions improved BMI, fat mass. Linear periodisation and undulating periodisation were more effective than aerobic training at improving insulin sensitivity, metabolic, lipid profile, inflammatory states (adiponectin). | 57.14 |
| Irwin | **Title:** Get Fit with the Grizzlies: a community-school-home initiative to fight childhood obesity  **Year:** 2010 **Country:** USA | **Aims/Purpose:** To determine if the Get Fit with the Grizzlies program was an effective in improving students’ health knowledge and health behaviours  **Sample Size:** 17066  **Participants:** Children in fourth and fifth grade | **Duration:** 1 year  **Physical Activity:** Elementary students attended PE (once/week, >30 minutes). **Diet/Nutrition + Healthy Lifestyle Education:** A 6-lesson unit focused on nutrition and exercise. The Get Fit activity/food log booklet had log sheets to record physical activity minutes and the number of food group servings consumed/day.   **Environment + Family-Based:** PE teachers attended a half-day training workshop. Partnered with Kellogs to add healthy breakfast options. Parents/caregivers were included in the lessons via homework (and their signature was necessary on their Get Fit activity/food log. | **Key Findings:** Students showed significant improvement knowledge-based items (p<.05). Consumption of soda/soft drinks did not improve with a significant decrease in their ability to identify length of time they should be exercising daily  **Conclusion:** Targeting childhood obesity by local sport organisations with community sponsors for financial support are valuable. | 71.43 |
| Jakubowski | **Title:** The Smart Nutrition and Conditioning for Kids (SNACK) Program: An Approach to Increasing Nutrition Knowledge of Second-Grade Students  **Year:** 2018 **Country:** USA | **Aims/Purpose:** To evaluate aspects of the program and to determine if students would gain basic nutritional knowledge  **Sample Size:** 71  **Participants:** Children in second grade | **Duration:** 8 weeks  **Physical Activity + Diet/Nutrition + Healthy Lifestyle Education:** Health education was integrated into PE classes. PE lasted 40-45 minutes. Classes were set up to allow 10-15 minutes for Fundamental Integrative Training, 10-15 minutes of traditional PE (warm-up 10-12 minutes, primary PE 20-25 minutes, nutrition content/movement activities 10-15 minutes) | **Key Findings:** The intervention change was significant improving from 72.59% to 82.96% (p<0.05). Despite this, group analysis found significantly increased CATCH Nutritional Knowledge scores.  **Conclusion:** The SNACK program was a partial success in increasing nutrition knowledge with less little improvement in the control. | 57.14 |
| Jansen | **Title:** Effectiveness of a primary school-based intervention to reduce overweight  **Year:** 2011 **Country:** Netherlands | **Aims/Purpose:** To evaluate the effect of a school-based intervention program to reduce overweight and improve fitness  **Sample Size:** 2416  **Participants:** Children aged 6-12 years in third grade | **Duration:** 1 year  **Physical Activity:** The intervention constitutes of the implementation of 3 PE sessions/week by a PE teacher for grades 3-8 (6-12 years of age). Organised additional sport and play activities outside school hours which can be attended on a voluntary basis   **Healthy Lifestyle Education + Diet/Nutrition:** 3 main lessons on healthy nutrition, active living and healthy lifestyle choices adapted for each grade. Each lesson finishes with joint goal setting.   **Environment:** The intervention targets school policies and curriculum and is based on the theory of planned behaviour. | **Key Findings:** For grades 3-5, increase in WC was significantly smaller, improvements in the 20 m shuttle run. Prevalence of overweight increased by 1.3%  **Conclusion:** Results provide evidence for the effectiveness of the Lekker Fit! in reducing overweight and WC, improving fitness in primary school children grades 3 – 5. Future studies should continue exploring school-based programs which focus on physical activity. | 71.43 |
| Jiang | **Title:** The effects of a 3-year obesity intervention in schoolchildren in Beijing  **Year:** 2007 **Country:** China | **Aims/Purpose:** To measure the effects of a primary school-based intervention program on the prevalence of obesity in Beijing  **Sample Size:** 2489  **Participants:** Children in first to fourth grade | **Duration:** 3 years  **Physical Activity:** From Monday to Thursday, all the overweight and obese children, along with the children who failed to pass routine school PE tests were asked to run for 20 minutes after class.   **Diet/Nutrition + Healthy Lifestyle Education + Family-Based:** Nutrition education aimed at both the children and their parents and included health consequences of childhood obesity, the food pyramid, and elements of a healthy lifestyle. A ‘traffic light’ food item list was given to the parents to help children decrease their energy intake and consume a balanced diet. | **Key Findings:** Prevalence of overweight and obesity were significantly lower  **Conclusion:** Our intervention produced a significant decrease in the prevalence of overweight and obesity. Future interventions should consider social/family support and improved education for children. | 71.43 |
| *Jones | **Title:** Acceptability and Potential Efficacy of Single-Sex After-School Activity Programs for Overweight and At-Risk Children: The Wollongong SPORT RCT  **Year:** 2015 **Country:** Australia | **Aims/Purpose:** To assess the acceptability and potential efficacy of single-sex after-school physical activity programs  **Sample Size:** 37  **Participants:** Overweight or obese children aged 8-11 years | **Duration:** 7 months  **Physical Activity:** Games and activities in MVPA (2 times/week). Participated in a 4-week Pedometer Challenge during the 2-week school holiday. Recorded daily steps online and completed a virtual walk across Australia from the beach to the outback.   **Diet/Nutrition + Healthy Lifestyle Education:** Evidence-based information on healthy lifestyles for children focused on general health messages, such as healthy hearts, food groups and the importance of eating breakfast.   **Family-Based:** Participants were provided with a ‘Health Passport’ containing weekly challenges to be completed at home with parents. A reward system (e.g., stickers, certificates) was used to promote completion of the ‘Health Passport’ challenges. | **Key Findings:** Significant decreases in BMI percentile (p=.001).   Significant improvement by having 2 or more servings of fruit (p<.001), soda consumption (p=.002), physical activity (p<.001), sedentary behaviour (p<.001) over the previous week.  **Conclusion:** StayingFit appears to support healthy weight regulation, improve weight/shape concerns, and increase healthy food consumption in adolescents. Future research should explore similar internet-based, universal, targeted intervention within the school. | 85.72 |
| *Jones | **Title:** Healthy weight regulation and eating disorder prevention in high school students: a universal and targeted Web-based intervention  **Year:** 2014 **Country:** USA | **Aims/Purpose:** To enhance healthy living skills, encourage healthy weight regulation, and improve weight/shape concerns with an online program  **Sample Size:** 336  **Participants:** Overweight and healthy children in ninth grade | **Duration:** 12 weeks  **Physical Activity:** Interactive online exercises **Diet/Nutrition + Healthy Lifestyle Education:** The StayingFit program is a 12-session online program (1 session/week) promoting healthy weight regulation and improved weight/shape concerns and included positive body image, nutrition education, physical activity, communicating with peers and family about health, weight stigma and social pressures related to body image. | **Key Findings:** Significant intervention effects for BMI, BMI z-score, %FFM, %BF (all p<.05). Statistically significant differences between groups for fasting glucose, LDL-c, TG and fasting glucose, % time in sedentary behaviour and LPA.  **Conclusion:** The positive results suggest that after-school single-sex physical activity programs show potential for improving body composition, reducing adiposity, promoting healthy lifestyles and modifying obesity risk factors among school-age children. | 71.43 |
| *Julian | **Title:** Eccentric cycling is more efficient in reducing fat mass than concentric cycling in adolescents with obesity  **Year:** 2019 **Country:** France | **Aims/Purpose:** To compare the impact of an eccentric cycling program vs concentric cycling program on whole‐BF mass percentage  **Sample Size:** 23  **Participants:** Children aged 13-14 years, BMI >90th percentile | **Duration:** 12 weeks  **Physical Activity:** The training program consisted of 3 phases. Phase 1 (2 weeks of habituation) load corresponding to 20% VO2max with exercise duration gradually increased by 10‐minute increments up to 30 minutes. Then, the exercise intensity ramped up progressively by 10% until achieving 50% VO2max. Phase 2 10‐minute warm‐up on concentric cycle ergometers- 30% VO2max; 30 minutes eccentric/concentric cycling- 50% VO2max; 5‐minute cool down. Phase 3 10‐minute warm‐up on concentric ergocycles-30% VO2max; 30 minutes eccentric/concentric cycling-70% VO2max; 5‐minute cool down.   **Diet/Nutrition:** Nutritional education (45 minutes/fortnight). The adolescents followed a balanced normo‐caloric diet (35% lipids, 55% carbohydrates, 15% proteins (not exceed 0.9 g/kg/day). | **Key Findings:** %FM, BMI significantly decreased. V̇O2peak, quadricep strength increased (p<0.05). Decreased HOMA‐IR was significant (p<0.05) with significant decrease in insulinemia (p<0.05) and glycemia (p<0.05).  **Conclusion:** Both ECC and CON cycling trainings efficiently decreased FM, ECC induces greater FM reduction, strength gains, and IR improvements; representing the optimal modality to recommend for obese adolescents. | 71.43 |
| Jurg | **Title:** A controlled trial of a school-based environmental intervention to improve physical activity in Dutch children: JUMP-in, kids in motion  **Year:** 2006 **Country:** Amsterdam | **Aims/Purpose:** To present the effectiveness of a 1-year intervention on physical activity as well as its social cognitive determinants   **Sample Size:** 510  **Participants:** Children in fourth, fifth, sixth grades | **Duration:** 1 year  **Physical Activity + Healthy Lifestyle Education + Family-Based + Environment:** The physical environment included the availability of physical activities and sport and policies. JUMP-in consisted of six program components: school sports activities, a pupil follow-up system, The Class Moves!, in-class exercises, Choose your Card! lessons aimed at increasing awareness, parental information services and an Activity-week. | **Key Findings:** Intervention group in Grades 4 and 5 increased their time spent in physical activity despite no significant changes. Perceived advantage of physical activity was significantly improved in Grade 4 children. Habit strength of physical activity was higher in Grade 4 children.  **Conclusion:** JUMP-in was successful in influencing physical activity behaviour, providing the basis for similar future interventions. | 71.43 |
| Kain | **Title:** School-based obesity prevention intervention in Chilean children: effective in controlling, but not reducing obesity  **Year:** 2014 **Country:** Chile | **Aims/Purpose:** To report the effectiveness of the multicomponent primary prevention program   **Sample Size:** 1474  **Participants:** Children aged 6-8 years in first to third grade | **Duration:** 1 year  **Healthy Lifestyle Education:** Classroom education consisted of a brief theoretical part and practical work in the form of activities like painting and puzzles.  **Environment:** Teachers of PE classes from 1st–3rd grade were trained (6 hrs) by a specialist on the use of a book containing a leaflet for each class which includes drawings of different exercises. Teachers in intervention schools were evaluated by the study nutritionist in terms of how well they applied the nutrition contents by comparing the results of their mean scores during the first versus the second semester. | **Key Findings:** Average BMI remained unchanged. BMI Z-score declined significantly (p<0.001).  **Conclusion:** Our kiosk intervention failed in the absence of clear incentives to provide healthier choices or limit the energy-dense snacks and sugar-rich drinks. Significant improvements across genders and schools in physical fitness. This intervention produced a desirable outcome relative to baseline nutritional status. | 85.72 |
| Kain | **Title:** School-based obesity prevention in Chilean primary school children: methodology and evaluation of a controlled study  **Year:** 2004 **Country:** Chile | **Aims/Purpose:** To assess the impact of a 6 months nutrition education and physical activity intervention on adiposity and physical fitness  **Sample Size:** 3086  **Participants:** Children in first to eighth grade | **Duration:** 6 months  **Physical Activity:** Provision of physical activity (90 minutes/week) to 3rd to 8th grades mainly oriented toward a certain sport (soccer, basketball and volleyball). During 1 daily recess (15 minutes/day), music was played, and children were encouraged to dance, play ping-pong, basketball or volleyball.  **Healthy Lifestyle Education:** Build knowledge about the benefits and importance of health and active living.  **Environment:** 2 meetings with owners providing information on healthy diets and potential healthy snacks, in an attempt to influence food being offered to children.   **Family-Based:** 2 meetings with parents of children from 4th to 8th grades directed at healthy eating, obesity prevention and to reinforce national food-based dietary guidelines. | **Key Findings:** BMI Z declined significantly in children. Prevalence of obesity remained unchanged Median class time was 60 minutes during the first semester, increasing non-significantly to 64.8, 67.9 minutes in classes by trained and untrained PE teachers, respectively.  **Conclusion:** This 12-month multicomponent intervention was effective in controlling obesity, but not preventing it, specifically those with the highest BMI Z score. | 28.57 |
| *Karacabey | **Title:** The effect of exercise on leptin, insulin, cortisol and lipid profiles in obese children  **Year:** 2009 **Country:** Turkey | **Aims/Purpose:** To investigate the effects of exercise on serum leptin, insulin, cortisol and lipid profiles  **Sample Size:** 40  **Participants:** Obese male children aged 10-12 years | **Duration:** 12 weeks  **Physical Activity:** At each training session, those in the exercise group performed warm-up exercises lasting 5 – 10 minutes, followed by a 20 – 45 minutes walking–jogging exercise with a targeted HHR of 60 – 65%, and 5 – 10 minutes of relaxation exercises at the end of the exercise period. | **Key Findings:** There were significant decreases in weight (p=0.01), BMI (p=0.001), leptin (p=0.001), cortisol (p=0.001) insulin levels (p=0.04), LDL-c levels (p=0.001) but increases in HDL-c (p=0.001).  **Conclusion:** Significant decrease in the risk of chronic disease associated with obesity is achievable if pharmacological treatment of obesity is combined with exercise and dietary intervention. | 42.86 |
| *Kaufman | **Title:** Aerobic-exercise training improves ventilatory efficiency in overweight children  **Year:** 2007 **Country:** USA | **Aims/Purpose:** To investigate the effect of an 8-week aerobic exercise training program on ventilatory threshold and ventilatory efficiency   **Sample Size:** 10  **Participants:** Overweight children aged 8-14 years | **Duration:** 8 weeks  **Physical Activity:** Participants in the exercise group trained on a stationary cycle ergometer four times per week starting at 50–60% of VO2max for 30 minutes per session. Intensity or duration of exercise was increased weekly until participants were exercising at 70–80% of VO2max for 50 minutes during the final 2 weeks. | **Key Findings:** Significant improvements in the intervention group for ventilatory efficiency (p<.05) potentially as a result of improvements in volume of oxygen at VT attributed to the training effect  **Conclusion:** 8 weeks of aerobic cycle training improves cardiopulmonary function and ventilatory efficiency in overweight children. Overall findings indicate reduced cardiovascular function and ventilatory efficiency | 57.14 |
| *Khammassi | **Title:** Health-related quality of life and perceived health status of adolescents with obesity are improved by a 10-month multidisciplinary intervention  **Year:** 2019 **Country:** France | **Aims/Purpose:** To assess the impact of a 10-month multidisciplinary weight management intervention on quality of life and health perception, body weight and body composition  **Sample Size:** 36  **Participants:** Obese children aged 11-15 years | **Duration:** 10 months  **Physical Activity + Diet/Nutrition + Healthy Lifestyle Education:** The program combined nutritional counselling, physical activity and health-related therapeutic education | **Key Findings:** Body weight, BMI, and total FM significantly reduced when compared to baseline (p<.0001), FFM remained stable. Significant improvements in physical functioning (p<.01), general health (p<.01), physical (p<.001) and mental score (p<.01), perceived physical condition (p<.01), adiposity (p<.0001), healthy balanced diet (p<.0001), general health (p<.05), and general health perceived (p<.0001).  **Conclusion:** This intervention favours both quality of life and health perception improvements in adolescents with obesity. | 85.72 |
| *King | **Title:** Go Girls!-Dance-Based Fitness to Increase Enjoyment of Exercise in Girls at Risk for PCOS  **Year:** 2019 **Country:** USA | **Aims/Purpose:** To introduce girls to a fun, non-threatening environment of exercise with peer support, and improve scores on a physical activity enjoyment scale   **Sample Size:** 27  **Participants:** Obese female children aged 7-21, BMI >85th percentile | **Duration:** 3 months or 6 months  **Physical Activity + Healthy Lifestyle Education:** Go Girls! is a dance-based fitness support group. Weekly sessions consisting of 45 minutes of dance-based exercise (primarily Zumba® Fitness or Kukuwa® African Dance) followed by 15–20 minutes of educational discussion about health-related topics, including complications of IR and PCOS. Participants set “SMART” goals and attainment for lifestyle modification.   **Diet/Nutrition:** Partnered with a local health-conscious restaurant (Roots Natural Kitchen) to provide cooking demonstrations/taste tests for participants. | **Key Findings:** BMI, BMI%, or z-score, WC, free testosterone, PACES scores was not different post-program (all, p>0.1). SBP percentiles were significantly lower (p = 0.04). Most claimed that they enjoyed PE, were exercising outside school and reported making lifestyle changes such as drinking fewer sugary beverages, less screen time, watching portion sizes  **Conclusion:** The intervention demonstrated several important benefits such as improved SBP. The supportive atmosphere provided a positive environment where girls demonstrated increased self-confidence during physical movement and social engagement. | 85.72 |
| *Kirschenbaum | **Title:** Treatment of morbid obesity in low-income adolescents: effects of parental self-monitoring  **Year:** 2005 **Country:** USA | **Aims/Purpose:** To examine the extent to which consistency of self-monitoring by participants and their parents was related to weight control   **Sample Size:** 83  **Participants:** Obese children mean age 13 years | **Duration:** 12 weeks  **Physical Activity:** A structured 12-week exercise program.  **Healthy Lifestyle Education:** The program included cognitive-behaviour therapy provided to small groups including self-monitoring and techniques such as stimulus control, chaining, stress management, and goal setting.   **Diet/Nutrition:** Monthly nutrition classes  **Family-Based:** Parents and participants signed a contract to self-monitor eating and exercising behaviours. | **Key Findings:** Participants monitored most consistently lost weight significantly (p =0.02).  **Conclusion:** These results indicate that self-monitoring is a cornerstone of successful weight control for morbidly obese low-income adolescents. | 42.86 |
| Klakk | **Title:** Effect of four additional PE lessons on body composition in children aged 8-13 years--a prospective study during 2 school years  **Year:** 2013 **Country:** Denmark | **Aims/Purpose:** To evaluate the effect of four additional PE lessons per week at primary schools on body composition and weight status  **Sample Size:** 632  **Participants:** Children aged 8-13 years in second to fourth grade | **Duration:** 2 years  **Physical Activity:** Implement four additional PE lessons per week to their usual PE program (resulting in a minimum of 4.5 hours PE per week divided over at least 3 sessions of at least 60 minutes)  **Environment:** Educate the specialized PE-teachers in specific age-related training principles. | **Key Findings:** No significant effect on BMI or %BF. Significant improvement on overweight/obesity prevalence and risk (p = 0.01)  **Conclusion:** No significant improvement in BMI or %BF despite significant improvement in the prevalence of overweight/obesity in primary schoolchildren. School based preventive interventions on body composition are likely to be less successful unless the effect is measured in high risk subgroups. | 42.86 |
| Kriemler | **Title:** Effect of school based physical activity program (KISS) on fitness and adiposity in primary schoolchildren: cluster randomised controlled trial  **Year:** 2010 **Country:** Switzerland | **Aims/Purpose:** To assess the effectiveness of a school based physical activity program during 1 school year on physical and psychological health  **Sample Size:** 502  **Participants:** Children in first to fifth grade | **Duration:** 1 year  **Physical Activity:** 2 additional PE lessons on the remaining school days. 3 to five short activity breaks (2 to five minutes each) during academic lessons—comprising motor skill tasks such as jumping or balancing on 1 leg, power games, or coordinative tasks—were introduced every day. The children received daily physical activity homework of about 10 minutes’ duration prepared by the PE teachers. This included aerobic, strength, or motor skill tasks such as brushing their teeth while standing on 1 leg, hopping up and down the stairs, rope jumping, or comparable activities.  **Environment:** A team of expert PE teachers prepared all five PE lessons for the children in the intervention group | **Key Findings:** Smaller increases in skinfolds and increased children’s aerobic fitness by. Positive change in MVPA post intervention was significant. Change in overall daily physical activity and quality of life was non-significant. Larger reductions in BMI and CVD risk factors- TG, HDL-c, glucose.  **Conclusion:** A school based, multi-component physical activity intervention including compulsory elements improved physical activity, fitness and reduced adiposity in children. | 71.43 |
| Krombholz | **Title:** The impact of a 20-month physical activity intervention in child care centers on motor performance and weight in overweight and healthy-weight preschool children  **Year:** 2012 **Country:** Germany | **Aims/Purpose:** To determine if daily child-centred physical activity would promote motor performance and reduce weight, BMI and skinfold thickness  **Sample Size:** 253  **Participants:** Children attending childcare centres | **Duration:** 20 months  **Physical Activity:** Children had more opportunities for unstructured and structured physical activities, which comprised activities like running, jumping, climbing, kicking, throwing and catching, and movement games. Children received PE sessions (45 minutes/week) and organised games (>20 minutes/day).  **Environment:** If necessary, the indoor and outdoor environments were rearranged to be more activity friendly. In addition, they were provided with educational materials for the implementation of physical activities programs. | **Key Findings:** High weight children in the intervention group showed an increase in motor skills (p= 0.18).  **Conclusion:** Children in the intervention surpassed control children in motor performance despite the strength of association between intervention and performance not being high. Increased physical activity had no effect on indices of body weight (BMI and skinfold thickness). | 57.14 |
| Kuhr | **Title:** Three times as much PE reduced the risk of children being overweight or obese after five years  **Year:** 2019 **Country:** Denmark | **Aims/Purpose:** To evaluate the effect that increasing PE lessons had on the BMI and WC   **Sample Size:** 1299  **Participants:** Children aged 5-11 years | **Duration:** 5 years  **Physical Activity:** Four extra PE lessons per week, in addition to the usual PE program for all children from pre-school to the fourth grade. These children, who were aged 5-11 years, got a minimum of 4.5 hours of PE classes per week, including 3 sessions that lasted at least 60 minutes. | **Key Findings:** Mean BMI increased (p=0.048). No change in average WC (p= 0.699) but participants with abdominal obesity at baseline showed considerable decreases in WC over the study period.  **Conclusion:** Providing 4.5 hours of PE lessons rather than the Danish standard of 1.5 hours, showed a favourable five-year effect on mean BMI values. It decreased risk of remaining overweight abdominally obesity with a positive impact on undesirable weight gain. | 71.43 |
| *Labayen | **Title:** Effects of Exercise in Addition to a Family-Based Lifestyle Intervention Program on Hepatic Fat in Children with Overweight  **Year:** 2019 **Country:** Spain | **Aims/Purpose:** To determine if supervised exercise, family-based lifestyle and psycho-education results in greater reduction of percentage of hepatic fat, adiposity and cardio-metabolic risk factors  **Sample Size:** 116  **Participants:** Overweight and obese children aged 10-11 years | **Duration:** 22 weeks  **Physical Activity:** Children attended 3 exercise program sessions per week. Sessions were supervised by exercise specialists and consisted of 5 minute of instruction time, 10 minute warm-up, 60 minute game-based cardiovascular endurance, 10 minute muscle strength exercises, 5 minute cool down and stretching exercises (overall, 90min/session) **Diet/Nutrition + Healthy Lifestyle Education + Family-Based:** Parents/caregivers and children separately attended the lifestyle (45 minutes) and psycho-educational (45 minutes) programs once every 2 weeks. The focus of the program was to increase: the parents' and children's knowledge about healthier dietary habits, their physical activity level, to promote sleep hygiene. | **Key Findings:** No significant differences were seen between the control and intervention in terms of any of cardiometabolic or type 2 diabetes risk factors as baseline. Significantly reduced BMI, FMI, and abdominal fat values.  **Conclusion:** A family-based multicomponent intervention including supervised exercise, lifestyle and psychoeducation, reduced hepatic and abdominal fat, IR, to improve dietary habits and psychological wellbeing in overweight/weight children. Future studies should explore such programs as part of paediatric obesity treatment. | 85.71 |
| *Lambrick | **Title:** The effectiveness of a high-intensity games intervention on improving indices of health in young children  **Year:** 2016 **Country:** UK | **Aims/Purpose:** To assess the effectiveness on improving physiological and anthropometrical indices of health and fitness   **Sample Size:** 55  **Participants:** Healthy-weight and obese children aged 8-10 years, BMI >95th percentile | **Duration:** 6 weeks  **Physical Activity:** Participation in twice weekly 60-minute exercise sessions, of which children were physically active for 40 minutes/session. There was a minimum 48-hour recovery period between sessions. Ensured children took part in each game at a high-intensity, different games were used for each 6-minute exercise period. Children took part in 6 games/session and a 4-minute circuit. | **Key Findings:** No differences in the rate of change in body mass or stature. Improved WC, MM. Significant increase in VO2max, peak running speed for intervention but not control.  **Conclusion:** Games-based exercises can improve a number of health-related outcome measures including VO2max and peak running speed, reduced O2 cost during submaximal exercise, improved WC, MM for those in the intervention group. | 85.71 |
| *Larsen | **Title:** The effect of a multi-component camp-based weight-loss program on children's motor skills and physical fitness: a randomized controlled trial  **Year:** 2016 **Country:** Denmark | **Aims/Purpose:** To determine how an immersive day-camp intervention program is influencing the development of motor skills and physical fitness in children  **Sample Size:** 115  **Participants:**  Children aged 12 years in fifth grade, BMI >25 | **Duration:** 6 weeks  **Physical Activity:** The camp lasted for six consecutive weeks, seven days a week, from 7 a.m. until 8.30 p.m. Children were engaged in physical activity classes (>3 hours) of structured exercise (e.g. dancing, team building, and alternative ball-games), health classes (1 hour) (focused on knowledge, theory, and behaviour change), and homework assignment (1 hour).  **Diet/Nutrition:** Healthy food was prepared by trained kitchen staff and the meals were supervised by the camp instructors. No diet restrictions were enforced.   **Family-Based:** After six weeks of day-camp intervention, a family-based intervention was initiated with the purpose of supporting the families in adopting the lifestyles attained during the day-camp intervention. Parents received health information e.g. about how to increase habitual physical activity and prepare healthy food. | **Key Findings:** Motor skills remained unchanged between groups during the entire trial. Physical fitness improved (p= 0.03). Effective in reducing BMI and BMI z-score  **Conclusion:** A small improvement in motor skills and physical fitness. Day-camp participants improved balance skills. Future studies should explore immersive intervention programs. | 85.71 |
| *Lau | **Title:** Effects of high-intensity intermittent running exercise in overweight children  **Year:** 2015 **Country:** China | **Aims/Purpose:** To examine the effects of a 6-week intermittent exercise intervention on body composition, functional walking and aerobic endurance performance   **Sample Size:** 48  **Participants:** Overweight children | **Duration:** 6 weeks  **Physical Activity:** Performed intermittent running (3 times/week). Participants had to run for 15 seconds at 100% or 120%, followed by 15 seconds of passive recovery. In order to match the distance coverage between the 2 intermittent exercise groups, LIIT performed more intervals (16 intervals, 8 minutes in total) and HIIT performed fewer intervals (12 intervals, 6 minutes in total). Attended the regular PE lessons (twice/week for 35 minutes). | **Key Findings:** No significant difference in % change of BMI. HIIT group had significantly improved (p<0.05) skinfold thickness and fewer steps during the obstacle test as compared with LIIT group and control. HIIT and LIIT had improvement in the intermittent aerobic endurance (p<0.05).  **Conclusion:** LIIT and HIIT may contribute to exercise behavioural by time efficiency, perception of tolerable physical exertion and thus improved adherence. | 42.86 |
| *Lee | **Title:** The Impact of a School-Based Weight Management Program Involving Parents via mHealth for Overweight and Obese Children and Adolescents with Intellectual Disability: A Randomized Controlled Trial  **Year:** 2017 **Country:** China | **Aims/Purpose:** To determine if school-based weight program involving parents via mHealth tools effects weight, knowledge and lifestyles.  **Sample Size:** 115  **Participants:** Overweight and obese children aged 8-16 years from special schools | **Duration:** 6 months  **Physical Activity:** Routine PE lesson (45 minutes) that were held twice per week  **Diet/Nutrition + Healthy Lifestyle Education + Family-Based:** The program promotes healthy eating and regular exercise via 24 training sessions at school and also extended to the home via mHealth tools to encourage parental involvement. Parents were encouraged to attend seminars, parent–child health promotion activities and regular dietary consultation sessions on a voluntary basis. The activities included posters to promote healthy lifestyle behaviours, and scheduled health talks on dietary habits. The mHealth tools were also introduced, and parents were recruited to promote healthy lifestyle behaviours. | **Key Findings:** Weight, BMI, skinfold thickness was significantly reduced. Lifestyle health knowledge scores in food pyramid tests, sports pyramid tests (p < 0.001), snack choice tests (p = 0.04) were significantly different. Scores of nutritional self-efficacy and self-efficacy in peer interaction were higher and significant.  **Conclusion:** Focusing on increasing both individual self and family collective efficacy is important to increase the likelihood of success with weight management interventions. | 71.43 |
| *Lee | **Title:** Effects of Exercise Modality on Insulin Resistance and Ectopic Fat in Adolescents with Overweight and Obesity: A Randomized Clinical Trial  **Year:** 2019 **Country:** Korea | **Aims/Purpose:** To examine whether a combined aerobic exercise and resistance exercise is more effective than aerobic or resistance exercise alone in improving insulin sensitivity and reducing body fat  **Sample Size:** 118  **Participants:** Overweight and obese children with sedentary lifestyles | **Duration:** 6 months  **Physical Activity:** All exercise sessions were by appointment and were supervised by an exercise physiologist or exercise science students. All participants were asked to attend exercise sessions, 3 times/ week, 60 minute/session, for 6 months. Participants in the aerobic exercise and combined resistance groups wore a HR monitor during aerobic exercise sessions to ensure achievement of the target HR (50%-65% of VO2max).   **Diet/Nutrition:** Participants were asked to follow the calorie intake targets determined at baseline to ensure that the negative energy balance was induced by regular exercise alone and not from dieting.  **Family-Based:** 8 of the 24 sessions involved parental participation in promoting participants' physical activity levels | **Key Findings:** Improvement in insulin-stimulated glucose disposal in the aerobic exercise group was greater than in the resistance exercise group (p<0.05). Fasting glucose levels did not change in any of the exercise groups. Significant reductions in weight, BMI, WC, %BF was observed in the aerobic exercise group (p<0.05). CRF and muscular strength increased independent of exercise group (p<0.05).  **Conclusion:** All 3 exercise modalities are beneficial in reducing total fat and skeletal muscle lipids and improving risk factors for T2DM. | 71.43 |
| *Lee | **Title:** Effects of regular exercise on obesity and type 2 diabetes mellitus in Korean children: improvements glycemic control and serum adipokines level  **Year:** 2015 **Country:** Korea | **Aims/Purpose:** To clarify the effects of regular exercise on lipid profiles and serum adipokines   **Sample Size:** 30  **Participants:** Healthy-weight and overweight children with type 2 diabetes mellitus, BMI>95th percentile | **Duration:** 12 weeks  **Physical Activity:** Aerobic exercise was conducted for 40–60 minutes/session (4 times/week). Participants achieved 50% of their oxygen consumption through the VO2max test. In weeks 1–4, participants engaged in 30–40 minutes of aerobic exercise (walking/running). Each session was preceded and followed by a 5-minute warm-up and cool-down | **Key Findings: Key Findings:** Body weight, BMI, and %BF, TC, TG, and LDL-c significantly decreased (p<0.05). VO2max, HDL-c was significantly increased (p<0.05).  **Conclusion:** Improved VO2max, ameliorated lipid profiles, glycaemic control, decreased adipokines levels. This demonstrates that regular exercise is beneficial for glycaemic control in Korean children with obesity or T2DM. | 57.14 |
| *Leite | **Title:** Effects of physical exercise and nutritional guidance on metabolic syndrome in obese adolescents  **Year:** 2009 **Country:** Brazil | **Aims/Purpose:** To evaluate the effect of physical training and nutritional guidance on body composition, physical fitness, lipid profile and IR   **Sample Size:** 64  **Participants:** Obese children aged 10-16 years +/- metabolic syndrome | **Duration:** 12 weeks  **Physical Activity:** Exercise sessions (2-3 times/week) consisted of 50 minutes of indoor cycling, 50 minutes of outdoor walking/running and 20 minutes of stretching. During the first four weeks (12 sessions), the intensity was set at 35-55% of VO2max, and this was increased to 55-75%.  **Diet/Nutrition + Healthy Lifestyle Intervention:** The obese participants with and without metabolic syndrome received nutritional and lifestyle education (4 times – 2x individual sessions, 2x groups). The prescribed nutritional guidance was based on an exchange list, by reducing 500 kilocalories (kcal) from the calorie total in the diet, in order to promote a 0.5kg reduction per week (2kg per month). | **Key Findings:** Reduced body mass (p=0.001), BMI z-score (p<0.001), WC (p<0.001), %BF (p=0.001), FM(p=0.001), resting HR (p=0.003) and maxHR (p=0.022). FFM was unchanged (p>0.05). Height (p<0.001), VO2max (p=0.011) increased. Reduced TG (p<0.001), glucose 120 (p=0.005), insulin 120 (p<0.001). Increased HDL-C (p<0.001) and QUICKI (p=0.021). No differences (p>0.05) in SBP, DBP, TC, LDL-C, glucose, insulin, HOMA-IR and QUICKI.  **Conclusion:** A multidisciplinary intervention in preventive measures and therapeutic procedures for obese children and adolescents improved their physical fitness and metabolic profile. | 71.43 |
| *Leite | **Title:** Age and menarcheal status do not influence metabolic response to aerobic training in overweight girls  **Year:** 2013 **Country:** Brazil | **Aims/Purpose:** To determine the effects of a 12-week multidisciplinary intervention, based on aerobic training, on metabolic markers   **Sample Size:** 85  **Participants:** Overweight female children aged 10-16 years | **Duration:** 12 weeks  **Physical Activity:** Exercise sessions (2-3 times/week) consisted of indoor cycling (45 minutes), outdoor walking/running (45 minutes), stretching (20 minutes). Intensity was set as 35–55% of HHR and was increased to 55–75% during the final eight weeks.  **Diet/Nutrition:** The nutritional intervention involved a qualitative and quantitative analysis of the subjects’ food intake. The diet emphasised the consumption of vegetables, fresh fruit, regular consumption of dairy products, fish and poultry consumed in low to moderate amounts, and a reduced intake of red meat. Total fat in this diet is 25% to 35% of the total caloric intake.   **Healthy Lifestyle Education:** All participants were encouraged to maintain an active lifestyle during and after the multidisciplinary program. The adolescents participated in educational meetings once/month for 60 minutes on physical activities and reduction of sedentary behaviour, such as watching television or playing computer games.  **Family-Based:** Families were provided with additional nutritional instruction, including interpretation of food labels and shopping, and were taught stimulus control to reduce access to high-calorie foods and increase access to healthy lower-calorie foods. | **Key Findings:** No differences in height, body mass and BMI during the 12-week training. The trend seems to be possibly beneficial in the WC and BMI z-score. Individual responses for the intervention group in TG (p<0.01), insulin (p<0.05) substantially decreased with HDL-C substantially increasing (p<0.01).  **Conclusion:** This intervention improved BMI z-score, WC, HDL-C, TG and insulin. Observed effects could not be associated with age and maturity status. Future analyses need to examine the relation between training responses, growth and maturation, through the observations of somatic maturation and skeletal age. | 57.14 |
| Li | **Title:** Effectiveness of a school-based physical activity intervention on obesity in school children: a nonrandomized controlled trial  **Year:** 2014 **Country:** China | **Aims/Purpose:** To identify the effectiveness of a school-based multi-component physical activity intervention  **Sample Size:** 921  **Participants:** Children aged 7-15 years | **Duration:** 12 weeks  **Physical Activity:** The program included PE improvement (3 times/week, 45-minute sessions), extracurricular physical activity (aerobics, jogging, rope jumping, other games).  **Diet/Nutrition + Healthy Lifestyle Education:** Students were taught how to judge exercise intensity and required to write exercise diary each day. Health education lectures for included the cause and harms of childhood obesity, BMI reference for screening overweight and obesity in Chinese school-age children, healthy eating (increasing consumption of vegetables and fruits, reducing consumption of meat, snacks, western fast foods and eating in restaurants, avoiding sugary drinks), and physical activity (intensity, duration, reducing sedentary time). | **Key Findings:** Significant reduction of BMI, incidence of overweight/obesity (p=0.015), triceps, subscapular and abdominal skinfold thickness, fasting glucose. No significant difference in WC and serum lipids (all p> 0.050). The increase of total MVPA duration was statistically significant.  **Conclusion:** A significant decrease in BMI, skinfold thickness, fasting glucose, and increased MVPA. These findings provide evidence for the development of effective and feasible school-based obesity interventions. | 100 |
| *LoMauro | **Title:** Effects of a multidisciplinary body weight reduction program on static and dynamic thoraco-abdominal volumes in obese adolescents  **Year:** 2016 **Country:** Italy | **Aims/Purpose:** To characterize static and dynamic thoraco-abdominal volumes in obese adolescents and to test the effects of a 3 weeks multidisciplinary body weight reduction program   **Sample Size:** 11  **Participants:** Obese male children | **Duration:** 3 weeks  **Physical Activity:** Aerobic physical activity program, including 2 30-minute sessions/day of cycle ergometer pedalling, treadmill walking, and stationary rowing, carried out in the afternoon for 5 days/week. The intensity of exercise was set at an average HR between 60% and 80% of the individual’s age predicted maxHR. Respiratory muscle endurance training performed for 5 days/week, 1 session/day, 12-18 minute/session, ~25 respiratory acts/session using a commercially available device.   **Diet/Nutrition + Healthy Lifestyle Education:** Personalized diet, daily monitored by a dietitian, formulated according to the Italian recommended daily allowances, involving an energy intake ~500 kcal lower than the measured resting energy expenditure. Psychological and nutritional counselling. | **Key Findings:** Significant decrease in weight, BMI, FM and FFM. Peak work rate significantly increased with progressive increase of minute ventilation due to similar rates of tidal volume and breathing frequency increase.  **Conclusion:** Improved exercise performance, reduced dyspnoea and to delay dynamic abdominal ribcage hyperinflation. These factors may contribute to improved exercise tolerance in otherwise healthy obese adolescents, therefore breaking the vicious cycle of inactivity and weight gain. | 57.14 |
| *Lopera | **Title:** Effect of water- versus land-based exercise training as a component of a multidisciplinary intervention program for overweight and obese adolescents  **Year:** 2016 **Country:** Brazil | **Aims/Purpose:** To compare the effects of water- versus land-based physical activity on body composition, physical fitness, and health-related quality of life  **Sample Size:** 151  **Participants:** Overweight or obese children aged 10-18 years | **Duration:** 16 weeks  **Physical Activity:** Exercised intensely in a playful and recreational way to increase the engagement. Intensity progressed with ability to perform activities Land-based group: Exercise sessions included (3 × 60 minutes/week): resistance training (sit-ups, bending arms, squats, and exercises with medicine balls); aerobic exercises (walking and running) and; collective games (such as basketball). Water-based group: Performed (3 × 60 minutes/week): immersed interval walking/running training; immersed interval running training with water-based equipment; resistance exercise with water-based equipment; swim exercises and diving to catch marbles; continuous recreational exercises (e.g., water polo). **Diet/Nutrition:**  Nutritional intervention (1-hour/week) to encourage reduced food consumption, healthy eating and discuss topics such as: food pyramid; energy density of food; micro and macronutrients; nutritional composition of food; portion control; strategies for eating out; strict vs. flexible dietary restraint; healthy food preparation; frequency of feeding.  **Healthy Lifestyle education:** Lectures (1 hour/week) on practice of exercise and its benefits, and to encourage adolescents to become more active outside the intervention environment (e.g., help their parents in domestic activities, avoid sedentary behaviours, etc.). Psychological intervention discussed the following topics: setting goals; self-observation and consequences (immediate, short and long-term); identification of feelings; body image; self-knowledge and analysis of internal events; self-motivation and self-control; interpersonal relationship (social skills). | **Key Findings:** Body weight was not significant. % body weight change was significantly (p < 0.05). % FM was significantly higher in intervention groups (land- and water-based) (p < 0.001) and significantly higher in the water-based compared with land-based p < 0.05). Physical (p < 0.001), social (p < 0.01) and psychosocial (p < 0.01) were significantly increased in the land-based group but only the physical dimension increased in the water-based group (p < 0.05).  **Conclusion:** This intervention improved body composition, physical fitness and health-related quality of life, regardless of the exercise mode. Future studies determining the long-term effects of both exercise modes are needed with focus on identify the potential benefits of the water-based intervention on cardiometabolic risk factors. | 57.14 |
| Lopes | **Title:** Effects of 12 weeks of combined training without caloric restriction on inflammatory markers in overweight girls  **Year:** 2016 **Country:** Brazil | **Aims/Purpose:** To investigate the effects of 12 weeks of moderate-to-high-intensity combined training without caloric restriction on pro- and anti-inflammatory markers  **Sample Size:** 33  **Participants:** Female children aged 13-17 years | **Duration:** 12 weeks  **Physical Activity:** The combined training protocol was composed of resistance training and aerobic training performed in the same session, 3 times a week, during approximately 60 minutes and divided into 3 stages, each consisting of 4 weeks of training. Resistance training was composed of six exercises (leg press, leg extension, leg curl, bench press, lateral pulldown and arm curl) and aerobic training consisted of walking/ running in an athletic track. The intensity of aerobic training according to VTI and respiratory compensation point was controlled by the speed achieved during the treadmill test. The participants were encouraged to perform the greatest number of repetitions when they came to the last set of each exercise, maintaining the same range of motion and execution speed previously determined. Workloads were increased by 1 kg for lower body and 0.5 kg for upper body for each repetition performed over the established training protocol in the last set of the last training session of the week. | **Key Findings:** Significant decrease in % BF, increase in FFM (p < 0.01). Homeostasis model assessment reduced and quantitative insulin sensitivity check index increased significantly (p< 0.05). Neutrophils count and neutrophils to lymphocytes ratio reduced (p< 0.05). Maximal strength on leg press, bench press and VO2max increased significantly (all, p< 0.001). Significant reduction in CRP (p<0.05), leptin (p< 0.05). No differences in tumour necrosis factor-alpha, IL-6, adiponectin, resistin and IL-10 post intervention. No significant differences in total energy consumption and macronutrients (p< 0.05).  **Conclusion:** Intervention improved inflammatory markers associated with obesity, such as and leptin, IR as well as BF and increase FFM, muscular strength and CRF in overweight adolescent girls, regardless of dietary intervention. | 71.43 |
| *Magnani Branco | **Title:** Effects of 2 Types of Resistance Training Models on Obese Adolescents' Body Composition, Cardiometabolic Risk, and Physical Fitness  **Year:** 2018 **Country:** Brazil | **Aims/Purpose:** To investigate the effects of 2 types of resistance training models in conjunction with interdisciplinary interventions to reduce BF and cardiometabolic risk, as well as to improve health-related physical fitness  **Sample Size:** 18  **Participants:** Obese male children | **Duration:** 12 weeks  **Physical Activity:** During the first 6 weeks, moderate intensity resistance exercises were performed, RPE 12-14. In the last 6 weeks, HIIT was used in an “all-out” mode (3 times/week) alternating in trainings A and B, in the form of a circuit, with the execution of 3 sets/session and a passive rest interval between the sets. One group was subjected to an intervention by means of activities that involved body mass and accessories, i.e., TRX (Total-body Resistance Exercise), elastics, medicine balls, and Swiss-balls. The other group executed the interventions on machines, bars, and dumbbells (weightlifting).  **Diet/Nutrition:** The main objective of the nutritional interventions (twice/week, 1 hour session) was to discuss topics such as the food pyramid, energy density of foods, importance of macronutrient and micronutrient and their correlation with health, nutritional composition of foods, differences between diet and light foods, importance of dietary re-education; ways to prepare healthy foods, and differences between natural, minimally processed, processed, and ultra-processed foods.   **Healthy Lifestyle Intervention:** The interventions provided several discussions among the participants regarding the central themes of the project (once/week, 1 hour sessions): such as anxiety control, self-esteem development, positive perceptions of body self-image and, above all, a process of behavioural change. The psychological aspects involved in the behavioural change process of an individual focus on his or her beliefs, skills, past experiences, motivation, and self-concept. | **Key Findings:** %BF (p = 0.004), WC (p=0.009) significantly reduced. Body mass, BMI, lean mass, bone mass, neck circumference, and blood pressure demonstrated no difference between groups (p>0.05). Maximal grip strength (p=0.005), VO2max (p=0.024) significantly increased.  **Conclusion:** The intervention was effective at reducing fat mass, BF, waist and hip circumferences, LDL-c, and TG, improving muscle strength and resistance, flexibility, and cardiorespiratory capacity. | 57.14 |
| Manley | **Title:** Self-efficacy, physical activity, and aerobic fitness in middle school children: examination of a pedometer intervention program  **Year:** 2014 **Country:** USA | **Aims/Purpose:** To determine whether a school-based pedometer intervention program would improve self-efficacy levels, physical activity, aerobic fitness, and body composition  **Sample Size:** 116  **Participants:** Children aged 11-13 years | **Duration:** 12 weeks  **Physical Activity:** During the health class, students participated in 10 minutes of physical activity. This activity was in addition to PE class or any other structured physical activity. The 10-minute physical activity was provided in a group setting and was led by the teacher. The activity consisted of student participation in MVPA beyond their usual activities in the form of walking or jogging, led by the PE teacher.  **Healthy Lifestyle Education:** Teachers provided encouragement to the students at the beginning of each day and education regarding the benefits of physical activity was provided in the health class. At the end of the day, teachers provided praise regarding the step counts that students received and continued to encourage increasing the number of steps. Students were encouraged to achieve and exceed the number of steps, if possible. | **Key Findings:** Non-significant improvements in mean baseline and post-intervention self-efficacy, aerobic fitness, and BMI. Increase in mean self-efficacy scores and mean aerobic fitness levels.  **Conclusion:** Although findings were not statistically significant, utilisation of a larger sample, collaborative school-based approach, and additional physical activity time have the potential to optimise outcomes. Further research focusing on community-based interventions that increase physical activity in this population is indicated. | 71.43 |
| Mardones | **Title:** Physical activity in the classroom to prevent childhood obesity: a pilot study in Santiago, Chile  **Year:** 2017 **Country:** Chile | **Aims/Purpose:** To test the suitability of the program for the students and teachers in the school as a way of increasing any form of physical activity within the classroom  **Sample Size:** 89  **Participants:** Children aged 6-7 years in first grade | **Duration:** 4 months  **Physical Activity:** The main objective of the study was to test the suitability of the program for the students and teachers in the school as a way of increasing any form of physical activity within the classroom. Adaptations were made to the TAKE10!® program activities in order to make them useful in this setting. It was necessary to adapt some of the programming to the specific local needs of the students in Chile. For example, local Chilean terms were used to modify the examples and instructions given on the cards, making them easier for the children to understand. Also, the lessons were modified to use phonetic sounds as opposed to letters for easier memorisation. | **Key Findings:** Height increased as expected. Modified variables of SBP and WC decreased.  **Conclusion:** Significant reduction in high WC ≥ 90th percentile, and in mean SBP. Anthropometry and hand grip strength were not modified. Weaknesses of this pilot study suggests further research with a larger sample and an experimental design is needed. | 85.71 |
| *Marild | **Title:** A controlled study of lifestyle treatment in primary care for children with obesity  **Year:** 2013 **Country:** Sweden | **Aims/Purpose:** To evaluate the efficacy of lifestyle treatment in primary care for children with obesity  **Sample Size:** 64  **Participants:** Obese children aged 9-13 years | **Duration:** 12 months  **Physical Activity:** A physiotherapist was engaged to highlight the importance of physical activity. Stimulate the child to reach the recommended duration of 60 minutes MVPA/day. Change transportation to and from school from passive to active (i.e. walking or cycling). Stimulate the child to participate in physical exercise lessons at school and to have 3 occasions each week with some kind of special training.   **Diet/Nutrition:** A paediatric nurse offered 8 visits and the dietitian offered 4 visits (individual and group meetings) with key messages including: eating breakfast; to eat at regular times; to eat meals together with the family; and to reduce processed sugar of any kind, especially in soft drinks. The nurse monitored the weight development and reinforced the diet-related messages.   **Healthy Lifestyle Education:** The nurse attempted to reduce inactivity, discuss the possibility of parents and children spending more time together, limit TV viewing, and highlight the importance of adequate sleep and sound bedtime routines. Reduce inactivity; a maximum of 3 hours in front of the television or computer was recommended. The physiotherapist provided telephone reminders between her scheduled meetings. | **Key Findings:** Mean BMI-z change reduction was significantly greater than the control. Change to follow-up for anthropometric data did not differ significantly. In the normal, overweight, and obese groups there was an increase in fasting insulin  **Conclusion:** Both 12-month lifestyle interventions were effective compared to control. No improvements in laboratory CVD or diabetes at follow-up. The favourable outcome of BMI is encouraging, but long-term follow-up is required for complete evaluation. | 71.43 |
| Martinez Vizcaino | **Title:** Assessment of an after-school physical activity program to prevent obesity among 9- to 10-year-old children: a cluster randomized trial  **Year:** 2008 **Country:** Spain | **Aims/Purpose:** To assess the impact of a physical activity program on obesity in primary school children  **Sample Size:** 1044  **Participants:** Children from 20 primary schools | **Duration:** 24 weeks  **Physical Activity:** It consisted of a non-competitive recreational physical activity program adapted to the children’s age and held after school at the school’s athletic facilities. The program consisted of 3x90-minute sessions per week for 24 weeks. The sessions included sports with alternative equipment (pogo sticks, frisbees, jumping balls, parachutes, and so on), cooperative games, dance and recreational athletics. Each 90-minute session included 15 minutes of stretching, 60 minutes of aerobic resistance and 15 minutes of muscular strength/resistance exercises. On average, these exercises required physical activity of moderate intensity throughout the 90 minutes of each session.  **Environment:** The sports instructors underwent a 2-day training program and a written plan of activities for each session was developed to ensure program standardisation in all 10 intervention schools. | **Key Findings:** No differences in BMI. Decrease in skinfold thickness in both boys (p<0.001) and girls (p<0.001), % BF in girls (p=0.02).   Intervention boys exhibited a decrease in apo B (p=0.03) and an increase in apo A-I l (p<0.001). No changes in TC, TG or blood pressure, but an increase in DBP (p=0.03).  **Conclusion:** Results demonstrated reduced adiposity, increased apo A-I, decreased apo B. Results also suggest that future programs should include more intense physical activity than in previous studies. | 71.43 |
| *Martinez-Lopez | **Title:** Intervention for Spanish overweight teenagers in PE lessons  **Year:** 2012 **Country:** Spain | **Aims/Purpose:** To determine the effect of a pedometer intervention on BF and BMI levels   **Sample Size:** 112  **Participants:** Overweight children from 5 secondary schools | **Duration:** 6 weeks  **Physical Activity:** The physical activity program consisted of at least 12000 and 10000 steps/day. To increase their motivation towards physical activity, this program enabled students to increase their final PE mark with up to 2 extra points according to their number of steps/day. Every day participants registered their personal number of steps/day into a record and sent it to their PE teacher via e-mail or SMS message every week. | **Key Findings:** Significantly reduced BMI (p < 0.05) with the difference observed in the number of steps/day between boys (12050) and girls (9566) was significant (p < 0.05).  **Conclusion:** Results demonstrated significant BMI reductions (p < 0.05) where the difference observed in the number of steps/day between boys (12050) and girls (9566) was significant in all measured periods (p < 0.05). | 100 |
| Matvienko | **Title:** The effects of a 4-week after-school program on motor skills and fitness of kindergarten and first-grade students  **Year:** 2010 **Country:** USA | **Aims/Purpose:** To examine the effects of an after-school NutriActive program on anthropometric measurements, motor skills, and fitness levels  **Sample Size:** 70  **Participants:** Children in kindergarten to first grade | **Duration:** 4 weeks  **Physical Activity:** Daily 15-minute morning walk and a 90- minute after-school session. Daily gym lessons focused on fitness-enhancing activities e.g. exercises and games to increase their upper- and lower-body strength and cardiovascular endurance (10 minutes); and motor skills (20 minutes) e.g. throwing for distance, kicking for accuracy, and various rope-jumping steps.  **Diet/Nutrition + Healthy Lifestyle Education:** The classroom curriculum covered body awareness, play safety and injury prevention, and nutrition. Body awareness/ safety lessons and nutrition lessons were taught on alternate days. | **Key Findings:** Significant improvement on some fitness and all motor skill tests. Skill levels emerged as predictors of CVF. Anthropometric measurements did not differ.  **Conclusion:** A short, intense program with emphasis on motor skill development rather than fitness may be effective for increasing overall levels of physical activity and fitness of young children. Such curriculum may be a suitable option for some schools and professionals who have limited time or resources. | 57.14 |
| Meyer | **Title:** Long-term effect of a school-based physical activity program (KISS) on fitness and adiposity in children: a cluster-randomized controlled trial  **Year:** 2014 **Country:** Netherlands | **Aims/Purpose:** To assess the 3-year follow- up of a cluster-randomized controlled school-based physical activity program over 9 month with beneficial immediate effects on BF, aerobic fitness and physical activity  **Sample Size:** 502  **Participants:** Children from 15 elementary schools | **Duration:** 3-year follow-up (9-month intervention)  **Physical Activity:** Children had 3 PE lessons per week (45 minutes each) given by the usual classroom teachers. The intervention group had 2 additional PE lessons (45 minutes each). The curriculum for all PE lessons for the intervention group was prepared by a team of expert PE teachers and the same curriculum aiming at increasing quality of PE and quantity of at least moderately intense physical activity was provided to all intervention classes. In addition, 3 to five short activity breaks (2 to five minutes each) were introduced every day during academic lessons, comprising motor skill tasks such as jumping or balancing on 1 leg. The children also received daily physical activity homework of about 10 minutes. | **Key Findings:** Significant higher aerobic fitness in the shuttle run. Continued to increase their fitness from post-intervention to the follow-up. Primary and secondary outcome variables were not significantly different. Skinfolds and WC at follow up were significantly lower in the 5th grade (p<0.03). Time spent in sports club was significantly higher (p = 0.022).  **Conclusion:** Apart from aerobic fitness, beneficial effects were not maintained post- intervention. | 85.71 |
| *Militao | **Title:** Effects of a recreational physical activity and healthy habits orientation program, using an illustrated diary, on the cardiovascular risk profile of overweight and obese schoolchildren: a pilot study in a public school in Brasilia, Federal District, Brazil  **Year:** 2013 **Country:** Brazil | **Aims/Purpose:** To evaluate the effects of a program of recreational physical activities on CVD risk   **Sample Size:** 34  **Participants:** Overweight children aged 9-11 years | **Duration:** 10 weeks  **Physical Activity:** Recreational physical activities and guidance on healthy habits (20 sessions, 60 minutes). Each session comprised 5 minutes of stretching, 40 minutes of basic motor recreational activities (running, jumping, and throwing), and 15 minutes recovery time.  **Diet/Nutrition + Healthy Lifestyle Education:** Each student received a journal containing pictures representing each weekday, and pages displaying various images of food, sedentary activities and active behaviour (colourful pictures = healthy habits; black/white pictures = unhealthy habits). Children were asked to paste pictures into their diary to represent the activities done and foods eaten.   **Family-Based:** The parents of the students received a note explaining the diary completion activity. | **Key Findings:** No significant differences in anthropometric parameters, biochemical parameters, blood pressure, or VO2max. Significant positive reductions in risk factors for CVD.   Healthy lifestyle guidance offered through the illustrative diary motivated the students to include recreational physical activities demonstrated with significant differences between post-intervention (p<0.01).  **Conclusion:** Results were effective in reducing risk factors for CVD and motivation to change unhealthy eating and physical activity habits. | 85.71 |
| Miller | **Title:** Can E-Gaming Be Useful for Achieving Recommended Levels of Moderate- to Vigorous-Intensity Physical Activity in Inner-City Children?  **Year:** 2013 **Country:** USA | **Aims/Purpose:** To compare the energy expenditure of e-gaming with a tethered videogame and traditional PE activities in meeting recommended levels of MVPA  **Sample Size:** 104  **Participants:** Children in 3^rd^-8^th^ grade | **Duration:** 1 year  **Physical Activity:** Participants completed 3 randomly ordered and supervised 20-minute bouts of DDR, "Orbis" and PE. A game such as DDR can induce a short bout of high intensity lower body exercise. "Orbis" can be played at a lower intensity but for a longer period of time. Traditional PE consists of students engaged primarily in basketball, dodge ball, obstacle courses, or double dutch jump roping. PE classes were 50 minutes in duration and included a warmup (5 minutes) and 2 core activity (35 minutes), followed by free play (10 minutes). | **Key Findings:** Sex differences in energy expenditure from PE approached statistical significance (p<0.03). No sex differences in energy expenditure from DDR or from "Orbis". No statistical differences in energy expenditure from DDR, "Orbis" or PE across BMI categories.  **Conclusion:** E-gaming may provide an effective adjunct to traditional PE activities in promoting MVPA – particularly in older girls, as they expended the lowest energy expenditure. | 71.43 |
| *Molina-Garcia | **Title:** Effects of Exercise on Plantar Pressure during Walking in Children with Overweight/Obesity  **Year:** 2019 **Country:** Spain | **Aims/Purpose:** To investigate the effect of a 13-week exercise program, based on “movement quality” and “multigames” work, on plantar pressure during walking   **Sample Size:** 70  **Participants:** Overweight and obese children aged 8-12.9 years | **Duration:** 13 weeks  **Physical Activity:** Group sessions (available Monday-Friday, attend > 3 per week, 90 minutes/session) were divided into 2 different parts: 30 minutes of “movement quality” work and 60 minutes of “multi-games”. The “movement quality” component had the aims of allowing children to acquire an awareness of analytical movement patterns (e.g., anterior and posterior pelvic tilt) and body posture (e.g., optimal spine position), to gain body segment mobility (e.g., hip flexion mobility) and stability (e.g., core stability), to gain muscular strength over a functional range of motion (e.g., bilateral lower limb push strength), and to learn basic exercise patterns (e.g., squat pattern). The “multi-games” component had the aims of allowing children to reach a MVPA minutes, to help them learn a wide range of fundamental movement skills (e.g., sprinting, hopping or throwing), and to make physical exercise more enjoyable. | **Key Findings:** Significantly smaller increase in plantar surface area (p=0.015), rearfoot surface area (p=0.054). Significantly greater increase in maximum force (p=0.012). No significant differences for remaining maximum force variables (all, p>0.05). Significantly reduced foot pain.  **Conclusion:** Results maintained total plantar pressure surface, increased the maximum force supported beneath the forefoot (specifically beneath the lateral and medial forefoot), led to positive functional changes in foot dynamics during walking in children with overweight/obesity. | 57.14 |
| Monsalves-Alvarez | **Title:** Motor skills and nutritional status outcomes from a physical activity intervention in short breaks on preschool children conducted by their educators: a pilot study  **Year:** 2015 **Country:** Chile | **Aims/Purpose:** To evaluate the results of a pilot intervention on motor skills and nutritional status in preschool children  **Sample Size:** 70  **Participants:** Children aged 4-5 years | **Duration:** 6 months  **Physical Activity:**  Classes (3 times/week, 45-minute session) included circuit activities (i.e. jumps, sprints, carrying medicinal balls, gallops and crawling’s. Tunnels, coordination scales cones, medicinal balls, ropes) were used in all the activities. to achieve more VPA and to avoid the inactivity | **Key Findings:** No significant differences in nutritional status (boys p=0.49, girls p=0.77). 12-meter run test found significant changes in boys (p = 0.002) and girls (p < 0.0001). Boys and girls (p<0.0001) significantly increased standing long jump.  **Conclusion:** This pilot study found an improvement in essential motor skills which are the first steps to increase MVPA and consequently, adequate energy expenditure and weight balance in children. | 71.43 |
| *Monteiro | **Title:** Concurrent and aerobic exercise training promote similar benefits in body composition and metabolic profiles in obese adolescents  **Year:** 2015  **Country:** Brazil | **Aims/Purpose:** To compare the effects aerobic and concurrent on body composition and metabolic profile   **Sample Size:** 32  **Participants:** Obese children aged 11-17 years | **Duration:** 20 weeks  **Physical Activity:** Concurrent training (3 times/week, 60 minute sessions) of 50% of resistance training time followed by 50% of the aerobic training. Aerobic training (3 times/week) with each session consisted of 50 minutes of walking/running. Resistance exercise (leg press, low rowing, bench press, squat rack, seated lat pull-down, leg curl, arm curl, seated chest fly, triceps, leg extension, sit up, and supine trunk extension) began with minimal loads and increased to an intensity of 55% 1RM for 2 weeks. The intensity was progressively increased every four weeks with the final intensity of 75% 1RM. | **Key Findings:** Concurrent (p=0.004) and the aerobic (p=0.0001) training resulted in significant decreases in % BF (p=0.004; p=0.0001) with no difference between groups. Changes in % android fat (p=0.001), intraabdominal adiposity tissue (p = 0.035), TG (p = 0.000), HDL-c (p = 0.030) and VLDL-c (p = 0.000) were different between groups.  **Conclusion:** Reduced % BF, improved lipid profiles, and other metabolic parameters suggest that the benefits of exercise can be achieved by either type of training. | 71.43 |
| *Morano | **Title:** A multi-modal training program to improve physical activity, physical fitness and perceived physical ability in obese children  **Year:** 2014 **Country:** Italy | **Aims/Purpose:** To assess the feasibility of a multimodal training program promoting changes in physical activity, fundamental motor skills and real and perceived physical abilities of obese children.   **Sample Size:** 41  **Participants:** Obese children aged 8-10 years | **Duration:** 8 months  **Physical Activity:** Fitness components (80 sessions, group/individual) included fundamental motor skills, muscle strength, power, aerobic fitness, speed, flexibility and prevention of injuries with gradual increase in volume and weekly number of sessions (50–60% maxHR).  **Diet/Nutrition:** Healthy eating behaviours (e.g. reduced consumption of high-fat foods, increased fruit and vegetable intake) were discussed with nutritional counselling  **Healthy Lifestyle Education + Family-Based:** Behavioural skills training was provided to participants and their families (1 day/week, 40 minutes) including review of physical activity diaries, food diaries, discussion of appropriate goals, self-monitoring incremental progress, social support and promoting awareness of the benefits of physical activity and fitness. | **Key Findings:** Weight, height and WC significantly increased. BMI, BMI z-score decreased. Significant time effects for behavioural, psychosocial outcomes, motor tests, with higher reporting physical activity and perceived physical ability scores.  **Conclusion:** Actual and perceived physical competence with gradual increase in the volume of activity improves lifelong exercise skills of obese children. | 85.71 |
| Muller | **Title:** Effect of a Multidimensional Physical Activity Intervention on BMI, Skinfolds and Fitness in South African Children: Results from a Cluster-Randomised Controlled Trial  **Year:** 2019 **Country:** Switzerland | **Aims/Purpose:** To investigate the dual disease burden among children in primary schools in disadvantaged neighbourhoods  **Sample Size:** 519  **Participants:** Children in fourth grade | **Duration:** 20 weeks  **Physical Activity:** The multidimensional physical activity intervention program consisted of: 2x 40-minute PE lessons/week (including 5-minute warmup/cool down); 1x 40-minute moving-to-music lesson/week; regular in-class physical activity breaks incorporated into the main school curriculum.   **Diet/Nutrition + Healthy Lifestyle Education:** Increased children’s awareness for communicable diseases and nutrition education to contribute to the awareness of healthy diet.  **Environment:** Enhancement of the school environment to be more physical activity friendly (e.g. installation of activity stations and a variety of painted games). | **Key Findings:** Significantly lower increase in mean BMI-z (p<0.001) and mean thickness of skinfolds (p=0.007). Changes in 20 m shuttle run test, VO2 max, self-reported physical activity were not significant (p>0.05). Frequency of overweight children slightly declined from 12.7% to 11.5%, while obesity increased from 5.0% to 6.5%.  **Conclusion:** Potential to lower the increase in BMI, skinfolds, despite no significant effects on CRF. | 71.43 |
| Naul | **Title:** 'Healthy children in sound communities' (HCSC/gkgk) - a Dutch-German community-based network project to counteract obesity and physical inactivity  **Year:** 2012 **Country:** Germany | **Aims/Purpose:** To report an integrated approach of a multicomponent intervention program to enhance an active lifestyle   **Sample Size:** 557  **Participants:** Children aged 6-10 years | **Duration:** 1 year  **PE + Family-Based + Environment:** 2x additional afternoon classes of movement and exercise to supplement the 3x PE lessons (total 60-90 minutes/day). The ‘walking bus’ (children walk to school along safe sidewalks accompanied by individual parents or other responsible persons) was introduced to provide an active school route between school and home.  **Diet/Nutrition + Healthy Lifestyle Education:** There were separate and joint cookery courses and ‘school fruit events’ for the schoolchildren and their parents. Schools organised and prepared a ‘healthy breakfast’. The curriculum for general and social studies includes 1 hour/week on the topics body, diet, lifestyle and health promotion, highlighting their joint contribution to healthy development. | **Key Findings:** Significant improvements for sit-ups (p<0.001), push-ups (p<0.001), 20-m run (p<0.001), standing broad jump (p<0.001), rapid alternations jumps (p<0.001), balance backwards (p<0.001), and 6- minute run (p<0.001).  **Conclusion:** Results of the German Cohort indicate possibility to counteract obesity and to increase levels of physical fitness and motor development by increasing endurance, coordination, velocity and force tasks. | 42.86 |
| *Nayak | **Title:** School Based Multicomponent Intervention for Obese Children in Udupi District, South India - A Randomized Controlled Trial  **Year:** 2016 **Country:** India | **Aims/Purpose:** To evaluate the effectiveness of multicomponent intervention on improving the lifestyle practices, reducing the BF and improving the self-esteem   **Sample Size:** 120  **Participants:** Obese children aged 10-16 years, BMI > 85th percentile | **Duration:** 6 months  **Physical Activity:** A video on aerobics was developed by the researcher in consultation with expert of aerobics. This was displayed in the television and children were trained to perform aerobic exercise.   **Healthy Lifestyle Education:** The areas covered in the informational booklet were meaning and causes, assessment, consequence and weight reduction strategies, lifestyle modification, importance of physical activity, healthy dietary practices, advise to reduce television viewing, to participate in recreational and other physical activities. Education of the children was done in the sequence of quiz; snake and ladder games and group discussion.   **Family-Based:** Education provided to parents on lifestyle modification. | **Key Findings:** The intervention observed a significant decrease in BMI (p=0.034). A statistically significant difference in the mean biceps (p<0. 001) and subscapular skinfold thickness (p<0. 001). Significantly improved the lifestyle practice scores (p<0.05).  **Conclusion:** Long-term aerobic exercise along with education of parents and children can significantly improve the lifestyle practices of children there by reducing BF of obese children. | 57.14 |
| *Nemet | **Title:** A combined dietary-physical activity intervention affects bone strength in obese children and adolescents  **Year:** 2006 **Country:** Israel | **Aims/Purpose:** To examine the effect of a combined nutritional-physical activity intervention on bone strength   **Sample Size:** 24  **Participants:** Obese children aged 6-16 years | **Duration:** 3 months  **Physical Activity:** The intervention (twice/week, 1 hour/session) varied in duration and intensity throughout the program and were designed as games. Endurance type activities accounted for most of the time spent in training (50% team sports, and 50% running games), with attention to coordination and flexibility skills. Subjects were instructed to add an extra 30–45 minute of walking/other weight bearing sport activities for at least once a week.   **Diet/Nutrition:** The participants met with the dietitian (6 times) with the first appointment (45–60 minutes) dedicated for acquaintance, learning about the reasons for childhood obesity, receiving information about food choices, dietary and cooking habits, understanding the motivation for weight loss, as well as trying to enrol the whole family to the “battle” against overweight. The following appointments (30–45 minutes) were devoted mainly for nutritional education (e.g., food pyramid, food choices, food labels, food preparation and cooking, eating habits). Subjects received a balanced hypocaloric diet. The diet consisted of 1200–2000 kcal or a caloric deficit of 30% from the reported intake, or 15% less of the estimated daily required intake.  **Healthy Lifestyle Education:**  Physicians, nutritionist and coaches encouraged reduced sedentary activities  **Family-Based:** Children 6-8 years**-**only the parents were invited for the first 2 meetings with the dietitian and the children joined the meetings thereafter. Children> 8 years were invited with both parents to all meetings. Pubertal subjects were invited to the first meeting with both parents, and then, alternately, the adolescent and his/her parents met the dietitians separately. | **Key Findings:** Group difference in body weight changes (p = 0.03); BMI percentile changes (p = 0.04); %BF changes (p = 0.03). Increased endurance (p = 0.045). Bone speed had a non-significant increase.  **Conclusion:** Results demonstrated significant differences in weight loss, BMI, BMI percentiles, BF, fitness and bone strength, thus emphasizing the importance of structured multi-disciplinary programs for the treatment of childhood and adolescence obesity. | 42.86 |
| Neumark-Sztainer | **Title:** New moves-preventing weight-related problems in adolescent girls a group-randomized study  **Year:** 2010 **Country:** USA | **Aims/Purpose:** To evaluate New Moves, a school-based program aimed at preventing weight-related problems in adolescent girls  **Sample Size:** 356  **Participants:** Female children from 12 high schools | **Duration:** 9 months  **Physical Activity:** New Moves program components included: The New Moves PE class, which incorporated nutrition and social support/self-empowerment sessions. Girls participated in physical activity (Be Fit) 4 days/week and nutrition (Be Fuelled) or social support/self-empowerment (Be Fab) classes 1 day/week. The intervention exposed the girls to fun activities (e.g., dance, hip hop, kickboxing) available in the community.  **Diet/Nutrition + Healthy Lifestyle Education:** The program targets socio-environmental factors (e.g., peer support), personal factors (e.g., body image), and behavioural factors (e.g., goal- setting), to bring about changes in physical activity, eating, and weight control behaviours. Eight behavioural objectives, targeted throughout the program, include: be more physically active; limit sedentary time; increase fruit and vegetable intake; limit sugar-sweetened beverages; eat breakfast every day; pay attention to portion sizes and your body’s signs of hunger and satiety; avoid unhealthy weight control behaviours; and focus on your positive traits.  **Environment:** Teachers received regular, ongoing support from New Moves staff throughout the program.  **Family-Based:** Minimal parent outreach activities. Six postcards were sent home to reinforce New Moves messages. On process surveys, which were mailed home and completed by parents. A parent– daughter retreat day at a local community centre that focused on New Moves messages. | **Key Findings:** Intervention girls increased their stage of change for physical activity (p=.039), physical activity goal-setting behaviours (p=.021) and self-efficacy to overcome barriers to physical activity (p=.003) and decrease total sedentary activity (p=.050). Improvements in dietary goal setting (p=.002), stage of change for fruit and vegetable intake (p=.002), regularity of breakfast eating (p=.028), portion control behaviours (p=.014) and for stage of change for portion control (p=.006). % engaging in unhealthy weight control behaviours decreased (p=.021), with significant improvements in body satisfaction (p=.045), perceived athletic competence (p=.044), and self-worth (.031).  **Conclusion:** Levels of satisfaction among girls and parents was effective in improving key weight-related attitudes and behaviours. It may be necessary to integrate New Moves into more comprehensive school-based interventions that involve ongoing educational efforts, changes in the school food and physical activity environments, and more intensive parent and community outreach activities. | 71.43 |
| *Nichols | **Title:** Short-term weight loss and exercise training effects on glucose-induced thermogenesis in obese adolescent males during hypocaloric feeding  **Year:** 1989 **Country:** USA | **Aims/Purpose:** To examine the effect of short-term weight loss and exercise training on the thermic effect of a glucose load  **Sample Size:** 17  **Participants:** Lean aged-matched and obese male children aged 13-16 years | **Duration:** 6 weeks  **Physical Activity:** Exercise training consisted of walking and/or jogging (3 days/week, 60 minutes/session), swimming (3 days/week, 30 minutes/session), aerobic dance and calisthenics (2 days/week, 30-45 minutes/session) and various sports (5 days/week, 30-45 minutes/session).  **Diet/Nutrition:** Obese subjects were maintained at a negative caloric balance of 1300-1500kcal/day. The diet, which consisted of balanced meals from the four food groups, contained 60 % carbohydrate, 20 % protein and 20 % fat. | **Key Findings:** Obese boys reduced weight by 12.1kg. Relative BF, lean body mass of obese boys decreased (p<0.01). Increased VO2 max.  **Conclusion:** Results demonstrate a reduction in body weight, fatness with improved aerobic capacity in morbidly obese adolescent males. The insulin response to an oral glucose load was reduced without change in glucose tolerance. However, the thermic response was to oral glucose was reduced. | 57.14 |
| *Nobre | **Title:** Twelve Weeks of Plyometric Training Improves Motor Performance of 7- to 9-Year-Old Boys Who Were Overweight/Obese: A Randomized Controlled Intervention  **Year:** 2017 **Country:** Brazil | **Aims/Purpose:** To examine the effects of 12 weeks of plyometric training on body composition, the performance in health-related physical fitness tests and gross motor  **Sample Size:** 59  **Participants:** Overweight and obese male children aged 7-9 years | **Duration:** 12 weeks  **Physical Activity:** Progressive plyometric training program (Tuesday/Thursday) was divided in 3 sections: warm-up (jogging at a self-selected comfortable pace followed by stretching for 3 minutes), training, and cool down. Training included level 1 included LIIT (lateral jump), level 2 (squat jump) and level 3 (increasing height). Plyometric exercises were performed in an explosive manner. | **Key Findings:** Increased body weight, height, and sitting height, but only trained group increase FFM. Groups showed small effect for FM and FFM for trained boys. Physical fitness and gross motor coordination demonstrated no differences. Improved handgrip strength, flexibility (sit and reach test), standing long jump, agility (square test), 1-mile run test, and abdominal strength (curl-ups).  **Conclusion:** No effects on reducing adiposity in obese boys, despite increased FF. Plyometric training improved gross motor coordination tests and therefore advantageous for children with overweight/obese. | 71.43 |
| Nogueira | **Title:** An in-school exercise intervention to enhance bone and reduce fat in girls: the CAPO Kids trial  **Year:** 2014 **Country:** Australia | **Aims/Purpose:** To determine the effect of a brief, simple, enjoyable, musculoskeletal- and fat-targeted exercise program on bone quality, fat and metabolic health  **Sample Size:** 151  **Participants:** Female children in fifth to sixth grade | **Duration:** 9 months  **Physical Activity:** Exercise bouts comprising 10 minutes of continuous HIIT. The program was largely based on capoeira, a Brazilian sport that combines martial arts with dance, and a broad range of continuous movements of medium to high impact, applied at varying speeds and directions including capoeira-specific movements (Ginga, kicks, and defence movements); jumps; hops; tuck jumps; jump squats; star jumps; cartwheels; and handstands. The number of repetitions was increased gradually and by the end of the year, a single session would include 150 jumps, 50 kicks, 30 to 40 movements in an inverted position (with upper extremity weight bearing). The majority of jumps were performed with maximal effort, while other movements had an emphasis on speed. | **Key Findings:** Improved resting HR (p<0.01), maximal vertical jump (p<0.001), estimated VO2 max (p<0.001), and WC (p<0.001). Gained significant weight (p<0.05). Increased calcaneal broadband ultrasound attenuation (p = 0.019).  **Conclusion:** Results demonstrated improved musculoskeletal, metabolic and fat outcomes in pre- and early-pubertal girls. The program was enjoyable, feasible to incorporate without disruption to academic schedule. | 71.43 |
| Nunez-Gaunaurd | **Title:** Health Outcomes of an Extracurricular Family-Based Intervention for Physical Activity in 3 Hispanic Male Children: A Case Series  **Year:** 2011 **Country:** USA | **Aims/Purpose:** To describe the feasibility and outcomes of a 12-week extracurricular family-based intervention designed to increase physical activity and led by physical therapists   **Sample Size:** 3  **Participants:** Male children aged 10-12 years | **Duration:** 12 weeks  **Physical Activity + Healthy Lifestyle Education + Diet/Nutrition + Family-Based + Environment:** The physical therapist-led KEBMM intervention targeted both children and their parents and focused on lifestyle modification related to children's food choices and physical activity. A guidebook was developed and consisted of 12 weekly sessions incorporating 10-15-minute lectures and group parent- and student- targeted activities related to nutrition and physical activity behaviours. Parents attended and participated in the same lectures and activities as their child. Behavioural modification, goal setting, use of existing social and family support structures and changed the home environment were strategies used to facilitate healthy behaviour changed related to physical activity and nutrition. | **Key Findings:** For the 3 subjects showed a substantial increase in the number of steps taken/day by the 12th week, with an average increase of 2509 steps or 33%. They also showed an average decrease of 8mmHg in resting SBP.  **Conclusion:** An extracurricular family-based intervention led by a physical therapist may be a feasible and effective strategy to improve health behaviours and fitness in children who are overweight | 71.43 |
| Nystrom | **Title:** Mobile-based intervention intended to stop obesity in preschool-aged children: the MINISTOP randomized controlled trial  **Year:** 2017 **Country:** Sweden | **Aims/Purpose:** To assess the effectiveness of a mobile health obesity prevention program on BF, dietary habits, and physical activity   **Sample Size:** 315  **Participants:** Children aged 4 years | **Duration:** 6 months  **Diet/Nutrition + Healthy Lifestyle Education + Family-Based:** The MINISTOP application was delivered via a smart-phone application to parents. It consisted a program of information and support grounded in social cognitive theory and behaviour change techniques. The intervention included 12 themes (healthy foods in general, breakfast, healthy small meals, physical activity and sedentary behaviour, candy and sweets, fruits and vegetables, drinks, eating between meals, fast food, sleep, foods outside the home, and foods at special occasions). | **Key Findings:** FM demonstrated no (p=0.922). Change in the intake of sweetened beverages (p=0.049). Improved 7-component composite score (p=0.021).  **Conclusion:** Although ineffective at improving FMI, positive effects for composite score comprised of FMI, dietary and physical activity variable and was highly accepted by parents. The effect of the intervention was more pronounced in higher FMI children. | 85.71 |
| Olvera | **Title:** BOUNCE: a community-based mother-daughter healthy lifestyle intervention for low-income Latino families  **Year:** 2010 **Country:** USA | **Aims/Purpose:** To assess the efficacy of the BOUNCE (Behaviour Opportunities Uniting Nutrition, Counselling, and Exercise) intervention for improving physical fitness and activity  **Sample Size:** 46  **Participants:** Latino mothers and their daughters (ages 7-12 years) | **Duration:** 12 weeks  **Physical Activity + Diet/Nutrition + Healthy Lifestyle Education:** The intervention involved a total of 3 weekly structured group aerobic (e.g., Salsa) or sport sessions (e.g., basketball) or free play recreational activities, 2 weekly nutrition sessions, and 1 weekly behavioural counselling session. Each session included 45 minutes of exercise and 45 minutes of either nutrition education or counselling. | **Key Findings:** Daughters exhibited higher levels of physical fitness (p<0.05). No significant difference MVPA of daughters. Although not statistically significant, trends indicate that daughters reported a reduced intake of high fat foods, sweetened beverages and an increase in consumption of fruits and vegetables.  **Conclusion:** Significant increase in physical fitness in daughters. The effect of the intervention on physical fitness is significant, despite no increase in physical. | 71.43 |
| *Paravidino | **Title:** Effect of Exercise Intensity on Spontaneous Physical Activity Energy Expenditure in Overweight Boys: A Crossover Study  **Year:** 2016 **Country:** Brazil | **Aims/Purpose:** To evaluate the effect of different exercise intensities on spontaneous physical activity energy expenditure  **Sample Size:** 24  **Participants:** Overweight male children aged 11-13 years | **Duration:** 6 days  **Physical Activity:** Sessions (60 minutes) were divided into 3 phases: warm-up (2.5 minutes), training (55 minutes) and cool-down (2.5 minutes). During the warm-up, subjects were instructed to start walking and progressively increase their pace until 64% maxHR. In the cool-down phase, subjects gradually reduce their pace to resting HR. Training consisted of 4x10 minutes walking at moderate intensity (64-76% maxHR), interspersed with 5 minutes of light walking (<64% maxHR). The VPA session consisted on 4x10 minutes running (77-95% maxHR), interspersed with 5 minutes of light walking (<64% maxHR). | **Key Findings:** Energy expenditure during the first hour following the intervention was significantly greater in the vigorous and moderate sessions (p<0.001) and greater in the vigorous compared to the moderate session (p = 0.04). Cumulative total energy expenditure for the 6 days following intervention was higher for the vigorous compared to moderate (p <0.001).  **Conclusion:** Results found a single aerobic exercise session modifies level of spontaneous physical activity among overweight adolescent. The practice of MVPA should be encouraged to promote a negative energy balance and facilitate weight loss. | 54.14 |
| *Park | **Title:** Effects of summer school participation and psychosocial outcomes on changes in body composition and physical fitness during summer break  **Year:** 2015 **Country:** USA | **Aims/Purpose:** To examine the effects of summer school participation and psychosocial outcomes on changes in body composition and physical fitness in underprivileged adolescents during the summer break  **Sample Size:** 138  **Participants:** Children aged 15-17 years | **Duration:** 5 weeks  **Physical Activity:** The summer school program was held five days a week during regular school hours (08:00-16:00) for 5 weeks. This was exactly the same schedule as the regular school days, with an hour of physical activity every day. During the physical activity period, students conducted aerobic exercises, resistive exercises with body weight (push-up, sit-up, etc) and a variety of games.   **Diet/Nutrition:** All summer school attendants were provided with breakfast (250-400 kcal) and lunch (700-900 kcal). | **Key Findings:** Non-attendants gained body weight (p=.003), % BF (p=.014). Step test results decreased significantly (p=.018). Push-up and sit-and-reach results did not change. Children who had parents that were educated beyond the university level (p<.05), lived with both parents (p=.0001), had day time adult caregivers during the summer break (p=.015), had higher house hold income (p=.002), or consumed fast-food less than once a week (p=.0001) gained less BF.  **Conclusion:** Results revealed that a long summer break may increase summer weight gain and decrease physical fitness indicating that a structured summer school program may be beneficial. Only for summer school non-attendants did a significant increase in body composition and decrease in physical fitness levels were affected by psychosocial outcomes. | 85.71 |
| Park | **Title:** Lifestyle plus exercise intervention improves metabolic syndrome markers without change in adiponectin in obese girls  **Year:** 2007 **Country:** Korea | **Aims/Purpose:** To investigate the effects of a 12-week lifestyle plus exercise intervention on adiponectin and metabolic syndrome markers   **Sample Size:** 44  **Participants:** Obese female children aged 13-15 years | **Duration:** 12 weeks  **Physical Activity:** The walking program was performed at an intensity of 55–75% maxHR 6 days/week and included 10 minutes of walking (Monday, Wednesday, Friday) in the morning and 30–40 minutes of walking in the afternoon (Tuesday, Thursday, Saturday) each day for 12 weeks.   **Healthy Lifestyle Education + Diet/Nutrition:** The behavioural modification program was composed of principles of self-monitoring, social skills, cognitive re-programming, control of stimuli, relapse prevention, changes in eating habits, positive reinforcement, dietetics, physiology, and physical activities. | **Key Findings:** No significant change in total daily caloric and macronutrient intakes, DBP, HDL-c, HbA1c, and adiponectin. Significant reduction in weight (p<0.001), BMI (p<0.001), %BF (p<0.001), WC (p<0.001), and waist-hip ratio (p<0.001), SBP (p=0.005), TC (p=0.014), LDL-c (p=0.007), TC/HDL-c (p=0.005), triacylglycerol (p=0.002), glucose (p=0.002), insulin (p = 0.016), IR (p=0.017), CRP (p=0.041), and leptin (p=0.032).  **Conclusion:** Results demonstrate improvements in BF, metabolic syndrome markers independent of adiponectin in obese girls. | 71.43 |
| Peralta | **Title:** Promoting healthy lifestyles among adolescent boys: the Fitness Improvement and Lifestyle Awareness Program RCT  **Year:** 2009 **Country:** Australia | **Aims/Purpose:** To assess the feasibility, acceptability, and potential efficacy of a school-based obesity prevention program   **Sample Size:** 33  **Participants:** Male children in seventh grade | **Duration:** 6 months  **Physical Activity:** The practical component of the intervention comprised of modified games and activities (60 minutes).  **Diet/Nutrition + Healthy Lifestyle Education:** Promoted physical activity through increasing self-esteem and -efficacy, reducing time spent in small screen recreation, decreasing sweetened beverage consumption, increasing fruit consumption and practice of self-regulatory behaviours.  **Family-Based:** Parents were emailed six newsletters throughout the program, which informed them of the program content, motivated them to help their son achieve their goals, suggested strategies to engage the entire family in healthy behaviours and created a stronger connection between parents and the school. | **Key Findings:** Boys had a smaller increase in BMI; greater reductions in WC, %BF, screen time on weekends; increased fitness, participation in total weekday physical activity.  **Conclusion:** This study contributes to the feasibility and acceptability data that addresses many of the shortcomings in secondary school-based trials. It shows that curriculum-based obesity prevention program is potentially efficacious | 85.71 |
| Prosper | **Title:** Healthy for Life/PE4ME: assessing an intervention targeting childhood obesity  **Year:** 2009 **Country:** USA | **Aims/Purpose:** To provide the first evaluation of the short-term outcomes of Healthy for Life and explore its effect on the contributory causes of childhood obesity  **Sample Size:** 1469  **Participants:** Children from 51 schools | **Duration:** 1 year  **Physical Activity:** The Sports, Play and Active Recreation for Kids (SPARK) curriculum is used for the preschool and elementary students with lessons incorporated into their school day (game cones, nylon rope, scooters, beach balls, foam and koosh balls, paddles, hoops, a 20-foot parachute and a crawl tunnel). For the middle and high school students, the program is offered as a graded PE class (body bars, dumb bells, jump ropes, lower body bands, exercise tubing and exercise balls) and the Fitness for Life textbook is used as a base for the curriculum and kickboxing workout twice/month.  **Diet/Nutrition + Healthy Lifestyle Education + Family-Based:** The nutrition education component of the program is incorporated into the PE class and a registered dietitian provides 2 hour-long nutrition presentations to the students after school. For preschool and elementary students, the presentations target their parents, offered in English and Spanish. The lessons and lectures are based on a variety of topics including the importance of eating breakfast daily, increasing vegetable and fruit consumption, methods on how to prepare cultural meals healthier, minimising the consumption of foods that are high in fat and sugar, eating out healthy, how to read a nutrition label, and portion distortion.  **Environment:** Each school is provided with the fitness equipment required for the program at no cost. PE curriculum adjustments made. | **Key Findings:** Mean decrease in BMI was significant (p=0.02). Improved self-esteem score for middle and high school students (p=0.014), score for overall healthy choices (p= 0.003). Fewer reported daily screen time of >2hours (p=0.001), more reported exercising >6x/week (p=0.001), more participants indicated that they eat junk food 0-2 times weekly (p=0.007), drinking soymilk, nonfat, 1% and 2% low fat milk (p= 0.001), more eat breakfast >6x/week (p<0.001).  **Conclusion:** Results indicate the program has potential to improve the health of underprivileged youth and provides a safe, accessible, no cost, and effective method to minimise some of the causal factors of obesity. | 71.43 |
| Puder | **Title:** Effect of multidimensional lifestyle intervention on fitness and adiposity in predominantly migrant preschool children (Ballabeina): cluster randomised controlled trial  **Year:** 2011 **Country:** Switzerland | **Aims/Purpose:** To test the effect of a multidimensional lifestyle intervention on aerobic fitness and adiposity in predominantly migrant preschool children  **Sample Size:** 652  **Participants:** Children from 40 public preschools | **Duration:** 1 year  **Physical Activity:** Program consisting of 4x45 minute sessions aimed to increase aerobic fitness and coordination skills. The sessions took place in or around the preschool classroom and once/week in the gym. A CD with specific music for most physical activity cards was created to increase pleasure.  **Diet/Nutrition + Healthy Lifestyle Intervention:**  There were 22 sessions on healthy nutrition, media use, and sleep. Positive and culturally independent nutritional messages were based on the five recommendations of the Swiss Society of Nutrition (“drink water,” “eat fruit and vegetables,” “eat regularly,” “make clever choices,” “turn your screen off when you eat”). Every other week children received a new funny physical activity or nutrition activity card based on the same themes and nutritional recommendations as the sessions and included specific exercises to be done at home. Healthy snacks during recess and healthy treats for anniversaries were promoted.  **Family-Based:** Parents participated in 3 interactive information and discussion evenings about promotion of physical activity, healthy food, limitation of TV use, and importance of sufficient sleep. Further support was provided by brochures, the physical activity or nutrition activity cards, and worksheets that children brought home.  **Environment:** Besides curricular changes, the built environment (climbing walls, hammocks, balls, cords, or stilts) in and around the preschool class was adapted to promote physical activity. Preschool classes were provided with a coloured poster of the “Ballabeina track” to be hung up on a classroom wall. | **Key Findings:** There was a significantly higher increase in aerobic fitness (20 m shuttle run test, running distance). No group difference in BMI and reductions in %BF, skinfolds and lower increases in WC. Improvement in motor agility but not in static or dynamic balance, with significant effects on physical activity, eating habits, and media use. No effect on prevalence of overweight, physical activity, sleep duration, cognitive abilities, and quality of life.  **Conclusion:** Results demonstrate no change in BMI but improvements in aerobic fitness, BF. Further dissemination of this program is needed as could contribute to reducing the burden of chronic diseases and of the health inequalities as a consequence of social inequities. | 85.71 |
| Quinn | **Title:** Introduction of active video gaming into the middle school curriculum as a school-based childhood obesity intervention  **Year:** 2013 **Country:** USA | **Aims/Purpose:** To incorporate active video gaming technology into PE classes as an intervention to increase adolescent activity  **Sample Size:** 86  **Participants:** Children in sixth, seventh, eighth grade | **Duration:** 6 weeks  **Physical Activity:** Just Dance software is an interactive dance video game by Ubisoft Entertainment that was released in 2009. Just Dance has songs from various genres and an on-screen silhouette dancer; up to four participants follow the on-screen dancer with gross motor arm and leg movements. During the active gaming fitness option, all students maintained a constant activity level for the 17 to 21 minutes of game play/dance. | **Key Findings:** Significantly more active in PE (p = .026) with slight increase but insignificant in time playing video games (p = .364).  Student attitude about PE class did not change (p=.720).  **Conclusion:** Results suggest positive modelling behaviours can encourage positive behaviours at home. Long-term efficacy within this school and expansion to other schools including alternate age groups warrants further investigation as no prior studies used Just Dance in the school setting as an active video game. | 71.43 |
| *Racil | **Title:** Plyometric exercise combined with high-intensity interval training improves metabolic abnormalities in young obese females more so than interval training alone  **Year:** 2016 **Country:** Tunisia | **Aims/Purpose:** To compare the effects of 12 weeks of plyometric exercise on anthropometric, biochemical and physical fitness data   **Sample Size:** 68  **Participants:** Obese females aged 16-18 years | **Duration:** 12 weeks  **Physical Activity:** HIIT groups (3 days/week) were composed of 2 blocks of six (in the first 4 weeks) or eight bouts of 30-second runs at 100% velocity at V̇O2max with 30 seconds of active recovery between bouts at 50% VO2max, on a 200-m outdoor track. The 2 blocks were separated by a 4-minute passive recovery period. The exercise intensities were increased by 5% VO2max at the start of each consecutive 4-week period. The plyometric group with HIIT group, had 2 blocks of 3 different plyometric exercises. Weeks 1-4: double-leg jump, medicine ball overhead throw, and medicine ball single-leg dip; weeks 5-8: hurdle hops, zig-zag jump drill, and medicine ball backward throw; weeks 9-12: single-leg cone hops, single-leg zig-zag drill, and medicine ball partner push pass were performed in each training session. Each plyometric exercise was maintained for 2 minutes (15 s of plyometric exercise vs 15 s of passive recovery). Passive recovery between each block (1 minute) and between each new exercise (30 seconds). All training sessions started with a standardised warm-up (10 minutes jogging 50% VO2max, 5 minutes dynamic stretching exercises, 5 accelerations over 20 m with 1 minute of recovery between) and ended with a cool-down (50% VO2max 10 minutes followed, 5 minutes of static stretching). | **Key Findings:** Significant decreases in body mass, BMI Z-score, BF and WC (p< 0.05). Plasma glucose and insulin concentrations were significantly decreased, resulting in a significant decrease in IR (p<0.05). Significant decrease in leptin; and increase in plasma adiponectin concentration. VO2max and squat jump and countermovement jump significantly increased (p < 0.05).  **Conclusion:**  HIIT appears to be an efficient strategy to combat obesity and may be recommended to improve adherence. Additional studies are nevertheless needed to elucidate the mechanisms of these specific adaptations. | 57.14 |
| *Racil | **Title:** Benefits of a regular vs irregular rhythm-based training program on physical fitness and motor skills in obese girls  **Year:** 2017 **Country:** Tunisia | **Aims/Purpose:** To examine the impact of a physical exercise program including rhythmic elements on physiological variables, motor skills  **Sample Size:** 36  **Participants:** Obese female children aged 9-12 years | **Duration:** 6 weeks  **Physical Activity:** The PE program (3 sessions/week) was composed of 3 parts: 15 minutes of warm-up, including: 8–10 different movements (10 minutes) and stretching (5 minutes); 40 minutes of exercises based on basic locomotor movements; 5 minutes of stretching. Performed exercises at different tempos and responded to various auditory stimuli. In 1/3 sessions, each participant was asked to transpose rhythmical structures presented on a sheet of paper to physical exercises. | **Key Findings:** HR recovery was significantly reduced (p=0.017). Significant decrease in SBP, DBP (p < 0.05) at rest and recovery period.  **Conclusion:** The main findings of support the hypothesis that transposing rhythmic structures into physical exercise improves cardiovascular parameters, physical performances (during jumping), and metabolic syndrome in obese girls. | 42.86 |
| *Rauber | **Title:** Effects of a physical activity and nutritional intervention in overweight and obese children through an educational and recreational camp  **Year:** 2018 **Country:** Brazil | **Aims/Purpose:** To investigate the impact of a health educational program for children composed of 5 days of camp and a 12-week follow up on the physical activity level, sedentary behaviour, anthropometric data and food intake  **Sample Size:** 12  **Participants:** Overweight an obese children aged 9-11 years | **Duration:** 5 days + 3 months  **Physical Activity + Diet/Nutrition + Healthy Lifestyle Education:** KIDS offered educational activities (through games and other non-sedentary strategies), daily thematic interventions and discussions on healthy nutrition, physical activities and stress management. Each theoretical activity had PE, psychology, nutrition and pedagogic content. Meals prepared by nutritionist contained fruit, vegetables, rice, beans, pasta, grains, roots, protein (from vegetable and animal sources) and a reduced amount of sugar and fat. The children collected fresh, green vegetables and fruit directly from the gardens at the farm. They were also encouraged to drink water during breaks between activities.   **Family-Based:** All families kept in touch with their children through phone calls > once/day. After 5 days at KIDS, the children and their parents participated in a planned intervention for 3 months. During this, the first 60 minutes were assigned to developing physical activity involving sports and street games. The second 60 minutes was an interdisciplinary educational activity. | **Key Findings:** Children lost nearly 1 kg of body weight and maintaining it over the 12 weeks. Skinfolds, WC and waist-height ratio, sedentary activity reduced significantly. Physical activity significantly increased. 58.4% started consuming a balanced diet, reduced soda, fast food.  **Conclusion:** Results demonstrate significant improvement in anthropometric data, nutritional habits, consumption of fruit and vegetables, consumption of sugar and soft drink plus improved physical activity reduced sedentary behaviour. | 71.43 |
| Reed | **Title:** Examining the impact of 45 minutes of daily PE on cognitive ability, fitness performance, and body composition of African American youth  **Year:** 2013  **Country:** USA | **Aims/Purpose:** To examine the effects of 45 minutes of daily PE on the cognitive ability, fitness performance and body composition   **Sample Size:** 470  **Participants:** Children from second to eighth grade | **Duration:** 8 months  **Physical Activity:** The PE requirements for elementary schools (1^st^- 5^th^ grade) is 45 minutes, 1 day/week; and 30 minutes, 1 day/week for kindergarteners. Middle schools (6^th^-8^th^ grade) provide 50 minutes/day for 1 semester. Elementary school PE content increased emphasis on fundamental skills. Middle school PE used a multiactivity sport theme curriculum.   **Healthy Lifestyle Education:** The experimental school implemented a comprehensive, multifaceted approach to education based on the premise that a ‘sound body nurtures a sound mind.’ | **Key Findings:** Experimental elementary school females significantly improved on all sections of FitnessgramR’s fitness battery but not elementary school males. Experimental elementary school females improved BMI (p= 0.0004). No significant changes in BMI percentiles for elementary school males, middle school females or middle school males.  **Conclusion:** 45 minutes of daily PE can perhaps increase cognitive ability, enhance fitness and decreasing prevalence of overweight and obese youth. | 28.57 |
| *Regaieg | **Title:** The effects of an exercise training program on body composition and aerobic capacity parameters in Tunisian obese children  **Year:** 2013 **Country:** Tunisia | **Aims/Purpose:** To determine whether a 16-week training program could have beneficial effects on body composition and aerobic capacity   **Sample Size:** 28  **Participants:** Obese children aged 12-14 years | **Duration:** 16 weeks  **Physical Activity:** Participated in habitual PE courses in school (60-minutes/week), with an additional 4 sessions/week of 60 minutes/session. This program comprised of warm up exercise (jog and stretch 5 minutes), main exercise (circuits, games and sports, 70-85% maxHR), and cool down exercise. Circuits: 1-2 sets; 3-4 exercises; period 5-10 minutes, rest between sets 1-2 minutes. Racing games (relay and other recreational activities): 1-2 games; 2-4 sets; 10-15 metres, 5-7 minutes, rest between sets 1-2 minutes. Sport games such as football, handball, and basketball: 20-25 minutes, 2 teams, 10-15 minutes, 2 minutes rest between sets. | **Key Findings:** Significant BMI reduction (p<0.001). A significant decrease in % BF mass (p< 0.001). Maximal aerobic capacity increased significantly. Significant increase for METmax (p<0.05). maxHR decreased in (p < 0.05).  **Conclusion:** Results demonstrated improvement in body composition and aerobic fitness and thus providing a sustainable and reproducible school and community approach for the management of children obesity. | 71.43 |
| Reznik | **Title:** A classroom-based physical activity intervention for urban kindergarten and first-grade students: a feasibility study  **Year:** 2015 **Country:** USA | **Aims/Purpose:** To evaluate the short-term impact of CHAM JAM on pedometer-determined physical activity levels during the school day  **Sample Size:** 988  **Participants:** Children from 4 elementary schools | **Duration:** 8 weeks  **Physical Activity:** The CHAM JAM (based on TAKE 10!) intervention is an audio CD (10-minute, 3 times/day, total 25 lessons) with aerobic activities. The academic skill in CHAM JAM was based on the material from the educational curriculum pertinent to each grade level and was developed in partnership with teaching experts. | **Key Findings:** Intervention took significantly greater steps than controls (p = 0.0048).  **Conclusion:** CHAM JAM is a promising program that increased physical activity and was generally well received by teachers and students. | 42.86 |
| *Rofey | **Title:** Cognitive-behavioural therapy for physical and emotional disturbances in adolescents with polycystic ovary syndrome: a pilot study  **Year:** 2009 **Country:** USA | **Aims/Purpose:** To evaluate the effectiveness of an enhanced cognitive–behavioural therapy for physical and emotional disturbances in adolescents with polycystic ovary syndrome  **Sample Size:** 12  **Participants:** Obese children aged 12-18 years with PCOS | **Duration:** 3 months  **Physical Activity:** Eight individual 45-60-minute sessions were conducted weekly. Subsequently, the 30–45-minute manualised treatment was delivered, and each session ended with 15–20 minutes of physical activity.   **Healthy Lifestyle Education:** Individual sessions focused on topics such as the comorbidity between physical illness and depression; scheduling behaviourally activating events; and engaging in positive thinking and cognitive restructuring.   **Family-Based:** 3x family-based sessions focusing on the family’s illness experience, the child’s ability to need share, and the maintenance of positive thinking. | **Key Findings:** Weight significantly decreased (p<.05). %BF did not change. Depressive symptoms significantly decreased. BMI improved with health related QOL improvement (p<.05). Menstrual regularity improved (p<.05). Sleep-related breathing improved (p<.05).  **Conclusion:** A manual-based cognitive behavioural therapy approach to treat depression in adolescents with PCOS and obesity appears to be promising | 57.14 |
| *Rowland | **Title:** Aerobic responses to walking training in sedentary adolescents  **Year:** 1991 **Country:** USA | **Aims/Purpose:** To investigate the effect of a school-based, alternative PE program of walking training on the aerobic fitness   **Sample Size:** 15  **Participants:** Obese children with mean age 15.7 years | **Duration:** 12 weeks  **Physical Activity:** Walking in 2 separate groups led by at least 1 instructor, 3 times weekly for 11 weeks. The program was conducted on measured routes (combination of flat and hilly terrain) outdoors except during inclement weather when subjects walked inside the school. Subjects were encouraged to walk briskly without resting | **Key Findings:** Mean values of height, weight and skinfold score did not change significantly. Significant improvement in mean VO2 max following walking training (p<0.05), while average treadmill endurance time increased by 2 minutes (23 % improvement).  **Conclusion:** In conclusion, walking training in sedentary, obese adolescents can be effective with small but significant improvements in aerobic fitness in the school setting. | 57.14 |
| *Ruebel | **Title:** Outcomes of a Family Based Pediatric Obesity Program - Preliminary Results  **Year:** 2011 **Country:** USA | **Aims/Purpose:** To determine which variables mediate changes in physical activity or energy intake behaviours and in turn impact the weight loss of the child  **Sample Size:** 22  **Participants:** Obese children aged 7-12 years and their families | **Duration:** 12 weeks  **Physical Activity + Healthy Lifestyle Education + Family-Based:** Consisted of (weekly sessions, 1.5-2-hour sessions) behaviour modification, nutrition education and family lifestyle physical activities (30 minutes). Families received information about ways to reduce sedentary activities and increase physical activity. Each family had individual consults with psychologist to determine barriers to healthy living and design strategies to meet weekly goals.  **Diet/Nutrition:** The weekly nutrition education sessions (30 minutes) with registered dietitian used The Stoplight Diet (low- (green), medium- (yellow), and high-calorie/fat (red) foods). Provided information on topics such as MyPyramid.gov, label reading, portion size, modifying recipes. | **Key Findings:** Children decreased %BF while FFM increased (p<0.05). Significant differences in body mass, BMI, BMI z-score, BF, CRF and energy intake. Parental variables that significantly changed in child’s behaviours included father’s change in nutrition knowledge which was associated with child’s decrease in fat intake (r = -0.92, p<0.05) and total energy intake (r = -0.85, p<0.05).  **Key Findings:** This highlights the importance of goal setting and behaviour change, the reduction of high calorie, high fat foods, involvement of the whole family in supporting healthy behaviours. | 42.86 |
| Sacchetti | **Title:** Effects of a 2-year school-based intervention of enhanced PE in the primary school  **Year:** 2013 **Country:** Italy | **Aims/Purpose:** To compare the physical activity habits, the physical performances, and BMI measurements in an intervention group and a control group   **Sample Size:** 521  **Participants:** Children aged 8-9 years in third grade | **Duration:** 2 years  **Physical Activity:** The type of physical activity (exercises, games, circuits, etc) was coordinated through monthly meetings, according to a previous standardised plan (>30 MVPA minutes/day). Twice weekly a further 50 minutes of PE was spent in the gym, according to the standard curriculum of PE. On average, then, during school hours, the children were engaged for around 45 minutes in MVPA. | **Key Findings:** Prevalence of overweight or obese children decreased in the intervention for both boys and girls. Practice of a sport did not vary significantly. Daily physical activity had significant differences (p<.05).  **Conclusion:** The school proved to be an ideal setting for promoting physical activity and achieving the required daily activity levels. | 71.43 |
| Sadeghi | **Title:** A 3-year multifaceted intervention to prevent obesity in children of Mexican-heritage  **Year:** 2019 **Country:** USA | **Aims/Purpose:** To reduce the rate of BMI growth among participating children   **Sample Size:** 700  **Participants:** Children aged 3-8 years with at least 1 Mexican-heritage parent | **Duration:** 3 years  **Physical Activity:** Implemented the Sport, Play and Active Recreation for Kids (SPARK) K-2 and Early Childhood PE curricula in grades K-2 and preschools, respectively (20–30-minute sessions/week). It included a health–fitness focus and activities that improved child motor/sport skills.   **Diet/Nutrition + Healthy Lifestyle Education + Family-Based:** Education delivered to parents at “family nights” (monthly) and to children in the school setting (22 classes) included an orientation, a healthy food cook-off event and various topics (shopping, healthy snacks, walking, eat more fruit and vegetables, reduce sugar-sweetened beverages, increase active play).   **Environment:** Provided monthly $25 fruit and vegetable voucher. | **Key Findings:** In boys across, BMI in the comparison group is higher than the intervention at the older end of the age spectrum. Obese girls in the comparison community have higher BMI relative to the intervention.  **Conclusion:** A community-based, multifaceted intervention was effective in slowing the BMI growth rate among obese Mexican-heritage children and normal weight boys older than 6 years old. The findings suggest that to enhance effectiveness of interventions, clinicians should work with a variety of community-based stakeholders, including parents, school officials, and policy makers. | 100 |
| Saenz | **Title:** The use of HR monitors in enabling children to self-regulate physical activity behaviors (Chapter 3-5)  **Year:** 2003 **Country:** USA | **Aims/Purpose:** To determine if the use of HR monitors can enable children to self-regulate physical activity behaviours  **Sample Size:** 34  **Participants:** Children in fourth grade | **Duration:** 1 year  **Physical Activity:** The study was conducted during the regular PE class. The fourth graders had 40 minutes of PE time in the first period after homeroom every other day. The participants were allowed to engage in any physical activity they chose, and many brought playground equipment such as soccer balls, footballs and jump ropes to the study area. | **Key Findings:** No statistically significant difference existed between the initial perceived exertion ratings and average HR. A statistically significant difference in mean number of minutes in the target HR zone.  **Conclusion:** Lack of correlation between perceived exertion and HR created uncertainty of the appropriateness of this scale's use in the current research. | 85.71 |
| Safdie | **Title:** Impact of a school-based intervention program on obesity risk factors in Mexican children  **Year:** 2013 **Country:** Mexico | **Aims/Purpose:** To evaluate the impact of an 18-month school obesity prevention intervention on health behaviours   **Sample Size:** 830  **Participants:** Children aged 9-11 years from public elementary schools | **Duration:** 2 years  **Physical Activity:** Specialised PE teachers were hired to teach 1 additional PE class per week for 4th and 5th grade students and to offer 15 to 20 minutes of moderate physical activity (calisthenics) referred to as “activation period” after the morning civics ceremony four days of each week. **Environment:** The aim of the nutrition intervention component was to improve the prevailing food environment by increasing availability of healthy food (fruits, vegetables, and non-fried dishes) and beverages (particularly water), by reducing the availability of energy-dense foods, and reducing the number of eating opportunities during the school day. Increased the availability of physical activity resources, by improving infrastructure and enhancing aesthetics. | **Key Findings:** Significant changes in the distribution of food among with an increase in the % of highly recommended food and by a reduction in the % of non-recommended food items (p<0.05) Availability of potable drinking water, increased significantly (p<0.05). Changes in MVPA during PE and recess were not significant. Steps taken during school significantly increased. Significant difference in BMI (p<0.001).  **Conclusion:** Statistically significant impacts in environmental and behavioural outcomes. Knowledge generated in this project can add to future design and implementation of the Mexican national guidelines in the school system to prevent childhood obesity. | 42.86 |
| Salcedo Aguilar | **Title:** Impact of an after-school physical activity program on obesity in children  **Year:** 2010 **Country:** Spain | **Aims/Purpose:** To assess the 2-year impact of the program on obesity, blood lipid levels, and blood pressure  **Sample Size:** 375  **Participants:** Children aged 9-10 years | **Duration:** 2 years  **Physical Activity:** MOVI (3 times/week, 90-minute sessions) was a non-competitive and recreational physical activity program, adapted to the children’s age and included sports with alternative equipment (pogo sticks, frisbees, jumping balls, small parachutes, etc), cooperative games, dance, and recreational athletics. The standard PE curriculum (3 hours/week, low-moderate intensity) was also provided.  **Environment:**  The sports instructors underwent a 2-day training program, and a written plan of activities for each session. A system of rewards was developed for adherence.  **Family-Based:** Parents were given a contact telephone number, through which they could obtain information, make suggestions, or ask about the program. | **Key Findings:** Lower frequency of overweight (p < .001), increased height (p < .001). No statistically significant differences in frequency of thinness and mean skinfold thickness. Lower TC (p<.001), apo B levels (p = .008). No significant changes in apo A-I, TG, or blood pressure, but SBP increased (p=.006) in boys. BMI (p=.02), %BF (p<.001), and SBP (p=.04) improved.  **Conclusion:** The physical activity program lowered the frequency of overweight in girls and reduced TC and apo B in both girls and boys. | 42.86 |
| Sallis | **Title:** Project SPARK. Effects of PE on adiposity in children  **Year:** 1993 **Country:** USA | **Aims/Purpose:** To determine the effects of a 2-year PE program on indicators of relative weight and adiposity in elementary school children  **Sample Size:** 740  **Participants:** Children in fourth grade | **Duration:** 3 years  **Physical Activity:** The program was (3x30-minute classes/week), divided into 3 segments: daily conditioning exercises (improving muscle strength and endurance); health fitness activities (promote CVF e.g. walk/run/jog, aerobic dance); sport skills (soccer, basketball, softball). Sessions had warm up and cool-down periods.   **Healthy Lifestyle Education:** Promoted physical activity outside of school by teaching behaviour change skills and self-management skills in weekly 30-minute classes.  **Environment:** The SPARK intervention consists of curricular and teacher in-service programs (the type and amount of equipment needed, class formations, and instructional cues) where detailed daily lessons plans were provided. Equipment was provided to all schools. | **Key Findings:** Boys and girls had no change in BMI and skinfold thickness.  **Conclusion:** Skinfolds and BMI improved despite no significant group differences in total skinfold. | 42.86 |
| Scheffler | **Title:** Does PE modify the body composition?--results of a longitudinal study of pre-school children  **Year:** 2007 **Country:** Berlin | **Aims/Purpose:** To analyse body composition, motor development and cardiovascular parameters of preschool children  **Sample Size:** 127  **Participants:** Children from 17 nursery schools | **Duration:** 24 months  **Physical Activity:** The playful-athletic exercise program was specially developed for this study. It includes 1-hour exercise training 3 times/week. The exercises should improve the pleasure of movement and train the motor basics like endurance, power, speed and skilfulness. | **Key Findings:** Increase in Frame Index but % BF decreased. Improved tested motor skills with significant lower values (p<0.001) in DBP. The duration of the program had a significantly positive effect on all tested motor skills.  **Conclusion:** Children should participate in a playful-athletic exercise program with special body movements to develop behaviour patterns in early childhood. Preventative programs continued during school increases the chance of decreasing the risks of overweight. | 42.86 |
| *Schwanke | **Title:** Differences in body posture, strength and flexibility in schoolchildren with overweight and obesity: A quasi-experimental study  **Year:** 2016 **Country:** Brazil | **Aims/Purpose:** To investigate whether a global exercise routine could result in positive changes in postural assessments and in the number of abdominal and flexibility exercise repetitions performed   **Sample Size:** 46  **Participants:** Overweight and obese children aged 7-17 years, BMI >85th percentile | **Duration:** 16 weeks  **Physical Activity:** The intervention program comprised (3x30-minute sessions/week, total 48 sessions) stretching and strengthening exercises using different equipment such as sleeping mats, tennis balls, volleyballs, basketballs, Swiss balls (Pilates), pool floats (spaghetti) and elastic bands, always with the supervision of a professional PE teacher and physical therapist. The sessions were scheduled to alternate between dynamic and isometric exercises for muscle strengthening and stretching. The same session was repeated twice per week so that subjects would be familiar with the activity. Initially, the exercises consisted of 3 series of repetitions with an interval of 15 seconds between them. For the dynamic exercises, the series included 10 repetitions, whereas the isometric exercises were maintained for 10 seconds. During the intervention period, an added degree-of-difficulty was incorporated into the implementation. | **Key Findings:** Significant differences in boys for trunk alignment (p=0.020), right knee angle (p=0.021). Significant differences in girls for cervical angle (p = 0.046) and flexibility (p = 0.003). Significant difference in thoracic angle for boys (p = 0.001) and girls (p = 0.010), abdominal strength/resistance in boys (p=0.016) and girls (p = 0.003).  **Conclusion:** A global postural exercise program resulted in changes of angle, distance measurements in postural assessments, improved abdominal strength and flexibility | 57.14 |
| *Seabra | **Title:** Effects of 6-month soccer and traditional physical activity programs on body composition, cardiometabolic risk factors, inflammatory, oxidative stress markers and cardiorespiratory fitness in obese boys  **Year:** 2016 **Country:** USA | **Aims/Purpose:** To investigate the effects of recreational soccer on body composition, cardiometabolic risk factors, inflammatory and oxidative markers, CRF and perceived psychological status  **Sample Size:** 88  **Participants:** Overweight and obese male children aged 8-12 years, BMI>2 SD | **Duration:** 6 months  **Physical Activity:** The soccer program (3 sessions/week, 60–90-minute/session) consisted of warm-up (10–20 minutes), technical drills and small-sided games (40–60 minutes), cool-down (10 minutes). The traditional activity program (3 sessions/week, 90 minutes/session) included a variety of exercise routines and included warm-up (10–20 minutes), generalised activities aimed at improving aerobic endurance, coordination, balance, flexibility and strength (40–60 minutes), cool-down (10 minutes). Aimed for 70-80% maxHR.  **Diet/Nutrition:** Nutritionist hosted 2x1 hour group sessions about energy balance and healthy diet. The energy balance session integrated basic concepts (energy intake, energy expenditure and body composition) and portion control based on the new Portuguese Food Wheel guide. | **Key Findings:** Decreased BMI z-score (p= 0.001), %BF (p=0.011), WC (p<0.001), and increased lean mass (p < 0.001), bone mineral content (p < 0.001) and bone mineral density z-score (p = 0.042). TC (p=0.006), HDL-c (p= 0.018), LDL-c (p < 0.001), TG (p=0.040) improved. Improved CRF (p=0.001), body image (p=0.003), self-esteem (p=0.032), quality of life (p=0.008), perceived themselves as more successful and physically competent (p = 0.011), and were more attracted to physical activity (p = 0.003).  **Conclusion:** Soccer and traditional physical activity interventions resulted in improvements in body composition, lipid profile, inflammatory and oxidative markers, CRF and perceived psychological status of obese boys. Given that soccer is a highly popular sport, inexpensive, accessible and easy to learn, it has the potential to be an effective tool for the prevention and reduction of childhood obesity | 85.71 |
| *Seltzer | **Title:** An effective weight control program in a public-school system  **Year:** 1970 **Country:** USA | **Aims/Purpose:** To present preliminary data on the effectiveness of the weight control program in reducing obesity  **Sample Size:** 350  **Participants:** Obese children in elementary and junior high school | **Duration:** 5 months  **Physical Activity:** Participated in special PE classes on the days where regular school PE was not scheduled, increasing physical activity to 5 times/week, 45-minute sessions. The emphasis was placed on team games (soccer, volleyball, and the like), competitive activities, and modified games, in addition to skill and drill activities. A variety of endurance activities were also included. Activities reported to be of special value were different types of dodge ball, "shipwrecked," chase, and relays. Subjects were encouraged to remain active during weekends and vacations by pursuing those sports or exercise activities.  **Diet/Nutrition +Family-Based:** The nutritional aspects of the project provided information (6 sessions) including daily food habits, functions, and sources, daily requirements of essential food nutrients, energy metabolism, and alterations in some individual caloric requirements. Individual conferences with the junior high school students and, at the elementary school level, with the students and their parents. Stress was laid on visual aids, wax food models to illustrate food portion sizes, pamphlets and booklets on good nutrition, posters, films, and comparison cards showing the vitamin, mineral, and caloric content of commonly accepted foods.  **Healthy Lifestyle Education:** Psychological support in terms of encouragement and motivation, a very important feature in weight control of youngsters, was supplied throughout the program. | **Key Findings:** Obese boys observed decreases in triceps skinfold thickness, body weight. No differences were found in the girls.  **Conclusion:** Not all youngsters benefited equally which is to be expected due to persistency of the effort expended, the extent of class participation, the degree of obesity, dietary habits etc. Future studies should explore why some youngsters showed no improvement whatsoever. | 28.57 |
| Sepulveda | **Title:** Feasibility, acceptability, and effectiveness of a multidisciplinary intervention in childhood obesity from primary care: Nutrition, physical activity, emotional regulation, and family **Year:** 2020 **Country:** Spain | **Aims/Purpose:** To examine the effectiveness of a family intervention vs. without family intervention  **Sample Size:** 49 **Participants:** Children aged 8-12 years with BMI >85th percentile | **Duration:** 6 months  **Physical Activity + Diet/Nutrition + Family-Based:** Parents and children attended the nutrition and physical activity sessions together. Parents were instructed and trained in skills to change habits and health commitment and to adapt parental democratic educational style to improve communication with their children. | **Key Findings:** Significant reduction in BMI-z, waist circumference and middle arm circumference. Significant reduction in sedentary activity and increase of LPA minutes. No change in MVPA. Significant reduction in anxiety symptomatology. No change in eating habits.  **Conclusion:** It is necessary to continue advancing in the study of psychological and family factors as factors of origin and maintenance of obesity. The inclusion of both factors in any intervention aimed at childhood obesity is paramount. | 57.10 |
| *Serra-Paya | **Title:** Effectiveness of a Multi-Component Intervention for Overweight and Obese Children (Nereu Program): A Randomized Controlled Trial  **Year:** 2015 **Country:** Spain | **Aims/Purpose:** To evaluate the effectiveness of the Nereu Program in improving anthropometric parameters, physical activity and sedentary behaviours, and dietary intake  **Sample Size:** 113  **Participants:** Overweight and obese children aged 6-12 years | **Duration:** 8 months  **Physical Activity:** Supervised physical activity sessions for children. The program offered 90 1-hour sessions (3 times/week).  **Healthy Lifestyle Education + Family-Based:** The aim was to enhance a physical activity behaviour, enjoy it during physical activity sessions, and learn healthy behavioural habits. The information content of the sessions was the same as their parents’ sessions. The 21 theoretical and practical group counselling sessions took place just once a week (60 minutes each) at the same time as the children’s sessions, giving the family the opportunity to exchange experiences and establish shared commitments later at home. 3 behaviour strategy sessions for both (children and parents) were designed to reinforce the acquisition of healthier physical activity and eating habits within the family. Extra family activities (e.g. a ski or water-park party) were organized on 3 weekends, in order to encourage and experience this more active behaviour. | **Key Findings:** BMI-z score had no significant difference between groups. Reduced sedentary behaviour, LPA and increased MVPA with significant differences (p<0.001). Increased their fruit consumption (p = 0.026), decreased their intake of processed meats (p = 0.118) sweetened juices and soft drinks (p = 0.047).  **Conclusion:** Significant improvements in activity and eating habits of participating children. Increase in MVPA and reduced sedentary behaviour time. Fruit consumption increased and daily servings of sugar-sweetened juices and soft drinks decreased. | 100 |
| Shofan | **Title:** A school-based program of physical activity may prevent obesity  **Year:** 2011 **Country:** Israel | **Aims/Purpose:** To evaluate the effects of a 2-year intervention program in elementary school on the prevention of obesity  **Sample Size:** 108  **Participants:** Children aged 9-11 years in fourth to sixth grade | **Duration:** 2 years  **Physical Activity:**  Double the PE hours. This activity was intense aerobic activity designed to increase the aerobic component by 50%.   **Diet/Nutrition + Family-Based:** During the 2 years of the program, the study group received eight nutritional education lessons. At regular parents’ meetings, which occurred once a month for 1 hour per session for 10 months a year, the paediatrician and the dietitian encouraged healthy dietary habits. | **Key Findings:** BMI percentile significantly reduced (p=0.0001). No increase physical exercise after school, no increase in fruit and vegetables consumption, no decrease in sedentary or fast food behaviour.  **Conclusion:** Early school intervention reduced overweight/obesity. | 57.14 |
| Siegrist | **Title:** Effects of a PE program on physical activity, fitness, and health in children: the JuvenTUM project  **Year:** 2013 **Country:** Germany | **Aims/Purpose:** To investigate the effects of a school-based prevention program on physical activity, fitness, and obesity  **Sample Size:** 724  **Participants:** Children from 8 primary schools | **Duration:** 1 year **Physical Activity:** The program (45 minutes/month) had 3 parts: 10-minute warm-up (running), 30-minute exercises to improve body awareness and self-esteem, and 5-minutes relaxation exercises. **Diet/Nutrition + Healthy Lifestyle Education + Family-Based:** The focus was on directly educating and encouraging children, and parents to live active and healthy lifestyles. Worksheets and homework assignments plus monthly newsletters intended to stimulate parent–child interaction and to support physical activity at home and in sports clubs. Parents participated in 2x training sessions in which they were given a program overview and practical instruction about health issues (3 hours total). They were informed about the development and course of the intervention program, received health-related journals, participated in practical instruction based on increasing motivation to spend more time being active with their children, and were asked to improve health behaviours with their family.  **Environment:** School environmental settings (e.g. the physical environment, organisation of school breaks, playing during school time, and sports facilities) were altered to promote more physical activity. | **Key Findings:** BMI and BMI-z score increased without significant differences. Reduction in WC (p<0.001). Number of days per week in 60 minutes physical activity, improved (p< 0.001). Inactive children (2 active day/week) increased physical activity (p< 0.001). Children who spent >2 hours/day consuming media reduced average time. Improved fitness (p < 0.001).  **Conclusion:** Health-related PE including supportive training of teachers, educating parents and children, and changes in the organisation and environment at schools, has the potential to increase physical activity independent of gender and socioeconomic status. The influence appears small but favourable in at risk, obese children. | 71.43 |
| Sigmund | **Title:** Does school-based physical activity decrease overweight and obesity in children aged 6-9 years? A two-year non-randomized longitudinal intervention study in the Czech Republic  **Year:** 2012 **Country:** Czech Republic | **Aims/Purpose:** To assess whether augmenting physical activity within the school setting resulted in increased daily physical activity and decreased overweight/obesity levels  **Sample Size:** 176  **Participants:** Children aged 6-9 years | **Duration:** 2 years  **Physical Activity:** The standard physical activity program comprised mandatory 2x45-minute PE lessons/week. The PE focussed on overall physical development through movement games (tag, games based on locomotion in rows/ circles, simplified versions of dodgeball/ football), simple gymnastic exercises (squats, sit-ups, bounces, etc.), and exercises with equipment e.g. ball, skipping rope, hoop, or benches. The physical activity intervention comprised: 1x20-minute recess with physical activity content; physical activity after-school (40-90 minutes); and an average of 2–3 short breaks/day (3–5 minutes) where physical activity could be carried out in the corridors. | **Key Findings:** Significant positive intervention effect found for steps/day and energy expenditure (all, p<0.0001). Level of school day physical activity in intervention children was higher (p<0.0001).  **Conclusion:** Reductions of overweight and obesity levels were observed about a year post intervention with a positive impact on leisure time physical activity on schooldays and weekends. Despite positive results the intervention did not achieve international levels of health maintaining physical activity. | 42.86 |
| *Silva | **Title:** Improvements on Cardiovascular Diseases Risk Factors in Obese Adolescents: A Randomized Exercise Intervention Study  **Year:** 2015 **Country:** Brazil | **Aims/Purpose:** To compare the effects of high- versus low-intensity aerobic training on CVD risk factors   **Sample Size:** 43  **Participants:** Obese children aged 13-18 years | **Duration:** 12 weeks  **Physical Activity:** Aerobic training on a treadmill, 3 times/week. HIIT at an intensity corresponding to the VTI, and LIIT at a speed 20% below the VTI. Exercise sessions were isocaloric, with energy expenditure set at 350 Kcal.  **Diet/Nutrition:** Nutritional group counselling (weekly, 1 hour) addressed healthy eating behaviour, the food pyramid, weight loss diets, diet versus reduced-calorie products, and recording energy intake and provided general nutrition information.   **Healthy Lifestyle intervention:** Psychologist group session (weekly, 1-hour) to discuss themes related to body image, eating disorders, relationship between food and feelings, family and social problems, mood, anxiety, and depression, psychological motivation. | **Key Findings:** Reduced weight, BMI, %BF, WC. LDL-c reduced in LIIT, without other blood or glucose variables. VO2max improved (p < .001).  **Conclusion:** HIIT does not promote additional improvements in CVD risk factors than LIIT. Further study is needed to determine the sustainability of these improvements. | 85.71 |
| *Silveira | **Title:** Effect of a pilot multi-component intervention on motor performance and metabolic risks in overweight/obese youth  **Year:** 2018 **Country:** Brazil | **Aims/Purpose:** To evaluate the effect of a multi-component intervention on motor performance and metabolic risk markers  **Sample Size:** 35  **Participants:** Overweight and obese children aged 7-13 years | **Duration:** 3 months  **Physical Activity:** The exercise sessions (60 minutes) included 10-minute warm-up (aerobic/anaerobic and recreational activities); 30-minute circuit training (6 stations, included activities that prioritised conditional and/or coordinative physical capacities); 15-minute pre-sports and recreational games; 5-minute resting activities.   **Diet/Nutrition:** The dietary intervention, designed by the nutritionist staff, consisted of 2 actions: 3 events (once/month) for dietary counselling with parents; and 3 daily dietary goals (frequency and quantity of adequate and inadequate food, and water consumption).  **Family-Based:** Exercise sessions were offered to all parents in order to encourage family support. This focused on improvement of 3 aspects: encouragement through provision of transportation to physical activity facilities; participation in physical activity with the children, when applicable; watching the children during physical activities. | **Key Findings:** Significant interaction improvements (p < 0.001) on motor performance, glycolytic and hepatic scores. Partial correlations and multiple linear regressions, and adjustments for MVPA, sedentary behaviour, showed negative associations (p < 0.05) between relative variance for motor performance score and relative variances for glycaemic), hepatic scores (β = -.424; 95%CI: −0.343: −0.022; and β = -.382 95%CI: −0.00: −0.009, respectively).  **Conclusion:** Results demonstrated moderate-to high effect on the motor performance, glycolytic, hepatic profiles of overweight/ obese youngsters. Changes in motor performance were inversely associated with metabolic profiles. | 71.43 |
| Singh | **Title:** Short-term effects of school-based weight gain prevention among adolescents  **Year:** 2007 **Country:** Netherlands | **Aims/Purpose:** To determine whether a multicomponent health promotion intervention for Dutch adolescents would be successful in influencing body composition and aerobic fitness  **Sample Size:** 978  **Participants:** Children with mean age 12.7 years | **Duration:** 8 months  **Healthy Lifestyle Education + Diet/Nutrition:** The intervention consisted of an individual component (i.e., an educational program covering 11 lessons for the courses of biology and PE). Behaviours targeted with regard to energy intake were consumption of sugar-containing beverages and high-energy snacks. Behaviours targeted with regard to energy output were physical activity and screen-viewing behaviour.   **Environment:** An environmental component included encouraging schools to offer additional PE classes and advice for schools on changes in and around school cafeterias | **Key Findings:** No significant intervention effects on BMI. Changes in waist-hip ratio differed significantly. Significantly favourable intervention effect on skinfolds was found in girls. Increased aerobic fitness was larger in the intervention group among both sexes, despite statistical insignificance.  **Conclusion:** Results observed improvements in sum of skinfolds and waist and hip circumference, emphasises the need for low-intensity population-wide studies. | 71.43 |
| Singh | **Title:** Dutch obesity intervention in teenagers: effectiveness of a school-based program on body composition and behavior  **Year:** 2009 **Country:** Netherlands | **Aims/Purpose:** To determine whether a multicomponent health promotion intervention would be successful in influencing body composition and dietary and physical activity behaviour   **Sample Size:** 1108  **Participants:** Children aged 12-14 years | **Duration:** 20 months  **Healthy Lifestyle Education:** The intervention consisted of an individual component (i.e., an educational program covering 11 lessons for the courses of biology and PE). Behaviours targeted with regard to energy intake were consumption of sugar-containing beverages and high-energy snacks. Behaviours targeted with regard to energy output were physical activity and screen-viewing behaviour.   **Environment:** An environmental component included encouraging schools to offer additional PE classes and advice for schools on changes in and around school cafeterias | **Key Findings:** Significant effect on biceps skinfold thickness among girls. For boys, WC and skinfold thickness was significantly improved. Consumption of sugar-containing beverages was significantly lower among boys and girls. Differences in screen-viewing behaviour with statistically significant differences in boys. No significant intervention effect on consumption of snacks or active commuting to school was found.  **Conclusion:** Beneficial effects on the sum of skinfold thickness in girls and consumption of sugar-containing beverages in both sexes in both the short and long term. | 57.14 |
| Slawta | **Title:** Promoting healthy lifestyles in children: a pilot program of be a fit kid  **Year:** 2008 **Country:** USA | **Aims/Purpose:** To describe the Be A Fit Kid pilot intervention and offer suggestions for ways in which universities and communities can implement health promotion programs   **Sample Size:** 75  **Participants:** Children aged 6-12 years | **Duration:** 12 weeks  **Physical Activity:** The physical activity component of the program emphasised CVF, flexibility, muscular strength, and bone development through running, jumping, yoga, and strength exercises. Began with lap running and running drills. Following the children’s running activity was strength training activities included lunges, squats, arm curls, chin-ups, sit-ups, and arm-hang; jumping activity involved jumping 30 to 40 times off 1½ foot blocks to the floor; and yoga involved several yoga poses and stretches. Hiking and ice-skating field trips were planned.   **Diet/Nutrition:** The nutritional aspect followed the physical activity component and focused on current dietary guidelines (diet rich in vegetables, fruits, unsaturated fats, and whole grains, and low in saturated fat and sugar). A variety of foods were distributed to the children each session for them to sample. A field trip to a local supermarket took place each term. Subjects were rewarded with healthy rewards.  **Family-Based:** Parents attended an initiation lecture prior to the start of the program that covered nutrition and physical activity principles. Parents were asked to participate in the program by reading the nutrition information sent home and contributing a healthy snack for the children to sample toward the end of the program. Every child received a healthy raffle prize each week if parents signed a form indicating that they reviewed the weekly nutrition material. | **Key Findings:** Significant improvements in all fitness measures, all body composition measures, nutrition knowledge, and some dietary habits. Significant reductions in HDL-C. Significant improvements in TC, TG. No significant changes in LDL-C levels. 75% of children increased intake of vegetables, fruits, whole grains, healthy fats, and water, and decreased cheese, red meat, candy, and soda. Positive dietary habits were maintained by majority of children 6 months post-intervention.  **Conclusion:** Such programs have the potential to address the needs of children receiving insufficient health and PE education. Future studies should explore expanding regular school day throughout southern Oregon communities | 85.71 |
| Smith | **Title:** Intervention effects and mediators of well-being in a school-based physical activity program for adolescents: The ‘Resistance Training for Teens’ cluster RCT  **Year:** 2018 **Country:** Australia | **Aims/Purpose:** To examine the impact of a school-based physical activity intervention on adolescents' self-esteem and subjective well-being  **Sample Size:** 508  **Participants:** Children in ninth grade from 16 schools | **Duration:** 10 weeks  **Physical Activity:** Focussed on the delivery of resistance training (bodyweight, resistance bands) and other ‘lifelong’ health and fitness activities (e.g., yoga, boxing, skipping, circuits). The program consisted of PE (once/week); an elective subject known as Physical Activity and Sport Studies (10 x ∼90 mins); lunch-time physical activity sessions (5×20 mins).  **Healthy Lifestyle Education:** Intervention components include: introductory seminar for students (1×30 mins); a web based smartphone application (10 weeks).   **Environment:** The intervention included teacher professional learning, provision of resources and physical activity session observation and feedback. | **Key Findings:** Insignificant improvement in MVPA (p=.78) or % BF (p=.11). Muscular fitness approached significance (p=.07). Among the overweight/obese a statistically significant intervention effect for % BF (p=.006), muscular fitness (p=.03).  **Conclusion:** Resistance training movement skill development had improvements in body composition, muscular fitness in adolescent males. Future research should explore the contribution of motivational factors as well as other lifelong activities. | 71.43 |
| Smith | **Title:** Mediating effects of resistance training skill competency on health-related fitness and physical activity: the ATLAS cluster randomised controlled trial  **Year:** 2016 **Country:** Australia | **Aims/Purpose:** To examine the mediating effect of resistance training skill competency on % BF, muscular fitness and physical activity   **Sample Size:** 361  **Participants:** Male children from 14 secondary schools | **Duration:** 8 months  **Physical Activity:** During the school sport sessions, students participated in a range of activities including resistance training (elastic band, body weight), HIIT, strength and aerobic-based games and modified ball games.  **Diet/Nutrition + Healthy Lifestyle Education:** Aimed to improve weight-related behaviours (i.e., screen-time, physical activity and sugared beverage consumption) and to increase boys’ perceived competence and self-efficacy for resistance training exercises  **Environment + Family-Based:** Seminars for students, provision of fitness equipment to schools, a smartphone application and website, pedometers for self-monitoring, parental strategies for reducing screen-time, lunch-time mentoring sessions and face-to-face activity sessions. | **Key Findings:** Intervention effects for self-esteem (p=.194) and subjective well-being (p=.509) were not statistically significant. No significant intervention effects for perceived fitness, intrinsic and identified regulations (all, p > .05). Statistically significant effect for resistance training self-efficacy (p < .001).  **Conclusion:** Resistance training did not have an overall impact on adolescents' self-esteem and subjective well-being, Future research should continue to examine the mechanisms that explain the relationship between physical activity and mental health using high quality experimental trials. | 71.43 |
| Sollerhed | **Title:** Physical benefits of expanded PE in primary school: findings from a 3-year intervention study in Sweden  **Year:** 2008 **Country:** Sweden | **Aims/Purpose:** To assess whether a school-based program with expanded PE lessons was effective in increasing children’s physical capacity and in preventing excessive weight gain in children  **Sample Size:** 132  **Participants:** Children aged 6-9 years | **Duration:** 3 years  **Physical Activity:** The intervention included an increase of allocated time for PE. The time was expanded from 1 or 2 lessons a week to four lessons, with every lesson being guaranteed to last for 40 minutes. On the 5th day, classes had outdoor physical activities with their classroom teacher for about 1 hour. Obese children had the possibility to have 1 extra voluntary lesson a week, with special attention paid to motor skills and self-esteem. | **Key Findings:** Significance in physical performance (p=0.003), as well as in endurance performance (p<0.001), motor skill performance (p=0.010). BMI changes were significantly better (p=0.033).  **Conclusion:** When PE was extended, this increase possibilities to increase children’s physical capacities. | 85.71 |
| *Son | **Title:** Combined exercise training reduces blood pressure, arterial stiffness, and insulin resistance in obese prehypertensive adolescent girls  **Year:** 2017 **Country:** USA | **Aims/Purpose:** To examine the impact of 12 weeks of combined resistance and aerobic exercise on arterial stiffness, blood pressure, blood nitrite/nitrate, ET-1 levels, homeostasis model assessment for IR and body composition   **Sample Size:** 40  **Participants:** Prehypertensive obese female children | **Duration:** 12 weeks  **Physical Activity:** Participants trained using combined resistance and aerobic exercise 3 days/week, 60 minutes/day. This combined resistance and aerobic exercise program was divided into warm-up (5 minutes), the main exercise (30 minutes of various exercises and 20 minutes of playing badminton), and cool-down (5 minutes). The warm-up and cool-down included static stretching. Combined resistance and aerobic exercise consisted of seven exercises (left- and right-side stroke, 1 line 1 jump, cross-jump, right and left jump, box jump, weighted squat jump, and jump rope) and badminton. Intensity of the exercise was gradually increased from 40–50% HHR and RPE 11–12 within the first 1–4 weeks to 60–70% HRR and RPE 15–16 in 9–12 weeks. | **Key Findings:** SBP was significantly reduced (p < 0.05). DBP was not significantly. Total nitrite/nitrate levels were significantly increased (p < 0.05) No significant difference in height, weight, or BMI. Significantly decreased %BF (p< 0.05) and increased %MM (p< 0.05)  **Conclusion:** Results observed included reduced blood pressure, arterial stiffness, IR, and %BF, decreases in abdominal adiposity, as well as an increase in nitrite/nitrate levels. Regular training can be an efficient precaution for reducing the risks of future obesity-related metabolic complications and CVD in obese adolescent girls. | 71.43 |
| *Song | **Title:** Effects of 12 weeks of aerobic exercise on body composition and vascular compliance in obese boys  **Year:** 2012 **Country:** USA | **Aims/Purpose:** To evaluate the impact of 12 weeks of air board exercise on CRF and vascular compliance and reduce % BF   **Sample Size:** 22  **Participants:** Obese male children | **Duration:** 12 weeks  **Physical Activity:** Supervised exercise training was performed on the Equbic air board, a square, flexible board that has an air spring attached to each corner. The air springs are designed as shock absorbers to reduce ground impact forces. Each training session involved group aerobic activity that consisted of:10 minutes warm up (joint rotations, muscle stretching and body twisting); 30 minutes of aerobic exercise consisting of walking, jogging, jumping, skipping, hoping, pumping, knee kicking, and sprinting equally distributed over this time period; and 10 additional minutes of joint rotations, muscle stretching and body twisting. Each exercise session was supervised and pre-programmed on a DVD and projected on a TV screen. All training was conducted after school hours on the school campus. | **Key Findings:** Decreases in skinfold thickness, BMI, suprailiac skinfold. Improvements in predicted VO2max Improvements in 20 m multistage endurance performance (p<0.05)/  **Conclusion:** Results observed improvements in CRF, vascular compliance, % BF, FM and BMI in young boys; suggesting air board aerobics provides an effective and convenient mode of exercise training. | 28.57 |
| *Southam | **Title:** A summer day camp approach to adolescent weight loss  **Year:** 1984 **Country:** USA | **Aims/Purpose:** To investigate a program offered during the summer where a multifaceted format for behavioural learning and practice of eating and exercise patterns with peer support and parental involvement was applied  **Sample Size:** 25  **Participants:** Overweight children | **Duration:** 4 weeks  **Physical Activity:** Subjects went to Stanford University 4 times/week. The aerobic exercise portion provided 45 minutes of activity. This session improved general physical condition. Tennis and swimming (45 minutes, alternate days) were chosen for leisure activity.   **Diet/Nutrition:** Practiced new eating habits during lunchtime which included a cafeteria, sack lunches, catered box lunches, catered buffets, and student-prepared potluck buffets  **Healthy Lifestyle Education:** 1 hour/day group sessions to discuss the change of eating and exercise behaviours, restricting the cues associated with eating, changing eating style and environment, planning for eating in social situations.  **Family-Based:** Parent seminars were conducted once/week. | **Key Findings:** % Overweight and skinfold thickness declined. The Knowledge Test measuring knowledge on basic nutrition and weight loss improved (p<0.01). Mean score on the Self-Esteem Scale had risen 4.9 from 4.5.  **Conclusion:** An intensive program of eating and exercise habit instruction, practice and monitoring in the home setting, may provide benefits to more traditional approaches to adolescent weight loss. | 85.71 |
| *Speaker | **Title:** Body size estimation and locus of control in obese adolescent boys undergoing weight reduction  **Year:** 1983 **Country:** USA | **Aims/Purpose:** To examine the relationships between body size employing a technique of body size estimation   **Sample Size:** 18  **Participants:** Obese male children aged 12-14 years | **Duration:** 7 weeks  **Physical Activity:** The camp program emphasised VPA. Each boy participated in at least six supervised hours of exercise /day e.g. jogging, swimming, hiking, bicycling, calisthenics, weightlifting and team sports such as baseball, basketball and soccer.  **Diet/Nutrition:**  Restriction of 1200kcal/day | **Key Findings:** Weight and BF content decreased. Lean body mass was unchanged. Physical fitness levels and/or motivation increased during camp: 1.5 mile run time improved and subjects saw themselves as thinner than they actually were.  **Conclusion:** This shift toward more internal locus of control could have positive implications for obesity and treatment and weight maintenance. | 85.71 |
| *Springer | **Title:** Weight watchers program for trainables  **Year:** 1973 **Country:** USA | **Aims/Purpose:** To describe an interdisciplinary effort to reduce or maintain the weight of 14 overweight trainables  **Sample Size:** 14  **Participants:** Overweight children from special education | **Duration:** 1 year  **Physical Activity:** The children's program consisted of exercises directed by the physical educator. Toe touch, side bends, sit ups, knee bends, leg lifts, body twists, stomach rockers and push ups were recommended.  **Diet/Nutrition:** The young adults had an opportunity to learn how to plan menus that were low in calories. With the help of the volunteer nutritionist, the teacher discussed with the students the main food groups that should be included in a meal, with appropriate stress made on the quantity. | **Key Findings:** Total weight gained and lost was practically the same. Important factors in the older children's weight loss include recognition of fattening foods, their willingness to cooperate and parental support/encouragement.  **Conclusion:** Parents should be actively involved in lunch time activities or cooking classes. Although there is still a great deal of improvement to be made in the program, nutrition education is the most stable means to permanent weight control. | 71.43 |
| *Steinberg | **Title:** The effect of a weight management program on postural balance in obese children  **Year:** 2013 **Country:** Israel | **Aims/Purpose:** To investigate whether obese children improve their balance and postural performance following a 6-month-weight management program  **Sample Size:** 29  **Participants:** Obese children aged 6-14 years | **Duration:** 6 months  **Physical Activity:** Training session (twice/week, 1 hour each) had 10-minute warm up, stretching and flexibility exercises. Strengthening, balance, agility and coordination exercises were followed by distance running to improve the children's aerobic endurance. The activities varied in duration and intensity. Endurance-type activities accounted for most of the time spent in training (50% team sports and 50% running games), with attention given, as noted, to coordination and flexibility skills. Subjects were instructed to add an extra 30 to 45 minutes of walking or other weight-bearing sport activities at least once per week.   **Diet/Nutrition:** The participants met with the dietitian (six times) for acquaintance, learning the reasons for childhood obesity, receiving information about food choices, dietary and cooking habits, understanding the motivation for weight loss, trying to enrol the whole family to the “battle” against overweight and nutritional education. Children received a balanced hypocaloric diet.  **Healthy Lifestyle Education:**  Subjects were encouraged throughout the program, by the staff, to reduce sedentary activities (e.g., to reduce television viewing and video game use, to use stairs instead of elevators, and to play outside instead of inside). | **Key Findings:** Significant increase in height and decrease in BMI percentile (p<.05). Interaction between BMI percentile differences and balance indicates improved balance, reduced vestibular stress/disturbances with weight loss.  **Conclusion:** The results suggest greater improvement in older male adolescents. Childhood obesity exercise programs should focus on balance to minimise the risk of falls. | 71.43 |
| *Steinberg | **Title:** Effects of a Program for Improving Biomechanical Characteristics During Walking and Running in Children Who Are Obese  **Year:** 2017 **Country:** Israel | **Aims/Purpose:** To investigate the influence of a weight-reduction program with locomotion-emphasis on improving biomechanical characteristics  **Sample Size:** 10  **Participants:** Obese children aged 7-12 years from fourth to fifth grade | **Duration:** 6 months  **Physical Activity:** Training session (twice/week, 1 hour each) had 10-minute warm up, stretching and flexibility exercises. Strengthening, balance, agility and coordination exercises were followed by distance running to improve the children's aerobic endurance. The locomotion-emphasis program had additional exercises aimed at improving gait.   **Diet/Nutrition + Family-Based:** The participants and parents met with the dietitian (6 times) to become acquainted with each other; learn the reasons for childhood obesity; receive information about food choices and dietary and cooking habits; understand the motivation for weight loss; receive nutritional education and to enrol in the "battle" against overweight. | **Key Findings:** Training program subjects significantly reduced their mass, but the locomotion-emphasis program and controls significantly increased their mass (p<0.05). Significant differences indicating training program subjects had significantly higher foot pressure values (p<0.05). Significant difference in temporal parameters indicating that locomotion-emphasis subjects had significantly lower values.  **Conclusion:** We found evidence to support the beneficial effects of combined dietary and physical activity/locomotion-emphasis exercises in the movement characteristics of children who are overweight | 71.43 |
| *Sung | **Title:** The effects of a 12-week jump rope exercise program on abdominal adiposity, vasoactive substances, inflammation, and vascular function in adolescent girls with prehypertension  **Year:** 2019 **Country:** Germany | **Aims/Purpose:** To examine the effects of a jump rope on cardiovascular risk factors, including body composition, vasoactive substances, inflammation, and vascular function   **Sample Size:** 40  **Participants:** Prehypertensive female children aged 14-16 years, BMI >95^th^ percentile | **Duration:** 12 weeks  **Physical Activity:** Participated in an exercise program (50 minutes/day, 5 times/week) that consisted of jump rope variations. Exercise was performed at 2:00 PM (± 1 h) Monday-Friday. This program was divided into a warm-up (5 minutes), the main exercise session (40 minutes of rope jumping variations), and a cool-down (5 minutes). Both the warm-up and the cool-down consisted of stretching, walking, and jogging. There were seven main rope jumping exercises, including 1 line 2 jump, jumping feet together, running jumping, open side jump, open back and forth jump, and rock paper scissor jump. | **Key Findings:** Nitrate and nitrite levels significantly increased (p ≤ 0.05). CRP, body mass, % BF, WC, SBP were significantly reduced (all, p ≤ 0.05) whilst significantly increasing lean body mass (p ≤ 0.05). DBP (DBP) did not significantly differ between groups.  **Conclusion:** Body composition, central adiposity, inflammation, and vascular function improved; suggesting that jump rope exercise is easily accessible, and cost-effective way to improve cardiovascular health and weight management. | 42.86 |
| *Tan | **Title:** Physical training of 9- to 10-year-old children with obesity to lactate threshold intensity  **Year:** 2010 **Country:** China | **Aims/Purpose:** To apply the lactate threshold concept to develop a more evidence-informed exercise program   **Sample Size:** 60  **Participants:** Obese children aged 9-10 years | **Duration:** 8 weeks  **Physical Activity:**  Participated in an 8-week supervised physical activity program (5 sessions/week, 50 minutes/session, total of 40 sessions) consisted of 5-minute warm-up (walking, jogging, and gentle stretching activities); 40-minute physical activities included running, jumping, squatting, crawling, and aerobic dance (5-6 minutes/bout, 2 minutes rest between exercise bouts); 5-minute cool-down (light activities). Children were asked to exercise up to and to try to maintain their HR at lactate threshold during the bouts. | **Key Findings:** BMI, skinfold, WC, CVF, running and jumping ability, as well as BMI showed significantly improved in the training group, while the control group observed significant increases in skinfolds and 5-minute run-walk distance.  **Conclusion:** Physical program with exercise intensity determined by HR produced significant benefits on body composition and functional capacities of obese children at 9–10 years old. The outcome provided the first-hand evidence to determine exercise intensity of physical training for children with obesity. | 71.43 |
| *Taylor | **Title:** Outcome of an exercise and educational intervention for children who are overweight  **Year:** 2005 **Country:** USA | **Aims/Purpose:** To investigate the outcome of an education- and exercise- based intervention  **Sample Size:** 41  **Participants:** Overweight children with mean age 10.5 years | **Duration:** 8 weeks  **Physical Activity:** The group intervention was scheduled twice weekly for 60-minute sessions and comprised both exercise and educational components. The exercise portion was conducted by a physical therapist and incorporated aerobic, stretching and strengthening routines using therapy balls, weights and resistance bands.   **Diet/Nutrition + Healthy Lifestyle Education:** The educational component covered a wide range of topics including risk of obesity, the energy balance model, the food pyramid, reading food labels, moderation and proportion of a serving, dining out, and strategies to stay motivated. | **Key Findings:** Significant differences in BMI (p=0.0001), WC (p<0.0001), hip girth (p<0.0001), SBP (p=0.0006), DBP (p=0.0181). No significant difference waist-hip ratio or distance walked in 6-minute walk test.  **Conclusion:** Childhood obesity is a pressing issue, yet few intervention programs exist for children as they struggle to manage their weight. Physical therapists with knowledge of exercise prescription in collaboration with other healthcare providers may provide a valuable service to these children and their families. | 85.71 |
| Telford | **Title:** PE and blood lipid concentrations in children: the LOOK randomized cluster trial  **Year:** 2013 **Country:** Australia | **Aims/Purpose:** To determine the effect of a modern elementary school PE program on the blood lipid concentrations   **Sample Size:** 708  **Participants:** Children from elementary schools | **Duration:** 4 years  **Physical Activity:** The PE intervention was provided by the not-for-profit Bluearth Foundation. 2x 50-minute classes of PE was replaced to meet current curriculum requirement of 150 minutes/week. | **Key Findings:** Significantly lower incidence of elevated LDL-c (p =0.02). Intervention effect across mean LDL-C in the boys (p =0.01) but not girls (p =0.2). Boys but not girls, improved daily moderate physical activity (p = 0.02), but no change in VPA (p =0.3) and CRF (p=0.3), %BF (p = 0.2), and total daily energy or macronutrient intake (all, p=0.3).  **Conclusion:** Well-designed specialist-delivered PE in elementary school can reduce the incidence of elevated LDL-c. | 57.14 |
| Ten Hoor | **Title:** Strength exercises during PE classes in secondary schools improve body composition: a cluster randomized controlled trial  **Year:** 2018 **Country:** Netherlands | **Aims/Purpose:** To investigate the efficacy of strength exercises in combination with monthly motivational lessons on the body composition and activity level   **Sample Size:** 695  **Participants:** Children aged 11-15 years | **Duration:** 1 year  **Physical Activity:** The intervention group received both a strength exercise intervention and a motivational intervention to promote after school physical activity, while the control group continued with their usual curriculum. The PE teachers in the intervention group spend at least 30% of the PE lessons on strength exercises (approximately 15–30 minutes per lesson – students have 3 hour of PE per week).  **Healthy Lifestyle Education:** Once a month, a 1-hour lesson was used to increase motivation to be more physically active. These motivational lessons were based on motivational interviewing. In the first five months, an extra monthly online motivational lesson was given. | **Key Findings:** FM (p=.007) and daily physical activity (p = .049) decreased. No differences in sedentary behaviour (p = .715), light physical activity (p = .833). A significant difference in MVPA minutes/day (p = .046).  **Conclusion:** An interdisciplinary and evidence-based program may not be a direct solution to combat obesity but may help in the long term with the prevention of obesity related health issues. | 71.43 |
| Thivel | **Title:** Effect of a 6-month school-based physical activity program on body composition and physical fitness in lean and obese school children  **Year:** 2011  **Country:** France | **Aims/Purpose:** To explore the effect of a 6-month school-based physical activity intervention on obese and lean children’s body composition and physical fitness  **Sample Size:** 457  **Participants:** Children aged 6-10 years | **Duration:** 6 months  **Physical Activity:** A physical activity program consisted of 120 minutes (2 times for 60 minutes) of supervised physical exercise in addition to 2 hour of PE classes per week. The sessions consisted of a 10-minute warm-up followed by psychometric activities and exercises to improve coordination, flexibility, strength, speed, and endurance. | **Key Findings:** In lean subjects, weight (p<0.001) and BMI (p< 0.05), WC, FFM, and skinfolds were significantly different. Cycling peak power (p<0.001), CRF (p<0.001), HR end (p<0.05), HR rest (p<0.001), HRR (p<0.001) were significantly different. In obese children, weight was significantly higher (p<0.05), while BMI (p<0.05), skinfolds (p<0.01), and FFM (p<0.001) differed. Cycling peak power (p<0.01), CRF (p<0.001), HR rest, and HRR (p<0.001) significantly improved.  **Conclusion:** Results provide positive anthropometric improvements, aerobic and anaerobic physical fitness. | 71.43 |
| *Togashi | **Title:** Effect of diet and exercise treatment for obese Japanese children on abdominal fat distribution  **Year:** 2010 **Country:** Japan | **Aims/Purpose:** To investigate the influence of a combination of exercise treatment corresponding to the fitness level of individual children and dietary treatment   **Sample Size:** 33  **Participants:** Obese children aged 7-14 years | **Duration:** 3 months  **Physical Activity:** Practiced Radio Gymnastics, 15-minute walk in the morning, 20-minute step exercise (light dumbbell and 10 cm platform), and 10-minute resistance exercise (body weight). Played basketball once/week and aerobics twice/week (1 hour). The exercise intensity was adjusted to the HR 50% VO2max.  **Diet/Nutrition:** Menus for dietary treatment were prepared by a dietitian based on the degree of obesity (calories controlled 75%–80% of recommended; 1400–1900 kcal/day; 55% carbohydrate, 20% protein, and 25% fat). | **Key Findings:** Significant increase in height and decrease in weight (all, p<0.001). % BF reduced significantly (p<0.001), with no significant difference in FFM. Abdominal subcutaneous and visceral fat, liver function indices and uric acid decreased (p<0.001). Serum lipids, TG, TC, fasting insulin significantly decreased, but HDL-C remained unchanged.  **Conclusion:** Results observed decreased TG, TC, insulin, liver function indices and uric acid, suggesting lipid, glucose metabolism, renal and liver functions improvement. | 57.14 |
| Tucker | **Title:** FitKids360: design, conduct, and outcomes of a stage 2 paediatric obesity program  **Year:** 2014 **Country:** USA | **Aims/Purpose:** To describe the methodology of FitKids360 and provide outcomes in patients who have completed the program  **Sample Size:** 258  **Participants:** Obese children aged 5-16 years | **Duration:** 7 weeks  **Physical Activity:** Physical activity is performed intermittently throughout the session for a total of 30–60 minutes.   **Diet/Nutrition + Healthy Lifestyle Education:** “Healthy counts” (8-7-6-5-4-3-2-1-0) are the cornerstone of the FitKids360 curriculum: 8 to 11 hours of sleep every night; 7 breakfasts every week; 6 home-cooked meals around the table every week; 5 servings of fruit and vegetables every day; 4 positive self-messages /day; 3 servings of low-fat dairy /day; 2 hours or less of screen time /day; 1 hour or more of physical activity /day; 0 sugar-sweetened beverages /day.  **Family-Based:** Buddy Program to help children and their families translate knowledge from weekly sessions into lasting behaviour changes. A “Buddy” is a case manager and mentor. | **Key Findings:** Increased MPVA (p=0.019). Reduced screen time (p<0.001), TV viewing (p<0.001), video game playing (p=0.027). Significant changes in reported nutrition behaviours (p<0.001) and an increase in the frequency of consumed whole grains (p<0.001), fruits and vegetables (p=0.017). Increased height (p<0.001) and weight (p=0.030) with significant reduction in BMI (p=0.011).  **Conclusion:** FitKids360 program observed improvements in physical activity, nutrition, and sedentary behaviours, as well as BMI. | 57.14 |
| *Vajda | **Title:** Effects of 3 hours a week of physical activity on BF and cardio-respiratory parameters in obese boys  **Year:** 2007 **Country:** Hungary | **Aims/Purpose:** To analyse the effects of a 20-week aerobic exercise program without dietary and lifestyle changes on body composition and cardiorespiratory functions   **Sample Size:** 49  **Participant:** Obese children aged 10 years | **Duration:** 20 weeks  **Physical Activity:** 2xcurricular PE classes (45 minutes) and had 3x extracurricular aerobic physical activity sessions (60-minutes) on Mondays (swimming and water games), Wednesday (folk dance) and Fridays (soccer).   **Healthy Lifestyle Education:** Every first Monday of the month, a paediatric psychologist met the children following the activity program and discussed the possible consequences and risks of their body composition and also of the benefits of their increased physical activity. | **Key Findings:** The change in BMI, BF was not significant. Difference in initial exercise physiological variables were not significant  **Conclusion:** The program was considered successful despite that the changes in the observed physiological and physical indicators appeared to be slight. | 42.86 |
| Van Middelkoop | **Title:** A multidisciplinary intervention program for overweight and obese children in deprived areas  **Year:** 2017 **Country:** Netherlands | **Aims/Purpose:** To evaluate the effectiveness of the Kids4Fit intervention on child’s weight status  **Sample Size:** 154  **Participants:** Children aged 6-12 years | **Duration:** 12 weeks  **Physical Activity:** 18 group sessions with a physiotherapist to increase activity and stimulate participation in sports. Focused on fitness, strength and included different types of sport activities. The first 6 weeks consisted of (twice/week, 1-hour) an indoor sport hall. The final 6 weeks of the program consisted of (once/week, 1-hour) a sport of their choice.  **Diet/Nutrition:** All children attended 4x1-hour group sessions with the dietitian focused on healthy eating behaviour, importance of breakfast, avoiding sugared drinks, limited use of television or computer and daily physical activity.  **Healthy Lifestyle Education:** All children consulted the child psychologist for 4x1-hour group sessions, aimed to support the nutritional and exercise advice and improve the child’s self-image. All parents attended 4x1-hour group sessions with the child psychologist focused incorporate healthy lifestyle and their position as a role model was addressed. | **Key Findings:** Significant reduction in WC (p=0.0166). Significant reduction in BMI-z (p=0.0015) and WC (p=0.0275) in not complaint compared to the waiting list expectancy. About 77% of the children had breakfast 7 days a week and this increased to 81% at 12 weeks.  **Conclusion:** A local multidisciplinary intervention program in deprived areas is effective in reducing WC of obese children, compared to a waiting list expectancy, but no significant changes in lifestyle and quality of life were shown. | 28.57 |
| *Vasconcellos | **Title:** Health markers in obese adolescents improved by a 12-week recreational soccer program: a randomised controlled trial  **Year:** 2016 **Country:** Portugal | **Aims/Purpose:** To investigate the impact of a 12-week recreational soccer program upon the body composition, biochemical risk markers for CVD, CRF, cardiac autonomic activity, and endothelial function  **Sample Size:** 30  **Participants:** Children aged 12-17 years, BMI>2SD and non-obese | **Duration:** 12 weeks  **Physical Activity:** Performed 3 times a week during 12 weeks from 8.30 to 9:30 am (Mondays, Wednesdays, and Fridays). Each session consisted of 10-minute warm-up followed by 40 minutes of games performed in small pitch areas (such as 2 vs. 2, 3 vs. 3 and 4 vs. 4) and 10-minute cool-down. | **Key Findings:** Significant difference in weight, BMI, and WC (p<0.01). Significant decrease in %BF (p<0.001). VO2max increased (p<0.001) significantly in the intervention. No significant change for maxHR, oxygen consumption at rest, and WC-height ratio. SBP was significantly lower in the intervention no changes were found for DBP or mean blood pressure. Significant increase in HDL-c and decrease in TC, TG, CRP, fasting glucose, and IR. No difference for basal blood flow (p=0.347), basal vascular conductance (p=0.237), blood flow after ischemia (p=0.562), blood flow after nitroprusside (p=0.487), and vascular conductance after nitroprusside (p=0.621).  **Conclusion:** 12-week recreational soccer intervention can improve body mass, body composition, blood pressure, cardiac autonomic activity, physical fitness, biochemical markers, and endothelial function. These results are original and reinforce the importance of sports practice by overweight and obese adolescents. | 85.71 |
| *Vignolo | **Title:** Five-year follow-up of a cognitive-behavioural lifestyle multidisciplinary program for childhood obesity outpatient treatment  **Year:** 2008 **Country:** Italy | **Aims/Purpose:** To examine the 5-year follow-up results of MI PIACE PIACERMI (I like to like and please myself)   **Sample Size:** 31  **Participants:** Obese children aged 6-12 years | **Duration:** 5 year follow up  **Physical Activity:** MI PIACE PIACERMI interventions consist of play-based exercise programs of progressive intensity, to increase motivation in physical activity and to improve body movement awareness and postural control.  **Healthy Lifestyle Intervention:** The basic objective of treatment is the progressive adoption of behaviours contributing to reaching a healthy lifestyle through the reduction of the importance given to weight and the promotion of the child’s self-control and self-efficacy. The program favours the acquisition of cognitive abilities to face ambivalent and conflictual circumstances and the development of abilities to control eating behaviour and physical activity. At the same time, lifestyle modifications aimed at reducing sedentary behaviours of both child and family are proposed and encouraged.   **Diet/Nutrition:** MI PIACE PIACERMI is also based on nutritional education, that is, general information on foods and their importance for health, food preparation and eating habits. It is based on the concept of ‘moderation’ that is both ethically relevant and makes it possible to simplify most of the problems.   **Family-Based:**  It actively involves parents and family members, who have to change their behaviour and lifestyle to favour an essential social support and to influence their child’s behaviour through parental ‘modelling’. | **Key Findings:** Significant BMI-z, BMI, WC reduction. Mean body weight increased. Total energy intake significantly reduced but macronutrients did not change. Consumption of fruit and vegetables increased significantly. Frequency of eating habits associated with overweight and obesity, decreased significantly. Improved relationships and participation in activities with their peers, with increased self-esteem.  **Conclusion:** MI PIACE PIACERMI demonstrated high compliance in children and parents with generally positive feedback about the program and improvements in health, psychological and behaviours aspects related to obesity. | 71.43 |
| Villa-Gonzalez | **Title:** Effects of a school-based intervention on active commuting to school and health-related fitness  **Year:** 2017 **Country:** Ecuador | **Aims/Purpose:** To investigate the effects of a school-based intervention on active commuting to school and health-related fitness  **Sample Size:** 494  **Participants:** Children aged 8-11 years | **Duration:** 6 months  **Physical Activity + Family-Based:** The intervention focused on increasing the frequency of active commuting to and from school among children. Children participated in 6 monthly activities (60–120 minutes) during school hours in addition to their regular PE lessons. The intervention included: introductory activities such as a questionnaire on the mode of commuting to school reported by families (parents or grandparents), reading a story and performing scenes related to active commuting to school, activity on knowledge about the environmental characteristics around the school, activity on road safety, activity on behaviours in the street, activity on traditional games. | **Key Findings:** No significant change on frequency of active commuting and modes of commuting. Significant change in all health-related fitness tests for VO2max, 20-m shuttle run, long jump test.  **Conclusion:** Results demonstrated increased rates of cycling to school among boys but was not effective on increasing rates of walking to school and health-related fitness. However, it did avoid increased rates of passive commuting in the experimental group, which were significantly increased in control girls. | 57.14 |
| Vissers | **Title:** Effect of a multidisciplinary school-based lifestyle intervention on body weight and metabolic variables in overweight and obese youth  **Year:** 2008 **Country:** Belgium | **Aims/Purpose:** To study the effect of a multidisciplinary school-based lifestyle intervention   **Sample Size:** 869  **Participants:** Children aged 16-19 years | **Duration:** 6 months  **Physical Activity:** Monthly counselling session with a physiotherapist to increase daily physical activity and a free subscription to a nearby fitness club. Work out sessions (>3 times/week) consisted of aerobic and strength exercises including: short warming up and stretching; aerobic exercises running such as treadmill, cycling, rowing, stepping; strength exercises focused on large muscle groups such as pectoral muscles, upper arm muscles, abdominal muscles and muscles of the legs and a cool down.   **Diet/Nutrition + Healthy Lifestyle Education:** Participants were offered nutritional counselling by a dietitian, individually or in a group, (once a month) to discuss healthy food choices and maintaining a proper energy balance, breakfast, snacks, drinks, dairy products, fast food, portion sizes, nutritional labelling, motivation and coping strategies. | **Key Findings:** Significant difference in weight and BMI over 6 months between groups (p<0.001). WC decreased (p =0.058). Significantly improved fasting glucose (p <0.005)  **Conclusion:** This approach appears promising approach in the reduction of body weight and improvement of aspects of the metabolic syndrome. | 57.14 |
| Walther | **Title:** Effect of increased exercise in school children on physical fitness and endothelial progenitor cells: a prospective randomized trial  **Year:** 2009 **Country:** Germany | **Aims/Purpose:** To examine whether additional school exercise would improve VO2max, BMI, motor and coordinative abilities, progenitor cells, and HDL-c  **Sample Size:** 182  **Participants:** Children aged 10-12 years in sixth grade | **Duration:** 1 year  **Physical Activity:** 1 unit of physical exercise (45 minutes) with at least 15 minutes of endurance training per school day was assigned. In addition to the randomized classes, 2 additional classes focused on competitive sports.   **Healthy Lifestyle Education:** Lessons on healthy lifestyle were included once monthly | **Key Findings:** Motor abilities improved in both groups and were stronger in the intervention despite lack of significance.  **Conclusion:** Results demonstrate improvement in CVF, progenitor cells, trend toward improvement in motor abilities and reduction in BMI-z. Longer-term observations will be required to determine whether improved attitudes toward physical activity, improved exercise, and decreased CVD risk will occur. | 71.43 |
| *Wang | **Title:** Evaluation of a comprehensive intervention with a behavioural modification strategy for childhood obesity prevention: a nonrandomized cluster-controlled trial  **Year:** 2015 **Country:** China | **Aims/Purpose:** To develop, implement and evaluate a comprehensive intervention with a combination of diet, physical activity, and cognitive behavioural modification strategies  **Sample Size:** 438  **Participants:** Overweight and obese children aged 7-12 years | **Duration:** 1 year  **Physical Activity:** Children were encouraged to gradually increase their physical activity levels with a 20 % increment to reach the overall recommended levels and to reduce unhealthy diet behaviours at each intervention cycle. Various grade-specific and space-appropriate physical activities for the classroom and were integrated into academic curriculums (e.g., math, science, languages, art). 2x “Happy 10” sessions were conducted per school day. Activity cards were used to illustrate how to perform the activities, and tracking posters and stickers were used in the classroom to follow progress.   **Diet/Nutrition + Family-Based:** The nutrition intervention was based on health education lectures given by researchers in the classroom and focused on nutrition and health knowledge, including the components of food nutrients, the importance of eating breakfast, the benefits of fruit, vegetables and water intake, and how to choose healthy snacks. Lectures were delivered eight times to students and twice to parents. Each lecture lasted a minimum of 40 minutes. Furthermore, ‘Dietary Pyramid for Chinese people’ posters were displayed on the walls of all participating classrooms. Cartoon handbooks containing all of this information were distributed to all participants in the nutrition education group to help clarify the concepts presented in the lectures.   **Healthy Lifestyle Education:** Health promotion regarding the benefits of physical activity, and the harm caused by sedentary lifestyles, was delivered to students and parents in 2 health education lectures. | **Key Findings:** Dietary behaviours improved particularly in the frequencies of breakfast and eating out. Children reported higher physical activity levels. No significant effects in BMI, WC (all, p>0.05). Reductions in SBP, DBP (p<0.001). Significant treatment effects over time in TC, TG, HDL-C, and blood glucose among the four groups (p<0.05).  **Conclusion:** Results generated improvements in body composition, blood pressure, BMI, WC and fasting glucose compared with physical activity only or diet only interventions. Lack of improvement in biochemical lipid metabolism indicators. Future studies should implement these interventions among other high-risk populations within randomized clinical trials and long-term follow-up studies to elaborate potential effectiveness. | 100 |
| Wanless | **Title:** Pedometers and aerobic capacity: evaluating an elementary after-school running program  **Year:** 2014 **Country:** USA | **Aims/Purpose:** To assess the impact of a pedometer-focused physical activity program on aerobic capacity  **Sample Size:** 24  **Participants:** Children aged 8-12 years | **Duration:** 12 weeks  **Physical Activity:** The program (2 days/week, 1-hour sessions) was based upon a series of progressive walking/jogging workouts and physical activity centred games and activities. Pedometers were used. The participants engaged in a warm-up activity (e.g., Kangaroo Tag) and stretching; group discussion involving a “running tip” (e.g., controlled breathing); prescribed running workout (e.g., 10-minute interval run); group activity (e.g., scavenger hunt); cool-down. In addition, home workouts were provided to encourage participants to engage in physical activity at home and to promote exercise as a family activity. A daily step goal was provided, and each participant was instructed to make daily pedometer step goals. | **Key Findings:** Significant differences between the PACER pre and post-test (p≤ 0.001). Results indicate improvement in aerobic capacity. No significant relationship between the difference in individual step count and PACER pre- and post-test (r=0.318, p= 0.130).  **Conclusion:** Results demonstrate improved fitness through pedometers as motivation to succeed. In this current study, the aerobic capacity of the students was improved even though pedometer step count was not a predictor. | 57.14 |
| Webber | **Title:** Cardiovascular risk factors among children after a 2 1/2-year intervention-The CATCH Study  **Year:** 1996 **Country:** USA | **Aims/Purpose:** The purpose of this paper is comparison between the intervention and control groups  **Sample Size:** 4019  **Participants:** Children in third grade | **Duration:** 2.5 years  **Physical Activity + Diet/Nutrition + Healthy Lifestyle Education:** The overall goal was to alter children's dietary and physical activity behaviours and prevent the onset of tobacco use. Dietary goals included reducing total fat in school food service to 30% of energy, saturated fat to 10%, sodium intake by 25% and increasing MVPA in PE class to 40% of the class period. | **Key Findings:** For BMI, a statistically significant ethnicity by intervention group interaction was noted (p<0.02). No change in BMI for Caucasian and Hispanic children and an increase in BMI For African American. SBP and DBP levels were higher at follow-up. No change in HR and serum TC.  **Conclusion:** CATCH positively affected the school environment and the health behaviours of the children. Although these changes did not translate to significant changes in risk factors at these ages, they have the potential for promoting positive health behaviours into adulthood. | 14.29 |
| *Whipp | **Title:** The effect of caloric restriction and physical training on the responses of obese adolescents to graded exercise  **Year:** 1971 **Country:** USA | **Aims/Purpose:** To study cardiopulmonary and metabolic responses of obese adolescents to weight supported exercise and also study the effect of weight loss and physical training on these responses  **Sample Size:** 7  **Participants:** Obese children aged 13-16 years | **Duration:** 6 weeks  **Physical Activity:** A program of physical exercise was prescribed. This consisted of daily periods of swimming, volleyball, hiking and supervised calisthenics.  **Diet/Nutrition:** The subjects were restricted to a daily intake of 1000 C. The diet was mixed with respect to carbohydrate, fats, and protein. | **Key Findings:** Results observed significant decreased weight by 9.2% (p<0.01), decreased oxygen cost of work. HR improved at rest and during exercise. SBP slightly reduced at rest but significantly reduced (p<0.01) during exercise. DBP reduced slightly, but not significantly (p>0.05).  **Conclusion:** Results demonstrated reduced weight, improved physical fitness and work rate. | 28.57 |
| Willi | **Title:** Cardiovascular risk factors in multi-ethnic middle school students: the HEALTHY primary prevention trial  **Year:** 2012 **Country:** USA | **Aims/Purpose:** To examine the effects of an integrated, multi-component, school-based intervention program on CVD risk factors  **Sample Size:** 4363  **Participants:** Children aged 11-12 years | **Duration:** 2.5 years  **Physical Activity + Diet/Nutrition + Healthy Lifestyle Education + Environment:** The intervention program consists of changes to the total school food environment and PE classes. These modifications were enhanced by educational outreach and behaviour change activities and promoted by a social marketing campaign consisting of reinforcing messages and images. | **Key Findings:** Proportion of Black males with pre-hypertension fell from 10.8% to 6.4%. White males observed reduction in the hypertension, which dropped from 11.5% to 6.5%. No significant effect on blood pressure in Hispanic youth. SBP was higher in boys than girls, while total LDL-c, and HDL-c were higher in boys at baseline but lower than girls post-intervention.  **Conclusion:** Results observed reducing rates of overweight/obesity despite lack of meaningful effect on other CVD risk factors. Specific subgroups may benefit from such programs. | 57.14 |
| *Wong | **Title:** Effects of a 12-week exercise training program on aerobic fitness, body composition, blood lipids and C-reactive protein in adolescents with obesity  **Year:** 2008 **Country:** Singapore | **Aims/Purpose:** To examine the effects of a training program which combined various forms of aerobic activities, resistance training, sports and games, and stair-climbing exercises, on aerobic fitness, body composition, serum CRP and lipid profile  **Sample Size:** 24  **Participants:** Obese male children aged 13-14 years | **Duration:** 12 weeks  **Physical Activity:** PE sessions (2 sessions/week, 40 minutes) and exercise program (2 session/week, 45-60 minutes) comprised circuit based aerobic exercises, strength conditioning and/or resistance training, and game activities such as soccer, handball, stair-climbing exercises (65-85% maxHR). Indoor activities were conducted in the school’s weight training gymnasium (20-35 minutes) included: circuit-based combination of light resistance training with 4-7 resistance stations, using body-weight (sit-ups, push-ups, chin-ups, squats) and eventually progressed to using medicine balls (varied weights from 2 to 5 kg) (simple press-up activities, variations of passing and tossing the medicine ball to partners, using legs to manipulate medicine ball movements along the ground), alternated with 3 to 5 aerobic stations (cycle ergometry and/or treadmill walking). The number of resistance exercise circuits gradually increased from 1 to 3 sets, 8 to 25 repetitions, and then by increasing resistance or load. The exercise period for the resistance stations gradually increased from 1 to 3 minutes, while the exercise period for each aerobic station lasted from 5 to 10 minutes depending. Likewise, the cycling load for the aerobic stations was also gradually increased to accommodate for individual improvement made. | **Key Findings:** Significant BMI reduction (p <0.05) and increase in lean body mass (p <0.05). No significant difference in FM and %BF. All blood lipids variables fell within the clinically specified normal ranges, except for CRP concentrations that were slightly elevated above the healthy normal range. Significant drop in SBP, resting HR (all, p<0.05).  **Conclusion:** Results demonstrate improvement in aerobic fitness. However, it is felt that this dose is insufficient to result in improvement on the parameters related to adiposity and risk factors for cardiovascular and metabolic diseases. Additional exercise should incorporate resistance or strength in order achieve greater energy expenditure essential to prevent further weight gain, if not to achieve substantial weight loss. | 57.14 |
| *Wright | **Title:** Impact of a nurse-directed, coordinated school health program to enhance physical activity behaviors and reduce BMI among minority children: a parallel-group, randomized control trial  **Year:** 2013 **Country:** USA | **Aims/Purpose:** To evaluate the impact of a nurse directed, coordinated, culturally sensitive, school-based, family-centred lifestyle program on activity behaviours and BMI  **Sample Size:** 251  **Participants:** Children aged 8-12 years with BMI >85^th^ percentile | **Duration:** 6 weeks  **Physical Activity:** The physical activity component (45 minutes) reduced sedentary behaviours. Students learned exercise in a non-structured program, including warm-up and stretching, basketball, soccer, Hip Hop and Salsa dancing, relay race activities, jump rope, power walking, and running.   **Diet/Nutrition:** Children and parents (45 minutes) had nutrition education/behavioural modification sessions. Emphasis on improving drink and food choices, lowering dietary fat intake, decreasing total calories, food pyramid, reduce total and saturated fat and cholesterol intake, limit carbohydrate intake, portion control, improving the quality of snacks, strategies for eating out, understanding food labelling, and healthy food shopping.  **Healthy Lifestyle Education:** Student psychological counselling was offered for all children who requested it or were identified as having a need for counselling.  **Environment:** Wellness policies included the availability of foods lower in energy density, with an emphasis on fruits, vegetables, whole grains and low-fat dairy, 100% fruit juice, low fat or non-fat milk and the elimination of sugary drinks such as sports drink and sodas. PE classes during school reflected policies that enforced the recommended types of moderate to physical activities.  **Family-Based:** Parents were taught the implications of obesity in children and adults, and the importance of healthy lifestyles. Parents participated in a parent support group, moderated by a registered nurse where they were able to discuss their challenges and success stories regarding diet and exercise modification. | **Key Findings:** Females observed BMI (p=0.047), BMI z-score (p=0.05) improvements. Increased participation of at least 60 minutes of physical activity /day was sustained in males (p=0.002) and females (p=0.005). Both sexes observed increased PE class attendance which was sustained for both males (p=0.003) and females (p=0.002). TV viewing significantly decreased for both sexes but only sustained at 12 months for males (p=0.030).  **Conclusion:** Results observed decreases BMI z-scores in females, decreased TV use in males; and increased daily physical activity and PE class attendance for both sexes, in high-risk school-age children Recognizing gender inequalities may allow for the development of more effective health promotion strategies. | 42.86 |
| *Yetgin | **Title:** The influence of physical training modalities on basal metabolic rate and leptin on obese adolescent boys  **Year:** 2018 **Country:** Istanbul | **Aims/Purpose:** To compare the effects of structured six-month resistance versus endurance-training programs on leptin, maximum oxygen consumption and basal metabolic rate   **Sample Size:** 16  **Participants:** Obese children aged 15-18 years | **Duration:** 6 months  **Physical Activity:** 3 sessions per week (3 days/week, 60 minutes/day). For the endurance exercise prescription, exercise intensity was designed for an intensity of 50- 60% for the first 2 months, 60- 70% for the third and fourth months, and for the fifth and sixth months at 70- 75% maxHR. Accordingly, the appropriate resistance exercise program for each individual was established by calculating 50% - 60% of 1RM during the first 2 months, 60% - 70% of 1RM during the second 2 months, and 70% - 75% of 1RM during the last 2 months  **Diet/Nutrition:** A dietitian provided counselling and recommendations on balanced nutrition but a structured diet program was not implemented throughout the study. | **Key Findings:** Significant differences between pre- and post-test values for leptin (p<0.05). RMR increased significantly (p<0.05). The post-test values for VO2max increased significantly in both groups (p<0.05)  **Conclusion:** Our results indicate that both endurance and resistance exercises without caloric restriction were effective in reducing % BF, increasing VO2max and decreasing leptin levels in obese adolescent boys. RMR increased only by endurance training. | 57.14 |
| Yin | **Title:** The impact of a 3-year after-school obesity prevention program in elementary school children  **Year:** 2012 **Country:** USA | **Aims/Purpose:** To report the results of the Medical College of Georgia FitKid Program and its effect on %BF and CRF   **Sample Size:** 574  **Participants:** Children in third grade | **Duration:** 3 years  **Physical Activity + Healthy Lifestyle Intervention:** The program was offered daily following the completion of regular school activities. Children were encouraged to attend at least 3 days/week to provide flexibility for them to attend other after-school activities. The 120-minute structured after-school program began with 40 minutes for snacks and teacher-assisted homework and academic enrichment activities in a classroom. On Fridays, a lesson was provided with a health-related focus. The next 80 minutes consisted of 20-minute skill-based physical activity that incorporated skill instructions, 40-minute VPA that used developmentally appropriate activities with a monthly theme, and 20-minute stretching/resistance training and cool down. The intensity goal for the 40-minute VPA portion was to reach a HR of ≥150 bpm. | **Key Findings:** Significant treatment by time interactions for % BF, CRF, and WC. % BF, CRF, and WC showed improvement in intervention group but rebounded to levels similar to those of the control group during summer months.  **Conclusion:** Results demonstrated that without dietary intervention, beneficial results were observed. However, these results were lost during the summer breaks. | 42.86 |
| *Zguira | **Title:** Effect of an 8-Week Individualized Training Program on Blood Biomarkers, Adipokines and Endothelial Function in Obese Young Adolescents with and without Metabolic Syndrome  **Year:** 2019 **Country:** Tunisia | **Aims/Purpose:** To investigate the effect of an 8-week individualized physical training program on endothelial function, blood biomarkers and adipokine levels  **Sample Size:** 122  **Participants:** Obese children aged 12-16 years, with and without metabolic syndrome | **Duration:** 8 weeks  **Physical Activity:** The design of the individualized training intervention was based on our previous studies. The individualized training intervention was performed within the usual 90-minutes of supervised activity /day at a HR that corresponded to Lipoxmax, 3 days per week, during the 8-week intervention period. | **Key Findings:** Body mass (p = 0.02), WC (p < 0.0001) decreased. Lipoxmax and rate of fat oxidation increased significantly (p<0.0001). Decreased blood glucose, TG, TC, LDL-c, leptin levels (all, p<0.05).  **Conclusion:** Results observed decreased glucose, TG, TC, LDL and leptin in obese with and without MS.  Adiponectin, endothelial dependent vasodilator increased. | 57.14 |
| *Zhang | **Title:** Aerobic exercise improves endothelial function and serum adropin levels in obese adolescents independent of body weight loss  **Year:** 2017 **Country:** China | **Aims/Purpose:** To investigate the effect of an exercise intervention on lipid metabolism, vascular endothelial function and serum adropin levels   **Sample Size:** 50  **Participants:** Obese children aged 16-19 years | **Duration:** 12 weeks  **Physical Activity:** Obese adolescents in the health promotion class received 12 weeks of aerobic exercise. The project gave priority to jogging and assisted in other sports, including badminton, table tennis, aerobics and cycling. All candidates trained 3–5 times/week for 90 minutes/session. | **Key Findings:** Results observed significant decreased weight, BMI, WC, waist-hip ratio and FM. SBP decreased (p<0.01), but DBP was not different post-intervention (p=0.601). Alanine aminotransferase, blood urea nitrogen and creatinine levels significantly reduced. TC, LDL-C, glucose, fasting insulin, and IR.  levels significantly reduced, and HDL-C was increased.  **Conclusion:** Our study demonstrated that 12 weeks of exercise intervention increased the serum level of adropin and improved endothelial function. | 85.71 |
| Zhou | **Title:** A policy-driven multifaceted approach for early childhood physical fitness promotion impacts on body composition and physical fitness in young Chinese children  **Year:** 2014 **Country:** China | **Aims/Purpose:** This study tested the effectiveness of a multifaceted intervention that integrated childcare centre, families, and community to promote healthy growth and physical fitness in preschool Chinese children  **Sample Size:** 357  **Participants:** Children aged 3-5 years | **Duration:** 12 months  **Physical Activity:** Daily outdoor play (morning 30 minutes, afternoon 30 minutes, for 3-years-old classes; morning 60 minutes, afternoon 30 minutes, for 4- and 5-years-old classes). Exercise routine (10 minutes) during morning recess.  **Diet/Nutrition:** Increased the quality of food to meet the nutrition regulations and to increase healthy eating choices.  **Healthy Lifestyle Education + Family-Based:** Intervention activities include: monthly health education seminars with parents on topics of child physical development, gross motor skill acquisition, family oriented physical activities, nutrition and healthy food, monitoring physical fitness, outdoor physical activities, common children’s illness and disease prevention, and promotion of emotional health; 12 monthly newsletters with tips on developing children’s health habits; making of a simple play equipment by child and parents; an interactive internet website that provided parents with updates on their child’s changes in physical fitness status and individualised feedback on physical activity and healthy eating and information related physical activity, nutrition and obesity; and family events organized by the childcare centre  **Environment:** All childcare teachers participated in a 20-hour training (bi-weekly, 60-minute sessions) on teaching PE. Food service workers received 2 training sessions, 3 hours each. The implementation of the outdoor physical activity curriculum was closely monitored for quality of the lessons and the amount of physical activity. The childcare centre received child-safe, portable play equipment that was used in implementing the PE curriculum. | **Key Findings:** Significant increases in weight (p<0.02), height (p<0.01), and MM (p<0.0001) and significant decreases in %BF (p<0.0001), FM (p<0.0001). Significant improvements in 20-meter agility run, broad jump (p<0.0001), tennis ball throwing (p<0.006), sit and reach (p<0.03), balance beam walk p<0.0001), 30-meter sprint (p<0.02), and 20-meter crawl (p<0.0001).  **Conclusion:** Results demonstrate significant improvement in body composition, fitness. Future plans include making outdoor play curriculum and intervention materials to facilitate replicability. | 100 |
| *Zorba | **Title:** Exercise training improves body composition, blood lipid profile and serum insulin levels in obese children  **Year:** 2011 **Country:** Turkey | **Aims/Purpose:** To determine the effects of regular exercise on hypertension, dyslipidaemia and diabetes  **Sample Size:** 40  **Participants:** Obese children aged 10-12 years, BMI>30 | **Duration:** 12 weeks  **Physical Activity:** The subjects in the exercise group underwent a walking-jogging exercise (20-45 minutes, 3 days/week) targeted HR 60- 65%. Each training session started with a warmup (5-10 minutes) and ended with relaxation exercise (5-10 minutes). | **Key Findings:** Significant improvements in weight, BMI values, blood lipids and serum insulin, decreased insulin, circumference measure of forearm, elbow, calf, knee, waist, chest and hip. Improvements also in TC, TG, LDL-c and VLDL-c levels and elevated HDL-c.  **Conclusion:** Results demonstrate maintenance of weight, reduction of LDL-c, elevation of HDL-c. Future studies should continue treating with an emphasis towards increasing regular physical activity with supported dietary interventions. | 85.71 |

*Note. Abbreviations* *-overweight/obesity management intervention, p- p-value SBP- systolic blood pressure, DBP- diastolic blood pressure, %- percentage, BF- body fat, TC- total cholesterol, LPA- light physical activity, MVPA- moderate-vigorous physical activity, VPA- vigorous physical activity, kcal-kilocalorie, PE- physical education, RM- repetition maximum, BMI- body mass index, FFM- fat free mass, FM- fat mass, FMI- fat mass index, MM- muscle mass, HIIT- high intensity interval training, LIIT- low intensity interval training, WC- waist circumference, maxHR- maximal heart rate, PCOS- polycystic ovarian syndrome, LH/FSH- luteinizing hormone/follicle-stimulating hormone, bpm- beats per minute, HHR- heart rate reserve, HR- heart rate, IR- insulin resistance, rpm- revolutions per minute, TG- triglycerides, TC- total cholesterol, VLDL-c- very low density lipoprotein-cholesterol, LDL-c- low density lipoprotein-cholesterol, HDL-c- high density lipoprotein-cholesterol, apo- apolipoprotein, VT- ventilatory threshold, PTH- parathyroid hormone, VO2max- maximal oxygen consumption, MUFA- monounsaturated fatty acid, PUFA- polyunsaturated fatty acid, RMR- resting metabolic equivalent of task, METmax- maximal metabolic equivalent of task, RPE- rate of perceived exertion, HbA1c- haemoglobin A1c, CRP- c-reactive protein, CRF- cardiorespiratory fitness, CVF- cardiovascular fitness, CVD- cardiovascular disease
