# Supplementary material for: Physiotherapy and related management for childhood obesity: A systematic scoping review
Source: PLoS One. 2021 Jun 14;16(6):e0252572. doi: 10.1371/journal.pone.0252572 (PMC8202913; doi:10.1371/journal.pone.0252572)
Supplement: S7 Table — (DOCX) [file pone.0252572.s007.docx]

**S7 Table. Sensitivity Analysis**

|  | Anthropometric | | | | CRF/CVF | | | | Blood serum analysis | | | | | Coordination | | | Strength | | | | Physical activity assessment | | | | Sedentary behaviour assessment | | | Self-esteem, -efficacy and -perception assessment | | | | Food/drink behaviour and consumption | | |
| --- | --- | --- | --- | --- | --- | --- | --- | --- | --- | --- | --- | --- | --- | --- | --- | --- | --- | --- | --- | --- | --- | --- | --- | --- | --- | --- | --- | --- | --- | --- | --- | --- | --- | --- |
|  | Original Analysis | | Sensitivity Analysis | | Original Analysis | | Sensitivity Analysis | | Original Analysis | | Sensitivity Analysis | | Original Analysis | | Sensitivity Analysis | | Original Analysis | | Sensitivity Analysis | | Original Analysis | | Sensitivity Analysis | | Original Analysis | | Sensitivity Analysis | Original Analysis | | Sensitivity Analysis | | Original Analysis | Sensitivity Analysis | |
| **Quantitative focused physical activity** | | | | | | | | | | | | | | | | | | | | | | | | | | | | | | | | | | |
| Walking/  running | n/N= 13/15 (87%) D | n/N= 13/13 (100%) D | |  | |  | | n/N= 14/14 (100%) D | | n/N= 14/14 (100%) D | |  | | | |  | |  | |  | |  |  |  | |  | | |  | |  |  | |  |
| Combined aerobic + resistance exercise | n/N=  9/12 (75%) D | n/N= 7/9 (78%) D | |  | |  | | n/N=13/18 (72%) D | | n/N= 9/9 (100%) D | |  | | | |  | | n/N=  4/9 (44%) ? | | n/N=  0/0 (0%) ? | |  |  |  | |  | | |  | |  |  | |  |
| Physical education + increased physical activity/  exercise | n/N=  30/41 (73%) D | n/N= 8/14 (58%) ? | | n/N=  16/19 (84%) D | | n/N=5/6 (83%) D | | n/N (+)=  1/7 (14%) n/N (0)= 6/7 (86%) 0 | | n/N= 0/0 (0%) ? | |  | | | |  | |  | |  | |  |  |  | |  | | |  | |  |  | |  |
| Sports-based | n/N=  6/10 (60%) ? | n/N=  6/10 (60%) ? | | n/N=  3/7 (43%) ? | | n/N=  3/7 (43%) ? | | n/N= 6/8 (75%) D | | n/N= 6/8 (75%) D | |  | | | |  | |  | |  | |  |  |  | |  | | |  | |  |  | |  |
| Intensity Training | n/N=  5/6 (83%) D | n/N=  0/0  (0%)  ? | |  | |  | |  | |  | |  | | | |  | |  | |  | |  |  |  | |  | | |  | |  |  | |  |
| **Multicomponent intervention** | | | | | | | | | | | | | | | | | | | | | | | | | | | | | | | | | | |
| Physical activity + diet/nutrition education | n/N= 14/24 (58%) ? | n/N= 14/21 (67%) D | | n/N=  5/7 (71%) D | | n/N= 5/5 (100%) D | | n/N=  9/9 (100%) D | | n/N=  9/9 (100%) D | |  | | | |  | |  | |  | |  |  |  | |  | | |  | |  |  | |  |
| Physical activity + environment | n/N (+)=  3/11  (27%) n/N (0)= 8/11  (73%) 0 | n/N= 0/0 (0%) ? | |  | |  | | n/N (+)=  1/6 (17%) n/N (0)=  5/6 (83%) 0 | | n/N= 0/0 (0%) ? | |  | | | |  | |  | |  | |  |  |  | |  | | |  | |  |  | |  |
| Diet/nutrition education + healthy lifestyle education + environment | n/N=  4/8 (50%) ? | n/N=  2/2 (100%) ? | |  | |  | |  | |  | |  | | | |  | |  | |  | |  |  |  | |  | | |  | |  |  | |  |
| Physical activity + healthy lifestyle education + diet/nutrition education | n/N=  42/74 (57%) ? | n/N=  40/66 (61%) ? | | n/N=  19/29 (66%) ? | | n/N=  17/24 (71%) D | | n/N  =33/54 (61%) ? | | n/N=  23/44 (52%) ? | |  | | | |  | | n/N=  8/11 (73%) D | | n/N=  8/11 (73%) D | | n/N=  3/6  (50%) ? | n/N=2/4 (50%) ? |  | |  | | |  | |  | n/N=  3/7 (43%) ? | | n/N=3/6 (50%) ? |
| Physical activity + diet/nutrition education + environment | n/N=  11/31  (35%)  ? | n/N (+)=  4/14 (29%) n/N (0)=  10/14 (71%) 0 | | n/N=  10/17 (59%) ? | | n/N=  5/8 (63%) ? | | n/N=  9/17 (38%) ? | | n/N=  11/14 (79%) D | | n/N=3/5 (60%) ? | | | | n/N=3/5  (60%) ? | | n/N=8/9  (89%) D | | n/N=3/3  (100%) ? | |  |  |  | |  | | |  | |  |  | |  |
| Physical activity + healthy lifestyle education + diet/nutrition education + environment | n/N=  38/78  (49%) ? | | n/N=  28/45 (62%) ? | | n/N=  15/26 (58%) ? | | n/N=  8/13 (62%) ? | | n/N=  17/28 (61%) ? | | n/N=  17/28 (61%) ? | |  | |  | | n/N=  4/5 (80%) D | | n/N=  0/0 (0%) ? | | n/N=  13/21 (62%) ? | | n/N=  4/7 (57%) ? | | n/N=  7/10 (70%) D | | n/N=2/3 (67%) ? | n/N=  5/7 (71%) D | | n/N=  2/4  (50%) ? | | n/N=  7/12 (58%) ? | n/N=  3/4 (75%) ? | |

| Key: | D | Desirable | 0 | No effect | U | Undesirable | ? | Questionable |  | No data |
| --- | --- | --- | --- | --- | --- | --- | --- | --- | --- | --- |
